# Total Synthesis of Tri‐, Hexa‐ and Heptasaccharidic Substructures of the O‐Polysaccharide of *Providencia rustigianii* O34

**DOI:** 10.1002/chem.202000496

**Published:** 2020-04-28

**Authors:** Somayeh Ahadi, Shahid I. Awan, Daniel B. Werz

**Affiliations:** ^1^ Technische Universität Braunschweig Institute of Organic Chemistry Hagenring 30 38106 Braunschweig Germany; ^2^ Georg-August-Universität Göttingen Institute of Organic and Biomolecular Chemistry Tammannstraße 2 37077 Göttingen Germany

**Keywords:** fucose, glycosylation, lipopolysaccharide (LPS), oligosaccharides, *Providencia rustigianii*

## Abstract

A general and efficient strategy for synthesis of tri‐, hexa‐ and heptasaccharidic substructures of the lipopolysaccharide of *Providencia rustigianii* O34 is described. For the heptasaccharide seven different building blocks were employed. Special features of the structures are an α‐linked galactosamine and the two embedded α‐fucose units, which are either branched at positions‐3 and ‐4 or further linked at their 2‐position. Convergent strategies focused on [4+3], [3+4], and [4+2+1] couplings. Whereas the [4+3] and [3+4] coupling strategies failed the [4+2+1] strategy was successful. As monosaccharidic building blocks trichloroacetimidates and phosphates were employed. Global deprotection of the fully protected structures was achieved by Birch reaction.

## Graphical abstract

**Embedded fucoses**: First syntheses of substructures of the O‐polysaccharide of *Providencia rustiginii* O34 are presented. The repeating unit is a heptasaccharide with seven different monosacchride units (see figure).

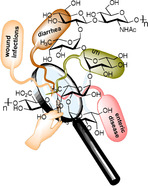

## Introduction

The outer membrane of Gram‐negative bacteria is covered with complex lipopolysaccharides.^1^ These are considered to be virulence factors. Structurally, they consist of three distinct domains: directly attached to the membrane is lipid A which is connected to a core oligosaccharide (core region). The latter is most often decorated with the so‐called O‐polysaccharide (O‐antigen) which differs strongly between different bacteria, but also within different serogroups of the same bacterial species. Thus, this coat is able to serve as a kind of fingerprint for the detection of specific bacteria, but also for the development of vaccine candidates.^2^

Carbohydrates serve as a perfect handle to transport specificity. The number of possible isomers for a given number of subunits dwarfs that of proteins by orders of magnitude.^3^ Because of several hydroxyl and/or amino groups and the possibility of two different stereoisomers when forming the glycosidic bond a plethora of structural isomers is possible. In addition, many bacteria use—besides the well‐known ten mammalian monosaccharides^4^—so‐called bacterial sugars which are either deoxygenated, equipped with further amino groups, alkylated at the carbon skeleton or enlarged to C_8_ and C_9_ sugars.^5^

In 2008 the carbohydrate chain of the lipopolysaccharide of *Providencia rustigianii* O34 was structurally elucidated.^6^ This bacterial genus causes numerous infections in humans and is classified into eight species (e.g. *P. alcalifaciens* and *P. rustigianii*).^7^ They are found in humans, and animal reservoirs, but also in soil, water and sewage. In humans, *Providencia* species have been isolated most commonly from urine, stool and blood, but as well from sputum, skin, and wound cultures. The genus *Providencia* includes urease‐producing Gram‐negative bacteria that are responsible for a wide range of human infections. Although most *Providencia* infections involve the urinary tract, they are also associated with gastroenteritis and bacteremia.^8^ In general, an emerging issue is arising from increasing incidence of antibiotic resistance secondary to extended‐spectrum beta‐lactamase.

Because it is difficult to isolate corresponding saccharides in acceptable purity and sufficient amount from the bacterial sources due to their microheterogeneous character, the preparation of the repeating subunits by chemical means is the method of choice.^9^

**Figure 1:**
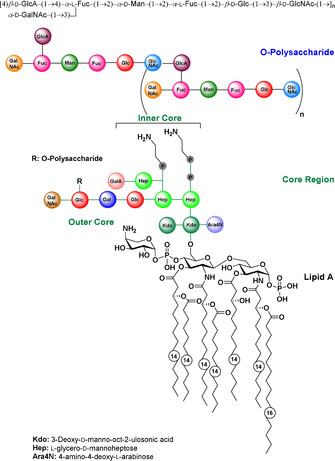
The structure of LPS derived from *Providencia rustigianii* O34.

The schematic illustration of lipid A, a core region and the repeating unit of the LPS derived from *P. rustigianii* O34, are depicted in Figure 1. Although no special bacterial monosaccharides are found in the glycan it shows some structural challenges. In contrast to mammalian oligosaccharides^4^ in which fucose is only known as a terminal unit, we encounter two α‐fucoses embedded in the chain. One of them is connected to the next monosaccharidic unit via its 2‐hydroxy group while the other is branched with further monosaccharide residues at the 3‐ and 4‐hydroxy groups. As terminal sugars at the non‐reducing end, β‐d‐glucuronic acid and α‐d‐galactosamine are found. In this paper, we describe our synthetic endeavors to access tri‐, hexa‐ and heptasaccharidic substructures of the O‐polysaccharide found on the outer surface membrane of *Providencia rustigianii* O34. Such fragments conjugated to a protein should be highly useful tools to elicit carbohydrate‐specific antibodies against these bacteria.

## Results and Discussion

**Scheme 1:**
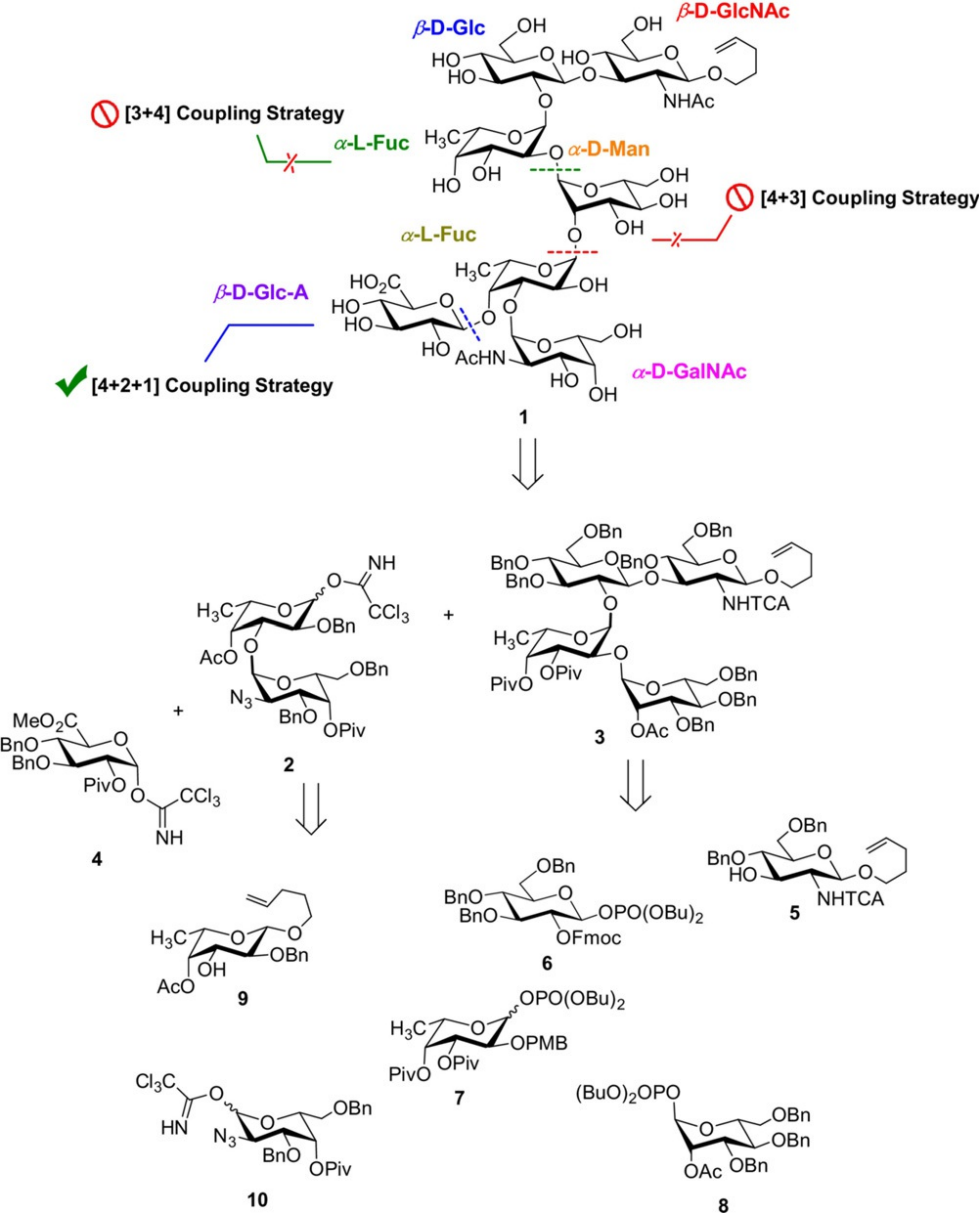
Different retrosynthetic strategies (failed [3+4] and [4+3]) and our successful [4+2+1] strategy to build up the heptasaccharide unit.

The target structure **1** was equipped with a pentenyl chain at the reducing sugar to have a handle for further modification, or as an attachment point for proteins (Scheme 1). Many retrosynthetic strategies can be envisioned for the desired compound. We started our attempts with [4+3]‐ and [3+4]‐couplings, but failed to achieve the glycosidic bonds between Fuc and Man or Man and Fuc, respectively (for more details see Supporting Information). Finally, a [4+2+1] coupling strategy was more successful. The retrosynthetic analysis of **1** is depicted in Scheme 1. In total, seven different building blocks **4**–**10** are necessary. As temporary protecting groups Fmoc, PMB and Ac are used whereas Bn and Piv groups permanently protect the hydroxyl groups. The amine functionalities are silenced by either trichloroacetamide (TCA) protection or are masked as azides.

**Scheme 2:**
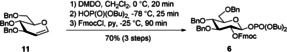
Synthesis of the d‐glucosyl phosphate donor **6**.

Our synthetic endeavor started with the preparation of the literature‐known *n*‐pentenyl d‐glucosamine acceptor **5**.^10^ The second building block, glucosyl dibutyl phosphate **6**, was prepared in a one‐pot procedure from perbenzylated d‐glucal **11** (Scheme 2). Dimethyldioxirane (DMDO)^11^ is able to attack the electron‐rich enol ether in a facially selective manner to form a highly reactive acetal epoxide which is easily opened under acidic conditions by dibutylphosphate. The emerging hydroxy group was converted by FmocCl into the respective carbonate. The carbonyl as neighboring‐participating group ensures the β‐selectivity in the glycosylation reaction.

The well‐known d‐mannosyl phosphate **8** was prepared in a few steps according to literature methods from commercially available mannose (see Supporting Information).^12^

**Scheme 3:**
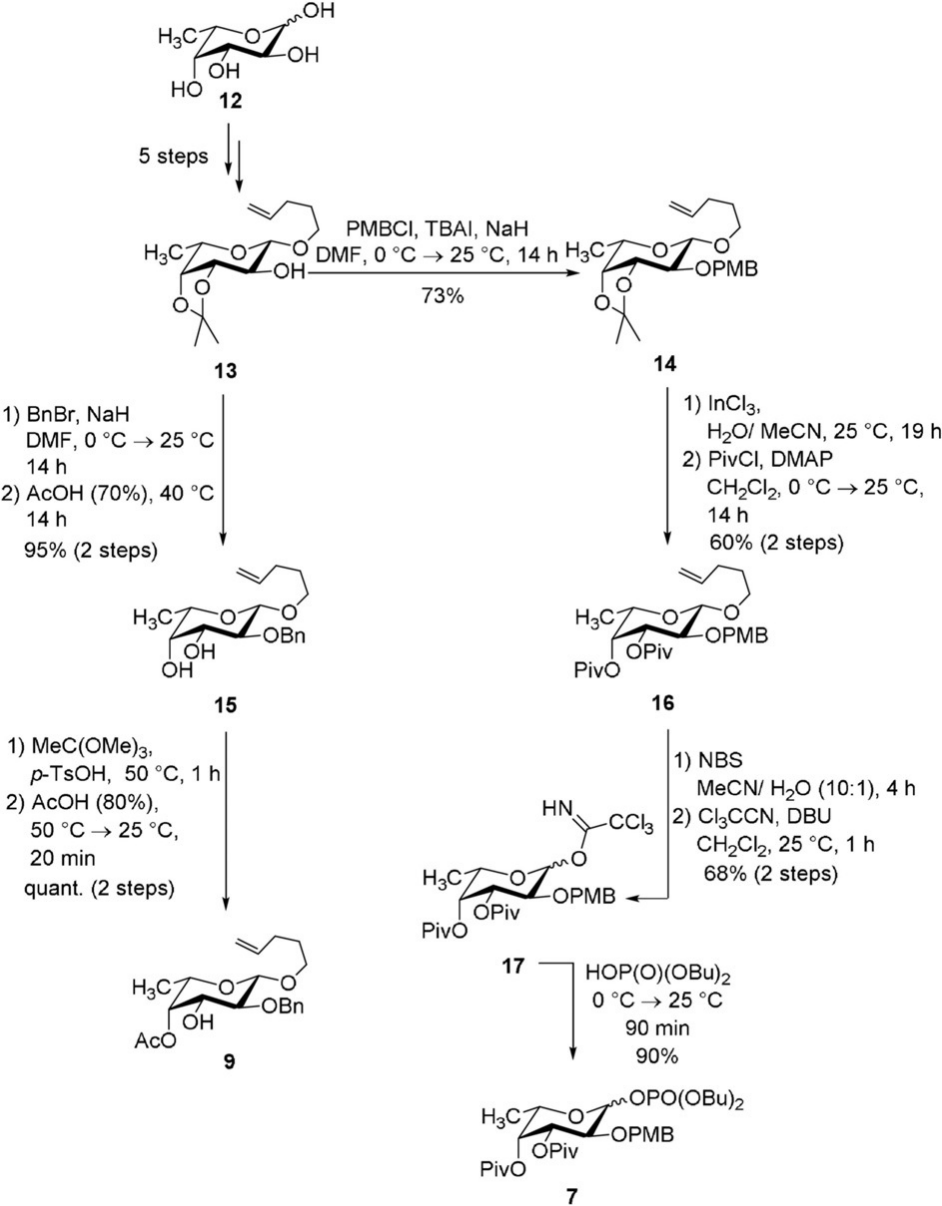
Synthesis of the l‐fucosyl phosphate donor **7** and l‐fucose acceptor **9**.

The two fucose building blocks were prepared from a common literature‐known 3,4‐isopropylidene‐protected fucose **13**.^13^ Both fucose moieties in the target structure are α‐linked; thus, it is prerequisite that no neighboring‐participating group is located in position 2. Since one of the fucose units is linked at the 2‐OH group a removable ether group as temporary protecting group is crucial. Therefore, we relied on *p*‐methoxybenzyl ether (PMB)^14^ which was installed in 73 % yield. Cleavage of the acetonide in **14** was achieved by using an indium‐based procedure.^15^ Besides the non‐participating group in position 2, an installation of electron‐withdrawing groups in position 3 and 4 is another possibility to further increase α‐selectivity in the glycosylation reaction.^16^ Thus, pivaloate esters were installed. The explanation for these groups being beneficial are various; there are reports that suggest these groups are remote participating groups^17^ whereas others only emphasize the electron‐withdrawing effect. Nevertheless, their utility to achieve high α‐selectivity has been demonstrated in many cases. The cleavage of the anomeric pentenyl protecting group was achieved by NBS in aqueous conditions. The resulting hemiacetal was converted into the Schmidt donor **17** in 68 % yield over two steps. Later, we found that the respective phosphate showed a better behavior in the glycoslation reaction; thus, the former donor was further transformed into the fucosyl phosphate **7** (Scheme 3).

The other fucose unit is branched with two different residues attached to position 3 and 4. In our [4+2+1] strategy this fucose serves as a starting point (i.e. as glycosyl acceptor) for a disaccharide synthesis; therefore, only one temporary protecting group is required. 2‐Benzyl protection ensures the α‐selectivity. Acidic conditions cleave the isopropylidene of **13**. A regioselective protection of the 4‐hydroxy group was achieved by the reaction with methyl orthoformate in the presence of *p*‐TsOH leading to an intermediate formation of the corresponding orthoester. The latter was opened by AcOH (80 %)^18^, ^19^ in such a way that only the axial acetate results; consequently, the desired fucosyl acceptor **9** was obtained in quantitative yield over two steps (Scheme 3).

**Scheme 4:**
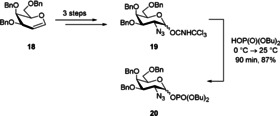
Synthesis of the d‐galactosyl trichloroacetimide **19** and phosphate **20**.

The terminal galactosamine building block was obtained via an azidonitration procedure^20^ starting from perbenzylated galactal **18**. In three steps the respective trichloroacetimidate was obtained. In some instances, glycosyl phosphates gave better results in our hands than the corresponding trichloroacetimidates; thus, we converted **19** into its phosphate analog **20** in 87 % yield (Scheme 4).

**Scheme 5:**
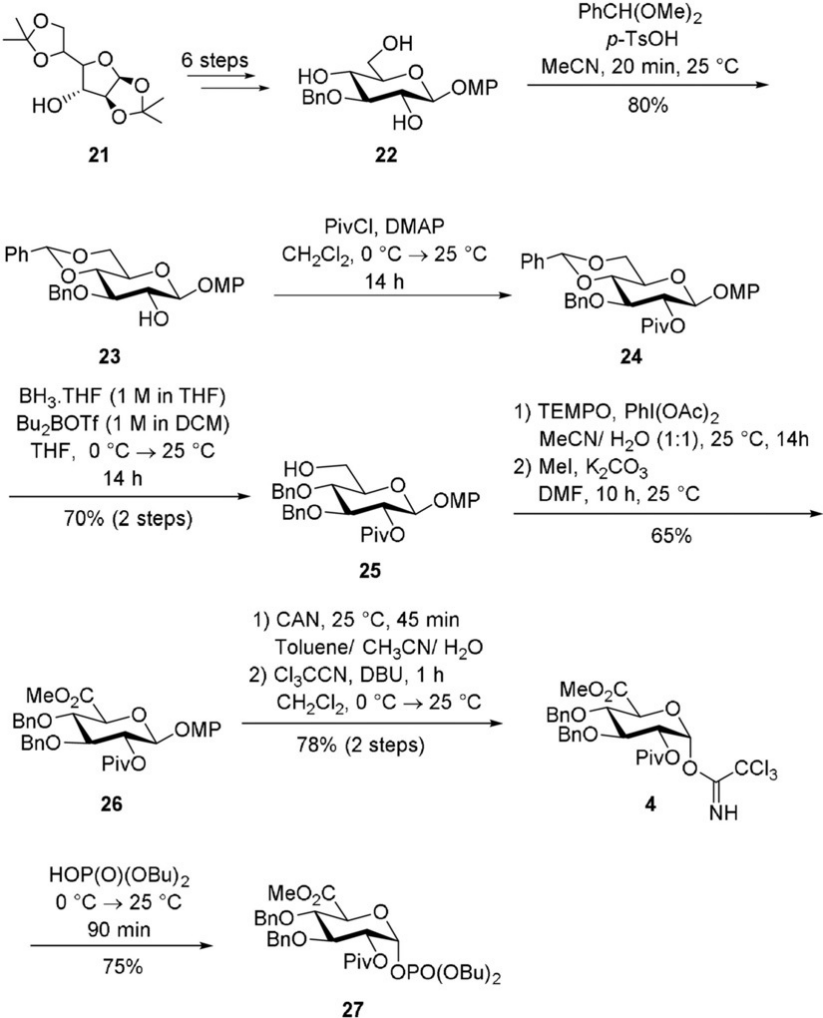
Synthesis of the d‐glucosyl trichloroacetimide **4** and phosphate donor **27**.

The final building block **27** is accessible from literature‐known triol **22**.^21^ Treatment of the triol with benzaldehyde dimethyl acetal under the catalytic influence of *p*‐TsOH provided benzylidene acetal **23** (Scheme 5). Protection of the 2‐hydroxy group by pivaloate, followed by subsequent regioselective ring‐opening of the benzylidene acetal using a borane‐THF complex in the presence of Bu_2_BOTf furnished alcohol **25**. Transformation to the corresponding glucuronic acid was achieved under oxidizing conditions, the emerging acid functionality was immediately protected by methylation with methyl iodide under basic conditions. Finally, the anomeric protecting group was removed by the action of cerium ammonium nitrate (CAN); the hemiacetal was transformed into trichloroacetimidate **4**. Only one anomer was formed; the small ^3^ *J*_1,2_ coupling constant of 3.6 Hz suggests the generation of the α anomer. The respective phosphate **27** was obtained in 75 % yield by the reaction of **4** with dibutyl phosphate.

**Scheme 6:**
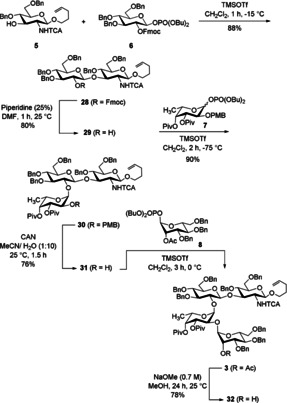
Synthesis of the tetrasaccharide acceptor **32**.

With these building blocks in hand we started to assemble the heptasaccharide. Galactosyl acceptor **5** and glucosyl phosphate **6** were coupled under Lewis‐acidic conditions applying TMSOTf as promotor leading to the (1→3)‐linked disaccharide **28** in 80 % yield (Scheme 6). High β‐selectivity was observed due to anchimeric assistance of the Fmoc group. Deprotection of the latter using piperidine generated disaccharide **29**. The glycosylation of **29** with fucosyl phosphate **7** in the presence of TMSOTf led to trisaccharide **30** with complete α‐selectivity. Because of the acidic conditions the PMB group was cleaved to some extent,^22^ nevertheless the PMB‐protected trisaccharide was obtained as major product. This compound was treated with CAN in aqueous medium to afford trisaccharide acceptor **31**. Glycosylation with mannosyl phosphate **8** yielded tetrasaccharide **3** in 80 % yield. A subsequent saponification removed the acetate in position 2 of the mannose unit affording **32** (Scheme 6).

**Scheme 7:**
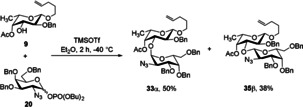
Synthesis of the disaccharide **33**.

**Scheme 8:**
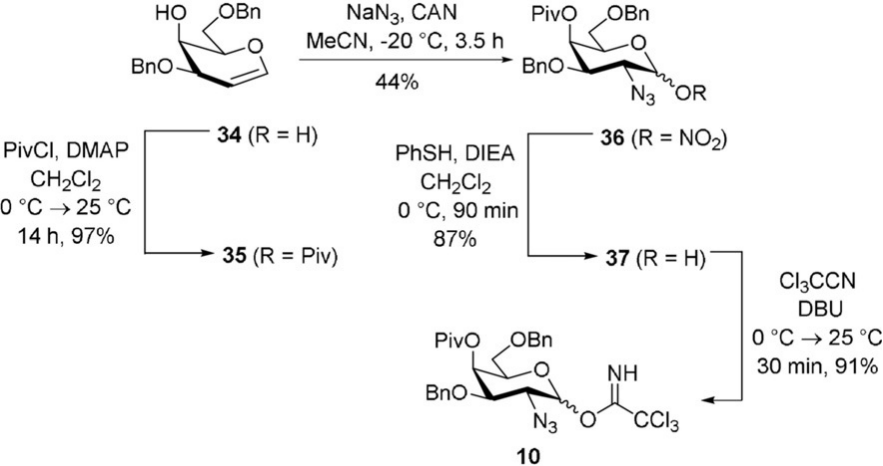
Synthesis of the d‐galactosyl donor **10**.

Initially, we tried a [4+3] approach to build up the heptasaccharide. Thus, we prepared a branched trisaccharide building block which contains fucose, galactosamine and glucuronic acid. We started to investigate the formation of this α‐linked disaccharide by using a more easily available perbenzylated galactosamine donor **20** with an azide moiety in position 2. Although ether was used as solvent which should favor the desired α‐selectivity, it was poor (α/*β*=1.3/1) (Scheme 7). Thus, a modified less reactive building block was prepared starting from the respective 3,6‐dibenzylated galactal. Protection of the 4‐hydroxy group by pivaloate and azidonitration, followed by reductive nitrate cleavage led to hemiacetal **37** which was further transformed into the trichloroacetimidate **10** (Scheme 8).

**Scheme 9:**
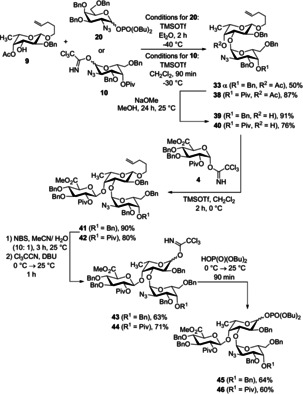
Synthesis of the trisaccharide phosphates **45** and **46**.

With the more electron‐poor galactosamine donor in hand we performed again the glycosylation to the disaccharide. Now, complete α‐selectivity was obtained. The following reactions were carried out with both the benzylated disaccharide (as described in Scheme 7) and the disaccharide with a Piv group in the 4‐position. The disaccharides **33α** and **38** were hydrolyzed under basic conditions (in the case of pivaloate strong basic conditions should be avoided); the emerging hydroxy groups were subsequently reacted with methyl glucoronate **4** under Lewis acidic conditions to furnish trisaccharides **41** and **42**, respectively. Cleavage of the pentenyl group generated the hemiacetals which were transformed via the trichloroacetimidates into the corresponding phosphates **45** and **46** (Scheme 9).

For the [4+3] coupling a plethora of different conditions were tested (see Supporting Information). All attempts remained unsuccessful and tetrasaccharide acceptor was recovered either unaffected or with partial decomposition. The trisaccharide donor often decomposed to its hydrolyzed form or was isolated as the trehalose‐like 1,1‐linked dimerization product. Steric hindrance of the branched trisaccharide seems to be too high for a successful glycosylation. As an alternative we also performed a [3+4] coupling starting with a trisaccharide acceptor and a tetrasaccharide donor; However, these attempts were also unsuccessful (see Supporting Information).

**Scheme 10:**
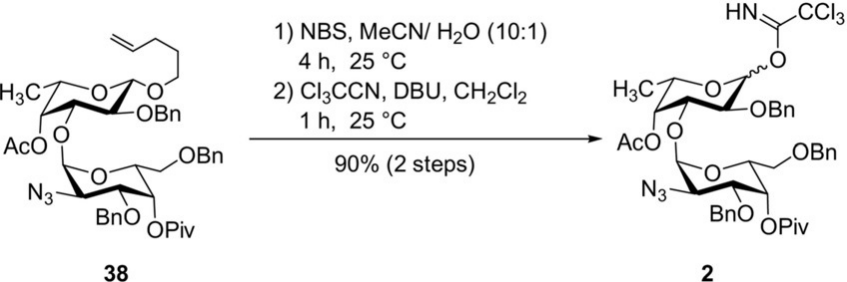
Synthesis of disaccharide trichloroacetimidate **2**.

**Scheme 11:**
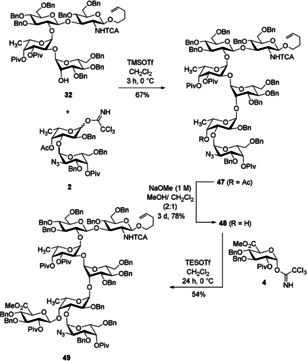
Synthesis of heptasaccharide **49** using a [4+2+1] strategy.

After these failures, we redesigned our strategy towards a [4+2+1] coupling. The monosaccharidic building blocks are the same as previously described (see Scheme 1). Instead of a trisaccharide donor, we prepared disaccharide trichloroacetimidate **2** from pentenyl disaccharide **38** described above (Scheme 10). This trichloroacetimidate was united with the tetrasaccharide (as described in Scheme 6). In contrast to the previous attempts to establish that linkage this [4+2] coupling afforded the desired hexasaccharide **47** in 67 % yield and based on recovered starting materials even with up to 90 % yield (Scheme 11). The subsequent transesterification under basic conditions removed the acetate in position 4 of the second fucose moiety to afford **48**.

To build up the heptasaccharide, extensive screening with respect to the promotor was necessary. The glycosidic bonds to the fucose residues tend to be cleaved under strong Lewis acidic conditions. Thus, it was very important to choose carefully the Lewis acid for the final glycosidic bond formation. With BF_3_⋅OEt_2_ only slow conversion was observed and a large amount of unreacted hexasaccharide acceptor was recovered from the reaction mixture. When TMSOTf was used to catalyze the glycosylation reaction increased reactivity was observed, but it was accompanied by decomposition of the starting materials. In contrast to TMSOTf, a milder Lewis acidic promotor such as TESOTf produced the desired product in a moderate yield. Further optimization of other parameters showed that this very sensitive glycosylation step gives the best yield after stirring for 24 h at 0 °C (Scheme 11). It is noteworthy that slow addition of the diluted activator was crucial (see Supporting Information).

Finally, we used size‐exclusion gel‐permeation HPLC to obtain the pure product **49** (see Supporting Information). As side products, smaller parts of the heptasaccharide were found in all cases. The glycosidic bonds to fucose units are not as stable as to Glc, Gal or Man. Under the influence of strong Lewis or Brønsted acids they tend to be cleaved because the emerging positive charge is much better stabilized in these 6‐deoxy sugars than in the 6‐oxygenated counterparts.

**Scheme 12:**
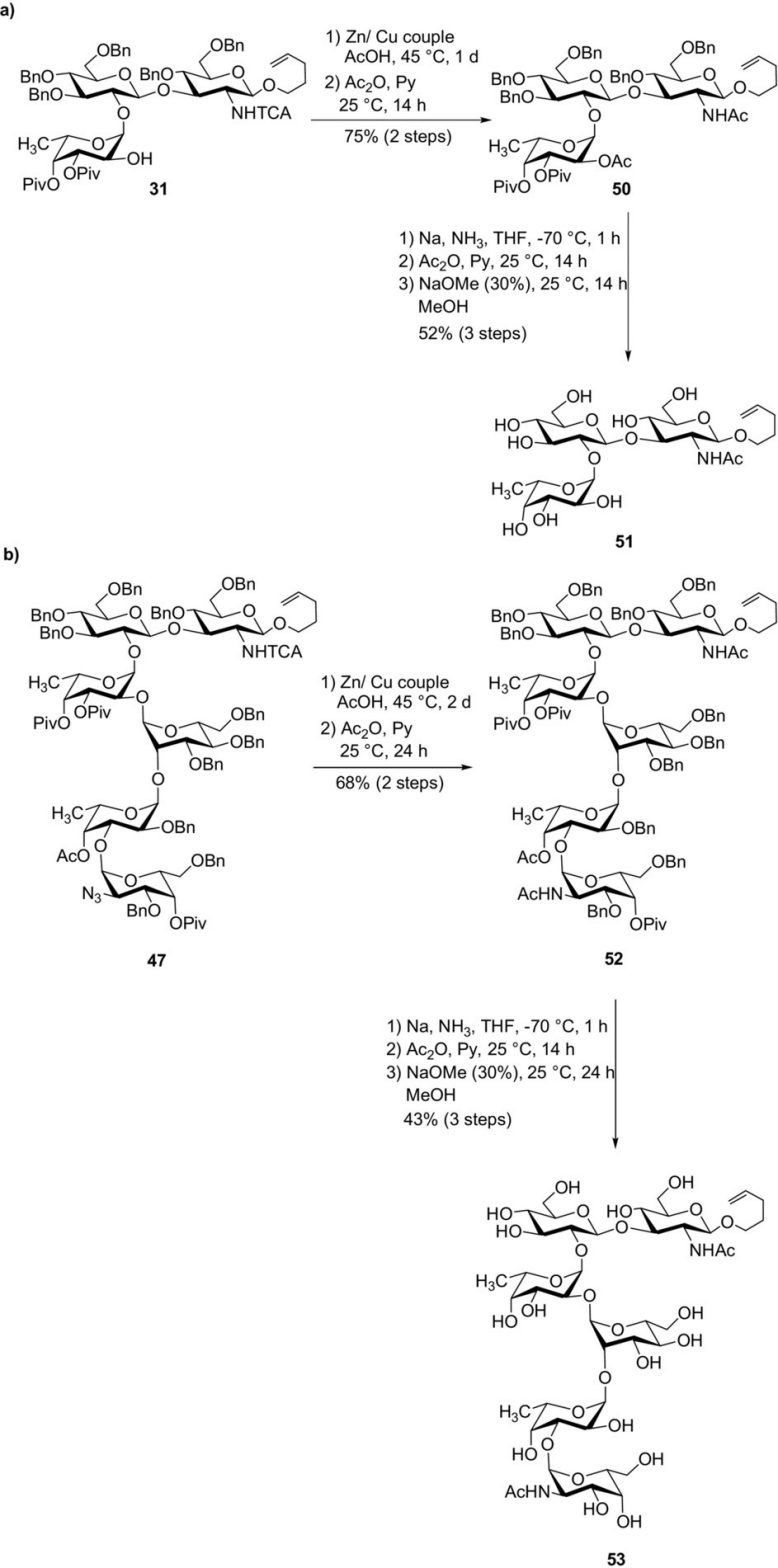
Global deprotection to tri‐ and hexasaccharides **51** and **53**.

With the tri‐, hexa‐ and heptasaccharides **31**, **47**, and **49** in hand we performed studies with respect to their global deprotection. Hydrogenation of the larger structures was associated with incomplete removal of the Bn groups; therefore, we employed the Birch reaction to achieve our goal. It is well‐known that the TCA group often causes problems in Birch reactions because the relatively acidic N−H bond gets deprotonated and the emerging anion acts as a good nucleophile.^23^ As a result, the adjacent glycosidic bond might break. Thus, we first used zinc‐copper couple as a reducing agent to convert the TCA group into its acetamide analog, followed by acetylation of other emerging amino groups since azide moieties are also reduced using this procedure. Global deprotection was obtained under Birch conditions. For simpler purification the naked sugars were acetylated and then treated with sodium methoxide in methanol. The desired target compounds **51** and **53**, respectively, were obtained in pure form after dialysis and lyophilization (Scheme 12).

**Scheme 13:**
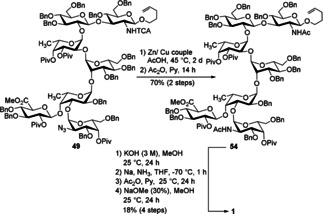
Synthesis of heptasaccharide **1**.

Encouraged by these results, we applied the optimized conditions to access the deprotected heptasaccharide. Firstly, acetamides were generated from the TCA‐protected amine and the azide, respectively. To avoid epimerization at C‐5 of the glucuronic acid under Birch conditions, we transformed the ester to the corresponding carboxylic acid. Global deprotection by Birch reaction, subsequent acetylation and deacetylation yielded the final product **1** which was confirmed by high‐resolution mass spectrometry and ^1^H NMR spectroscopy including HSQC (Scheme 13). Because of the very small amount we were unable to record a proper ^13^C NMR spectrum. Although we performed a chromatographic purification for three times by using a Sephadex LH20 column, the compound did not reach the high purity we obtained for the deprotected tri‐ and hexasaccharides **51** and **53**.

## Conclusions

Herein, we presented the first syntheses of the tri‐, hexa‐ and heptasaccharidic substructures of the O‐polysaccharide in *Providencia rustigianii* O34. The polymeric structure consists of a repeating unit with seven different monosaccharide moieties; thus, seven different monosaccharidic building blocks were used. Because relatively labile α‐linked fucosyl residues are embedded in the structure special care is needed for the choice of the glycosylation conditions. Lewis acids which are too harsh led to a decomposition of the oligomer by cleaving α‐fucosidic bonds. The reducing end of all structures was equipped with a pentenyl handle, which can be used for attachment to surfaces and proteins. In future, these structures will be employed to elicit carbohydrate‐specific antibodies that might be useful for the detection of *Providencia rustigianii* O34 or even for vaccination studies.

## Conflict of interest

The authors declare no conflict of interest.

## Supporting information

As a service to our authors and readers, this journal provides supporting information supplied by the authors. Such materials are peer reviewed and may be re‐organized for online delivery, but are not copy‐edited or typeset. Technical support issues arising from supporting information (other than missing files) should be addressed to the authors.

Supplementary
