# Supplementary material for: Total Synthesis of Tri‐, Hexa‐ and Heptasaccharidic Substructures of the O‐Polysaccharide of Providencia rustigianii O34
Source: Chemistry. 2020 Apr 28;26(28):6264–70. doi: 10.1002/chem.202000496 (PMC7318715; doi:10.1002/chem.202000496)
Supplement: Supplementary file 1 — Supplementary [file CHEM-26-6264-s001.pdf]

Chemistry–A European Journal

Supporting Information

Total Synthesis of Tri-, Hexa- and Heptasaccharidic Substructures of the O-Polysaccharide of *Providencia rustigianii* O34

Somayeh Ahadi+,[a] Shahid I. Awan+,[b] and Daniel B. Werz*[a]

Total Synthesis of Tri-, Hexa- and Heptasaccharidic Substructures of the O-Polysaccharide of *Providencia rustigianii O34*

Somayeh Ahadi,[a] Shahid I. Awan,[b] and Daniel B. Werz*[a]

[a]Institut für Organische Chemie, Technische Universität Braunschweig Hagenring 30, 38106 Braunschweig, Germany
[b]Institute of Organic and Biomolecular Chemistry, Georg-August-Universität Göttingen Tammannstraße 2, 37077, Göttingen, Germany

Supporting Information

Table of Contents

1. General Information S2
2. Synthesized building blocks S3
3. Spectroscopic data and procedure of prepared building blocks S6
   3.1 Data for glycoside acceptor **5** S6
   3.2 Data for glycosyl donor **6** S6
   3.3 Data for fucosyl donor **7** S9
   3.3 Data for fucosyl acceptor **9** S9
   3.5 Data for galactosyl donor **10** S9
   3.6 Data for mannosyl donor **8** S11
   3.7 Data for glucosyl donor **4** S11
4. NMR Spectra for Building Blocks S15
5. Glycosylation reactions S30
6. Spectroscopic data of synthesized final compounds in glycosylation reactions S33
   6.1 Data for disaccharide acceptor **29** S33
   6.2 Data for trisaccharide acceptor **31** S34
   6.3 Data for tetrasaccharide acceptor **32** S35
   6.4 Data for disaccharide donor **2** S37
   6.5 Data for hexasaccharide acceptor **48** S38
   6.6 Data for hexasaccharide **52** S40
   6.7 Data for heptasaccharide **49** S41
   6.8 Data for heptasaccharide **97** S42
7. NMR Spectra for glycosylation reactions S44
8. Reactions of deprotection by Birch reaction S57
9. Spectroscopic data of synthesized final compounds in Birch reactions S59
   9.1 Data for deprotected trisaccharide **51** S59
   9.2 Data for deprotected hexasaccharide **53** S61
   9.2 Data for deprotected heptasaccharide **1** S63
10. NMR Spectra for final deprotected products S65
11. Spectroscopic data of synthesized compounds in failure stratagies S73
12. NMR Spectra for compounds in failure stratagies S81
13. Table of attempted assembly of heptasaccharide **49** by [4+3] coupling S89
14. Table of attempted assembly of heptasaccharide **49** by [3+4] coupling........ S90
15. Table of attempted assembly of tetrasaccharide **105** by [3+1] coupling........ S91
16. Table of optimization of reaction conditions for the [6+1] coupling........ S92
17. Table of optimization hydrolysis of methyl ester **54** S92
18. The chart of recycle preparative HPLC for heptasaccharide........ S92
19. References S93

Experimental Section

1. General Information

All solvents were purchased as HPLC grade solvents and stored over molecular sieves. All reactions were carried out in oven-dried glassware, septum-capped under atmospheric pressure of argon. Commercially available compounds were used without further purification unless otherwise stated. Proton (^{1}H) and carbon (^{13}C) NMR spectra were recorded on 300, 400, 500 or 600 MHz spectrometers using the residual signals from tetramethylsilane (TMS) δ = 0.00 ppm, as internal references for ^{1}H and ^{13}C chemical shifts, respectively. The following abbreviations were used to explain the multiplicities: s = singlet, d = doublet, t = triplet, q = quintet, m = multiplet. ESI-HRMS mass spectrometry was carried out on a FTICR instrument and IR spectra were measured on an ATR spectrometer. Optical rotations were measured on a common polarimeter.

2. Synthesized building blocks

2.1. Synthesis of the glycoside acceptor 5.

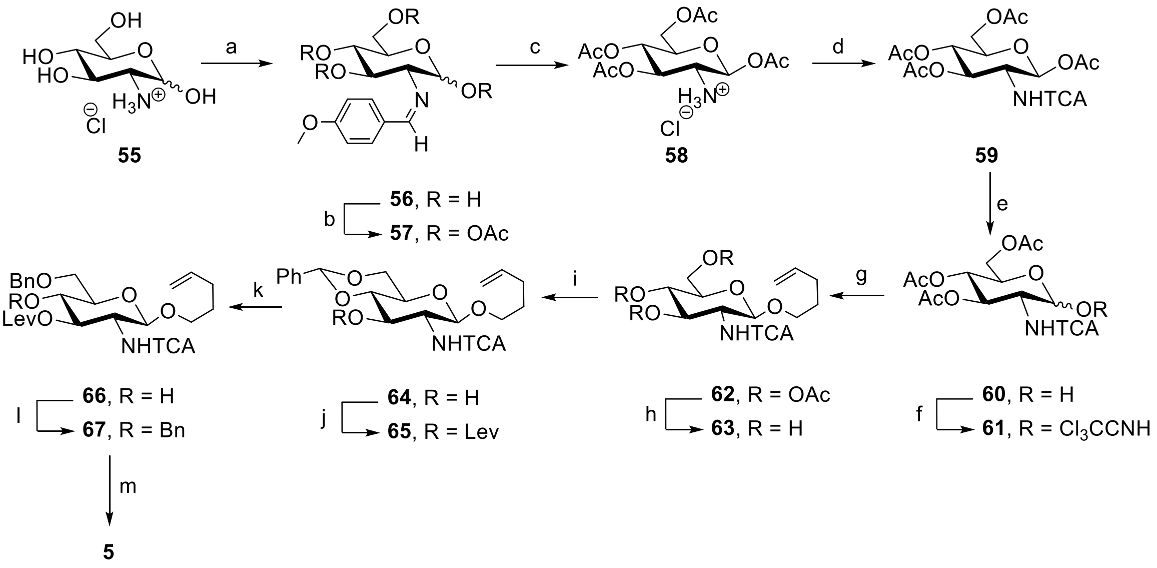

a) *p*(MeO)C_6H_4CHO, NaOH (1 M), 25 ºC **b)** Ac_2O, py., cat. DMAP, 25 ºC **c)** Refluxing in acetone, Then 5 N HCl **d)** Cl_3CCOCl, py, CH_2Cl_2, 0 °C → 25 ºC **e)** 5% piperidine in THF **f)** Cl_3CCN, DBU, 0 °C → 25 ºC **g)** 4-penten-1-ol, TMSOTf, CH_2Cl_2, 0 °C **h)** NaOMe (30%), MeOH, 25 ºC **i)** $PhCH(OMe)_2$, *p*-TsOH, MeCN, 25 ºC **j)** LevOH, DIC, DMAP, CH_2Cl_2, 0 °C → 25 ºC **k)** Et_3SiH, TFAA, TFA, CH_2Cl_2, 0 °C → 25 ºC **l)** BnBr, Ag_2O, CH_2Cl_2, 0 °C → 25 ºC **m)** N_2H_4.AcOH, CH_2Cl_2/ MeOH (5:1).

2.2. Synthesis of the glycosyl donor 6.

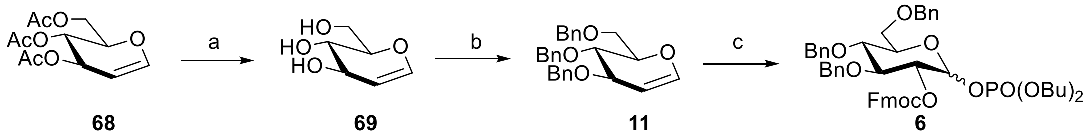

a) NaOMe (30%), MeOH, 25 ºC **b)** BnBr, NaH, DMF, 0 °C → 25 ºC **c)** DMDO, $HOP(O)(OBu)_2$, FmocCl, py, CH_2Cl_2.

2.3. Synthesis of the fucosyl donor 7.

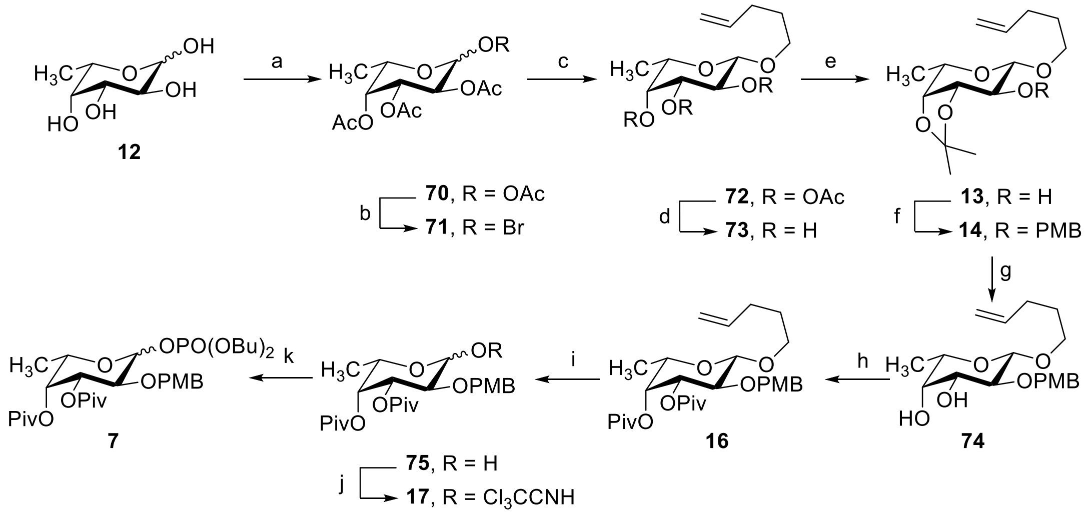

a) Ac_2O, py, 0 °C → 25 ºC **b)** HBr (35% in AcOH), HOAc, 25 ºC **c)** 4-penten-1-ol, Ag_2CO_3, MS 4 Å, CH_2Cl_2 **d)** NaOMe (30%), MeOH **e)** $Me_2C(OMe)_2$, *p*-TsOH, DMF, 25 ºC **f)** PMBCl, TBAI, NaH, DMF, 0 °C → 25 ºC **g)** $InCl_3$, H_2O, MeCN, 25 ºC **h)** PivCl, DMAP, CH_2Cl_2, 0 °C → 25 ºC **i)** NBS, MeCN/ H_2O (10:1) **j)** Cl_3CCN, DBU, CH_2Cl_2, 25 ºC **k)** $HOPO(OBu)_2$, 0 °C → 25 ºC

2.4. Synthesis of the fucosyl acceptor 9.

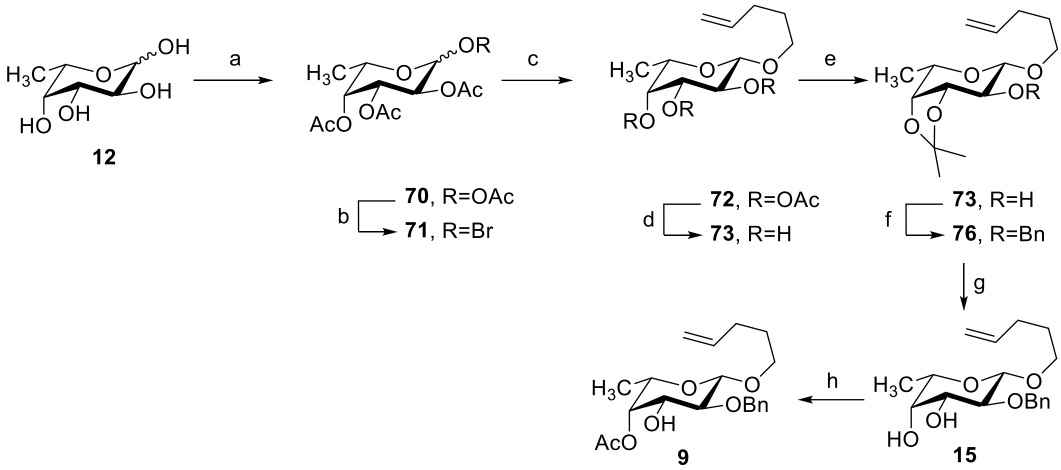

a) Ac_2O, py, 0 °C → 25 ºC **b)** HBr (35% in AcOH), HOAc, 25 ºC **c)** 4-penten-1-ol, Ag_2CO_3, MS 4 Å, CH_2Cl_2 d) NaOMe (30%), MeOH e) $Me_2C(OMe)_2$, *p*-TsOH, DMF, 25 ºC **f)** BnBr, NaH, DMF, 0 °C → 25 ºC g) AcOH (70 %) **h)** $MeC(OMe)_3$, *p*-TsOH, AcOH (80 %), 50 °C → 25 ºC.

2.5. Synthesis of the galactosyl donor 10.

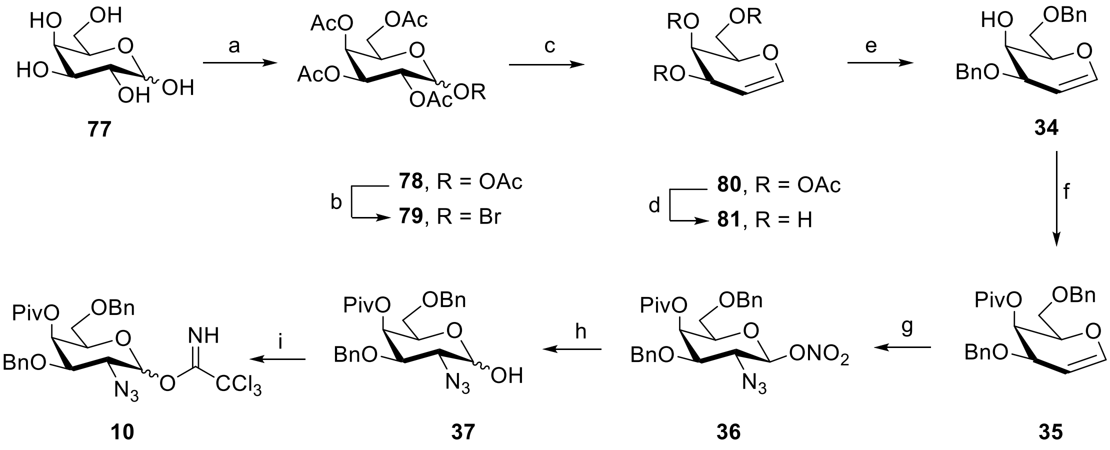

a) Ac_2O,$HClO_4$ (70%), 0 °C **b)** HBr 35% in AcOH, 25 ºC **c)** Zn, AcOH (50% in H_2O), -20 °C → 0 **d)** NaOMe (30%), MeOH **e)** $(nBu_3Sn)_2O$, BnBr, TBAI, Benzen, 90 - 110 °C **f)** PivCl, DMAP, CH_2Cl_2, 0 °C → 25 ºC **g)** NaN_3, CAN, MeCN, -20 °C **h)** PhSH, DIEA, CH_2Cl_2, 0 °C **i)** Cl_3CCN, DBU, 0 °C → 25 ºC.

2.6. Synthesis of the mannosyl donor 8.

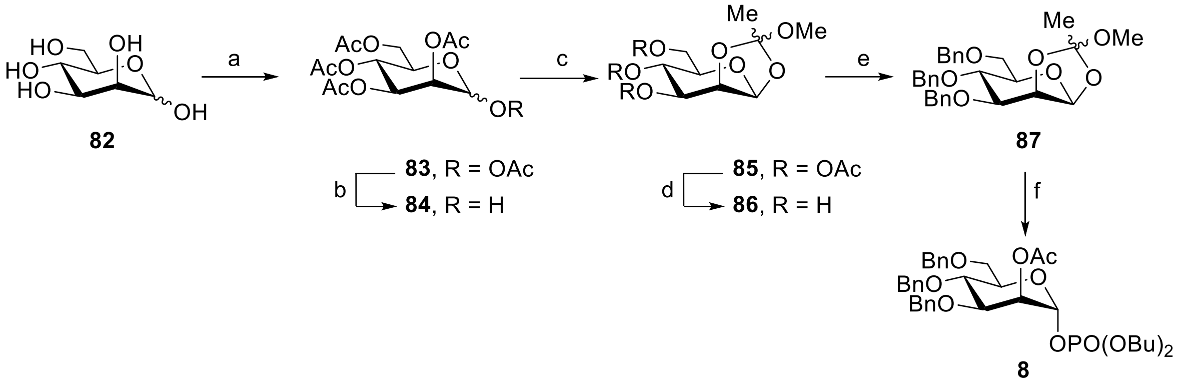

a) Ac_2O, $HClO_4$ (70%), 0 °C → 25 °C **b)** HBr 35% in AcOH, 25 °C **c)** MeOH, lutidine, CH_2Cl_2 **d)** NaOMe (30%), MeOH **e)** BnBr, NaH, DMF, 0 °C → 25 °C **f)** $HOPO(OBu)_2$ (3 M in CH_2Cl_2), MS 4 Å, NEt_3, CH_2Cl_2, 0 °C → 25 °C.

2.7. Synthesis of the glucosyl donor 4.

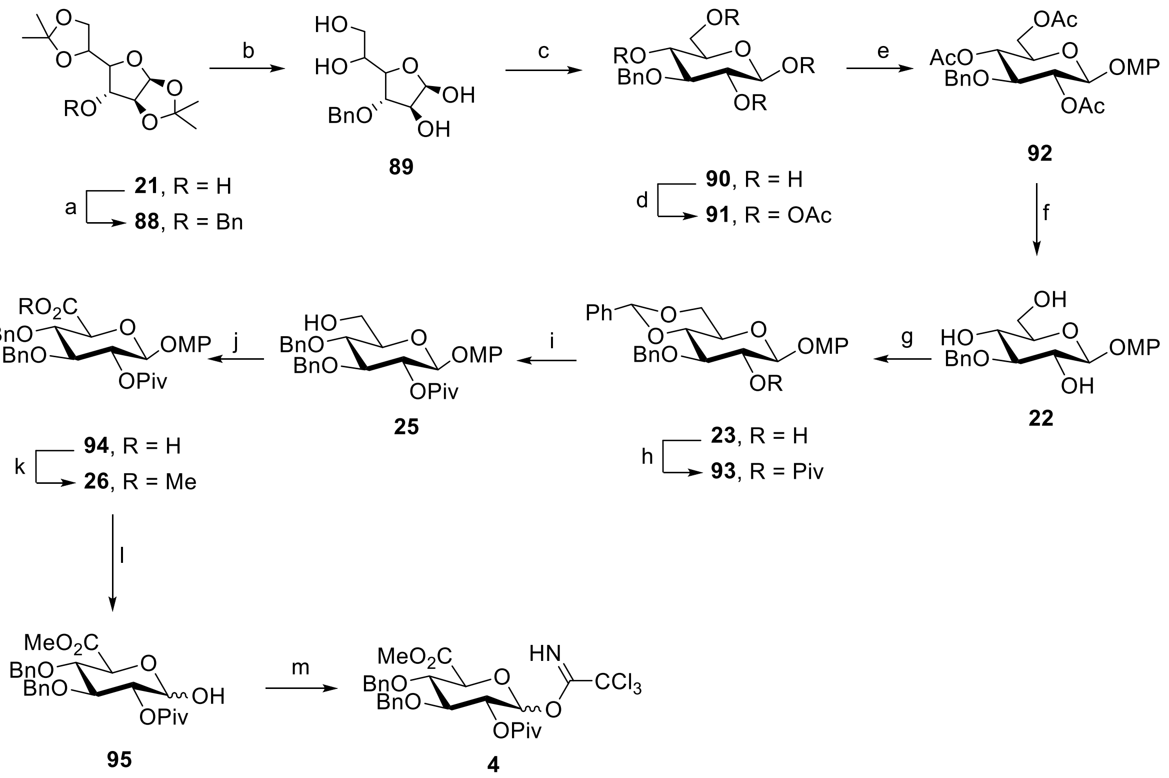

a) BnBr, NaH, DMF, 2 h, 90 °C **b)** HOAc (60%), overnight, 90 °C **c)** H_2SO_4 (0.1 M), 2 h, 90 °C **d)** NaOAc, Ac_2O, 6 h, 110 °C **e)** 4-Methoxyphenol, TMSOTf, MS 4 Å, CH_2Cl_2, 0.5 h, 0 °C **f)** NaOMe (30%), MeOH, Overnight, 25 °C **g)** $PhCH(OMe)_2$, *p*-TsOH, MeCN, 20 min, 25 °C **h)** PivCl, DMAP, CH_2Cl_2, Overnight, 0 °C → 25 °C **i)** BH_3.THF (1 M in THF), Bu_2BOTf (1 M in DCM), THF, overnight, 0 °C → 25 °C **j)** TEMPO, BAIB, MeCN / H_2O (1:1), 25 °C **k)** MeI, K_2CO_3, DMF, 10 h, 25 °C **l)** CAN, Toluene / CH_3CN / H_2O (1:1.5:1), 1 h, 25 °C **m)** Cl_3CCN, DBU, CH_2Cl_2, 0 °C → 25 °C.

3. Spectroscopic Data of Prepared Building Blocks

3.1. Synthesis of the glycoside acceptor 5.

***n*-Pentenyl 4,6-di-*O*-benzyl-2-*N*-trichloroacetyl-β-D-glucosaminopyranoside (5)**

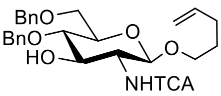

Compound **5** was prepared base on references [1-5], a slight variation from the reported procedure was applied, instead of a direct installation of *n*-pentenyl to **59** in the presence of boron trifluoride etherate ($BF_3{\cdot}OEt_2$) [2], the above route was pursued to ensure the desired *β*-linkage at the reducing end of the monosaccharide. Spectral data for **5** was consistent with that previously reported.

3.2. Synthesis of the glycosyl phosphate 6.

3,4,6-Tri-*O*-benzyl-2-*O*-fluorenylmethoxycarbonyl-β-D-glucopyranosyl dibutyl phosphate (6)

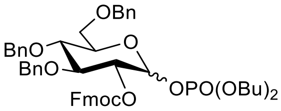

Tri-*O*-benzylglucal **11** (2 g, 4.80 mmol) was co-evaporated with toluene (3x), dried in vacuum for 1 h, dissolved in CH_2Cl_2 (45.0 mL) under argon and cooled to 0 °C. A solution of DMDO in acetone (0.080 M, 84 mL, 6.72 mmol) was added and the reaction was mixture stirred for 20 min at 0 °C. Volatiles were removed at 0 °C and the residue (colorless oil) was dried for 20 min in vacuum. Then it was dissolved in CH_2Cl_2 (45.0 mL), cooled to -78 °C and a solusion of dibutyl phosphate (1.14 mL, 5.76 mmol) in CH_2Cl_2 (3.10 mL) was added over a period of 5 min. After 20 min the solusion. was allowed to warm to -25 °C and pyridine (2.33 mL, 28.8 mmol) and FmocCl (2.47 g, 9.6 mmol) were added. After stirring for 90 min, while the temperature was raised to -10 °C, a mixture of pentane/ EtOAc (100 mL, 2:1) was added affording colorless precipitates. The mixture was flushed through a plug of silica gel, concentrated and purified by silica gel column chromatography (pentane / EtOAc, 2:1, α / β 1:4) affording 2.90 g (70%) of phosphate **18** as a colorless oil (the analytical data is for the β anomer).

$[\boldsymbol{\alpha}]_{\mathbf{D}}^{\mathbf{24}}$=+12.3 (c = 0.65, $CHCl_3$).

^{1}H NMR (600 MHz, $CDCl_3$): *δ* (ppm) 0.77 (t, *J* = 7.4 Hz, 3 H), 0.88 (t, *J* = 7.4 Hz, 3 H), 1.23 (m_c, 2 H), 1.32–1.47 (m, 4 H), 1.60 (m_c, 2 H), 3.61 (ddd, *J* = 9.6, 3.8, 2.0 Hz, 1 H), 3.73–3.84 (m, 4 H), 3.92 – 3.86 (m, 1 H), 3.99 – 3.94 (m, 1 H), 4.03– 4.08 (m, 2 H), 4.17 (t, *J* = 7.5 Hz, 1 H) , 4.28 (dd, *J* = 10.4, 7.7 Hz, 1 H), 4.38 (dd, *J* = 10.4, 7.4 Hz, 1 H), 4.50 (d, *J* = 12.0 Hz, 1 H), 4.57 – 4.60 (m, 2 H), 4.73 (d, *J* = 11.3 Hz, 1 H), 4.79 – 4.82 (m, 2 H), 4.93 (dd, *J* = 9.4, 8.0 Hz, 1 H), 5.27 (dd, *J* = 8.0, 6.9 Hz, 1 H), 7.17 – 7.24 (m, 6 H), 7.26 – 7.33 (m, 11 H), 7.37 – 7.40 (m, 2 H), 7.57 – 7.60 (m, 2 H), 7.74 – 7.76 (m, 2 H).

^{13}C NMR (150 MHz, $CDCl_3$): *δ* (ppm) 13.2, 13.3, 18.3, 18.4, 31.7, 31.7, 31.8, 31.8, 46.4, 67.6, 67.6, 67.7, 67.7, 67.9, 70.2, 73.3, 74.9, 75.2, 75.4, 76.9, 77.2, 77.3, 82.2, 96.0 (d, $^2J_{C,P}$ = 5.1 Hz, 1 C, C-1), 119.8, 124.9, 125.0, 127.0, 127.0, 127.5, 127.6, 127.7, 127.7, 127.8, 128.1, 128.2, 128.2, 137.5, 137.6, 137.6, 141.0, 141.2, 142.9, 143.0, 154.0.

IR (ATR): $\tilde{\nu}$ (cm^{-1}) 3290, 3063, 3030, 2958, 2932, 2871, 1809, 1751, 1721, 6101, 1585.

HR-MS (ESI): m/z calcd. for $C_{50}H_{57}O_{11}PNa^+$ 887.3531, found: 887.3515.

3.3. Synthesis of the fucosyl phosphate 7.

***n*-Pentenyl 2-*O*-*p*-methoxybenzyl-β-L-fucopyranoside (74)**

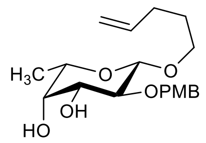

Compound **14** (3.25 g, 8.29 mmol) was dissolved in CH_3CN (90.0 mL) and H_2O (590 µL, 33.16 mmol). $InCl_3$ (3.64 g, 16.6 mmol) was added and the reaction mixture was stirred for 19 h at room temperature. Afterwards the reaction was quenched by addition of water (150.0 ml) and extracted with dichloromethane (3x). The combined organic layer was dried over $MgSO_4$ and evaporated under reduced pressure to dryness. The resulting crude product was purified by silica gel column chromatography (pentane / EtOAc, 4:1 → 1:4) to afford 1.75 g (60%) of **74** as a pale yellow oil.

$[\boldsymbol{\alpha}]_{\mathbf{D}}^{\mathbf{20}}$ = - 21.7° (c = 0.83, $CHCl_3$).

^{1}H NMR (300 MHz, $CDCl_3$): δ (ppm) 1.33 (d, *J* = 6.5 Hz, 3 H), 1.71–1.81 (m, 2 H), 2.14–2.22 (m, 2 H), 2.48 (d, *J* = 3.7 Hz, 1 H), 2.26 (d, *J* = 4.4 Hz, 1 H), 3.41 (dd, *J* = 9.5, 7.7 Hz, 1 H), 3.49–3.56 (m, 3 H), 3.70 (d, *J* = 3.3 Hz, 1 H), 3.80 (s, 3 H), 3.95–4.00 (m, 1 H), 4.32 (d, *J* = 7.6 Hz, 1 H), 4.60 (d, *J* = 11.2 Hz, 1 H), 4.89 (d, *J* = 11.2 Hz, 1 H), 4.95–5.07 (m, 2 H), 5.84 (m_c, 1 H), 6.88 (m_c, 2 H), 7.29 (m_c, 2 H).

^{13}C NMR (75 MHz, $CDCl_3$): δ (ppm) 16.2, 28.9, 30.3, 55.2, 69.1, 70.2, 71.2, 73.4, 74.1, 78.4, 103.5, 114.0, 114.9, 129.8, 130.5, 138.1, 159.4.

IR (Film): $\tilde{\nu}$ (cm^{-1}) 3424, 3075, 3036, 2977, 2936, 2874, 1640, 1613, 1586, 1514, 1463.

HR-MS (ESI): m/z calcd. for $C_{19}H_{28}O_6Na^+$ 375.1778, found: 375.1784.

***n*-Pentenyl 2-*O*-*p*-methoxybenzyl-3,4-di-*O*-pivaloyl-β-L-fucopyranoside (16)**

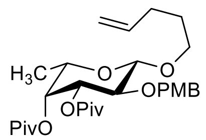

Compound **74** (2.27 g, 6.5 mmol) and DMAP (6.34 g, 52 mmol) in CH_2Cl_2 (24.00 mL) was cooled to 0 °C and PivCl (3.19 mL, 26 mmol) was added. The reaction mixture was stirred overnight at room temperature. It was diluted with a mixture of pentane and EtOAc (1:1) and filtered through a plug of silica gel. Volatiles were removed and the resulting crude product was purified by silica gel column chromatography (pentane / EtOAc, 7:1) to furnish 3.04 g (90%) of **16** as a pale yellow oil.

$[\boldsymbol{\alpha}]_{\mathbf{D}}^{\mathbf{20}}$ = - 15.7° (c = 1.1, $CHCl_3$).

^{1}H NMR (300 MHz, $CDCl_3$): δ (ppm) 1.13 (s, 9 H), 1.16 (d, *J* = 6.4 Hz, 3 H), 1.23 (s, 9 H), 1.77 (m_c, 2 H), 2.16 (m_c, 2 H), 3.51–3.59 (m, 2 H), 3.75-3.76 (m, 1 H), 3.78 (s, 3 H), 3.89-4.00 (m, 1 H), 4.41 (d, *J* = 7.7 Hz, 1 H), 4.57 (d, *J* = 10.8 Hz, 1 H), 4.79 (d, *J* = 10.8 Hz, 1 H), 4.94–5.05 (m, 3 H), 5.20 (dd, *J* = 3.4, 1.1 Hz, 1 H), 5.81 (m_c, 1 H), 6.83 (m_c, 2 H), 7.21 (m_c, 2 H).

^{13}C NMR (75 MHz, $CDCl_3$): δ (ppm) 16.1, 27.1, 27.2, 28.9, 30.2, 38.7, 39.0, 55.2, 69.1, 69.6, 70.4, 72.9, 74.2, 75.9, 103.8, 113.7, 114.9, 129.4, 130.3, 137.9, 159.2, 177.4, 177.5.

IR (Film): $\tilde{\nu}$ (cm^{-1}) 3368, 3076, 2974, 2937, 2911, 2872, 1736, 1641, 1613, 1514, 1480.

HR-MS (ESI): m/z calcd for $C_{29}H_{44}O_8Na^+$ 543.2928, found: 543.2921.

2-*O*-*p*-Methoxybenzyl-3,4-di-*O*-pivaloyl-L-fucopyranose (75)

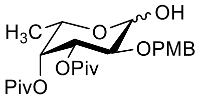

Compound **16** (1.48 g, 2.85 mmol) was dissolved in CH_3CN (37.0 mL) and H_2O (370 µL). NBS (750 mg, 4.27 mmol) was added at room temperature and the reaction mixture was stirred for 4 h. Afterwards the reaction mixture was diluted with a solution of $Na_2S_2O_3$ (10%) and extracted with EtOAc (3 x). The combined organic layer was washed with brine, dried over $MgSO_4$ and concentrated in vacuum. The resulting crude product was purified by silica gel column chromatography (pentane / EtOAc, 3:1 → 1:1) yielded 1.09 g (85%) of **75** as a pale yellow oil (the analytical data is for a mixture of β/α anomers).

^{1}H NMR (300 MHz, $CDCl_3$): δ (ppm) 1.08 (d, *J* = 6.6 Hz, 1 H), 1.15 (d, *J* = 5.0 Hz, 1 H), 1.16 (s, 9 H), 1.18 (s, 9 H), 1.24 (s, 9 H), 1.27 (s, 9 H), 1.63 (s, 1 H), 2.04 (s, 1 H), 3.03 (d, *J* = 1.9 Hz, 1 H), 3.31 (d, *J* = 5.4 Hz, 1 H),), 3.56 (dd, *J* = 10.2, 7.6 Hz, 1 H), 3.79 (s, 3 H), 3.80 (s, 3 H), 3.82–3.84 (m, 2 H), 4.34–4.40 (m, 1 H), 4.53 (d, *J* = 11.6 Hz, 1 H), 4.63 (d, *J* = 3.9 Hz, 1 H), 4.66 (d, *J* = 4.6 Hz, 1 H), 4.73–4.79 (m, 2 H), 5.00 (dd, *J* = 10.2, 3.3 Hz, 1 H), 5.20–5.23 (m, 2 H), 5.27 (dd, *J* = 3.2, 1.2 Hz, 1 H), 5.32 (dd, *J* = 10.4, 3.2 Hz, 1 H), 6.83–6.89 (m, 4 H), 7.22–7.25 (m, 4 H).

^{13}C NMR (75 MHz, $CDCl_3$): δ (ppm) 15.9, 16.1, 27.1, 27.2, 38.7, 38.7, 39.1, 55.3, 65.2, 69.4, 70.1, 70.3, 71.0, 72.9, 73.0, 73.2, 74.3, 77.1, 91.8, 97.4, 113.8, 113.9, 129.5, 129.6, 130.1, 159.3, 159.6, 177.3, 177.4, 177.4, 177.5.

IR (Film): $\tilde{\nu}$ (cm^{-1}) 3447, 3062, 2974, 2936, 2909, 2873, 2839, 2062, 1735, 1613, 1586.

HR-MS (ESI): m/z calcd. for $C_{24}H_{36}O_8Na^+$ 475.2302, found: 475.2294.

2-*O*-*p*-Methoxybenzyl-3,4-di-*O*-pivaloyl-L-fucopyranosyl trichloroacetimidate (17)

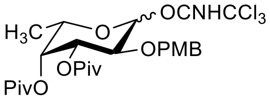

Compound **75** (830 mg, 1.84 mmol) was dissolved in CH_2Cl_2 (10.0 mL), Cl_3CCN (3.7 mL, 36.8 mmol) followed by a catalytic amount of DBU (68.65 µL, 460 µmol) were added. Purification by column chromatography (pentane / EtOAc, 5:1 → 1:1) afforded 860 mg (79%, α/β 4:1) of **17** as a pale yellow oil (The analytical data is for the α anomer).

$[\alpha]_D^{20}$= - 27.7° (c = 0.43, $CHCl_3$).

^{1}H NMR (300 MHz, $CDCl_3$): δ (ppm) 1.11 (d, *J* = 6.5 Hz, 3 H), 1.17 (s, 9 H), 1.23 (s, 9 H), 3.79 (s, 3 H), 3.99 (dd, *J* = 10.5, 3.2 Hz, 1 H), 4.34 (q, *J* = 6.7 Hz, 1 H), 4.58 (s_{bs}, 2 H), 5.34–5.43 (m, 2 H), 6.44 (d, *J* = 3.6 Hz, 1 H), 6.84 (m_c, 2 H), 7.21 (m_c, 2 H), 8.59 (s, 1 H).

^{13}C NMR (75 MHz, $CDCl_3$): δ (ppm) 16.0, 27.1, 27.2, 38.7, 39.1, 55.3, 67.7, 69.8, 70.5, 71.8, 72.3, 91.2, 94.6, 113.8, 129.3, 129.6, 159.4, 161.4, 177.2, 177.4.

IR (Film): $\tilde{\nu}$ (cm^{-1}) 3062, 2975, 2936, 2873, 2839, 2061, 1734, 1613, 1514, 1480, 1462.

HR-MS (ESI): m/z calcd. for $C_{26}H_{36}Cl_3NO_8Na^+$ 618.1399, found: 618.1404.

2-*O*-*p*-Methoxybenzyl-3,4-di-*O*-pivaloyl-L-fucopyranosyl dibutyl phosphate (7)

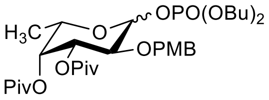

Compound **17** (2.72 g, 5.86 mmol) was dissolved in CH_2Cl_2 (136 mL), dibutyl phosphate (1.39 mL, 7.03 mmol) was added at 0 °C and stirred for 90 min. After completion of the reaction the mixture was evaporated to dryness. The resulting crude product was purified by silica gel column chromatography (pentane / EtOAc, 5:1 → 1:1) to yield 3.6 g (96%, α / β 2:1) of **7** as a viscous colorless oil (The analytical data is for the β anomer).

$[\boldsymbol{\alpha}]_{\mathbf{D}}^{\mathbf{20}}$ = - 27.8° (c = 0.79, $CHCl_3$).

^{1}H NMR (300 MHz, $CDCl_3$): δ (ppm) 0.88 (m_c, 6 H), 1.10 (s, 9 H), 1.12 (d, J = 6.6 Hz, 3 H), 1.26 (s, 9 H), 1.29–1.41 (m, 4 H), 1.62 (m_c, 4 H), 3.70 (dd, J = 7.7, 10.3 Hz, 1 H), 3.77 (s, 3 H), 3.89 (q, J = 6.0, 1 H), 3.98-4.11 (m, 4 H), 4.58 (d, J = 10.7 Hz, 1 H), 4.74 (d, J = 10.6 Hz, 1 H), 5.00 (dd, J = 10.3, 3.2 Hz, 1 H), 5.17 (t, J = 7.5 Hz, 1 H, H-1), 5.22 (m_c, 2 H), 6.82 (m_c, 2 H), 7.20 (d, J = 8.7 Hz, 2 H).

^{13}C NMR (75 MHz, $CDCl_3$): δ (ppm) 13.6, 15.9, 18.6, 27.1, 27.2, 27.2, 32.0, 32.1, 32.1, 32.2, 38.7, 39.0, 55.2, 67.7, 69.9, 70.0, 72.7., 74.3., 76.0, 98.7–98.8 (d, $^2J_{C,P}$ = 6.3 Hz, 1 C, C-1), 113.6, 129.0, 129.7, 159.0, 177.0.

IR (Film): $\tilde{\nu}$ (cm^{-1}) 2963, 2935, 2909, 2874, 1737, 1613, 1586, 1514, 1480, 1463, 1396.

HR-MS (ESI): m/z calcd. for $C_{32}H_{53}O_{11}PNa^+$ 667.3218, found: 667.3229.

3.4. Synthesis of the fucosyl acceptor 9.

***n*-Pentenyl 4-*O*-Acetyl-2-*O*-benzyl-*β*-L-fucopyranoside (9)**

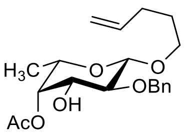

Compound **15** was prepared base on references [6, 7], to a solution of **15** (3.1 g, 9.63 mmol) in DMF (55.6 mL) were added trimethylorthoacetate (1.45 mL, 11.56 mmol) and *p*-TsOH (44 mg, 0.23 mmol). After stirring for 1 h at 50 °C the volatiles were removed to afford the corresponding orthoester. Further it was dissolved in an aq. solution of AcOH (80%, 55.6 mL) and stirred for 20 min at room temperature. Volatiles were removed and the resulting crude product was purified by silica gel column chromatography (pentane / EtOAc, 5:1 → 1:1) to yield 3.26 g (93%) of **9** as a viscous colorless oil.

$[\boldsymbol{\alpha}]_{\mathbf{D}}^{\mathbf{24}}$= - 14.6° (c = 0.55, $CHCl_3$).

^{1}H NMR (300 MHz, $CDCl_3$): δ (ppm) 1.20 (d, J = 6.5 Hz, 3 H), 1.78 (m_c, 2 H), 2.14 (s, 3 H), 2.15 (m_c, 2 H), 3.06 (bs, 1 H), 3.45-3.58 (m, 3 H), 3.65-3.70 (m, 1 H), 3.93-4.02 (m, 1 H), 4.36 (d, J = 7.7 Hz, 1 H, H-1), 4.67 (d, J = 11.2 Hz, 1 H), 4.95-5.01 (m, 2 H), 5.05, (q, J = 1.7 Hz, 1 H), 5.16 (dd, J = 3.6, 1 Hz, 1 H), 5.82 (m_c, 1 H), 7.27-7.37 (m, 5 H).

^{13}C NMR (75 MHz, $CDCl_3$): δ (ppm) 16.0, 20.7, 29.1, 30.2, 69.3, 69.6, 72.0, 72.3, 74.8, 79.2, 104.0 (C–1), 114.7, 127.9, 128.1, 128.6, 138.0, 138.4, 171.4.

IR (ATR): $\tilde{\nu}$ (cm^{-1}) 3454, 3065, 3030, 2981, 2938, 2870, 1739, 1640, 1497, 1454, 1371.

HR-MS (ESI): m/z calcd. for $C_{20}H_{28}O_6Na^+$ 387.1778, found: 387.1779.

3.5. Synthesis of the galactosyl donor 10.

3,6-Di-*O*-benzyl-4-*O*-pivaloyl-D-galactal (35)

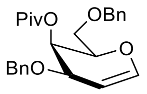

Compound **34** (4.5 g, 13.8 mmol) and DMAP (6.73 g, 55.2 mmol) in CH_2Cl_2 (134 mL) was cooled to 0 °C and PivCl (3.4 ml, 27.6 mmol) was added. The reaction mixture was stirred overnight at room temperature. It was diluted with a mixture of pentane and EtOAc (1:1) and filtered through a plug of silica gel. Volatiles were removed and the resulting crude product was purified by silica gel column chromatography (pentane / EtOAc, 15:1) to afforded 5.09 g (90%) of **35** as a colorless oil.

$[\alpha]_D^{20}$= + 6.9° (c = 0.26, $CHCl_3$).

^{1}H NMR (300 MHz, $CDCl_3$): δ (ppm) 1.17 (s, 9 H), 3.52 (dd, *J* = 9.9, 6.0 Hz, 1 H), 3.63 (dd, *J* = 9.9, 6.9 Hz, 1 H), 4.20 (t, *J* = 6.4 Hz, 1 H), 4.23 (m, 1 H), 4.44 (d, *J* = 11.7 Hz, 1 H), 4.49 (d, *J* = 11.9 Hz, 1 H), 4.59 (d, *J* = 11.9 Hz, 1 H), 4.64 (d, *J* = 11.7 Hz, 1 H), 4.73 (dt, *J* = 6.3, 1.9 Hz, 1 H), 5.54 (m_c, 1 H), 6.38 (dd, *J* = 6.4, 1.9 Hz, 1 H), 7.24–7.40 (m, 10 H).

^{13}C NMR (75 MHz, $CDCl_3$): δ (ppm) 27.1, 39.0, 62.8, 68.5, 69.8, 70.9, 73.6, 74.4, 101.1, 127.5, 127.7, 127.8, 127.9, 128.3, 128.5, 137.5, 138.1, 144.1, 177.8.

IR (ATR): $\tilde{\nu}$ (cm^{-1}) 3030, 2969, 2907, 2869, 1727, 1650, 1479, 1454, 1396, 1367, 1336.

HR-MS (ESI): m/z calcd. for $C_{25}H_{30}O_5Na^+$ 433.1985, found: 433.1985.

2-Azido-3,6-di-*O*-benzyl-2-deoxy-4-*O*-pivaloyl-D-galactopyranosyl nitrate (36)

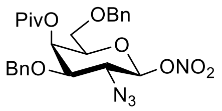

Compound **35** (1.70 g, 4.14 mmol) was dissolved in CH_3CN (25.0 mL). Sodium azide (400 mg, 6.15 mmol) and CAN (7.94 g, 14.5 mmol) were added at - 20 °C and the mixture was stirred for 3.5 h. Silica gel column chromatography (pentane / EtOAc, 18:1) afforded 945 mg (45%) of compound **36** as a pale yellow oil (The analytical data is for the β anomer).

^{1}H NMR (400 MHz, $CDCl_3$): δ (ppm) 1.15 (s, 9 H), 3.44 (dd, *J* = 9.6, 7.0 Hz, 1 H), 3.51 (dd, *J* = 9.6, 6.0 Hz, 1 H), 3.95 (dd, *J* = 10.7, 4 Hz, 1 H), 4.26 (dt, *J* = 7.0, 6.4, 1.4 Hz, 1 H), 4.42-4.53 (m, 3 H), 4.76 (d, *J* = 10.4 Hz, 1 H), 5.73 (dd, *J* = 2.9, 1.3 Hz, 1 H), 6.28 (d, *J* = 4.0 Hz, 1 H), 7.27-7.36 (m, 10 H).

^{13}C NMR (100 MHz, $CDCl_3$): δ (ppm) 27.1, 39.1, 57.8, 65.1, 67.5, 71.0, 71.6, 73.8, 74.7, 97.5, 127.9, 127.9, 128.1, 128.4, 128.4, 128.5, 136.6, 137.3, 176.9.

IR (ATR): $\tilde{\nu}$ (cm^{-1}) 3064, 3032, 2970, 2930, 2871, 2114, 1734, 1701, 1653, 1598, 1585.

HR-MS (ESI): m/z calcd. for $C_{25}H_{30}N_4O_8Na^+$ 537.1955, found: 537.1953.

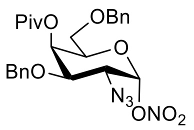

The analytical data is for the α anomer.

^{1}H NMR (300 MHz, $CDCl_3$): δ (ppm) 1.17 (s, 9 H), 3.47 (dd, J = 9.6, 7.1 Hz, 1 H), 3.53-3.58 (m, 2 H), 3.62 (dd, J = 9.9, 8.3 Hz, 1 H), 3.88 (ddd, J = 7.0, 5.8, 1.1 Hz, 1 H), 4.42-4.54 (m, 3 H), 4.76 (d, J = 10.9 Hz, 1 H), 5.46 (d, J = 8.4 Hz, 1 H), 5.62 (dd, J = 2.9, 1.1 Hz, 1 H), 7.28-7.36 (m, 10 H).

^{13}C NMR (75 MHz, $CDCl_3$): δ (ppm) 27.1, 39.1, 59.3, 64.1, 67.3, 71.8, 73.5, 73.8, 78.4, 98.1, 128.0, 128.1, 128.4, 128.4, 128.5, 136.6, 137.3, 176.9.

IR (ATR): $\tilde{\nu}$ (cm^{-1}) 3064, 3032, 2970, 2930, 2871, 2114, 1734, 1701, 1653, 1598, 1585.

HR-MS (ESI): m/z calcd. for $C_{25}H_{30}N_4O_8Na^+$ 537.1956, found: 537.1952.

2-Azido-3,6-di-*O*-benzyl-2-deoxy-4-*O*-pivaloyl-D-galactalopyranosyl trichloracetimidate (10)

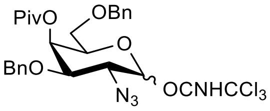

The solution of **36** (2.68 g, 5.21 mmol) in CH_2Cl_2 (60.0 mL) was cooled to 0 °C, PhSH (1.59 mL, 15.6 mmol) and DIEA (0.900 mL, 5.21 mmol) were added and stirred for 90 min. The volatiles were removed and the resulting crude product was purified by silica gel column chromatography (pentane/EtOAc, 2:1) to obtain 2.12 g (87%) of hemiacetal **37** as a pale yellow oil. Then, compound **37** (2.70 g, 5.75 mmol) was dissolved in CH_2Cl_2 (55.0 mL), CCl_3CN (11.5 mL, 0.115 mmol) and DBU (130 µL, 0.860 mmol) were added. The reaction mixture was stirred for 30 min at room temperature and volatiles were removed. Purification by silica gel column chromatography (pentane / EtOAc, 7:1) yielded 3.22 g (91%) of trichloroacetimidate **10** as a light yellow oil (The analytical data is for α anomer).

$[\boldsymbol{\alpha}]_{\mathbf{D}}^{\mathbf{20}}$= + 33.9° (c = 0.36, $CHCl_3$).

^{1}H NMR (600 MHz, $CDCl_3$): δ (ppm) 1.17 (s, 9 H), 3.47 (dd, J = 9.5, 7.5 Hz, 1 H), 3.56 (dd, J = 9.5, 5.8 Hz, 1 H), 3.86 (dd, J = 10.6, 3.4 Hz, 1 H), 4.07 (dd, J = 10.6, 3.0 Hz, 1 H), 4.31- 4.35 (m, 1 H), 4.43 (d, J = 11.7 Hz, 1 H), 4.47 (d, J = 10.4 Hz, 1 H), 4.50 (d, J = 11.7 Hz, 1 H), 4.82 (d, J = 10.4 Hz, 1 H), 5.80 (dd, J = 3.0, 1.3 Hz, 1 H), 6.40 (d, J = 3.5 Hz, 1 H), 7.25-7.39 (m, 10 H), 8.70 (s, 1 H).

^{13}C NMR (150 MHz, $CDCl_3$): δ (ppm) 27.1, 39.0, 58.9, 65.2, 67.6, 70.7, 71.4, 73.6, 74.3, 90.8, 95.0, 127.8, 127.9, 128.2, 128.4, 128.5, 136.8, 137.4, 160.7, 177.1.

IR (ATR): $\tilde{\nu}$ (cm^{-1}) 3340, 3032, 2971, 2930, 2872, 2112, 1733, 1673, 1496, 1478, 1455.

HR-MS (ESI): m/z calcd. for $C_{27}H_{31}N_4O_6Cl_3Na^+$ 635.1201, found: 635.1201.

3.6. Synthesis of the mannosyl phosphate 8.

Dibutyl-(2-*O*-acetyl-3,4,6-tri-*O*-benzyl-α-D-mannopyranosyl) phosphate (8)

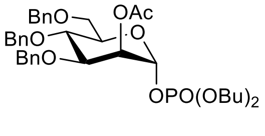

Compound **8** was prepared base on references [8-14]. Spectral data for final product **50** and all mannose Building Blocks were consistent with that previously reported.

3.7. Synthesis of the glucosyl donor 4.

4-Methoxyphenyl 3-*O*-benzyl-4,6-*O*-benzylidine-β-D-glucopyranoside (23)

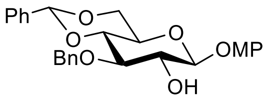

To a solution of triol **22** (2.64 g, 7.01 mmol) in MeCN (80.0 mL) benzaldehyde dimethyl acetal (3.37 mL, 22.44 mmol) and *p*-TsOH (33.0 mg, 175 μmol) were added at room temperature. After a reaction time of 15 min the mixture was quenched by the addition of saturated solution of $NaHCO_3$ and extracted with dichloromethane (3x). The combined organic layer was washed with brine, dried over $MgSO_4$ and concentrated to dryness. The colorless solid was subjected to the next step without further purification. To confirm the analytical data, the crude product was washed with cold diethyl ether and re-crystallized (EtOH) to afford 2.6 g (80%) of **23** as a colorless solid. The analytical data for compound **23** was in accordance with the reported data [15-18].

4-Methoxyphenyl 3,4-di-*O*-benzyl-2-*O*-pivaloyl-β-D-glucopyranoside 25

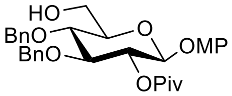

Compound **23** (3.06 g, 6.59 mmol) and DMAP (3.22 g, 26.36 mmol) in CH_2Cl_2 (73.0 mL) was cooled to 0 °C and PivCl (1.62 mL, 13.18 mmol) was added. The reaction mixture was stirred overnight at room temperature. Mixture was diluted with a mixture of pentane and EtOAc (1:1) and filtered through a plug of silica gel. Purification by silica gel column chromatography (pentane/EtOAc, 6:1) afforded 3.35 g (93%) of the corresponding glycoside as a colorless crystalline solid.

Compound **93** (1 g, 1.82 mmol) in THF (20.0 mL), BH_3·THF (1 M, 3.64 mL, 3.64 mmol) was added at 0 °C. After stirring for 5 min, Bu_2BOTf in CH_2Cl_2 (1 M, 0.364 mL, 0.364 mmol) was added dropwise and the reaction mixture was stirred overnight at room temperature. After completion of the reaction it was quenched by the addition of triethyl amine, followed by MeOH. The reaction mixture was evaporated to dryness and the resulting crude product was purified by silica gel column chromatography (pentane/EtOAc, 4:1) to afford 952 mg of **25** (95%) as a colorless crystalline solid.

$[\boldsymbol{\alpha}]_{\mathbf{D}}^{\mathbf{20}}$= – 18.0° (c = 0.46, $CHCl_3$).

^{1}H NMR (600 MHz, $CDCl_3$): δ (ppm) 1.18 (s, 9 H), 1.92 (dd, *J* = 7.8, 6.1 Hz, 1 H), 3.50-3.55 (m, 1 H), 3.75 (s, 3 H), 3.76-3.94 (m, 4 H), 4.62-4.80 (m, 4 H), 4.92 (d, *J* = 8.0 Hz, 1 H), 5.26 (dd, *J* = 9.1, 8.0 Hz, 1 H), 6.77-6.91 (m, 4 H), 7.24-7.35 (m, 10 H).

^{13}C NMR (150 MHz, $CDCl_3$): δ (ppm) 27.3, 38.9, 55.6, 61.8, 72.8, 75.0, 75.1, 75.5, 76.7, 76.9, 82.9, 100.3, 114.5, 117.8, 127.3, 127.6, 127.9, 127.9, 128.3, 128.4, 137.6, 137.8, 151.0, 155.2, 176.6.

IR (ATR): $\tilde{\nu}$ (cm^{-1}) 3594, 3089, 3064, 3028, 2997, 2958, 2931, 2907, 2875, 2834, 1719.

HR-MS (ESI): m/z calcd. for $C_{32}H_{38}O_8Na^+$ 573.2450, found: 573.2459.

Methyl (4-methoxyphenyl 3,4-di-O-benzyl-2-O-pivaloyl-β-D-glucopyranoside)uronate (26)

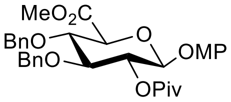

BAIB (644 mg, 2.00 mmol) and TEMPO (28 mg, 182.0 μmol) were added to the solution of compound **25** (500 mg, 0.910 mmol) in a 1:1 mixture of acetonitrile and water (10 mL) at room temperature. The reaction mixture was stirred overnight and diluted with EtOAc. The organic layer was successively washed with a solution of $Na_2S_2O_3$, $NaHCO_3$ and brine. The obtained organic layer was dried over $MgSO_4$, filtered, concentrated and purified by silica gel column chromatography (CH_2Cl_2 / MeOH, 1:0 → 5:1) to afford 368 mg (70%) of the corresponding acid as a colorless oil. Iodomethane (121.4 μL, 1.95 mmol) was added at room temperature under dry argon to a solution of the above isolated acid in dry DMF (5 mL) and K_2CO_3 (0.359 g, 2.6 mmol). The reaction mixture was stirred overnight and evaporated to dryness. The resulting crude product was diluted with EtOAc. The organic layer was extracted with H_2O and brine, dried over $MgSO_4$ and concentrated to dryness. Final product was recrystallized in ether/ hexane to furnish 264 mg (78%) of **26** as a colorless powder.

$[\alpha]_D^{20}$= – 26.1° (c = 0.87, $CHCl_3$).

^{1}H NMR (600 MHz, $CDCl_3$): δ (ppm) 1.18 (s, 9 H), 3.69 (s, 3 H), 3.74 (s, 3 H), 3.78-3.83 (m, 1 H), 4.03–4.05 (m, 2 H), 4.60–4.77 (m, 4 H), 4.90 (d, *J* = 7.5 Hz, 1 H), 5.31 (dd, *J* = 8.9, 7.5 Hz, 1 H), 6.79 (m_c, 2 H), 6.92(m_c, 2 H), 7.19–7.32 (m, 10 H).

^{13}C NMR (150 MHz, $CDCl_3$): δ (ppm) 27.1, 38.8, 52.6, 55.6, 72.6, 74.6, 74.9, 75.0, 78.7, 82.1, 100.8, 114.5, 118.3, 127.4, 127.7, 127.9, 128.0, 128.40, 128.4, 130.1, 133.6, 137.6, 137.8, 151.1, 155.5, 168.4, 176.7.

IR (ATR): $\tilde{\nu}$ (cm^{-1}) 3088, 3064, 3032, 2972, 2956, 2935, 2905, 2872, 1748, 1634, 1595.

HR-MS (ESI): m/z calcd. for $C_{33}H_{38}O_9Na^+$ 601.2408, found 601.2402.

Methyl 3,4-di-O-benzyl-2-*O*-pivaloyl-1-*O*-trichloroacetimidoyl-α-D-glucopyran-uronate (4)

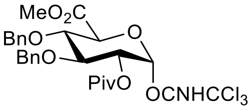

Compound **94** (539 mg, 0.931 mmol) was dissolved in a 1:1.5:1-mixture of toluene, acetonitrile and water (14.0 mL), respectively. CAN (2.55 g, 4.65 mmol) was added at room temperature and the reaction mixture was stirred for 45 min. After the completion of the reaction it was poured into an ice-water mixture and extracted with EtOAc (3x). The organic layer was successively washed with an aqueous solution of $NaHCO_3$ and water, dried over $MgSO_4$ and evaporated to dryness. The resulting crude product was purified by silica gel column chromatography (pentane/ EtOAc 2:1) to obtain 380 mg (87%) of a yellow oil. Compound 63 was dissolved in CH_2Cl_2 (10.0 mL), Cl_3CCN (1.61 mL, 16.0 mmol) followed by a catalytic amount of DBU (0.180 μL, 0.120 mmol) were added. Purification by silica gel column chromatography (pentane/EtOAc, 7:1) afforded 444 mg (90%) of **4** as a pale yellow solid.

$[\alpha]_D^{20}$= + 35.0° (c = 0.70, $CHCl_3$).

^{1}**H NMR** (600 MHz, $CDCl_3$): δ (ppm) 1.15 (s, 9 H), 3.70 (s, 3 H), 3.93 (dd, *J* = 10.1, 9.0 Hz, 1 H), 4.14 (t, *J* = 9.5 Hz, 1 H), 4.44 (d, *J* = 10.0 Hz, 1 H), 4.59 (d, *J* = 10.5 Hz, 1 H), 4.75-4.82 (m, 3 H), 5.12 (dd, *J* = 10.0, 3.6 Hz, 1 H), 6.58 (d, *J* = 3.6 Hz, 1 H, H-1), 7.19-7.32 (m, 10 H), 8.65 (s, 1 H).

^{13}**C NMR** (150 MHz, $CDCl_3$): δ (ppm) 27.0, 38.7, 52.6, 71.7, 72.5, 75.4, 75.54, 78.9, 78.9, 90.7, 93.2 (C–1), 127.4, 127.7, 128.0, 128.1, 128.4, 128.4, 137.3, 137.8, 160.5, 168.7, 177.3.

IR (ATR): $\tilde{\nu}$ (cm^{-1}) 3321, 3244, 3185, 3063, 3032, 2965, 2933, 2874, 1734, 1697, 1617.

HR-MS (ESI): m/z calcd. for $C_{28}H_{32}Cl_3NO_8Na^+$ 640.1058, found 640.1066.

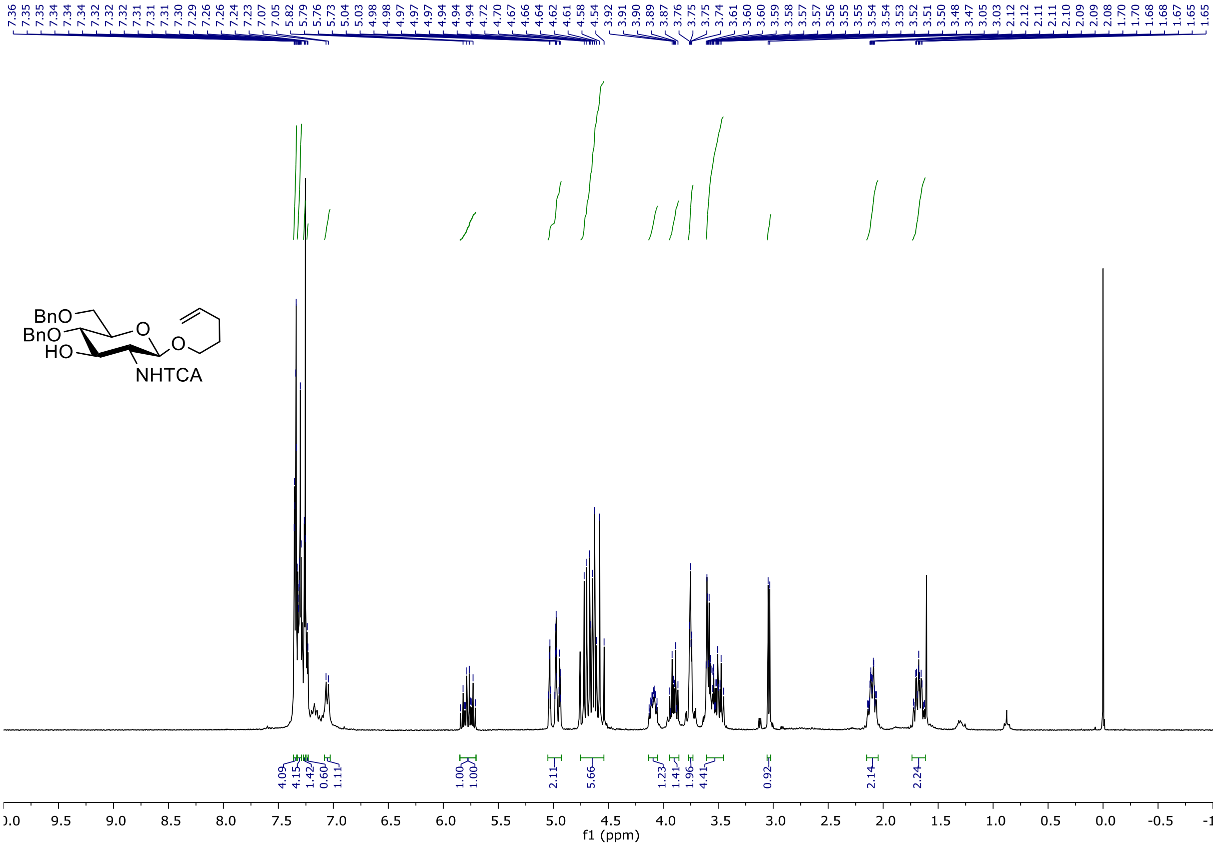

Figure S1. ^{1}H NMR ($CDCl_3$, 300 MHz) spectrum of **glycoside acceptor 5**

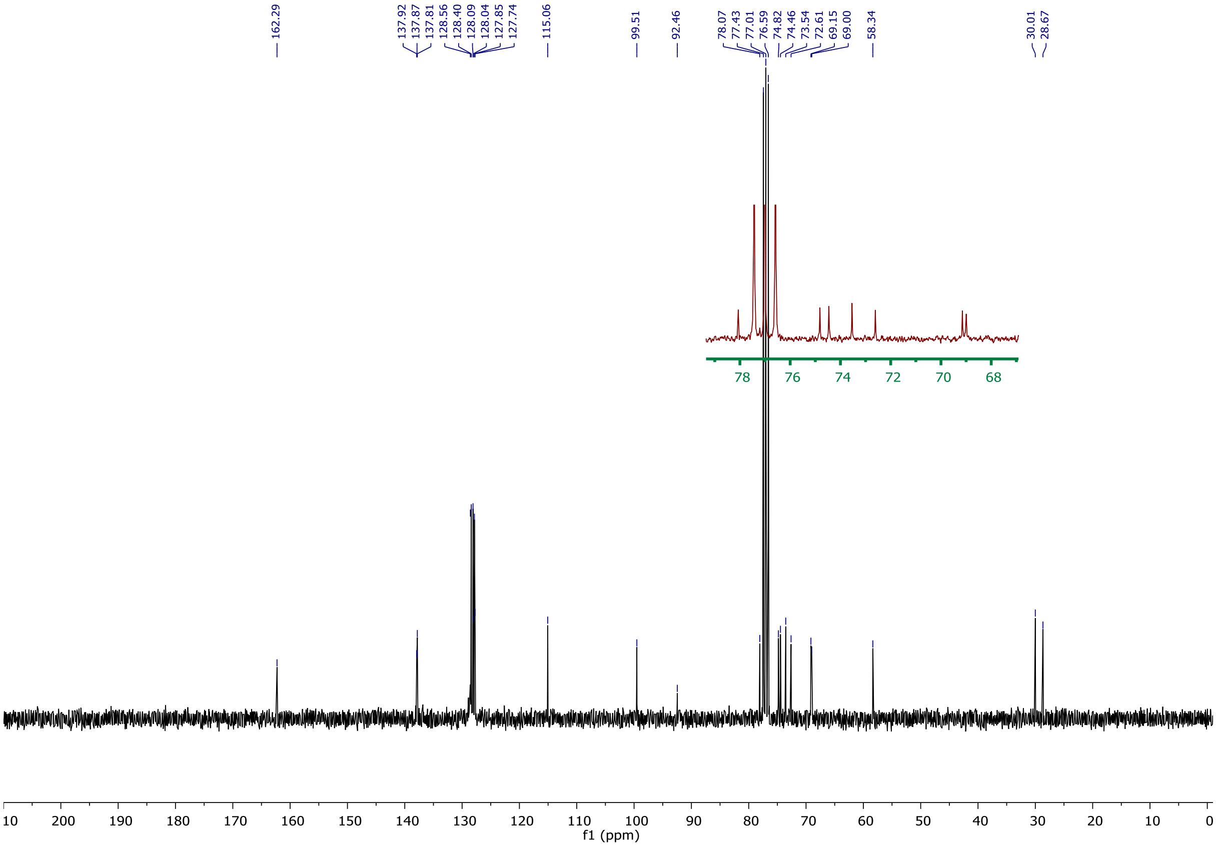

Figure S2. ^{13}C NMR ($CDCl_3$, 75 MHz) spectrum of **glycoside acceptor 5**

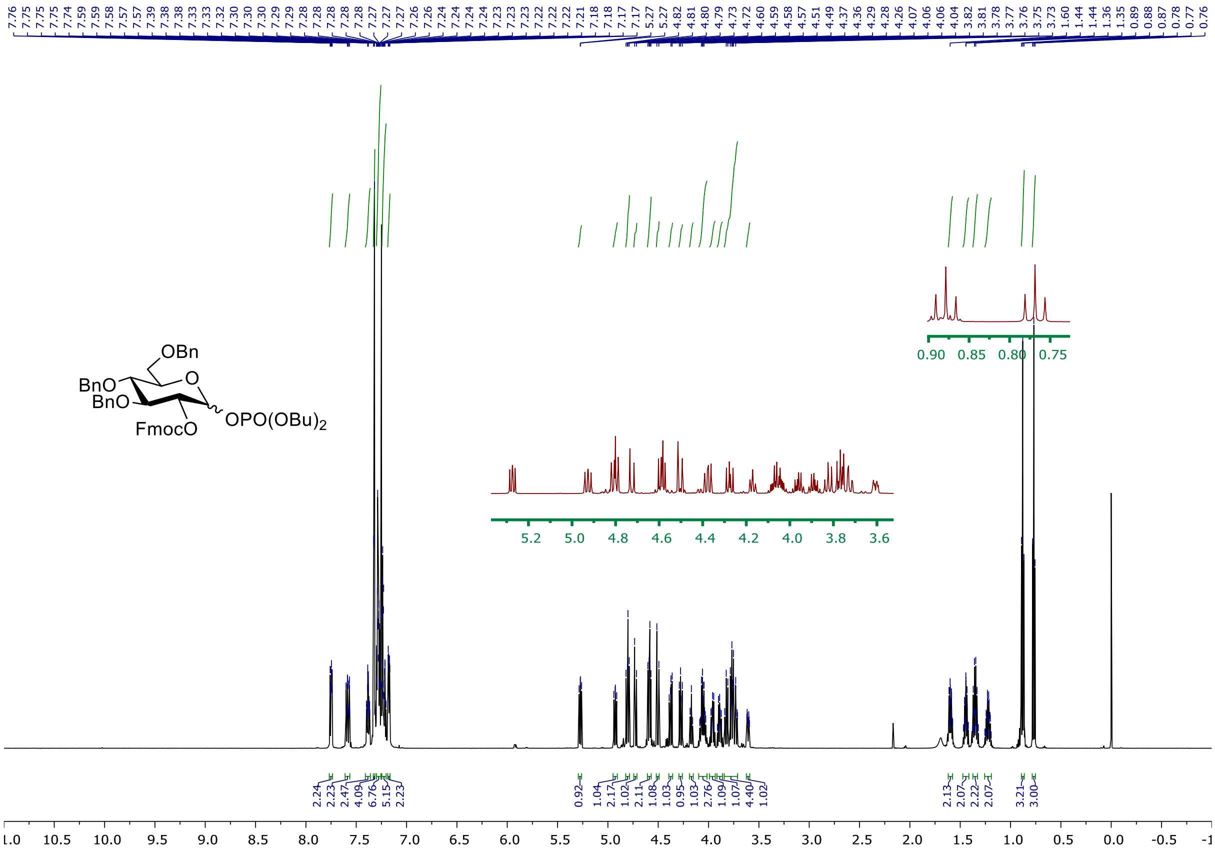

Figure S3. ^{1}H NMR ($CDCl_3$, 600 MHz) spectrum of **glycosyl donor 6**

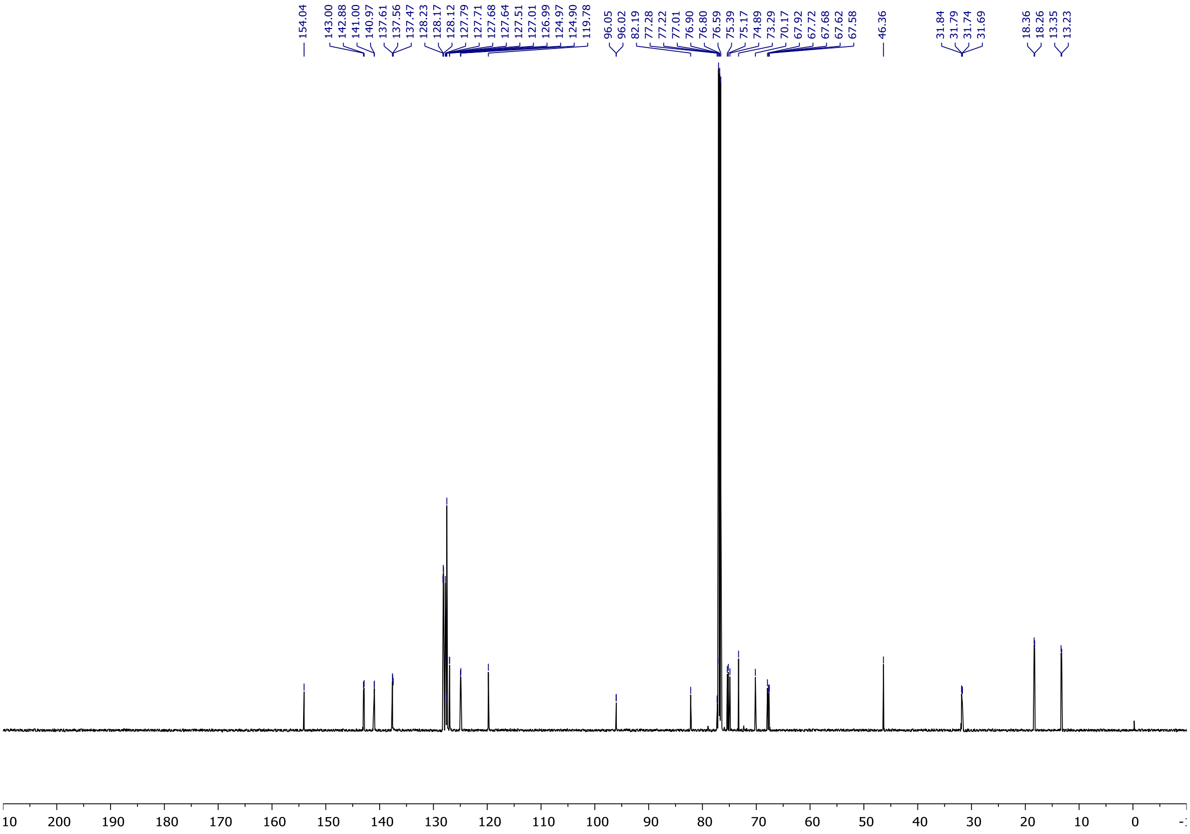

Figure S4. ^{13}C NMR ($CDCl_3$, 150 MHz) spectrum of **glycosyl donor 6**

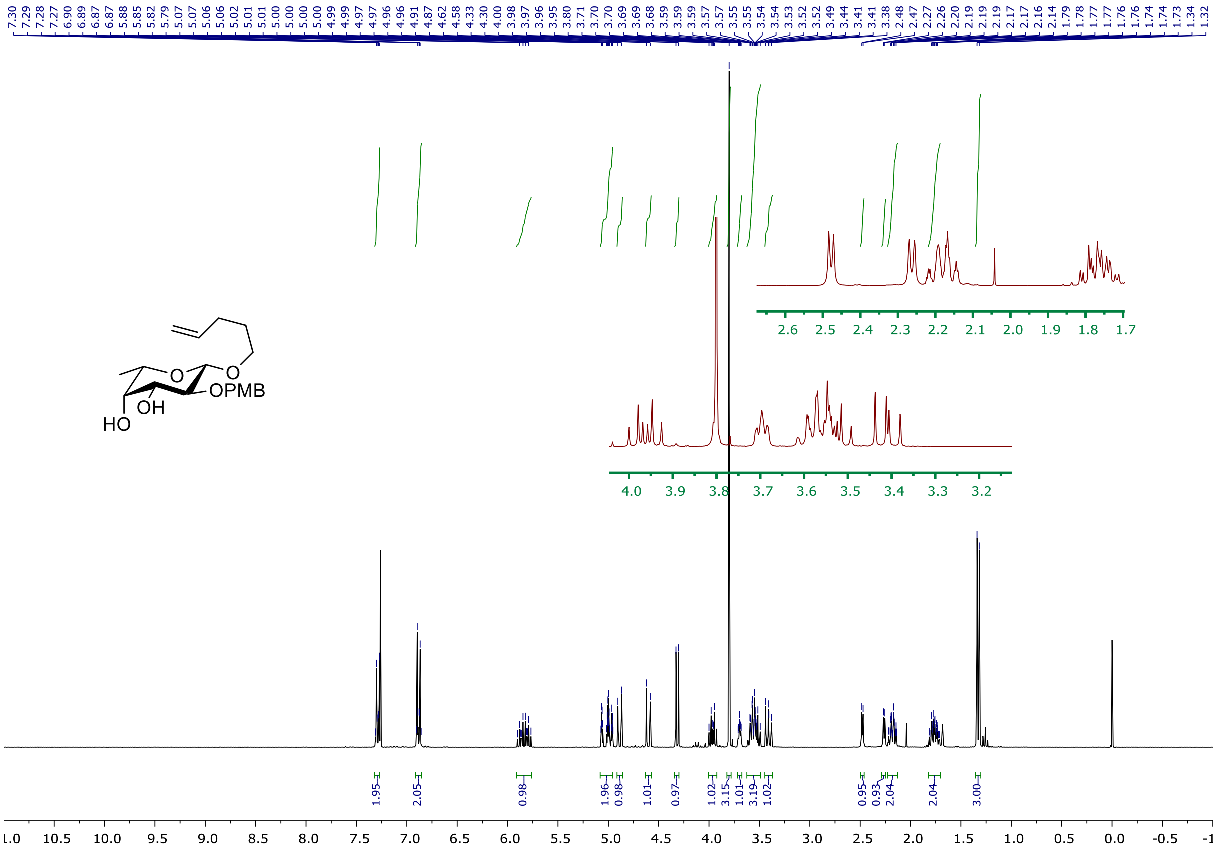

Figure S5. ^{1}H NMR ($CDCl_3$, 300 MHz) spectrum of **fucose building block 74**

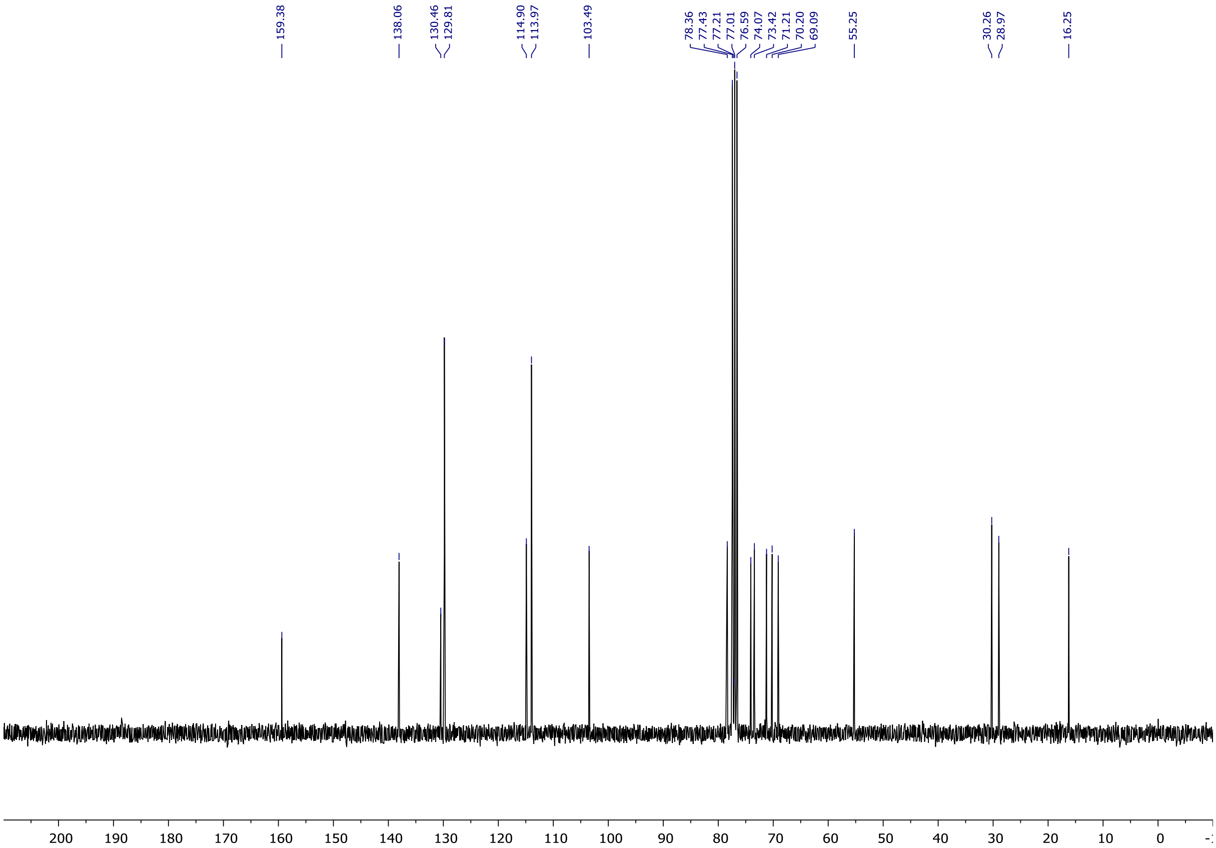

Figure S6. ^{13}C NMR ($CDCl_3$, 75 MHz) spectrum of **fucose building block 74**

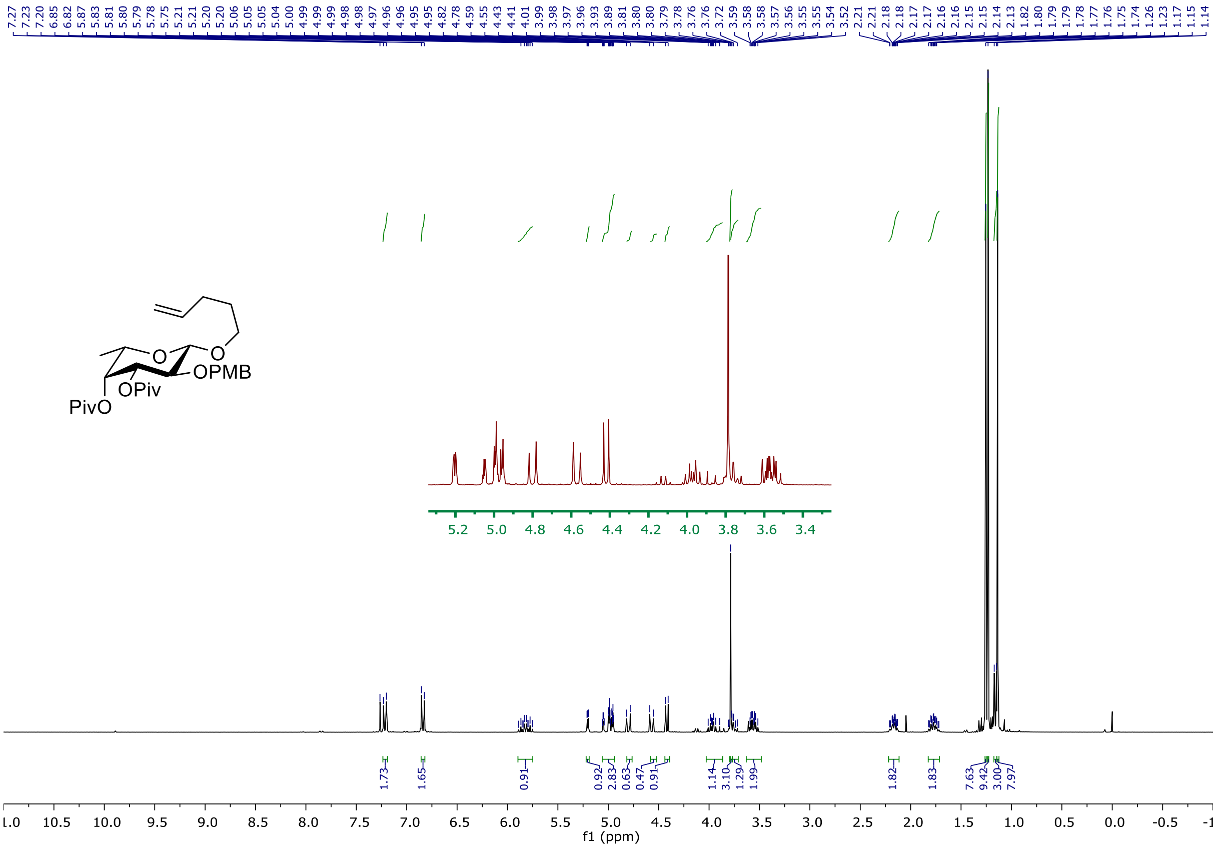

Figure S7. ^{1}H NMR ($CDCl_3$, 300 MHz) spectrum of **fucose building block 16**

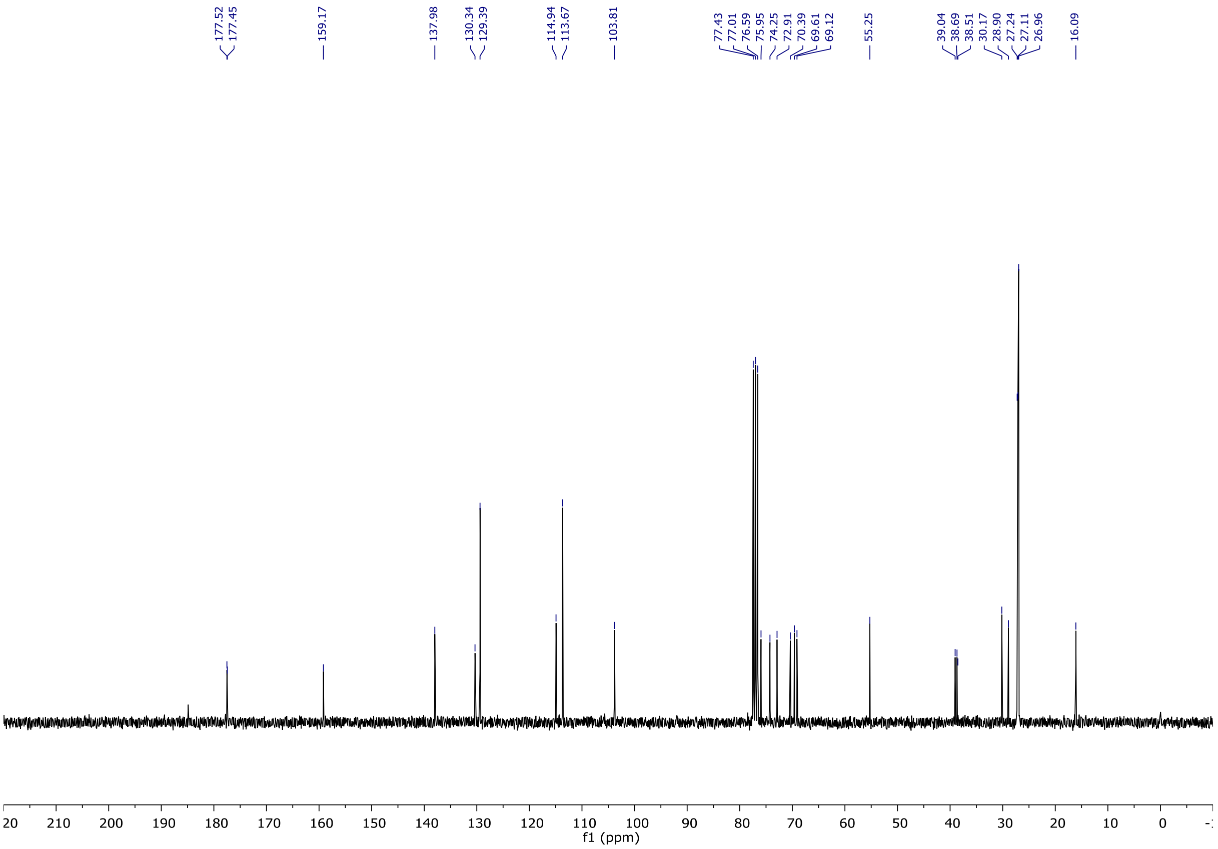

Figure S8. ^{13}C NMR ($CDCl_3$, 75 MHz) spectrum of **fucose building block 16**

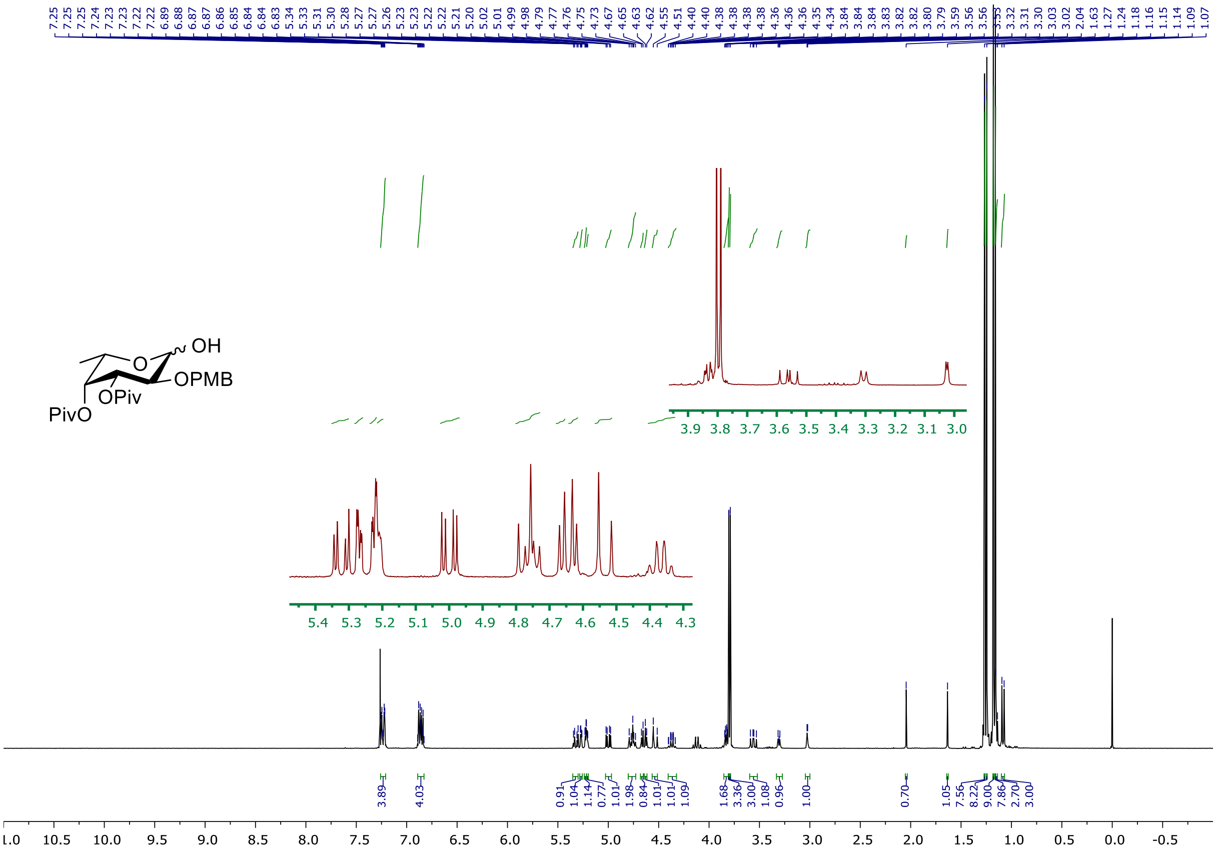

Figure S9. ^{1}H NMR ($CDCl_3$, 300 MHz) spectrum of **fucose building block 75**

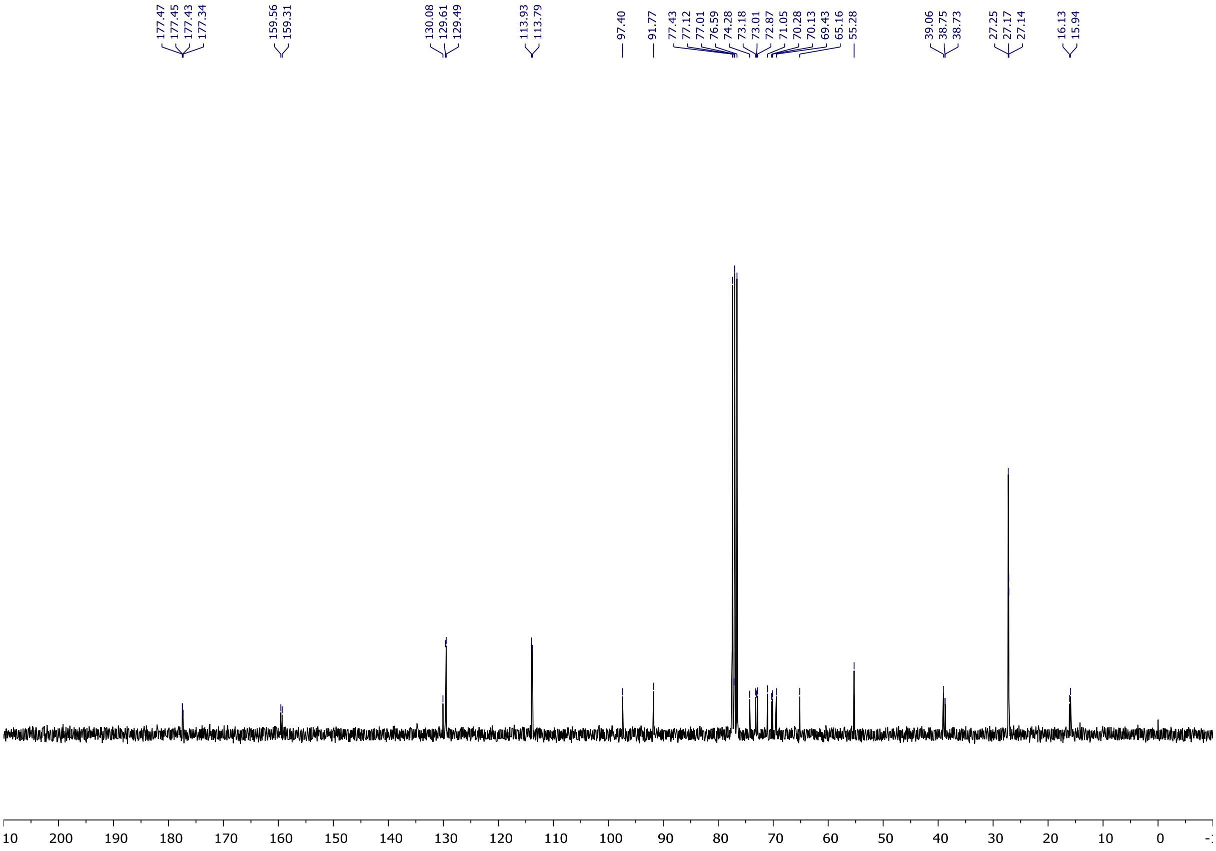

Figure S10. ^{13}C NMR ($CDCl_3$, 75 MHz) spectrum of **fucose building block 75**

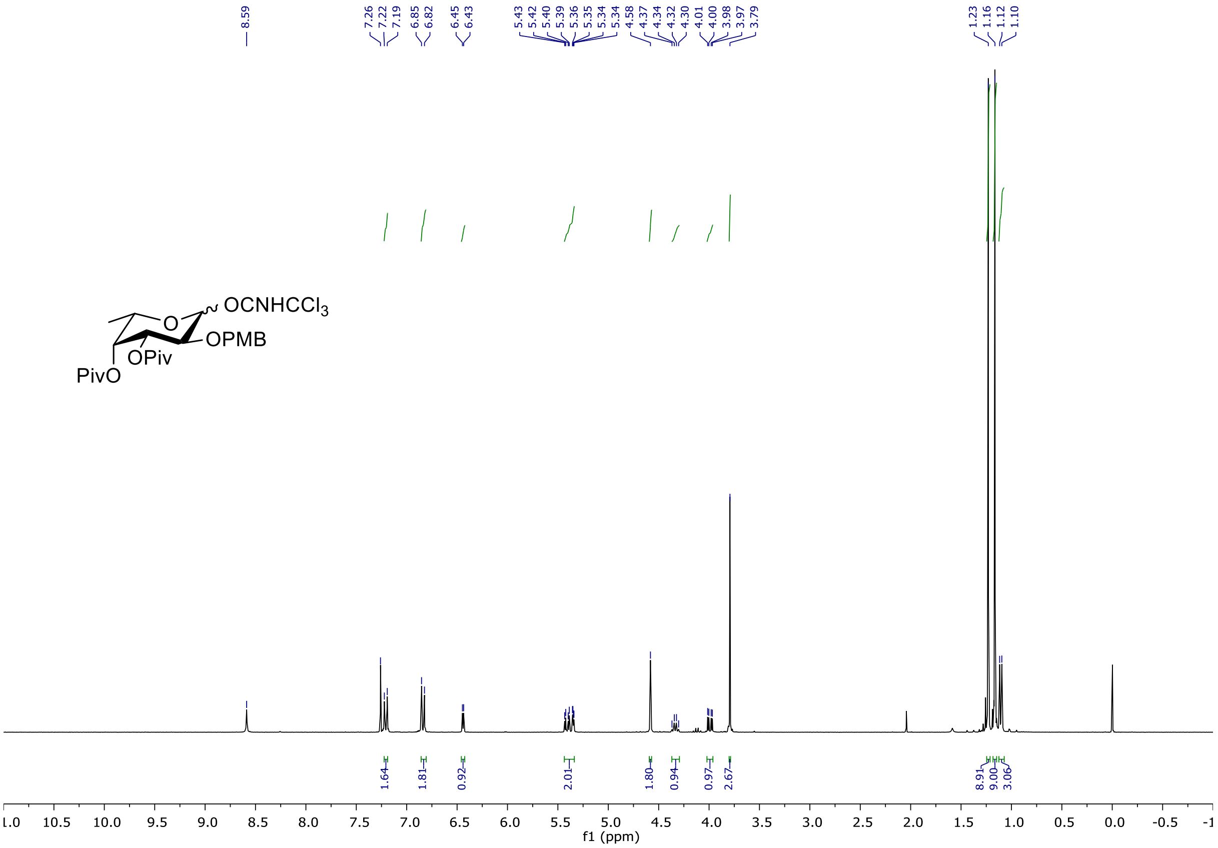

Figure S11. ^{1}H NMR ($CDCl_3$, 300 MHz) spectrum of **fucosyl donor 17**

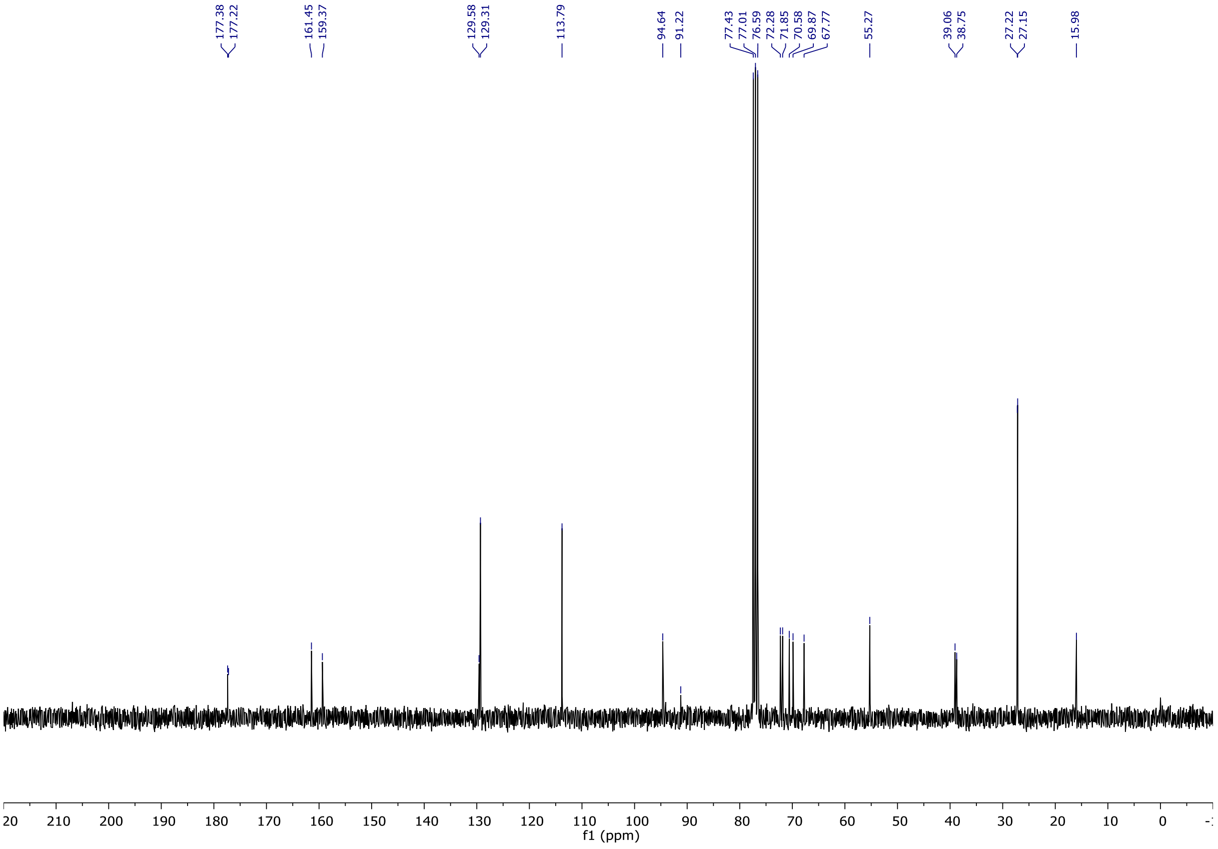

Figure S12. ^{13}C NMR ($CDCl_3$, 75 MHz) spectrum of **fucosyl donor 17**

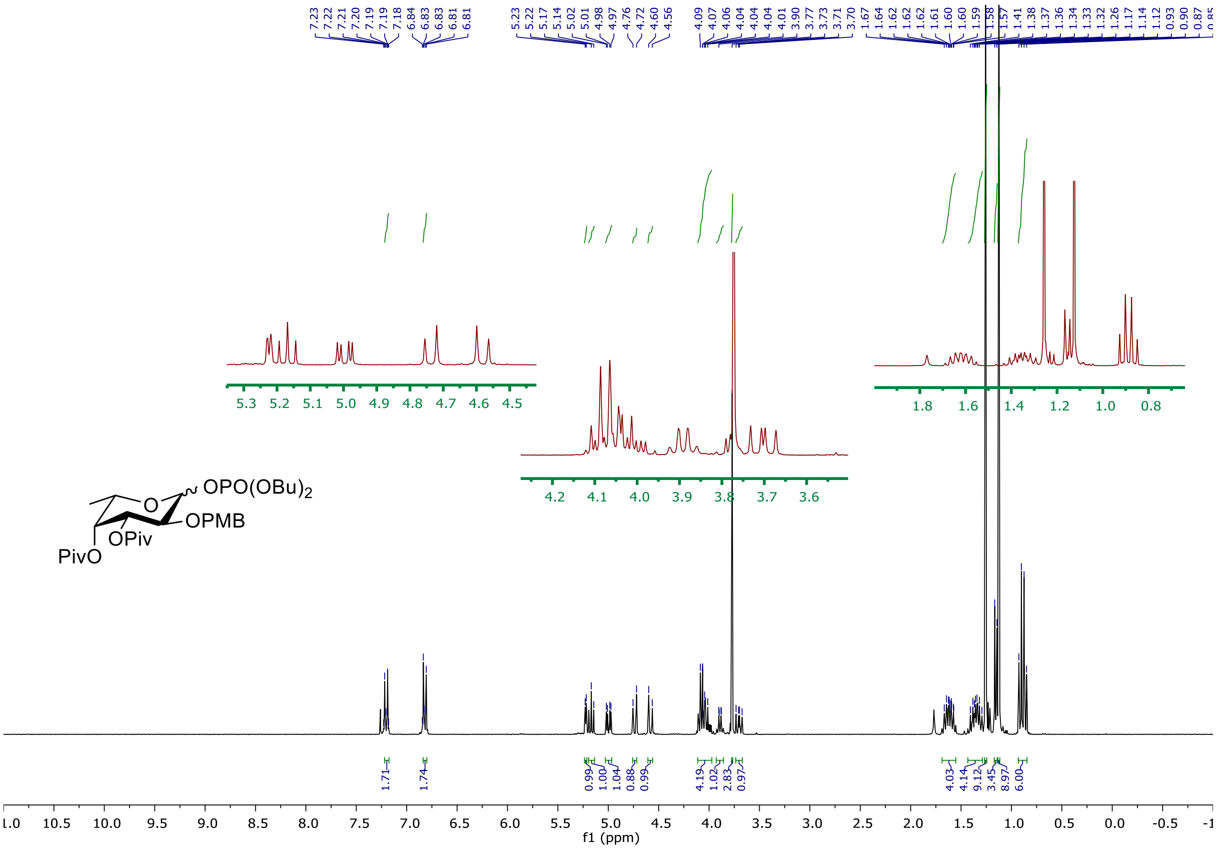

Figure S13. ^{1}H NMR ($CDCl_3$, 300 MHz) spectrum of **fucosyl donor 7**

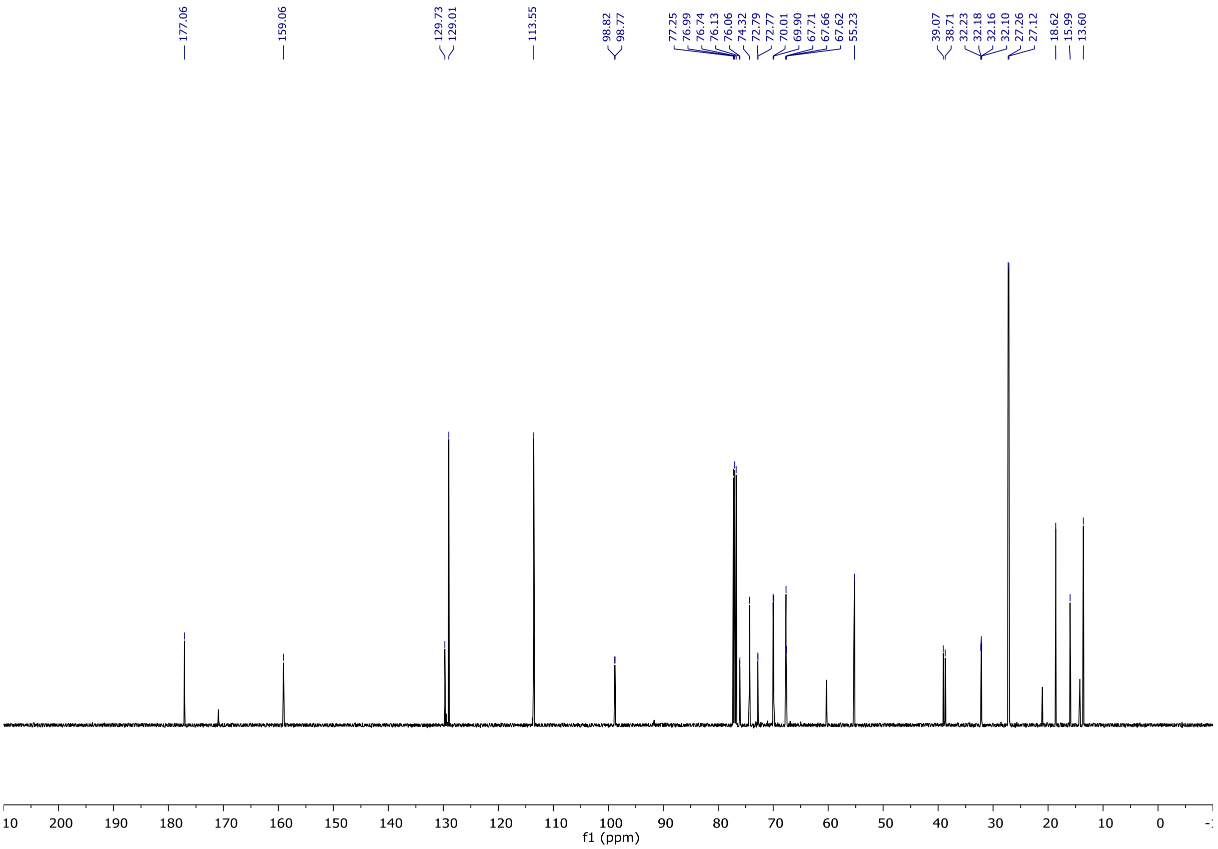

Figure S14. ^{13}C NMR ($CDCl_3$, 75 MHz) spectrum of **fucosyl donor 7**

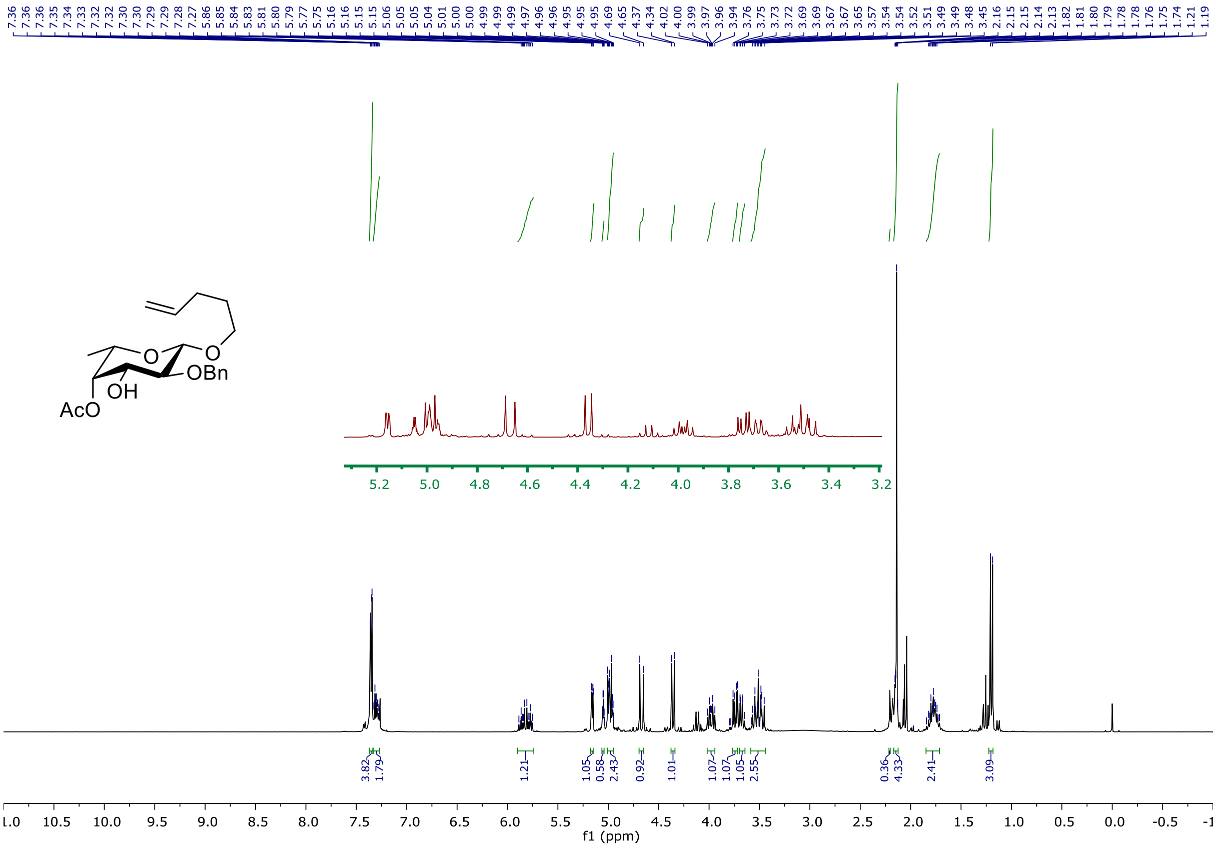

Figure S15. ^{1}H NMR ($CDCl_3$, 300 MHz) spectrum of **fucosyl acceptor 9**

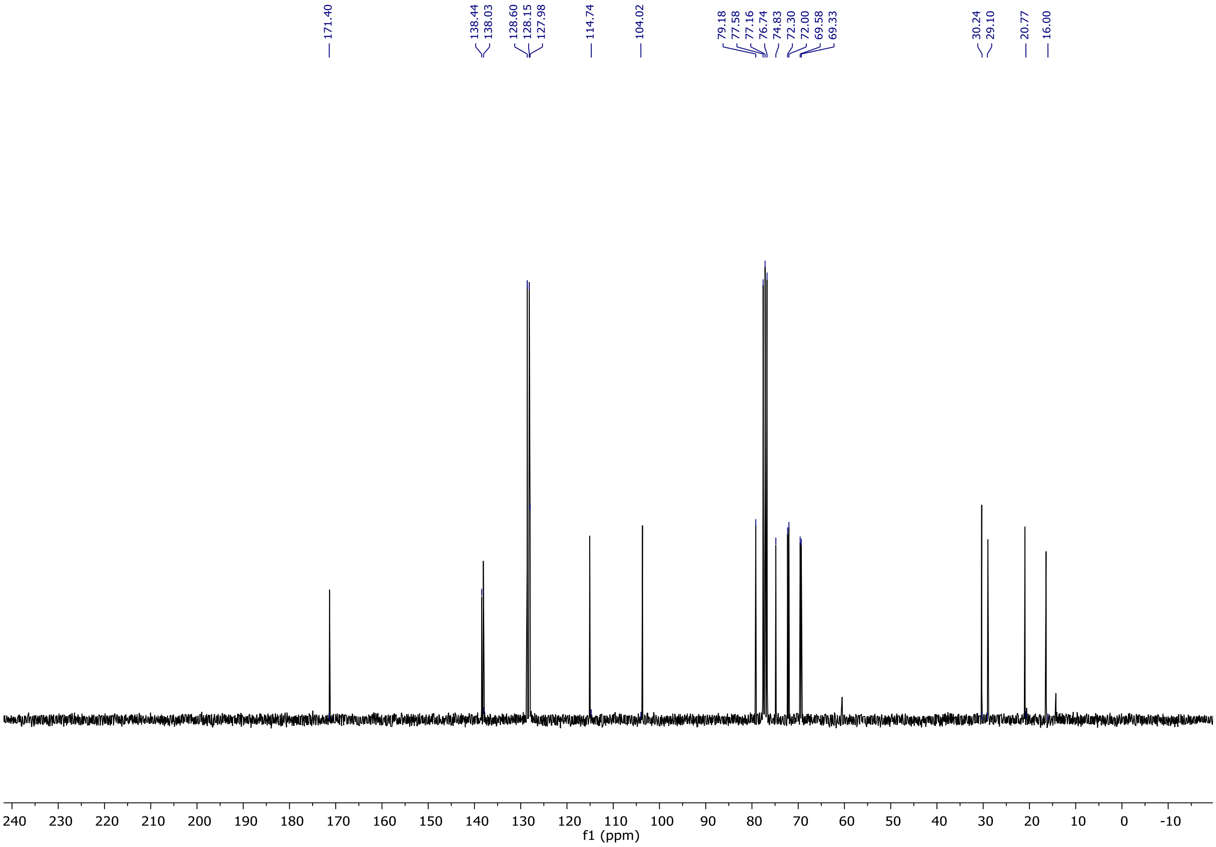

Figure S16. ^{13}C NMR ($CDCl_3$, 75 MHz) spectrum of **fucosyl acceptor 9**

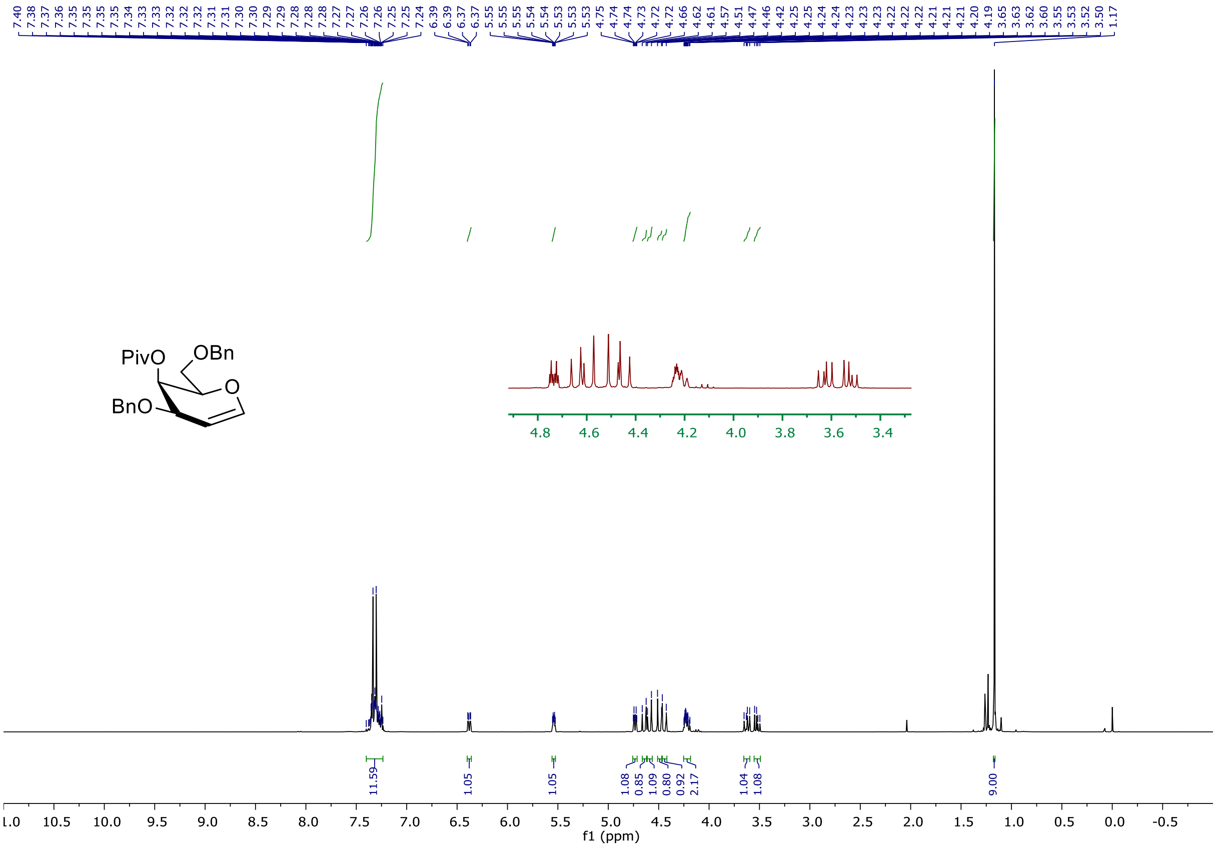

Figure S17. ^{1}H NMR ($CDCl_3$, 300 MHz) spectrum of **galactal 35**

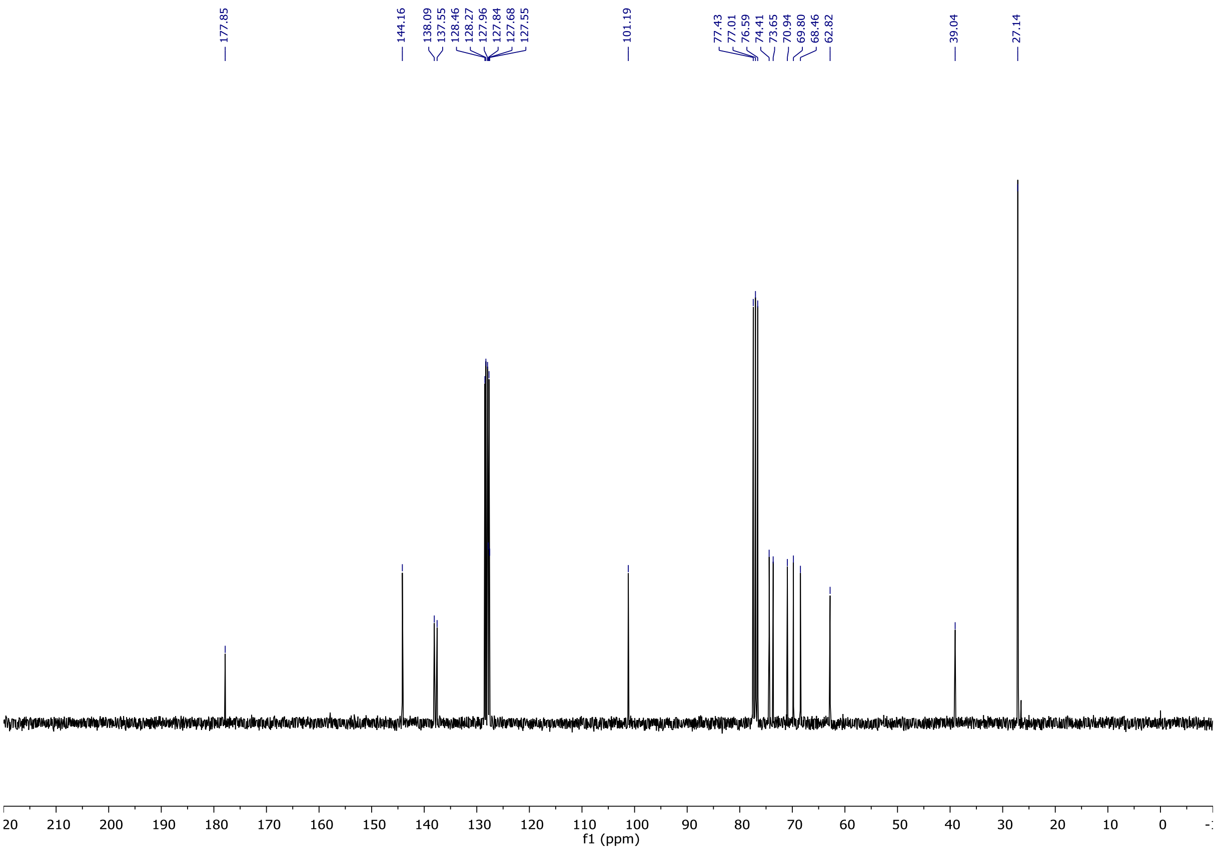

Figure S18. ^{13}C NMR ($CDCl_3$, 125 MHz) spectrum of **galactal 35**

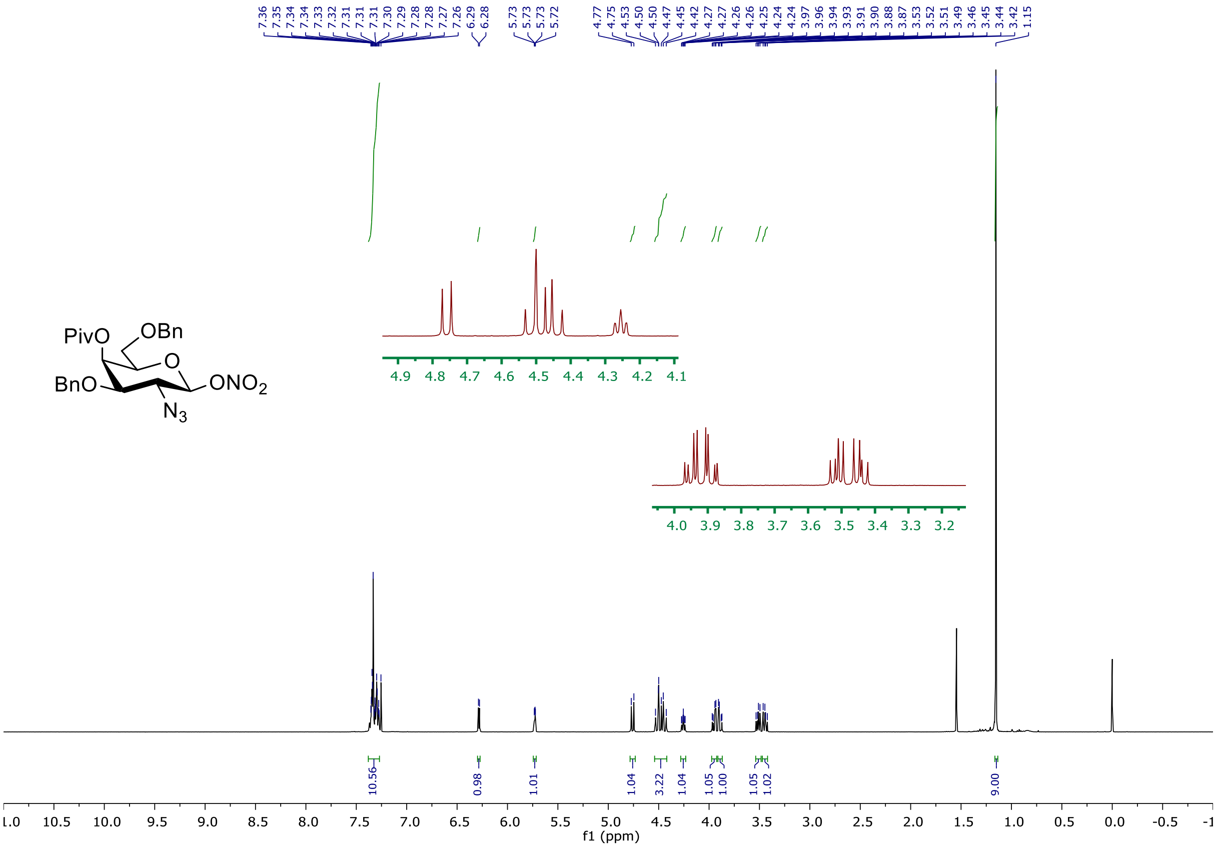

Figure S19. ^{1}H NMR ($CDCl_3$, 400 MHz) spectrum of **galactose building block 36 (*β*-anomer)**

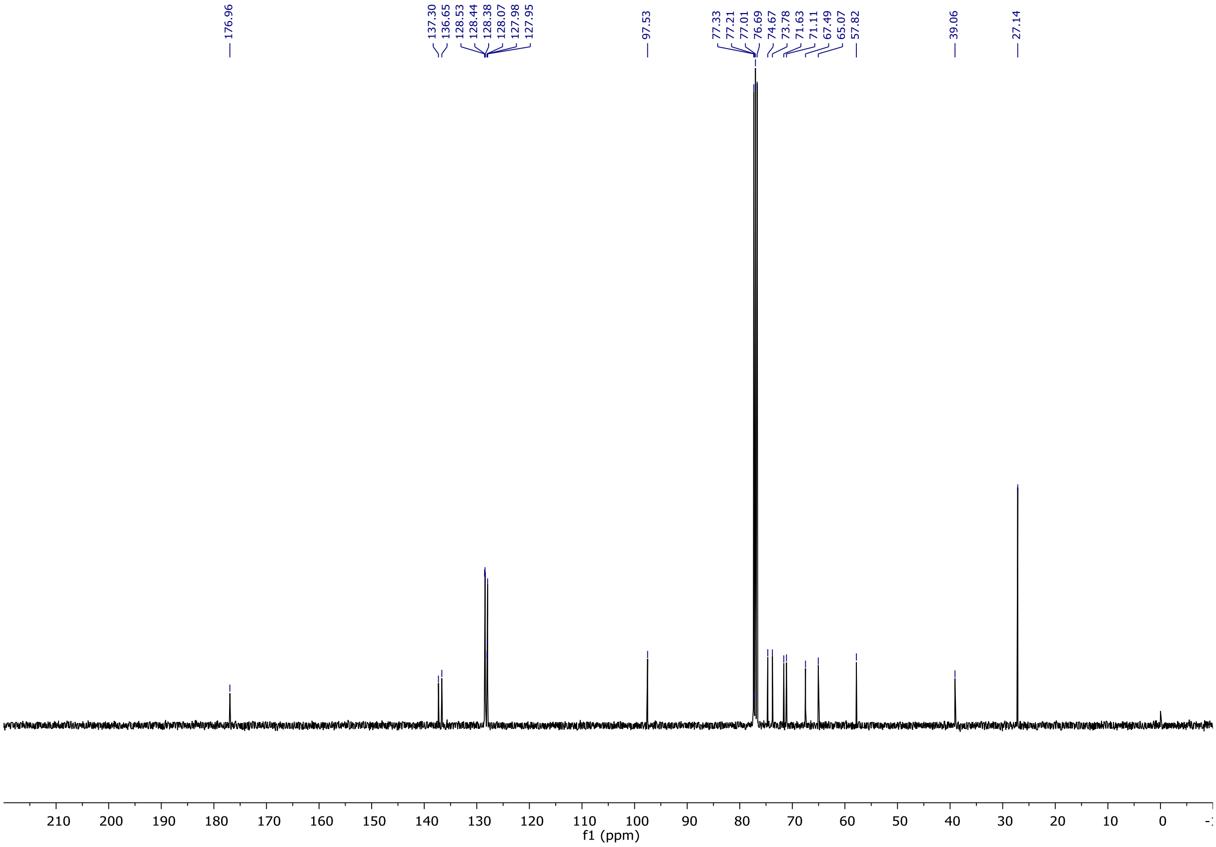

Figure S20. ^{13}C NMR ($CDCl_3$, 100 MHz) spectrum of **galactose building block 36 (*β* -anomer)**

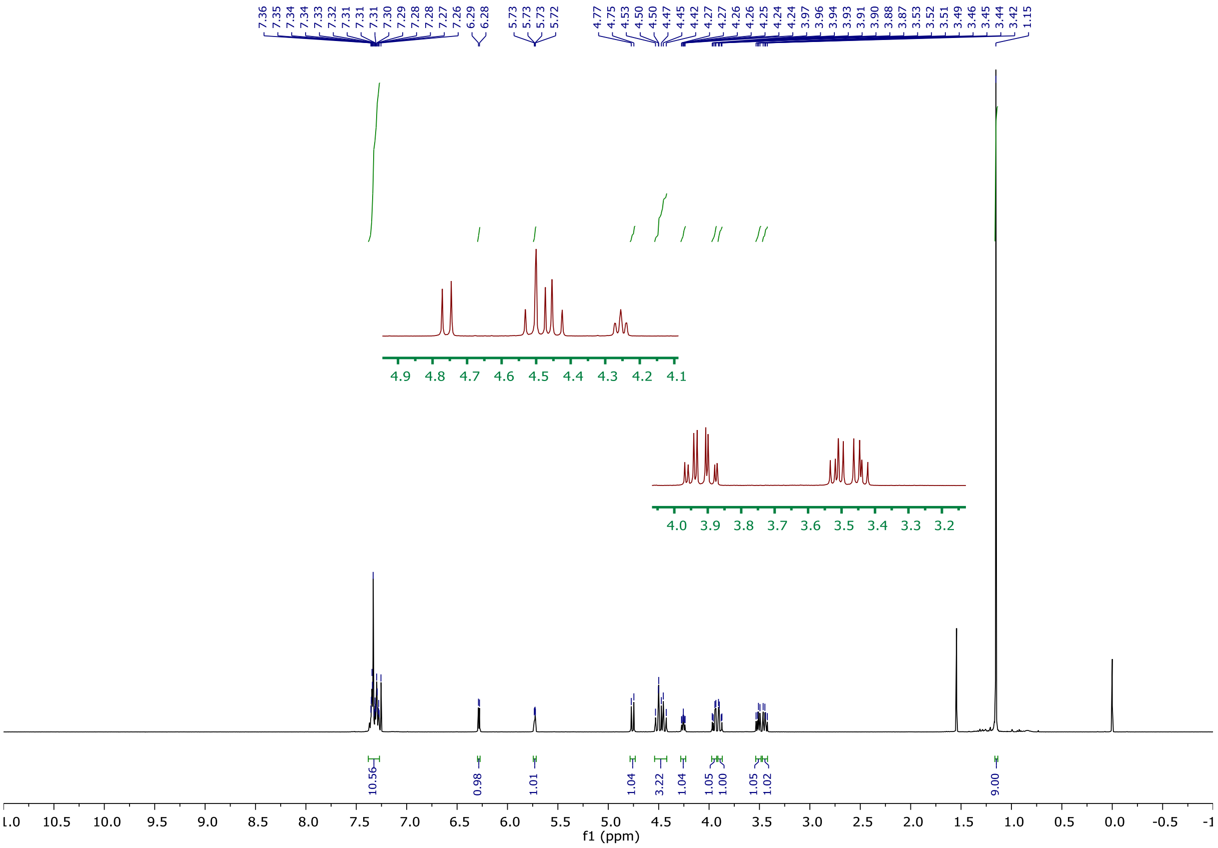

Figure S21. ^{1}H NMR ($CDCl_3$, 300 MHz) spectrum of **galactose building block 36 (α-anomer)**

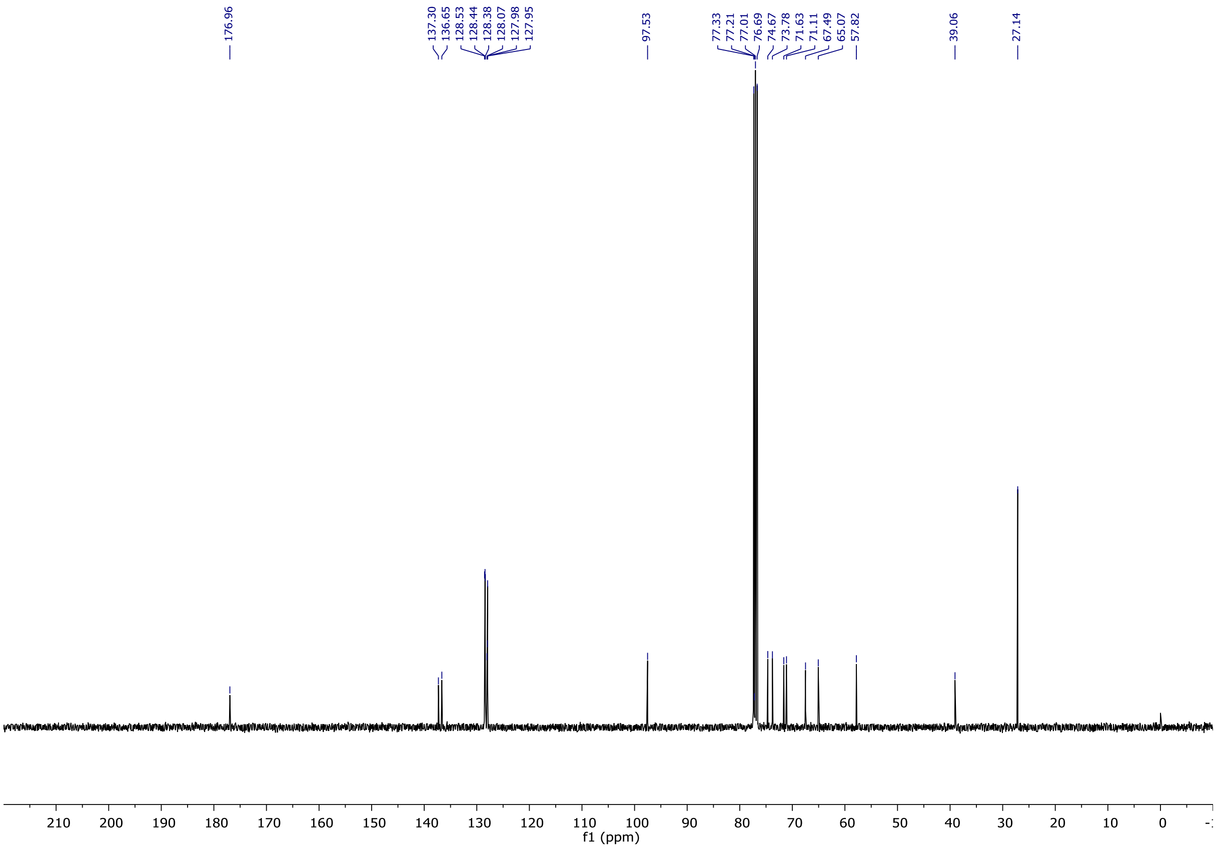

Figure S22. ^{13}C NMR ($CDCl_3$, 75 MHz) spectrum of **galactose building block 36 (α-anomer)**

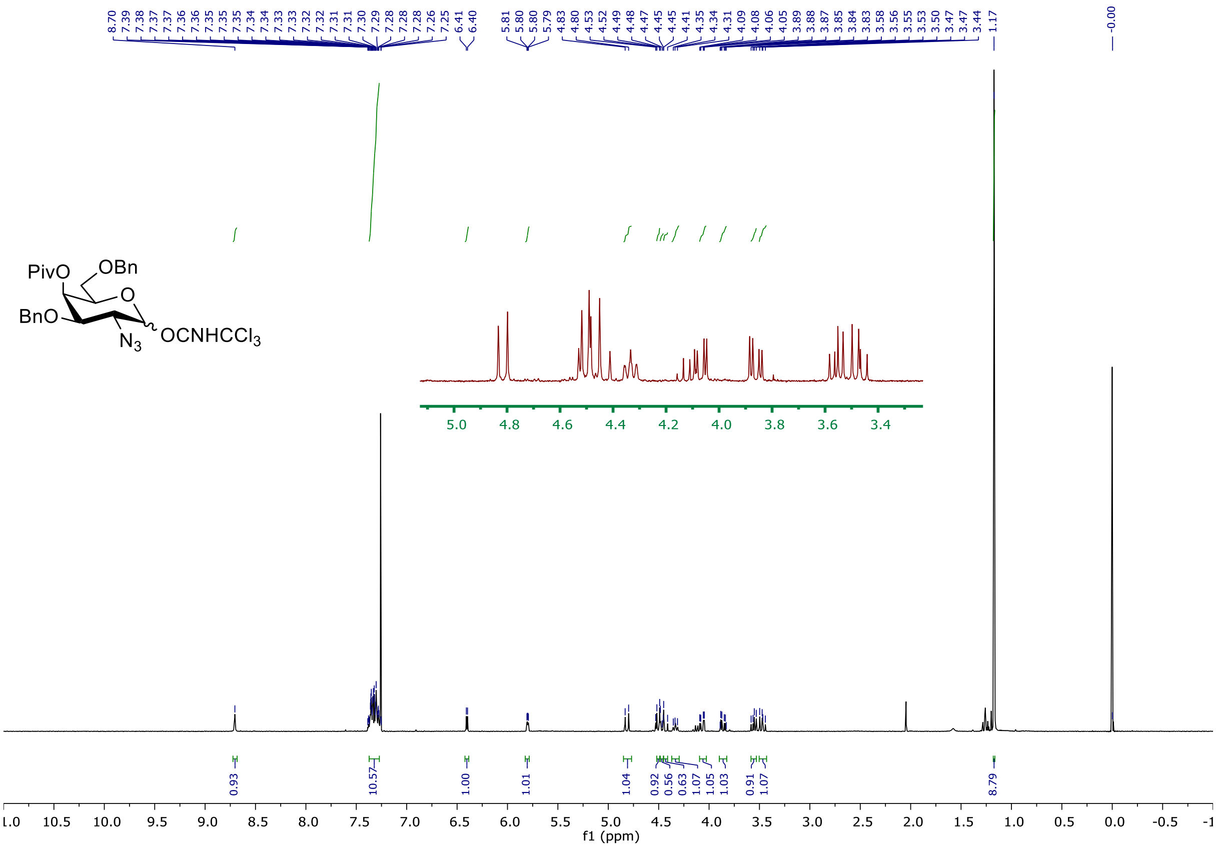

Figure S23. ^{1}H NMR ($CDCl_3$, 300 MHz) spectrum of **galactosyl donor 10**

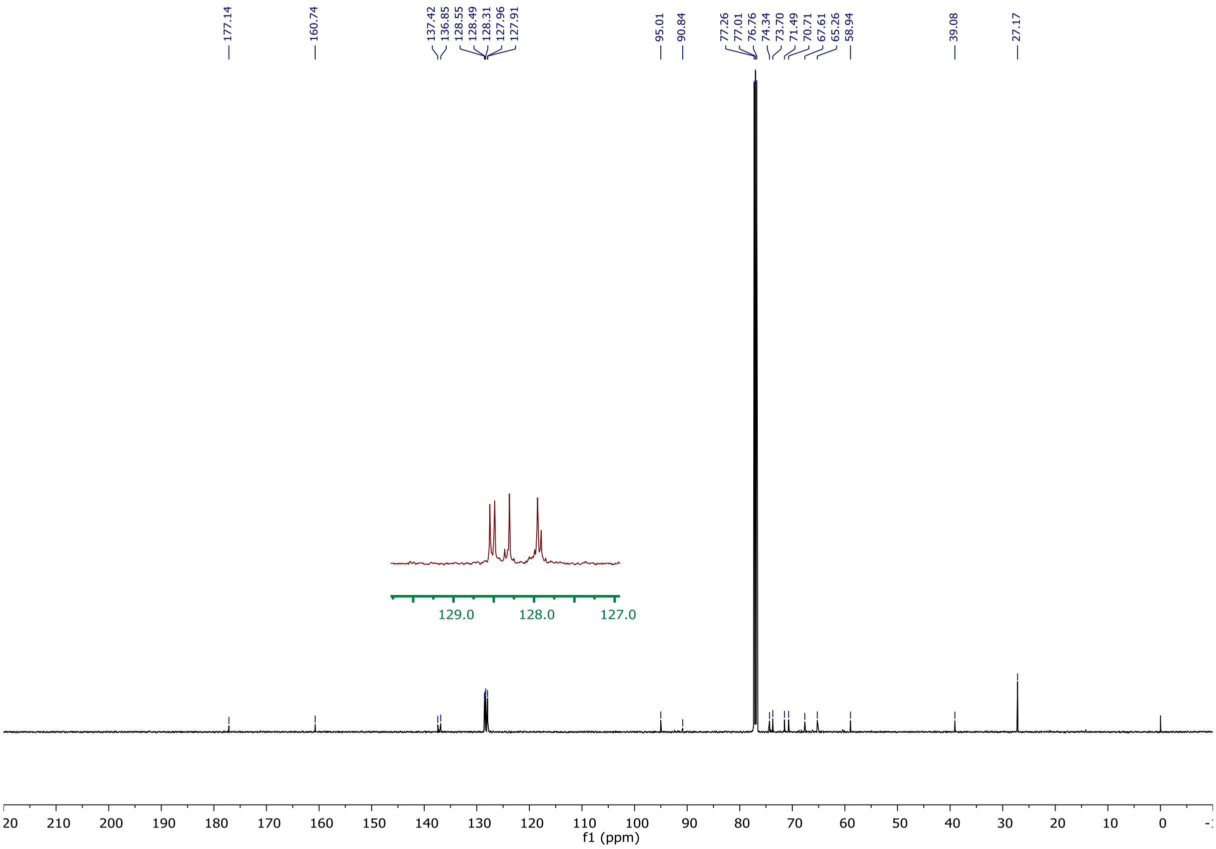

Figure S24. ^{13}C NMR ($CDCl_3$, 125 MHz) spectrum of **galactosyl donor 10**

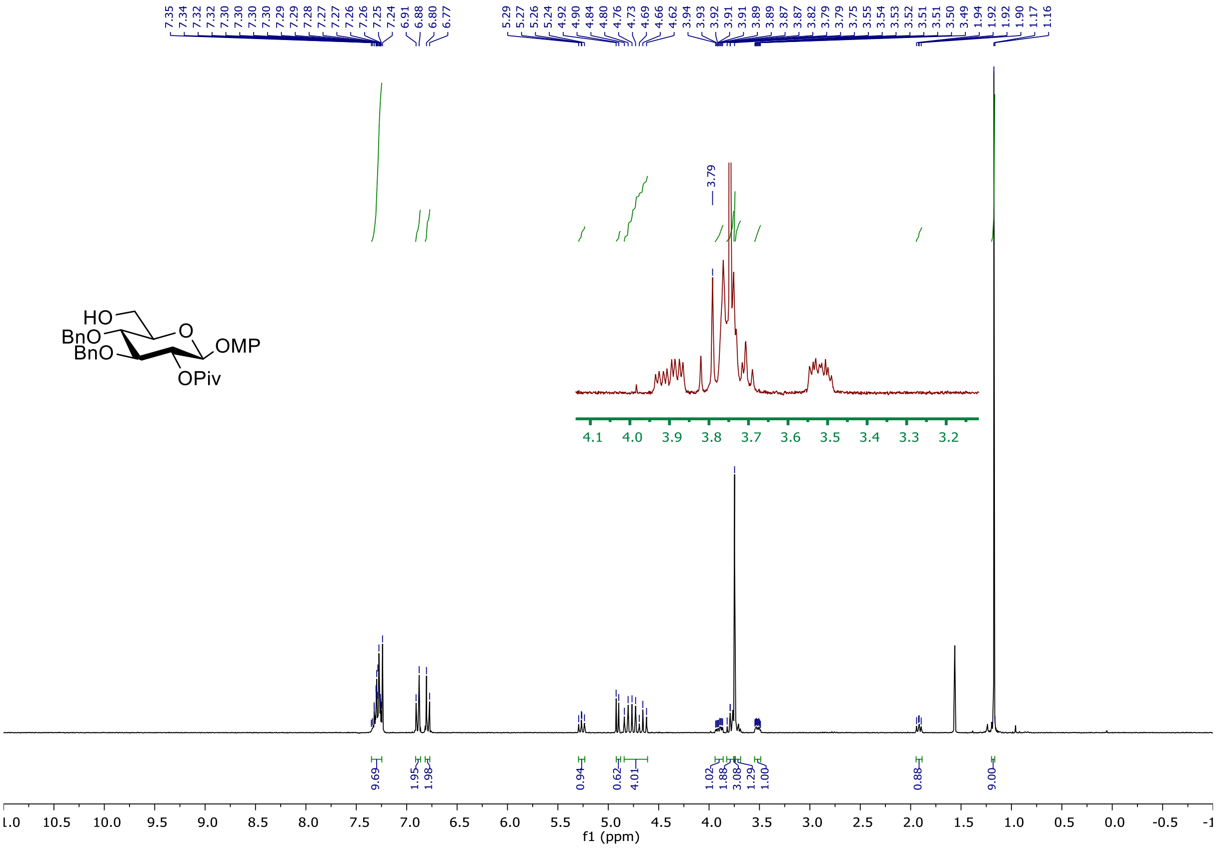

Figure S25. ^{1}H NMR ($CDCl_3$, 300 MHz) spectrum of **glucose building block 25**

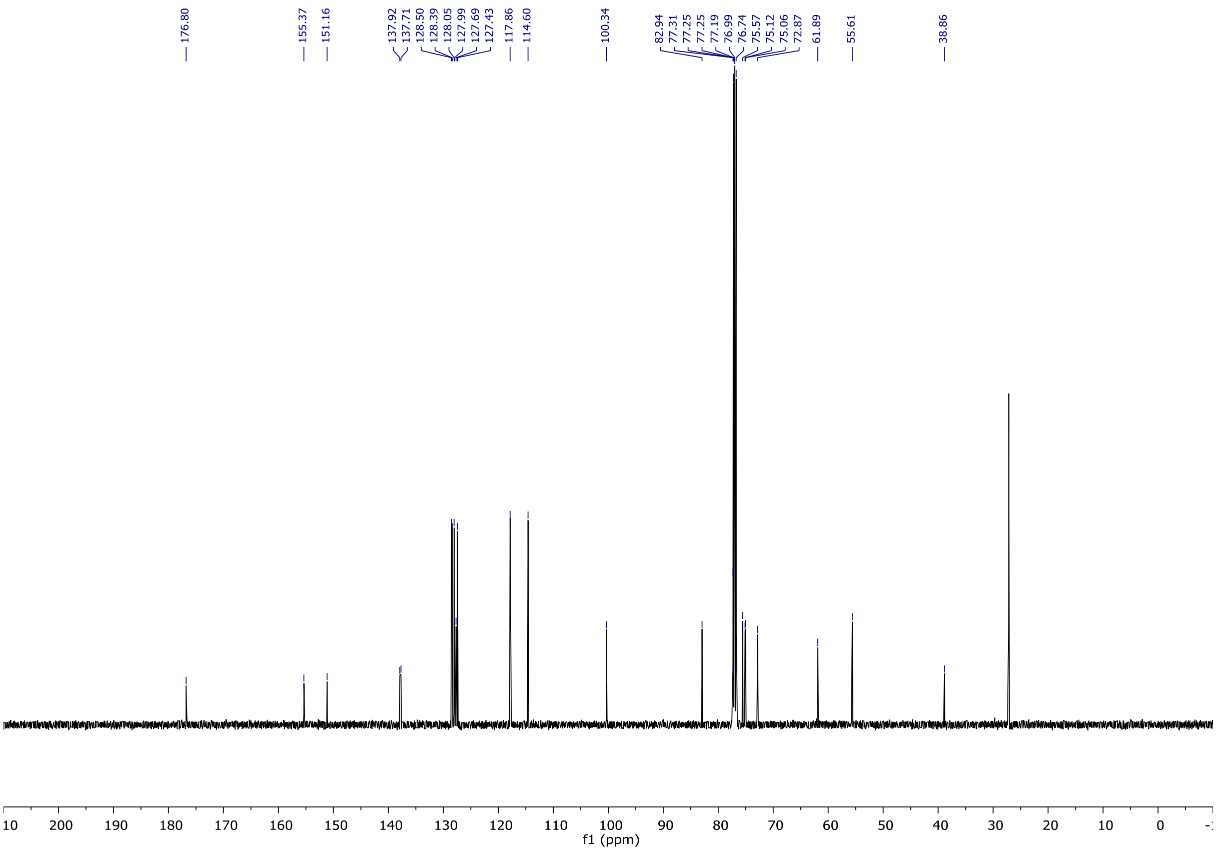

Figure S26. ^{13}C NMR ($CDCl_3$, 125 MHz) spectrum of **glucose building block 25**

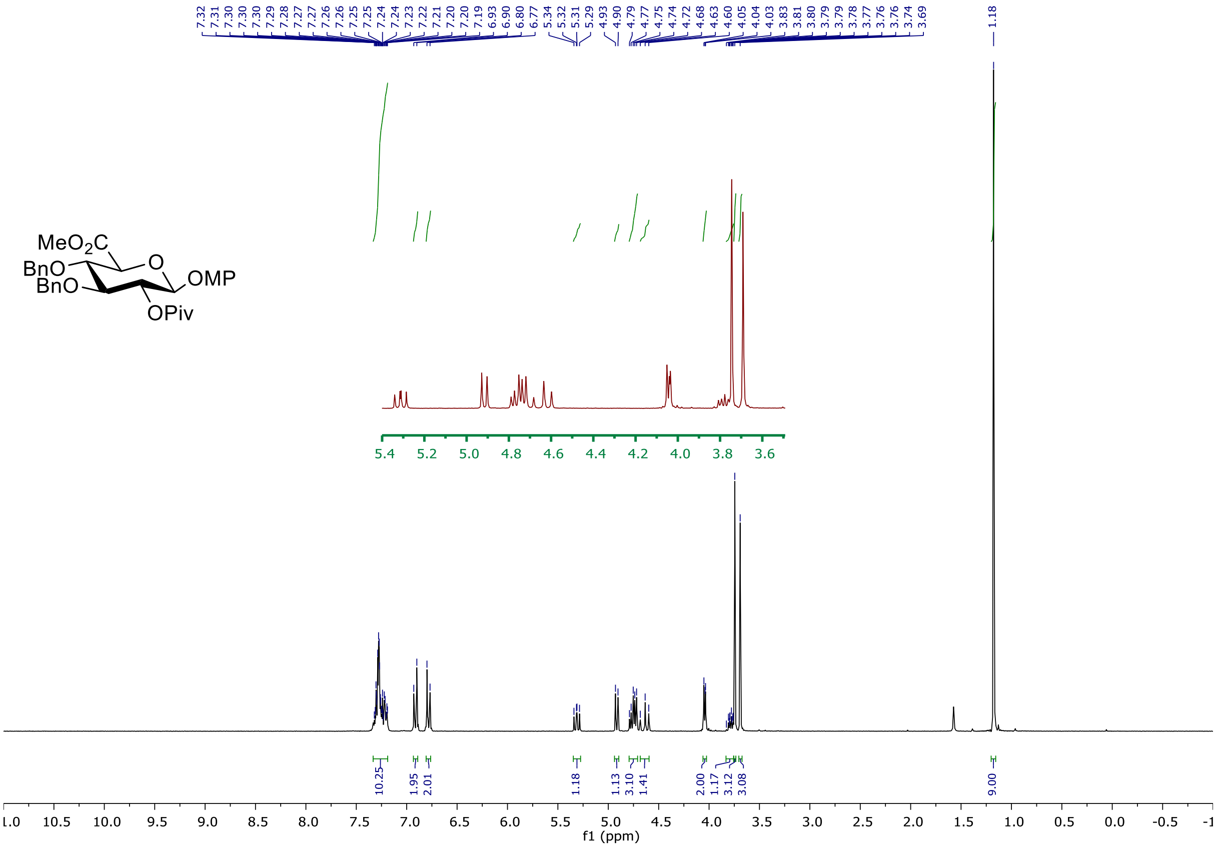

Figure S27. ^{1}H NMR ($CDCl_3$, 300 MHz) spectrum of **glucose building block 26**

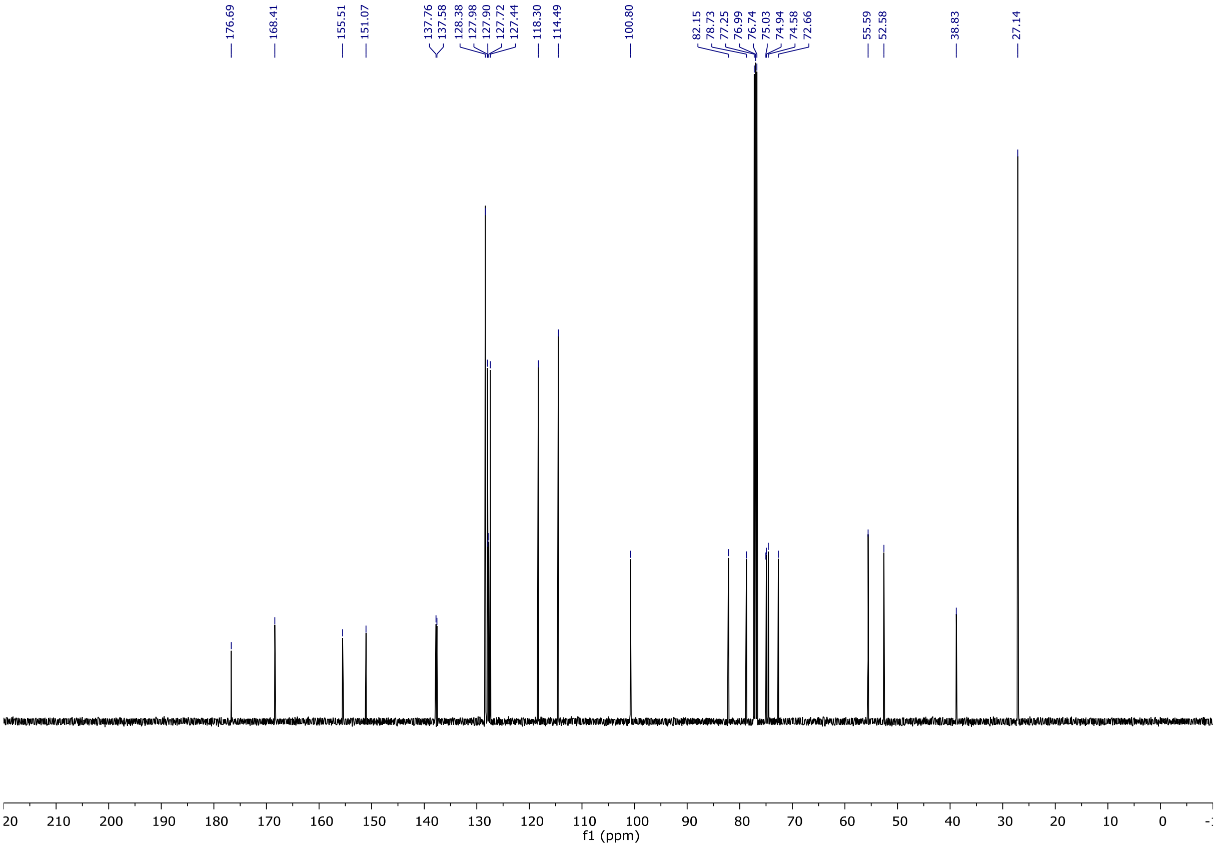

Figure S28. ^{13}C NMR ($CDCl_3$, 125 MHz) spectrum of **glucose building block 26**

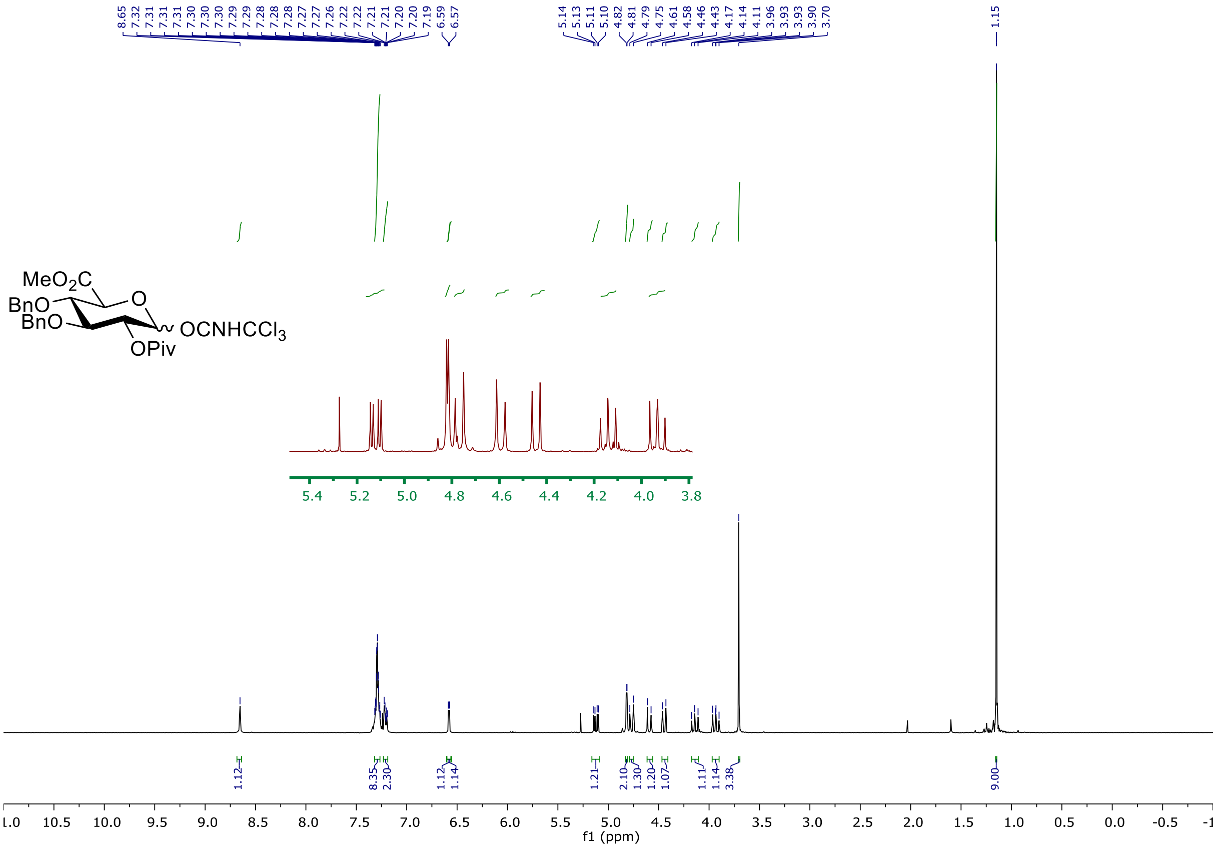

Figure S29. ^{1}H NMR ($CDCl_3$, 300 MHz) spectrum of **glucosyl donor 4**

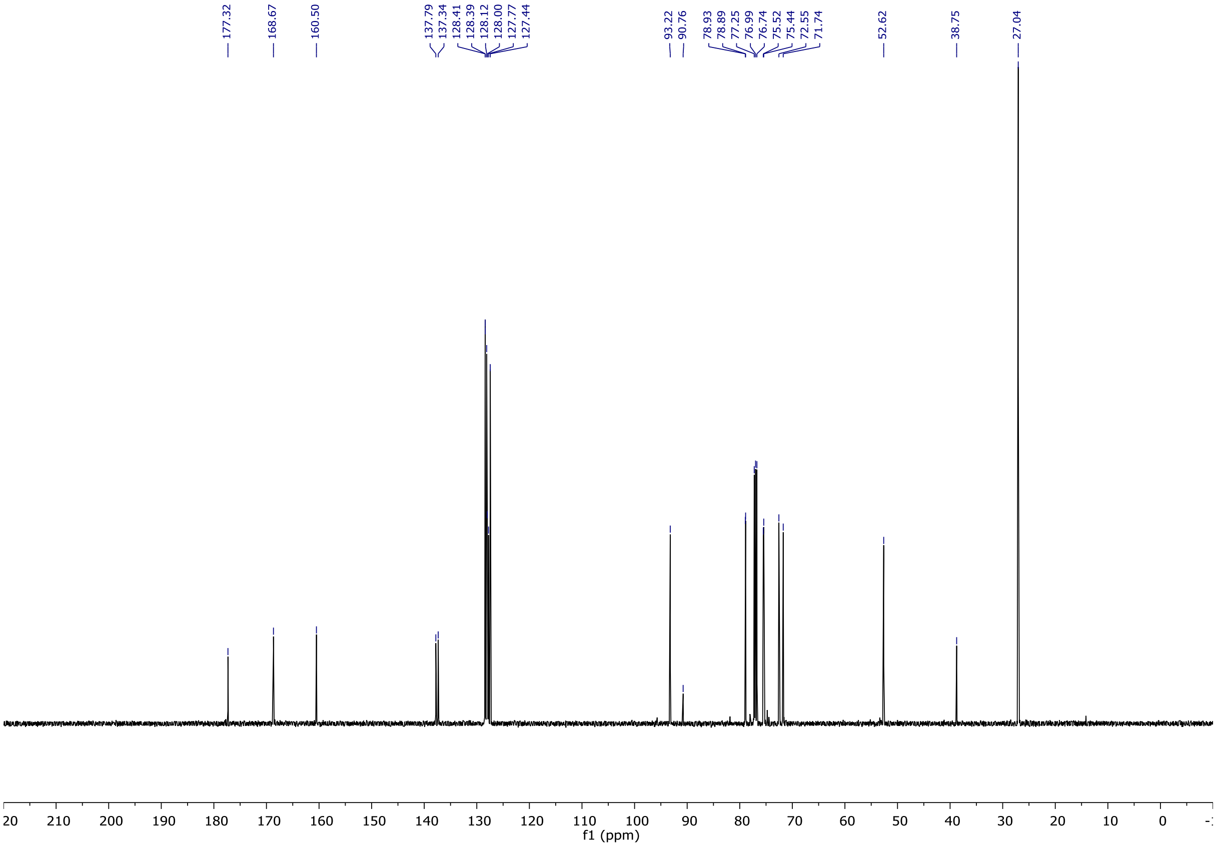

Figure S30. ^{13}C NMR ($CDCl_3$, 125 MHz) spectrum of **glucosyl donor 4**

5. Glycosylation reactions

5.1. Synthesis of the disaccharide acceptor 29.

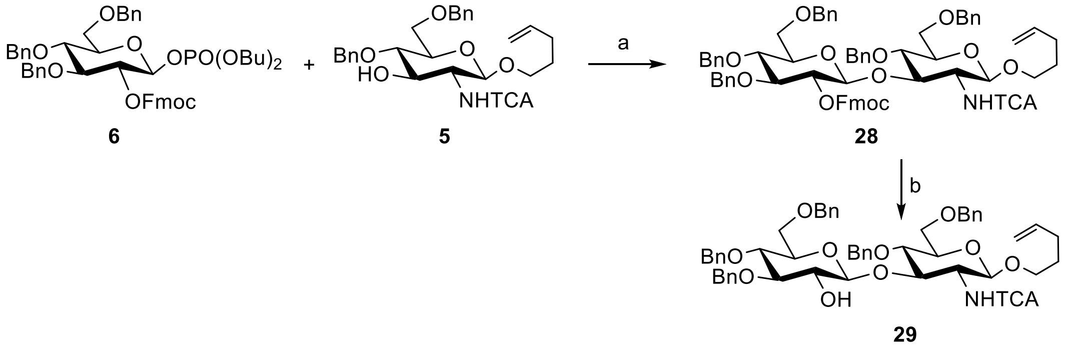

a) TMSOTf (1.1 eq), CH_2Cl_2, 1 h, -15 °C **b)** DMF/ piperidine (4:1), 1 h, 25 ºC.

5.2. Synthesis of the trisaccharide acceptor 31.

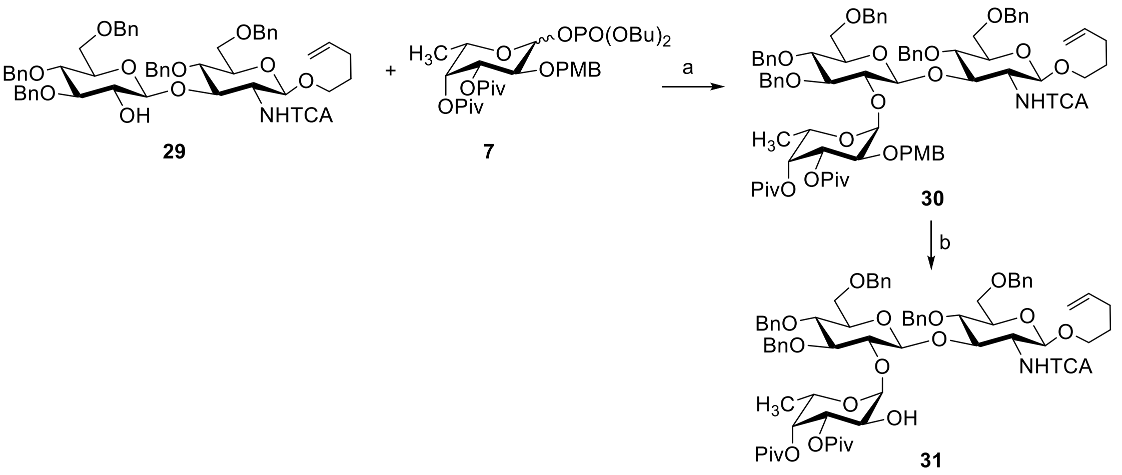

a) TMSOTf (1.8 eq), CH_2Cl_2, 2 h, -75 °C **b)** CAN, MeCN / H_2O (1:10), 25 ºC.

5.3. Synthesis of the tetrasaccharide acceptor 32.

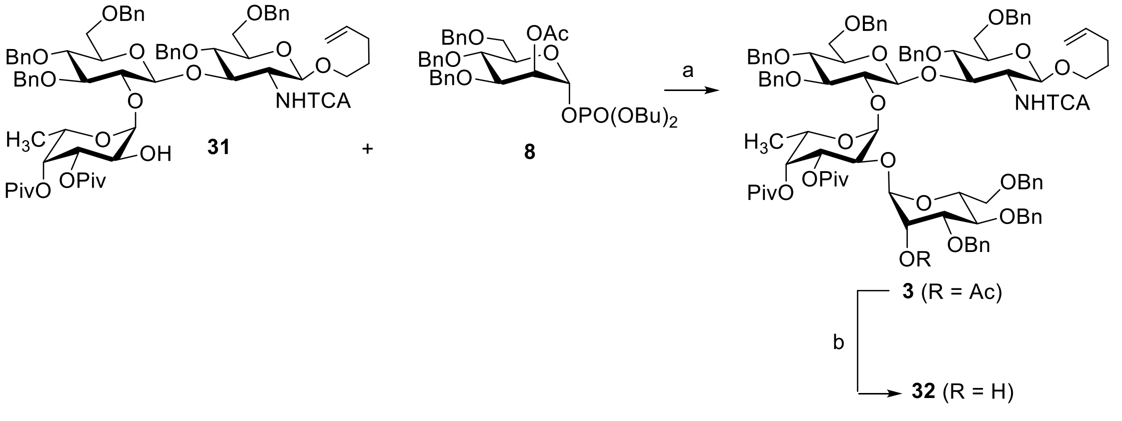

a) TMSOTf (4 eq), CH_2Cl_2, 3 h, 0 °C **b)** NaOMe (0.7 M), MeOH, 24 h, 25 ºC.

5.4. *Synthesis of the disaccharide donor 2.*

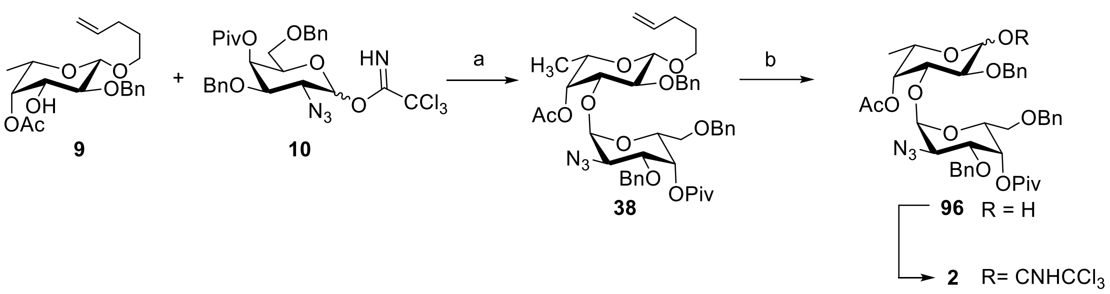

a) TMSOTf (0.15 eq), Et_2O, 90 min, -30 °C **b)** NBS, MeCN / H_2O (10:1), 3 h, 25 ºC **c)** Cl_3CCN, DBU, 0 °C → 25 ºC.

5.5. *Synthesis of the hexasaccharide acceptor 48*

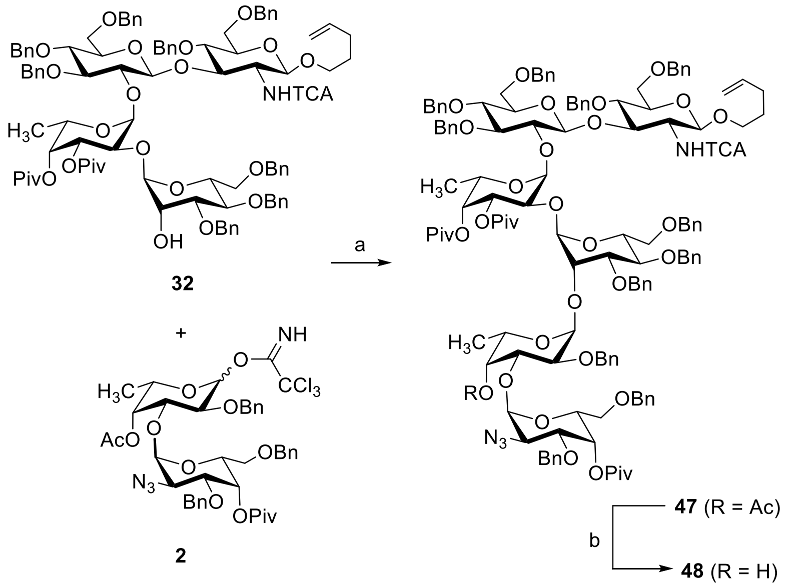

a) TMSOTf (0.15 eq), CH_2Cl_2, 3h, 0 °C **b)** NaOMe (1 M) in MeOH: CH_2Cl_2 (4:1)

5.6. *Synthesis of the hexasaccharide 52*

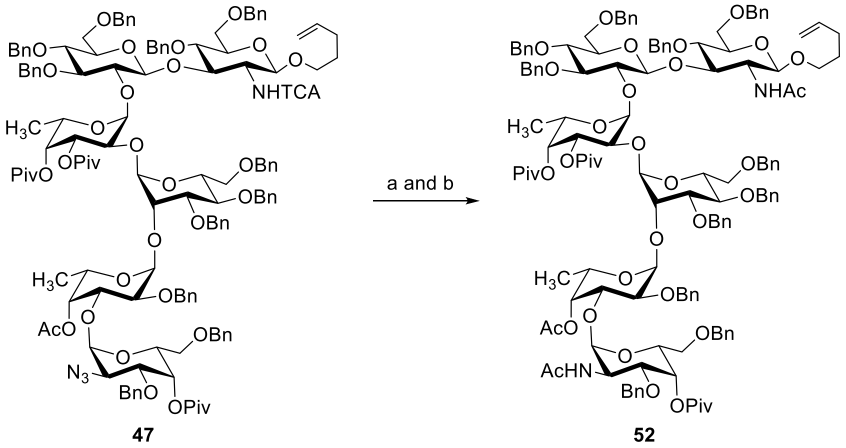

a) Zn / Cu Couple, AcOH, 2 d, 45 °C **b)** Ac_2O, Py, DMAP, Overnight, 25 ºC.

5.7. *Synthesis of the heptasaccharide 49*

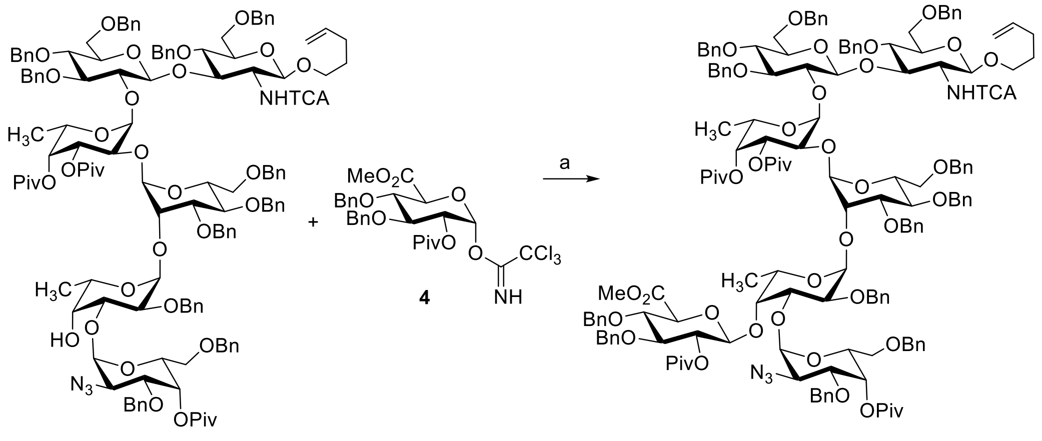

48 **49**

a) A diluted soln. TESOTf (0.15 eq) in CH_2Cl_2, CH_2Cl_2, 1 d, 0 °C

5.8. *Synthesis of the heptasaccharide 97*

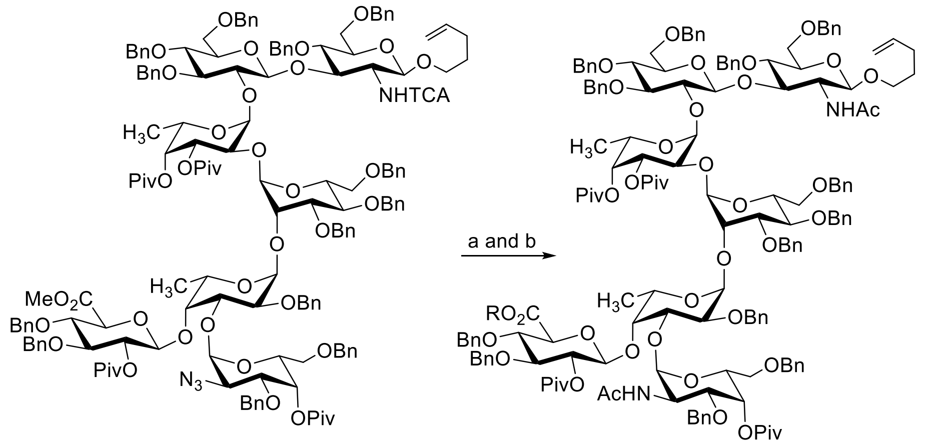

49

54 (R = Me)

97 (R = H)

a) Zn/ Cu Couple, AcOH, 2d, 45 °C **b)** Ac_2O, Py, DMAP, Overnight, 25 ºC **c)** KOH (3 M), MeOH.

6. Spectroscopic data of synthesized final compounds in glycosylation reactions

6.1. Synthesis of the disaccharide acceptor 28 and 29.

***n*-Pentenyl (3,4,6-tri-*O*-benzyl-2-*O*-fluorenylmethoxycarbonyl-β-D-glucopyranosyl)-(1→3)-4,6-di-*O*-benzyl-2-deoxy-2-trichloroacetamido-β-D-glucopyranoside (28)**

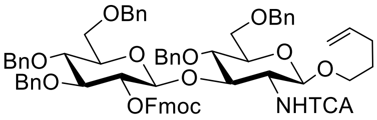

Compound **5** (0.36 g, 0.62 mmol) and compound **6** (0.59 g, 0.69 mmol) were mixed, co-evaporated with toluene (3x), dried in vacuum for 1 h, dissolved in CH_2Cl_2 (12 mL) under argon and cooled to – 15 °C. TMSOTf (0.12 mL, 0.69 mmol) was added and the reaction mixture was stirred for 1 h. After the completion of the reaction pyridine was added to quench the reaction and evaporated to dryness. The resulting crude product was purified by silica gel column chromatography (pentane / EtOAc, 8:1 → 4:1) to furnish 678 mg (88%) of disaccharide **28** as a colorless oil.

$[\boldsymbol{\alpha}]_{\boldsymbol{D}}^{\mathbf{20}}$= + 19.1° (c = 1.15, $CHCl_3$).

^{1}H NMR (300 MHz, $CDCl_3$): δ (ppm) 1.63-1.74 (m, 2 H), 2.12 (m_c, 2 H), 3.02 (q, *J* = 8.0 Hz, 1 H), 3.34–3.78 (m, 10 H), 3.91 (m_c, 2 H), 4.16 (t, *J* = 6.5 Hz, 1 H), 4.24–4.44 (m, 4 H), 4.45 (d, *J* = 8.1 Hz, 1 H, H – 1B), 4.54–4.77 (m, 8 H), 4.93–4.98 (m, 2 H), 5.02 (d, *J* = 7.8 Hz, 1 H, H – 1A), 5.08 (d, *J* = 11.1 Hz, 1 H), 5.78 (m_c, 1 H), 7.02 (d, *J* = 7.1 Hz, 1 H), 7.09–7.34 (m, 29 H), 7.54–7.61 (m, 2 H), 7.74-7.78 (m, 2 H).

^{13}C NMR (125 MHz, $CDCl_3$): δ (ppm) 28.9, 30.2, 47.1, 59.9, 68.6, 69.0, 69.3, 69.4, 73.3, 73.4, 74.5, 74.6, 74.9, 75.1, 75.2, 76.4, 77.3, 77.7, 77.9, 79.4, 82.1, 92.1, 98.1 (C – 1A), 100.9 (C – 1B), 114.9, 120.2, 120.3, 124.6, 124.7, 127.2, 127.2, 127.3, 127.4, 127.5, 127.5, 127.6, 127.6, 127.7, 127.7, 127.7, 127.7, 127.7, 127.8, 127.8, 127.9, 127.9, 128.0, 128.0, 128.0, 128.1, 128.1, 128.2, 128.2, 128.3, 128.3, 137.7, 137.7, 137.9, 137.9, 138.1, 138.5, 141.2, 141.2, 143.0, 143.0, 154.3, 161.5.

IR (ATR): $\tilde{\nu}$ (cm^{-1}) 3064, 3031, 2920, 2870, 1756, 1714, 1640, 1606, 1586, 1524, 1496.

HR-MS (ESI): m/z calcd. for $C_{69}H_{70}NO_{13}Na^+$ 1251.3811, found 1251.3831.

***n*-Pentenyl(3,4,6-tri-*O*-benzyl-β-D-glucopyranosyl)-(1→3)-4,6-di-*O*-benzyl-2-deoxy-2-trichloroacetamido-β-D-glucopyranoside (29)**

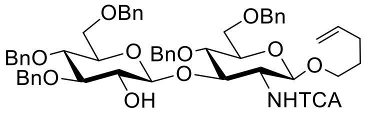

Compound **28** (0.25 mg, 0.20 mmol) was dissolved in DMF (4.0 mL), piperidine (1.0 mL) was added at room temperature and the reaction mixture was stirred for 1 h. After completion of the reaction, it was evaporated to dryness and the resulting crude product was purified by silica gel column chromatography (pentane / EtOAc, 6:1 → 2:1) to afford 160 mg (80%) of **29** as a colorless oil.

$[\boldsymbol{\alpha}]_{\boldsymbol{D}}^{\mathbf{20}}$= + 4.0° (c = 1.0, $CHCl_3$).

^{1}H NMR (300 MHz, $CDCl_3$): δ (ppm) 1.63 (m_c, 2 H), 2.04 (m_c, 2 H), 3.14 (s, 1 H), 3.43–3.89 (m, 13 H), 4.28 (t, *J* = 6.8 Hz, 1 H), 4.41 (d, *J* = 9.6 Hz, 1 H, H–1B), 4.45–4.57 (m, 6 H), 4.79 (d, *J* = 7.3 Hz, 1 H), 4.84 (d, *J* = 7.3

Hz, 1 H, H–1A), 4.88–4.97 (m, 3 H), 5.01–5.03 (m, 1 H), 5.73 (m_c, 1 H), 7.12–7.16 (m, 2 H), 7.22–7.37 (m, 23 H), 7.77 (d, *J* = 8 Hz, 1 H).

^{13}C NMR (120 MHz, $CDCl_3$): δ (ppm) 28.4, 29.8, 68.7, 69.1, 69.3, 73.2, 73.7, 73.8, 74.5, 74.8, 74.8, 75.1, 75.7, 76.8, 77.2, 84.2, 92.3, 98.8 (C-1A), 102.3 (C-1B), 114.9, 127.4, 127.5, 127.5, 127.6, 127.8, 127.8, 128.2, 128.2, 128.2, 128.3, 137.7, 137.8, 137.8, 137.9, 138.5, 161.7.

IR (ATR): $\tilde{\nu}$ (cm^{-1}) 3335, 3087, 3063, 3030, 2923, 2868, 1876, 1809, 1748, 1700, 1640.

HR-MS (ESI): m/z calcd. for $C_{54}H_{60}Cl_3NO_{11}Na^+$ 1028.3099, found 1028.3095.

6.2. Synthesis of the trisaccharide acceptor 31.

n-Pentenyl (2-*O*-4-methoxybenzyl-3,4-di-*O*-pivaloyl-α-L-fucopyranosyl)-(1→2)-(3,4,6- tri-*O*-benzyl-β-D-glucopyranosyl)-(1→3)-4,6-di-*O*-benzyl-2-deoxy-2-trichloroacetamido-β-D-glucopyranoside (30)

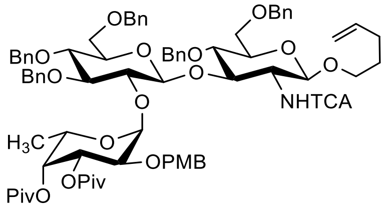

Compound **29** (80 mg, 79 µmol) and compound **7** (94 mg, 0.14 mmol) were mixed, co-evaporated with toluene (3x), dried in vacuum for 1 h, dissolved in CH_2Cl_2 (3.0 mL) under argon and cooled to - 75 °C. TMSOTf (25 µL, 0.14 mmol) was added and the reaction mixture was stirred for 2 h. After completion of the reaction pyridine was added and volatiles were removed. The resulting crude product was purified by silica gel column chromatography (pentane / EtOAc, 6:1 → 2:1) to obtain 121 mg (90%) of trisaccharide **30** as a colorless oil.

$[\alpha]_D^{20}$= – 16.7° (c = 0.49, $CHCl_3$).

^{1}H NMR (600 MHz, $CDCl_3$): δ (ppm) 1.09 (d, *J* = 6.7 Hz, 3H), 1.13 (s, 9 H), 1.23 (s, 9 H), 1.68 (m_c, 2 H), 2.17 (m_c, 2 H), 3.20 (m_c, 1 H), 3.41–3.71 (m, 5 H), 3.74 (s, 3 H), 3.76–3.82 (m, 4 H), 3.93 (dt, *J* = 9.1, 6.3 Hz, 1 H), 4.32–4.44 (m, 5 H), 4.47 (d, *J* = 11.7 Hz, 1 H), 4.50 (d, *J* = 12.5 Hz, 1 H), 4.53 (s, 3 H), 4.62 (d, *J* = 12.5 Hz, 1 H), 4.77 (d, *J* = 11.8 Hz, 1 H), 4.81–5.01 (m, 6 H), 5.09 (d, *J* = 11.0 Hz, 1 H), 5.22 (bs, 1 H), 5.56 (d, *J* = 3.1 Hz, 1 H), 5.60 (dd, *J* = 10.7, 2.8 Hz, 1 H), 5.78 (m_c, 1 H), 6.69 (d, *J* = 8.6 Hz, 2 H), 6.97– 7.03 (m, 2 H), 7.14–7.25 (m, 17 H), 7.28–7.31 (m, 8 H), 8.15 (d, *J* = 6.5 Hz, 1 H).

^{13}C NMR (150 MHz, $CDCl_3$): δ (ppm) 15.6, 27.1, 27.3, 28.9, 30.0, 38.9, 39.0, 55.2, 60.5, 65.5, 68.7, 69.1, 69.9, 69.9, 71.8, 72.3, 72.4, 73.3, 73.4, 73.7, 74.4, 74.6, 74.9, 74.9, 75.2, 76.6, 77.7, 78.3, 86.1, 92.5, 96.4 (C–1C), 97.9 (C–1A), 101.8 (C–1B), 113.6, 114.6, 126.4, 127.1, 127.4, 127.5, 127.5, 127.6, 127.7, 127.8, 128.0, 128.2, 128.2, 128.3, 128.3, 128.3, 128.3, 128.9, 129.7, 137.8, 138.2, 138.3, 138.4, 138.5, 138.7, 159.1, 162.9, 177.3, 178.5.

IR (ATR): $\tilde{\nu}$ (cm^{-1}) 3063, 3031, 2973, 2934, 2871, 1952, 1879, 1735, 1713, 1640, 1612.

HR-MS (ESI): m/z calcd. for $C_{78}H_{94}Cl_3NO_{18}Na^+$ 1462.5406, found 1462.5424.

n-Pentenyl(3,4-di-*O*-pivaloyl-α-L-fucopyranosyl)-(1→2)-(3,4,6-tri-*O*-benzyl-β-D-glucopyranosyl)-(1→3)-4,6-di-*O*-benzyl-2-deoxy-2-trichloroacetamido-β-D-glucopyranoside (31)

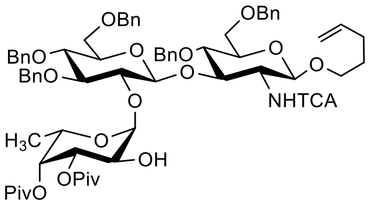

Compound **30** (115 mg, 80 µmol) was dissolved in CH_2Cl_2 (4.0 mL) and water (400 µL), CAN (131 mg, 240 µmol) was added and the reaction mixture was stirred for 1.5 h. The reaction mixture was poured into a saturated solution of $NaHCO_3$ and extracted with CH_2Cl_2 (3x). Afterwards the combined organic layers were dried over $MgSO_4$ and evaporated to dryness. The resulting crude product was purified by silica gel column chromatography (pentane / EtOAc, 6:1 → 2:1) to furnish 74 mg (70%) of **31** as a colorless oil.

$[\alpha]_D^{20}$= – 31.9° (c = 0.36, $CHCl_3$).

^{1}H NMR (300 MHz, $CDCl_3$): δ (ppm) 1.09 (s, 9 H), 1.12 (d, J = 6.5 Hz, 3 H), 1.27 (s, 9 H), 1.64 (m_c, 2 H), 2.11 (m_c, 2 H), 2.70 (d, J = 12.0 Hz, 1 H), 3.36–3.95 (m, 15 H), 4.32 (d, J = 11.0 Hz, 1 H), 4.47–4.67 (m, 7 H), 4.71 (dd, J = 10.0, 7.2 Hz, 1 H), 4.85 (d, J = 11.3 Hz, 1 H), 4.90–5.01 (m, 4 H), 5.08 (d, J = 11.0 Hz, 1 H), 5.24 (s_{br}, 2 H), 5.44 (d, J = 3.6 Hz, 1 H), 5.75 (m_c, 1 H), 7.01–7.05 (m, 2 H), 7.19–7.32 (m, 23 H), 7.65 (d, J = 6.8 Hz, 1 H).

^{13}C NMR (125 MHz, $CDCl_3$): δ (ppm) 16.0, 27.0, 27.3, 28.9, 30.1, 38.8, 39.1, 60.1, 66.8, 68.0, 68.5, 68.9, 69.1, 70.6, 71.0, 73.3, 73.4, 74.5, 74.6, 74.8, 75.0, 77.8, 78.6, 83.8, 92.6, 98.1, 99.3, 101.9, 114.7, 127.2, 127.4, 127.4, 127.5, 127.7, 127.7, 127.8, 127.9, 128.2, 128.2, 128.2, 128.3, 128.3, 137.4, 137.5, 137.9, 137.9, 138.0, 138.3, 161.7, 177.2, 178.3.

IR (ATR): $\tilde{\nu}$ (cm^{-1}) 3412, 3064, 3031, 2972, 2933, 2871, 2305, 1952, 1732, 1716, 1641.

HR-MS (ESI): m/z calcd. for $C_{70}H_{86}Cl_3NO_{17}Na^+$ 1342.4830, found 1342.4881.

6.3. Synthesis of the tetrasaccharide acceptor 32.

_n_-Pentenyl(2-_O_-acetyl-3,4,6-tri-_O_-benzyl-α-D-mannopyranosyl)-(1→2)-(3,4-di-_O_-pivaloyl-α-L-fucopyranosyl)-(1→2)-(3,4,6-tri-_O_-benzyl-β-D-glucopyranosyl)-(1→3)-4,6-di-_O_-benzyl-2-deoxy-2-trichloroacetamido-β-D-glucopyranoside (3)

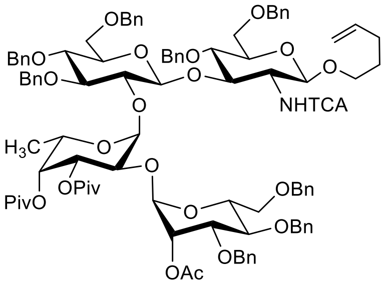

Compound **31** (85 mg, 64 µmol) and compound **8** (176 g, 257 µmol) were mixed, co-evaporated with toluene (3x), dried in vacuum for 1 h, dissolved in CH_2Cl_2 (2.00 mL) under argon and cooled to 0 °C. TMSOTf (46.0 µL, 257 µmol) was added and the reaction mixture was stirred for 2 h. After the completion of the reaction pyridine was added to quench the reaction and volatiles were removed. The resulting crude product was purified by HPLC (gel permeation chromatography) to furnish 98 mg (85%) of tetrasaccharide **3** as a colorless oil.

$[\alpha]_D^{20}$= – 4.6° (c = 0.70, $CHCl_3$).

^{1}H NMR (300 MHz, $CDCl_3$): δ (ppm) 1.12 (s, 9 H), 1.16 (d, *J* = 7.2 Hz, 3 H), 1.27 (s, 9 H), 1.70 (m_c, 2 H), 2.10 (s, 3 H), 2.19 (m_c, 2 H), 2.90–3.97 (m, 20 H), 4.16 (dd, J = 16.3, 11.4 Hz, 2 H), 4.25 (dd, *J* = 10.7, 2.9 Hz, 1 H), 4.43 (d, *J* = 8.6 Hz, 1 H, H-1B), 4.45–4.54 (m, 7 H), 4.65 (d, *J* = 12.5 Hz, 1 H), 4.75 (dd, *J* = 16.0, 10.8 Hz, 2 H),4.82 (d, *J* = 12.3 Hz, 1 H), 4.90-5.06 (m, 5 H), 5.09 (d, *J* = 11.2 Hz, 1 H), 5.25 (s_{br}, 1 H), 5.44 (s_{br}, 1 H), 5.69 (dd, *J* = 10.8, 2.9 Hz, 1 H, H-1C), 5.79 (m_c, 1 H), 6.89-7.36 (m, 40 H), 8.25 (d, *J* = 6.4 Hz, 1 H).

^{13}C NMR (75 MHz, $CDCl_3$): δ (ppm) 15.6, 20.9, 26.9, 27.4, 27.4, 29.0, 30.0, 38.9, 39.1, 60.5, 65.6, 67.5, 67.6, 68.7, 69.0, 70.1, 70.5, 71.4, 71.8, 71.8, 73.2, 73.3, 73.4, 73.5, 73.6, 74.6, 74.8, 74.9, 75.2, 76.1, 77.9, 78.3, 78.9, 85.4, 92.7, 96.8 (C–1C), 97.9 (C–1A), 98.8 (C–1D), 101.4 (C–1B), 114.6, 125.9, 126.8, 127.4, 127.5, 127.6, 127.6, 127.7, 127.7, 127.8, 127.9, 128.0, 128.1, 128.1, 128.1, 128.2, 128.2, 128.2, 128.2, 128.3, 128.3, 128.3, 128.4, 137.6, 137.9, 138.0, 138.1, 138.2, 138.2, 138.3, 138.5, 138.5, 162.6, 169.7, 177.4, 179.1.

IR (ATR): $\tilde{\nu}$ (cm^{-1}) 3088, 3031, 2958, 2932, 2871, 2361, 1952, 1872, 1737, 1714, 1640.

HR-MS (ESI): m/z calcd. for $C_{99}H_{116}Cl_3NO_{23}Na^+$ 1816.6874, found 1816.6886.

n-Pentenyl(3,4,6-tri-*O*-benzyl-α-D-mannopyranosyl)-(1→2)-(3,4-di-*O*-pivaloyl-α-L-fucopyranosyl)-(1→2)-(3,4,6-tri-*O*-benzyl-β-D-glucopyranosyl)-(1→3)-4,6-di-*O*-benzyl-2-deoxy-2-trichloroacetamido-β-D-glucopyranoside (32)

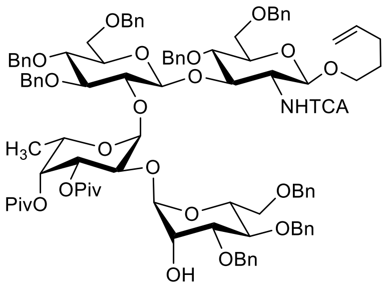

Compound **3** (124 mg, 69.0 μmol) in CH_3OH (1.00 mL), and a methanolic solution of NaOMe (1 M, 6.90 μL, 6.91 μmol) was added at room temperature. After 16 h, the reaction mixture was neutralized with Amberlite acidic resin (IR-120) filtered through a sintered glass funnel and concentrated *in* vacuum. The resulting crude product was purified by silica gel column chromatography (pentane / EtOAc, 7:1 → 4:1) afforded 94 mg (78%) of **32** as a colorless oil.

$[\alpha]_D^{23}$= + 7.5° (c = 0.25, $CHCl_3$).

^{1}H NMR (600 MHz, $CDCl_3$): δ (ppm) 1.14 (d, *J* = 6.0 Hz, 1 H), 1.16 (s, 9 H), 1.26 (s, 9 H), 1.67 (m_c, 2 H), 2.18 (m_c, 2 H), 2.35 (s, 1 H), 2.90 (dd, *J* = 10.8, 3.4 Hz, 1 H), 2.99 (dd, *J* = 10.9, 1.9 Hz, 1 H), 3.17-3.21 (m, 1 H), 3.36–3.51 (m, 6 H), 3.62 (t, *J* = 9.0 Hz, 1 H), 3.66–3.69 (m, 3 H), 3.76–3.82 (m, 3 H), 3.85 (dd, *J* = 8.9, 7.9 Hz, 1 H), 3.93– 3.95 (m, 2 H), 4.16 (d, *J* = 12.2 Hz, 1 H), 4.24-4.27 (m, 2 H), 4.38–4.46 (m, 6 H), 4.49 (d, *J* = 7.8 Hz, 1 H, H-1B), 4.51 (d, *J* = 3.1 Hz, 1 H), 4.66-4.72 (m, 4 H), 4.83 (d, *J* = 12.5 Hz, 1 H), 4.90-4.94 (m, 2 H), 4.97 (d, *J* = 8.1 Hz, 1 H, H-1A), 4.99-5.02 (m, 2 H), 5.05 (d, *J* = 1.6 Hz, 1 H, H–1D), 5.08 (d, *J* = 11.2 Hz, 1 H), 5.23–5.24 (m, 1 H), 5.58 (d, *J* = 3.1 Hz, 1 H, H–1C), 5.68 (dd, *J* = 10.8, 2.8 Hz, 1 H), 5.79 (m_c, 1 H), 6.93-6.99 (m, 4 H), 7.01–7.08 (m, 4 H), 7.11-7.23 (m, 20 H), 7.25-7.33 (m, 10 H), 7.36-7.38 (m, 2 H), 8.24 (d, *J* = 6.5 Hz, 1 H).

^{13}C NMR (150 MHz, $CDCl_3$): δ (ppm) 15.6, 27.1, 27.4, 29.1, 30.1, 38.9, 39.1, 60.5, 65.6, 67.6, 67.9, 68.7, 69.0, 69.1, 70.3, 70.5, 71.5, 72.1, 73.2, 73.2, 73.3, 73.4, 73.6, 73.7, 74.6, 74.8, 74.9, 75.0, 75.2, 76.0, 77.2, 77.9, 78.8, 80.1, 85.5, 92.7, 96.9 (C–1C), 97.9 (C–1A), 100.0 (C–1D), 101.4 (C–1B), 114.5, 125.9, 126.7, 127.3, 127.3, 127.3,

127.4, 127.4, 127.5, 127.6, 127.6, 127.7, 127.8, 127.9, 127.9, 127.9, 128.0, 128.1, 128.1, 128.1, 128.2, 128.2, 128.2, 128.4, 137.5, 137.8, 138.0, 138.1, 138.1, 138.2, 138.2, 138.4, 138.4, 162.4, 177.2, 178.8.

IR (ATR): $\tilde{\nu}$ (cm^{-1}) 3348, 3086, 3062, 3030, 2955, 2921, 2854, 1736, 1713, 1640, 1605.

HR-MS (ESI): m/z calcd. for $C_{97}H_{114}Cl_3NO_{22}Na^+$ 1776.6825, found 1776.6838.

6.4. Synthesis of the disaccharide donor 2.

_n_-Pentenyl(2-azido-3,6-di-_O_-benzyl-2-deoxy-4-_O_-pivaloyl-α-D-galactopyranosyl)-(1→3)-4-_O_-acetyl-2-_O_-benzyl-β-L-fucopyranoside (38)

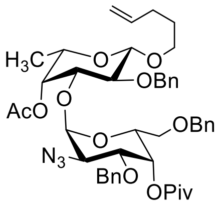

Compound **9** (300 mg, 0.82 mmol) and compound **10** (755 mg, 1.23 mmol) were mixed, co-evaporated with toluene (3x), dried *in* vacuum for 1 h and dissolved in Et_2O (15 mL). To the cooled solution at - 30 °C, TMSOTf (22 μL, 0.12 mmol) was added and reaction mixture was stirred for 90 min. After completion of the reaction pyridine was added and the reaction mixture was evaporated to dryness. The resulting crude product was purified by silica gel column chromatography (pentane / EtOAc, 9:1 → 2:1) to furnish 595 mg (87%) of disaccharide **38** as a colorless oil.

$[\boldsymbol{\alpha}]_{\mathbf{D}}^{\mathbf{20}}$= + 83.5° (c = 0.63, $CHCl_3$).

^{1}H NMR (400 MHz, $CDCl_3$): δ (ppm) 1.10 (d, J = 6.4 Hz, 3 H), 1.16 (s, 9 H), 1.75 (m_c, 2 H), 2.04 (s, 3 H), 2.14 (m_c, 2 H), 3.32 (dd, J = 10.8, 3.7 Hz, 1 H), 3.43-3.53 (m, 4 H), 3.67 (dd, J = 9.8, 7.8 Hz, 1 H), 3.81-3.87 (m, 2 H), 3.95 (dt, J = 9.5, 6.5 Hz, 1 H), 4.30 (d, J = 7.8 Hz, 1 H, H–1A), 4.44 (d, J = 11.2 Hz, 1 H), 4.48 (t, J = 5.9 Hz, 1 H), 4.53 (d, J = 2.8 Hz, 1 H), 4.69 (d, J = 10.3 Hz, 1 H), 4.73 (d, J = 11.2 Hz, 1 H), 4.94-5.02 (m, 3 H), 5.07 (dd, J = 3.5, 1.0 Hz, 1 H, H–1B), 5.43 (d, J = 3.7 Hz, 1 H), 5.43 (d, J = 3.7 Hz, 1 H), 5.63 (d, J = 2.0 Hz, 1 H), 5.81 (m_c, 1 H), 7.26-7.40 (m, 15 H).

^{13}C NMR (100 MHz, $CDCl_3$): δ (ppm) 16.0, 20.8, 27.2, 28.9, 30.2, 39.1, 58.9, 66.6, 68.8, 68.9, 69.4, 69.6, 71.0, 72.4, 72.4, 73.2, 74.6, 74.9, 77.2, 79.5, 99.3 (C–1B), 103.8 (C–1A), 114.9, 127.3, 127.6, 127.9, 128.2, 128.3, 128.3, 128.4, 128.4, 137.2, 137.9, 138.2, 138.3, 171.1, 177.5.

IR (ATR): $\tilde{\nu}$ (cm^{-1}) 3064, 3031, 2972, 2934, 2870, 2108, 1735, 1640, 1496, 1478, 1455.

HR-MS (ESI): m/z calcd. for $C_{45}H_{57}N_3O_{11}Na^+$ 838.3885, found 838.3906.

2-Azido-3,6-di-_O_-benzyl-2-deoxy-4-_O_-pivaloyl-α-D-galactopyranosyl-(1→3)-4-_O_-acetyl-2-_O_-benzyl-L-fucopyranosyl trichloroacetimidate (2)

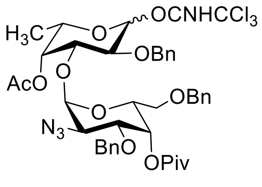

Compound **38** (590 mg, 0.720 mmol) was dissolved in CH_3CN (7.00 mL) and H_2O (70.0 μL). Then NBS (167 mg, 0.940 mmol) was added at room temperature. Purification by silica gel column chromatography (pentane/ EtOAc, 3:1 → 1:1) gave 413 mg (77%) of the hemiacetal as a pale yellow oil. Further, it was dissolved in CH_2Cl_2 (7.00 mL) and Cl_3CCN (1.10 mL, 11.0 mmol) and DBU (12.0 μL, 82.0 μmol) were added. Purification by silica gel column chromatography (pentane / EtOAc, 7:1 → 4:1) furnished 442 mg (90%) of **2** (α / β 6:1) as a pale yellow oil.

The analytical data is for the α anomer.

$[\alpha]_D^{25}$= + 103.4 (c = 0.25, $CHCl_3$).

^{1}H NMR (500 MHz, $CDCl_3$): δ (ppm) 1.06 (d, J = 6.5 Hz, 3 H), 1.16 (s, 9 H), 2.04 (s, 3 H), 3.32 (dd, J = 10.8, 3.6 Hz, 1 H), 3.52-3.59 (m, 3 H), 3.82 (dd, J = 10.8, 3.1 Hz, 1 H), 4.05 (dd, J = 10.1, 3.5 Hz, 1 H), 4.30 (dd, J = 10.0, 3.4 Hz, 1 H), 4.44-4.58 (m, 5 H), 4.67 (d, J = 1.8 Hz, 1 H), 4.74 (d, J = 11.3 Hz, 1 H), 5.23 (dd, J = 3.5, 1.3 Hz, 1 H), 5.44 (d, J = 3.7 Hz, 1 H, H-1B), 5.65 (d, J = 2.4 Hz, 1 H), 6.48 (d, J = 3.5 Hz, 1 H, H-1A), 7.29–7.40 (m, 15 H), 8.55 (s, 1 H).

^{13}C NMR (125 MHz, $CDCl_3$): δ (ppm) 15.9, 20.8, 27.2, 39.1, 58.9, 66.5, 67.6, 69.0, 69.3, 70.9, 71.4, 72.4, 72.8, 73.2, 76.1, 91.1, 94.1 (C-1A), 99.3 (C-1B), 127.2, 127.3, 127.5, 127.7, 127.8, 127.8, 127.9, 128.2, 128.2, 128.3, 128.3, 128.3, 137.1, 137.5, 138.0, 161.1, 170.7, 177.3.

IR (ATR): $\tilde{\nu}$ (cm^{-1}) 3335, 3063, 3031, 2972, 2933, 2907, 2870, 2933, 2907, 2106, 1733.

HR-MS (ESI): m/z calcd. for $C_{42}H_{49}Cl_3N_4O_{11}Na^+$ 913.2356, found 913.2358.

6.5. Synthesis of the hexasaccharide acceptor 52

_n_-Pentenyl (2-azido-3,6-di-_O_-benzyl-2-deoxy-4-_O_-pivaloyl-α-D-galactopyranosyl)-(1→3)-(4-_O_-acetyl-2-_O_-benzyl-α-L-fucopyranosyl)-(1→2)-(3,4,6-tri-_O_-benzyl-α-D-manno-pyranosyl)-(1→2)-(3,4-di-_O_-pivaloyl-β-L-fucopyranosyl)-(1→2)-(3,4,6-tri-_O_-benzyl-β-D-glucopyranosyl)-(1→3)-4,6-di-_O_-benzyl-2-deoxy-2-trichloroacetamido-β-D-glucopyranoside (47)

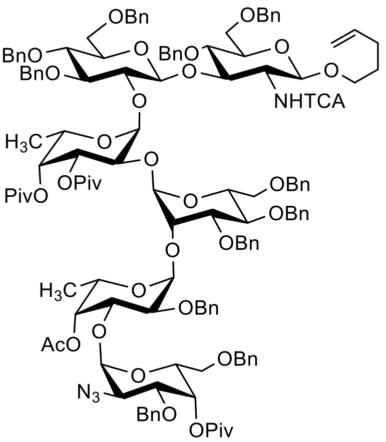

Compound **32** (94.0 mg, 53.0 μmol) and compound **2** (191 mg, 214 μmol) were mixed, co-evaporated with toluene (3x), dried in vacuum for 1 h and dissolved in dichloromethane (2.0 mL). TMSOTf (1.50 μL, 8.00 μmol) was added at 0 °C and the reaction mixture was stirred for 3 h. Afterwards it was quenched by the addition of pyridine and evaporated to dryness. The resulting crude product was purified by HPLC (gel permeation chromatography) to obtain 89 mg (67% isolated, 91% brsm) of hexasaccharide **47** as a colorless oil.

$[\alpha]_D^{24}$= + 24.5° (c = 0.11, $CHCl_3$).

^{1}H NMR (600 MHz, $CDCl_3$): δ = 0.78 (d, J = 6.7 Hz, 3 H), 1.13 (s, 9 H), 1.15 (s, 9 H), 1.17 (d, J = 6.5 Hz, 3 H), 1.19 (s, 9 H), 1.68 (m_c, 2 H), 2.00 (s, 3 H), 2.17 (m_c, 2 H), 3.14 (dd, J = 11.4, 4.5 Hz, 1 H), 3.17 (s, 1H), 3.20 (dd, J = 10.8, 3.5 Hz, 1 H), 3.24–3.26 (m, 1 H), 3.39 (t, J = 8.9 Hz, 1 H), 3.43–3.68 (m, 11 H), 3.73 (t, J = 9.4 Hz, 1 H), 3.78–3.96 (m, 9 H), 4.08–4.10 (m, 2 H), 4.21 (dd, J = 10.0, 3.5 Hz, 1H), 4.35–4.51 (m, 14 H), 4.52 (d, J = 7.5 Hz, 1 H, H-1B), 4.63–4.76 (m, 6 H), 4.86 (dd, J = 13.2, 6.6 Hz, 1 H), 4.92–4.98 (m, 3 H), 4.90 (d, J = 2.2 Hz, 1 H, H-1A), 5.00 (d, J = 4.3 Hz, 1 H, H-1E), 5.00–5.01 (m, 1 H, H-1D), 5.06–5.08 (m, 2 H), 5.14 (d, J = 3.6 Hz, 1 H, H-1F), 5.25 (s_{br}, 1 H), 5.64 (d, J = 2.8 Hz, 1 H), 5.66 (d, J = 2.9 Hz, 1 H, H-1C), 5.68 (d, J = 2.3 Hz, 1 H), 5.79 (m_c, 1 H), 6.98-6.99 (m, 5 H), 7.08–7.38 (m, 50 H), 8.11 (d, J = 6.6 Hz, 1 H).

^{13}C NMR (125 MHz, $CDCl_3$): δ = 15.6, 15.7, 20.9, 27.2, 27.3, 27.3, 29.1, 30.1, 38.9, 39.1, 58.9, 60.3, 64.8, 65.6, 66.3, 68.3, 68.5, 68.8, 68.9, 68.9, 69.1, 70.0, 70.6, 70.9, 71.4, 71.4, 71.7, 71.9, 72.2, 72.5, 72.9, 73.2, 73.2, 73.3, 73.4, 73.4, 73.6, 73.8, 74.6, 74.8, 74.9, 75.0, 75.2, 76.1, 77.9, 78.8, 78.9, 85.1, 92.7, 94.4 (C-1E), 96.9 (C-1C), 97.8 – 97.9 (C-1A, C-1D), 99.1 (C-1F), 101.4 (C-1B), 114.6, 126.4, 126.8, 126.9, 127.1, 127.2, 127.5, 127.5, 127.5, 127.6, 127.6, 127.7, 127.9, 127.9, 128.0, 128.0, 128.1, 128.1, 128.2, 128.2, 128.2, 128.2, 128.3, 128.3, 137.3, 137.6, 137.8, 137.8, 138.0, 138.1, 138.1, 138.2, 138.2, 138.2, 138.4, 138.7, 162.2, 170.7, 177.1, 177.2, 178.8.

IR (ATR): $\tilde{\nu}$ (cm^{-1}) = 3086, 3063, 3030, 2971, 2931, 2868, 2304, 2175, 2107, 1952, 1735.

HR-MS (ESI): m/z calcd. for $C_{137}H_{161}Cl_3N_4O_{32}Na^+$ 2502.0052, found 2502.0050.

n-Pentenyl (2-azido-3,6-di-*O*-benzyl-2-deoxy-4-*O*-pivaloyl-α-D-galactopyranosyl)-(1→3)-(2-*O*-benzyl-α-L-fucopyranosyl)-(1→2)-(3,4,6-tri-*O*-benzyl-α-D-mannopyranosyl)-(1→2)-(3,4-di-*O*-pivaloyl-α-L-fucopyranosyl)-(1→2)-(3,4,6-tri-*O*-benzyl-β-D-glucopyranosyl)-(1→3)-4,6-di-*O*-benzyl-2-deoxy-2-trichloroacetamido-β-D-glucopyranoside (48)

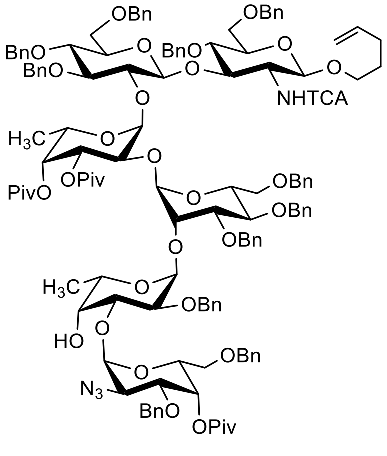

Compound **47** (168 mg, 67.0 µmol) was dissolved in a mixture of CH_3OH and CH_2Cl_2 (5.0 mL, 4:1) and a methanolic solution of NaOMe (1.2 M, 6.70 µL, 6.70 µmol) was added. After a reaction time of 3 d, the mixture was neutralized with Amberlite acidic resin (IR-120), filtered and volatiles were removed. Purification by silica gel column chromatography (pentane/EtOAc, 6:1 → 2:1) yielded 128 mg (78%) of **75** as a colorless oil.

$[\alpha]_D^{23}$= + 4.4° (c = 0.33, $CHCl_3$).

^{1}H NMR (600 MHz, $CDCl_3$): δ = 0.75 (d, J = 6.5 Hz, 3 H), 1.10 (s, 9 H), 1.16 (d, J = 6.7 Hz, 3 H), 1.17 (s, 9 H), 1.21 (s, 9 H), 1.68 (m_c, 2 H), 2.17 (m_c, 2 H), 2.90 (d, J = 5.1 Hz, 1 H), 3.11 (dd, J = 11.2, 4.5 Hz, 1H), 3.16–3.27 (m, 3 H), 3.37 (t, J = 9 Hz, 1 H), 3.46–3.66 (m, 11 H), 3.72 (t, J = 9.5 Hz, 1 H), 3.77–4.01 (m, 11 H), 4.30 (dd, J

= 10.8, 3.0 Hz, 1H), 4.37–4.46 (m, 14 H), 4.49 (d, *J* = 8.2 Hz, 1 H, H-1B), 4.64–4.74 (m, 6 H), 4.86 (q, *J* = 6.8 Hz, 1 H), 4.90 (d, *J* = 3.5 Hz, 1 H), 4.92-4.97 (m, 4 H), 4.99 (d, *J* = 8.1 Hz, 1 H, H-1A), 5.00–5.00 (m, 1 H, H-1E), 5.01, (s_{br}, 1 H, H-1D), 5.02 (d, *J* = 3.8 Hz, 1 H), 5.07 (d, *J* = 11.2 Hz, 1 H), 5.12 (d, *J* = 3.5 Hz, 1 H, H-1F), 5.23 (s_{br}, 1 H), 5.56 (d, *J* = 3.2 Hz, 1 H), 5.63 (d, *J* = 1.8 Hz, 1 H, H-1C), 5.65 (d, *J* = 2.8 Hz, 1 H), 5.79 (m_c, 1 H), 6.95–7.34 (m, 55 H), 8.15 (d, *J* = 6.6 Hz, 1 H).

^{13}C-NMR (125 MHz, $CDCl_3$): δ = 15.7, 15.8, 27.2, 27.2, 27.3, 29.0, 30.1, 38.9, 39.1, 39.1, 59.9, 60.3, 65.6, 65.6, 66.2, 68.6, 68.7, 68.9, 68.9, 69.1, 69.4, 69.8, 70.5, 71.2, 71.3, 71.4, 71.5, 71.8, 72.1, 73.1, 73.3, 73.3, 73.9, 74.1, 74.3, 74.6, 74.7, 74.8, 74.9, 75.1, 76.0, 77.9, 78.9, 79.0, 85.1, 92.7, 95.7 (C-1E), 96.9 (C-1C), 97.9 – 98.0 (C-1D, C-1A), 99.0 (C-1F), 101.4 (C-1B), 114.5, 126.4, 126.8, 126.9, 127.2, 127.3, 127.3, 127.3, 127.4, 127.6, 127.6, 127.8, 127.8, 127.9, 127.9, 127.9, 128.0, 128.1, 128.1, 128.2, 128.2, 128.2, 128.4, 136.8, 136.9, 137.6, 138.1, 138.1, 138.1, 138.2, 138.4, 138.4, 138.6, 138.7, 162.2, 177.2, 177.3, 178.9.

IR (ATR): $\tilde{\nu}$ (cm^{-1}) = 3351, 3086, 3063, 3030, 2958, 2930, 2869, 2309, 2109, 1732, 1713.

HR-MS (ESI): m/z calcd. for $C_{135}H_{159}Cl_3N_4O_{31}Na^+$ 2462.9955, found 2462.9989.

6.6. *Synthesis of the hexasaccharide 52*

***n*-Pentenyl (2-acetamido-3,6-di-*O*-benzyl-2-deoxy-4-*O*-pivaloyl-α-D-galactopyranosyl)-(1→3)-(4-*O*-acetyl-2-*O*-benzyl-α-L-fucopyranosyl)-(1→2)-(3,4,6-tri-*O*-benzyl-α-D-manno-pyranosyl)-(1→2)-(3,4-di-*O*-pivaloyl-β-L-fucopyranosyl)-(1→2)-(3,4,6-tri-*O*-benzyl-β-D-glucopyranosyl)-(1→3)-4,6-di-*O*-benzyl-2-deoxy-2-acetamido-β-D-glucopyranoside (52)**

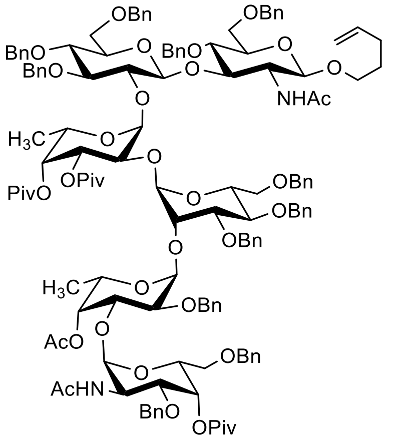

Compound **47** (40 mg, 16 μmol) was dissolved in AcOH (2.9 mL). Zn/Cu couple (407 mg) was added and the reaction mixture was stirred at 45 °C for 2 d. Afterwards it was cooled to room temperature, filtered through Celite and concentrated *in* vacuum. The residue was further dissolved in pyridine (3 mL), Ac_2O (1.5 mL) and a catalytic amount of DMAP was added. The reaction mixture was stirred overnight concentrated and co-evaporated with toluene. After filtration through a plug of silica, purification was performed by recycling HPLC (gel permeation chromatography) to obtain 26 mg (68%) hexasaccharide **52** as a colorless oil.

^{1}H NMR (600 MHz, $CDCl_3$): δ = 0.70 (d, *J* = 6.3 Hz, 3 H), 1.10 (s, 9 H), 1.12 (d, *J* = 6.0 Hz, 3 H), 1.18 (s, 9 H), 1.19 (s, 9 H), 1.64 (m_c, 2 H), 1.70 (s, 3 H), 1.88 (s, 3 H), 2.00 (s, 3 H), 2.17 (m_c, 2 H), 2.90–2.95 (m_c, 1 H), 3.19 (dd, *J* = 10.8, 4.6 Hz, 1 H), 3.32-3.37 (m, 3 H), 3.40-3.55 (m, 1 H), 3.65–3.67 (m, 2 H), 3.70-3.73 (m, 3H), 3.77-3.79 (m, 2 H), 3.88-3.94 (m, 4H), 3.97 (s_{br}, 1 H), 4.04 (dd, *J* = 10.2, 3.4 Hz, 1 H), 4.16 (d, *J* = 7.2, 1 H), 4.17-4.27 (m, 5 H), 4.31-4.58 (m, 21 H), 4.59 (d, *J* = 7.7 Hz, 1 H), 4.61-4.64 (m, 2 H), 4.66 (d, *J* = 7.3 Hz, 1 H), 4.70 (d, *J* =

12.3 Hz, 1 H), 4.73 (d, J = 10.1 Hz, 1 H), 4.80-4.83 (m, 1 H), 4.91–5.04 (m, 8 H), 5.09 (d, J = 3.5 Hz, 1 H), 5.25 (s_{br}, 1 H), 5.50 (dt, J = 10.6, 3.5 Hz, 1 H), 5.60 (d, J = 10.6 Hz, 1 H), 5.63 (d, J = 2.7, 1 H), 5.73 (d, J = 3.1 Hz, 1 H), 5.80 (m_c, 1 H), 6.97-7.29 (m, 55 H).

^{13}C NMR (125 MHz, $CDCl_3$): δ = 15.8, 20.6, 22.7, 23.4, 27.1, 27.2, 27.2, 28.9, 29.9, 38.8, 39.0, 39.1, 48.8, 52.8, 59.3, 64.9, 65.6, 65.6, 67.7, 68.7, 68.9, 68.9, 69.1, 69.4, 70.3, 70.6, 71.0, 71.2, 71.7, 72.2, 72.5, 72.9, 73.1, 73.2, 73.2, 73.3, 73.3, 73.4, 73.7, 73.6, 74.3, 74.3, 74.5, 74.6, 74.7, 74.9, 75.6, 77.6, 77.7, 78.1, 78.8, 84.7, 93.8, 96.9, 97.9, 98.8, 100.2, 101.6, 114.7, 126.8, 127.1, 127.2, 127.3, 127.3, 127.4, 127.4, 127.5, 127.5, 127.6, 127.7, 127.7, 127.8, 128.0, 128.1, 128.1, 128.2, 128.2, 128.2, 128.2, 128.3, 128.4, 128.4, 128.5, 137.3, 137.3, 137.8, 137.9, 138.0, 138.2, 138.3, 138.3, 138.4, 138.4, 138.8, 169.6, 170.2, 170.8, 177.3, 177.3, 177.6.

IR (ATR): $\tilde{\nu}$ (cm^{-1}) = 3080, 3050, 3020, 2975, 2930, 2870, 1734, 1670, 1605, 1529, 1496.

HR-MS (ESI): m/z calcd. for $C_{139}H_{168}N_2O_{33}Na^+$ 2417.1423, found 2417.1456.

6.7. *Synthesis of the heptasaccharide 49*

n-Pentenyl (2-azido-3,6-di-*O*-benzyl-2-deoxy-4-*O*-pivaloyl-α-D-galactopyranosyl)-(1→3)-[(methyl3,4-Di-*O*-benzyl-2-*O*-pivaloyl-β-D-glucopyranosyluronate)-(1→4)]-(2-*O*-benzyl-α-L-fucopyranosyl)-(1→2)-(3,4,6-tri-*O*-benzyl-α-D-mannopyranosyl)-(1→2)-(3,4-di-*O*-pivaloyl-α-L-fucopyranosyl)-(1→2)-(3,4,6-tri-*O*-benzyl-β-D-glucopyranosyl)-(1→3)-4,6-di-*O*-benzyl-2-deoxy-2-trichloroacetamido-β-D-glucopyranoside (49)

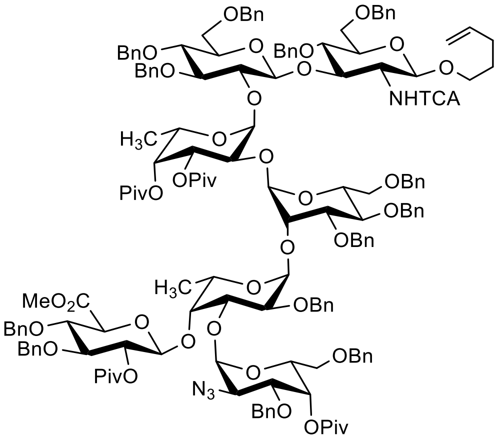

Compound **48** (20 mg, 8.2 μmol) and compound **4** (20 mg, 33 μmol) were mixed, co-evaporated with toluene (3x), dried in vacuum for 1 h and dissolved in dichloromethane (0.50 mL). A diluted solution of TESOTf (0.22 μL, 1.2 μmol) in CH_2Cl_2 was added slowly at 0 °C and the reaction mixture was stirred for 24 h. Afterwards it was quenched by the addition of pyridine and evaporated to dryness. The resulting crude product was purified by HPLC (gel permeation chromatography) to obtain 13 mg (54% isolated, 64% brsm) of heptasaccharide **49** as a colorless oil.

$[\alpha]_D^{25}$ = – 0.9° (c = 0.11, $CHCl_3$).

^{1}H NMR (600 MHz, $CDCl_3$): δ = 0.83 (d, J = 6.7 Hz, 3 H), 1.06 (s, 9 H), 1.14 (s, 3 H), 1.13 (s, 9 H), 1.14 (s, 9 H), 1.16 (s, 9 H), 1.66 (m_c, 2 H), 2.15 (m_c, 2 H), 2.90 (d, J = 4.5 Hz, 1 H), 3.19-3.58 (m, 12 H), 3.60 (s, 3 H), 3.62–3.66 (m, 6 H), 3.69 (t, J = 9.5 Hz, 1 H), 3.76–3.94 (m, 10 H), 3.95 (dd, J = 10.3, 3.2 Hz, 1 H), 4.04 (t, J = 2.5 Hz, 1 H), 4.07 (d, J = 11.5 Hz, 1 H), 4.14 (q, J = 6.5 Hz, 1 H), 4.19 (dd, J = 11.1, 3.3 Hz, 1 H), 4.27 (t, J = 6.3 Hz, 1 H), 4.32– 4.46 (m, 8 H), 4.47 (d, J = 8.2 Hz, 1 H), 4.48-4.69 (m, 9 H), 4.72 (s, 1 H), 4.74-4.75 (m, 1 H), 4.79-4.82

(m, 2 H), 4.90-4.95 (m, 4 H), 4.97 (d, J = 7.5 Hz, 1 H), 4.97 (d, J = 3.6 Hz, 1 H), 4.99–5.01 (m, 3 H), 5.04 (d, J = 11.6 Hz, 1 H), 5.17 (dd, J = 9.3, 7.7 Hz, 1 H), 5.22 (s_{br}, 1 H), 5.59 (d, J = 2.8 Hz, 1 H), 5.61 (d, J = 2.8 Hz, 1 H), 5.64 (s_{br}, 1 H), 5.77 (m_c, 1 H), 6.92– 7.40 (m, 65 H), 8.04 (d, J = 5.5 Hz, 1 H).

^{13}C NMR (125 MHz, $CDCl_3$): δ = 15.7, 16.7, 27.2, 27.2, 27.3, 27.4, 29.1, 30.1, 38.8, 38.8, 39.0, 39.0, 52.7, 59.5, 60.3, 65.7, 66.3, 67.1, 68.2, 68.8, 68.9, 69.0, 69.1, 69.1, 69.1, 70.7, 71.1, 71.2, 71.3, 71.3, 71.7, 71.7, 72.8, 72.9, 72.9, 73.1, 73.3, 73.3, 73.5, 73.7, 74.2, 74.6, 74.7, 74.9, 75.1, 76.1, 77.9, 78.8, 78.8, 79.8, 82.4, 85.0, 92.7, 95.0 (C–1E), 97.0, (C–1C), 97.8 – 97.9 (C-1A, C-1D)*, 99.1 (C-1F), 100.9 (C-1G), 101.5 (C-1B), 114.5, 126.6, 126.7, 126.7, 126.8, 126.9, 126.9, 127.2, 127.2, 127.3, 127.3, 127.3, 127.4, 127.4, 127.5, 127.5, 127.6, 127.7, 127.8, 127.9, 127.9, 127.9, 128.0, 128.0, 128.0, 128.1, 128.2, 128.2, 128.2, 128.2, 128.3, 128.4, 128.5, 128.7 128.7, 137.3, 137.7, 137.7, 138.0, 138.1, 138.1, 138.2, 138.3, 138.3, 138.3, 138.4, 138.4, 138.8, 138.9, 162.1, 168.9, 176.2, 177.2, 177.5, 178.7.

IR (ATR): $\tilde{\nu}$ (cm^{-1}) = 3086, 3062, 3029, 2962, 2927, 2869, 2357, 2108, 1735, 1715, 1604.

HR-MS (ESI): m/z calcd. for $C_{161}H_{189}$ $Cl_3N_4O_{38}Na^+$ 2917.1948, found. 2917.1989.

6.8. *Synthesis of the heptasaccharide 54.*

_n_-Pentenyl (2-acetamido-3,6-di-_O_-benzyl-2-deoxy-4-_O_-pivaloyl-α-D-galactopyranosyl)-(1→3)-[(methyl-3,4-di-_O_-benzyl-2-_O_-pivaloyl-β-D-glucopyranosyluronate)-(1→4)]-(2-_O_-benzyl-α-L-fucopyranosyl)-(1→2)-(3,4,6-tri-_O_-benzyl-α-D-mannopyranosyl)-(1→2)-(3,4-di-_O_-pivaloyl-α-L-fucopyranosyl)-(1→2)-(3,4,6-tri-_O_-benzyl-β-D-glucopyranosyl)-(1→3)-2-acetamido-4,6-di-_O_-benzyl-2-deoxy-β-D-glucopyranoside (54)

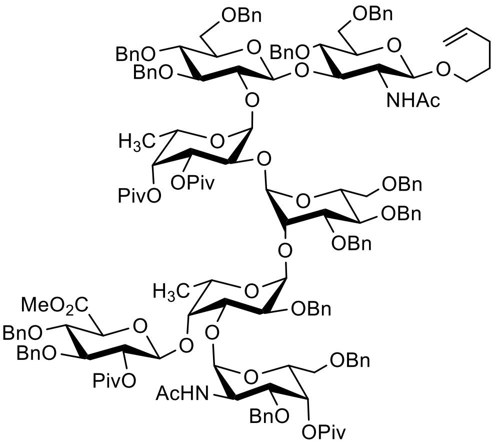

Compound **49** (79 mg, 27 µmol) was dissolved in AcOH (5 mL). Zn/Cu couple (690 mg) was added and the reaction mixture was stirred at 45 °C for 2 d. Afterwards it was cooled to room temperature, filtered through Celite and concentrated *in* vacuum. The residue was further dissolved in pyridine (5 mL), Ac_2O (2.5 mL) and a catalytic amount of DMAP was added. The reaction mixture was stirred overnight, concentrated and co-evaporated with toluene. After filtration through a plug of silica, purification was performed by recycling HPLC (gel permeation chromatography) to obtain 76 mg (68%) heptasaccharide **54** as a colorless oil

$[\alpha]_D^{22}$= – 14.5° (c = 0.10, $CHCl_3$).

^{1}H NMR (600 MHz, $CDCl_3$): δ = 0.74 (d, J = 6.7 Hz, 3 H), 1.04 (s, 9 H), 1.14 (d, J = 6.9 Hz, 3 H), 1.15 (s, 9 H), 1.16 (s, 9 H), 1.20 (s, 9 H), 1.63 (s, 3 H), 1.66 (m_c, 2 H), 1.88 (s, 3 H), 2.15 (m_c, 2 H), 2.90–2.91 (m, 1 H), 3.10 (dd, J = 11.0, 5.5 Hz, 1 H), 3.32–3.40 (m, 4 H), 3.44–3.64 (m, 8 H), 3.65 (s, 3 H), 3.65–3.73 (m, 4 H), 3.75 (dd, J

= 11.1, 3.1 Hz, 1 H), 3.80– 3.99 (m, 10 H), 4.03 (dd, *J* = 3.3, 10.5 Hz, 1 H), 4.09 (q, *J* = 7.7 Hz, 1 H), 4.14 (dd, *J* = 10.5, 3.3 Hz, 1 H), 4.20–4.61 (m, 18 H), 4.64 (d, *J* = 3.5 Hz, 1 H), 4.66 (d, *J* = 11.0 Hz, 1 H), 4.70 (d, *J* = 3.1 Hz, 1 H), 4.71–4.79 (m, 6 H), 4.81 (d, *J* = 11.0 Hz, 1 H), 4.92–5.05 (m, 5 H), 5.09 (d, *J* = 7.7 Hz, 1 H), 5.10 (d, *J* = 3.5 Hz, 1 H), 5.23–5.24 (m, 2 H), 5.54 (d, *J* = 2.5 Hz, 1 H), 5.71 (d, *J* = 2.8 Hz, 1 H), 5.64 (s_{br}, 1 H), 5.79 (m_c, 1 H), 5.97 (d, *J* = 9.5 Hz, 1 H), 6.93–7.36 (m, 67 H).

^{13}C NMR (125 MHz, $CDCl_3$): δ = 15.9, 16.8, 23.1, 23.4, 27.2, 27.2, 27.3, 27.4, 29.0, 30.0, 38.7, 38.9, 39.0, 39.2, 48.5, 52.8, 53.4, 59.2, 65.7, 66.2, 67.1, 68.7, 68.8, 69.0, 69.3, 69.4, 69.5, 70.6, 70.6, 71.0, 71.1, 71.5, 71.5, 71.9, 72.1, 72.9, 73.2, 73.2, 73.3, 73.4, 73.9, 74.3, 74.4, 74.5, 74.6, 74.7, 74.9, 75.0, 76.0, 77.7, 78.1, 79.2, 79.7, 82.0, 84.6, 94.6, 97.0, 98.2, 98.7, 99.9, 100.6, 101.8, 114.6, 126.6, 127.0, 127.1, 127.1, 127.1, 127.2, 127.3, 127.3, 127.4, 127.5, 127.5, 127.5, 127.6, 127.7, 127.7, 127.8, 127.8, 127.8, 127.9, 127.9, 128.0, 128.0, 128.1, 128.1, 128.1, 128.2, 128.2, 128.3, 128.3, 128.7 128.8, 137.2, 137.6, 137.7, 137.7, 137.9, 138.0, 138.0, 138.0, 138.1, 138.2, 138.3, 138.3, 138.4, 138.8, 138.9, 168.8, 169.4, 170.6, 176.8, 177.2, 177.9.

IR (ATR): $\tilde{\nu}$ (cm^{-1}) = 3086, 3063, 3030, 2970, 2930, 2870, 1734, 1679, 1605, 1529, 1496.

HR-MS (ESI): m/z calcd. for $C_{163}H_{196}N_2O_{39}Na^+$ 2828.3307, found. 2828.3317.

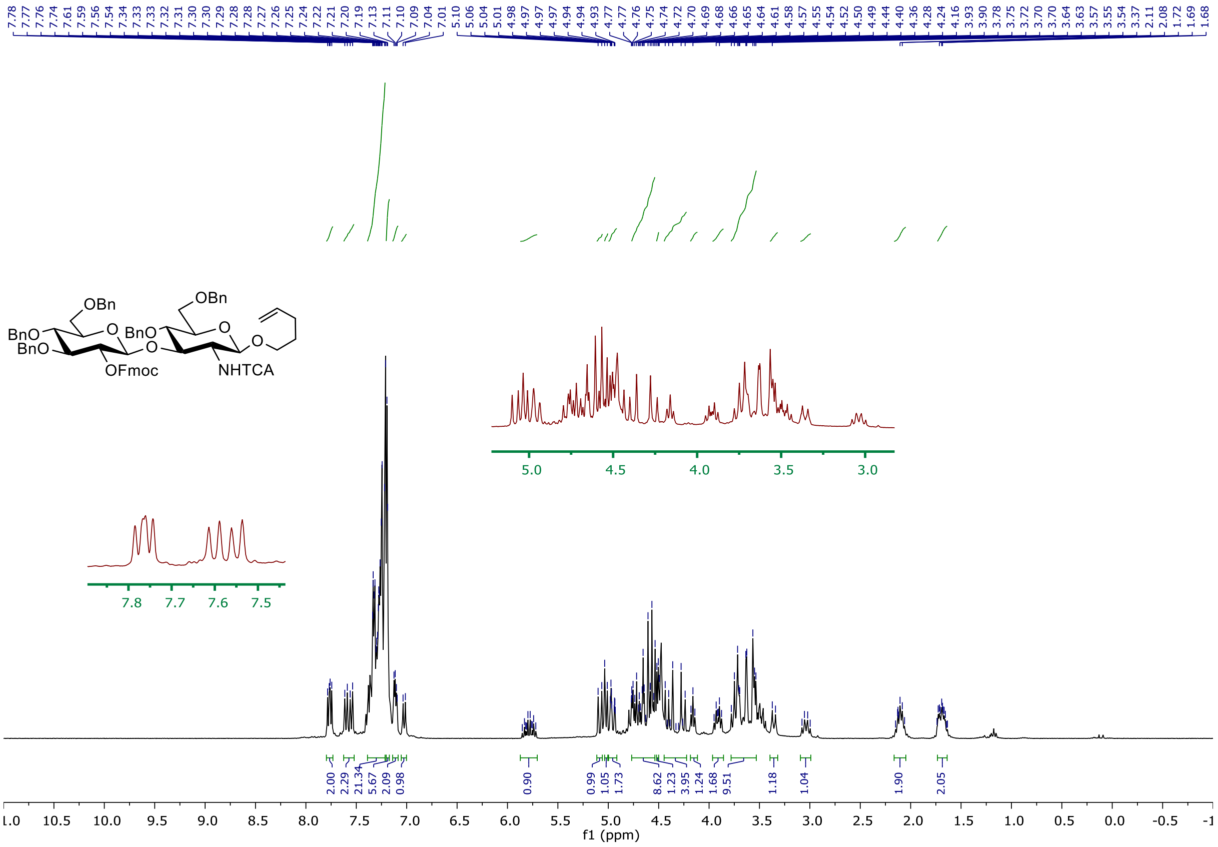

Figure 31. ^{1}H NMR ($CDCl_3$, 300 MHz) spectrum of **disaccharide 28**

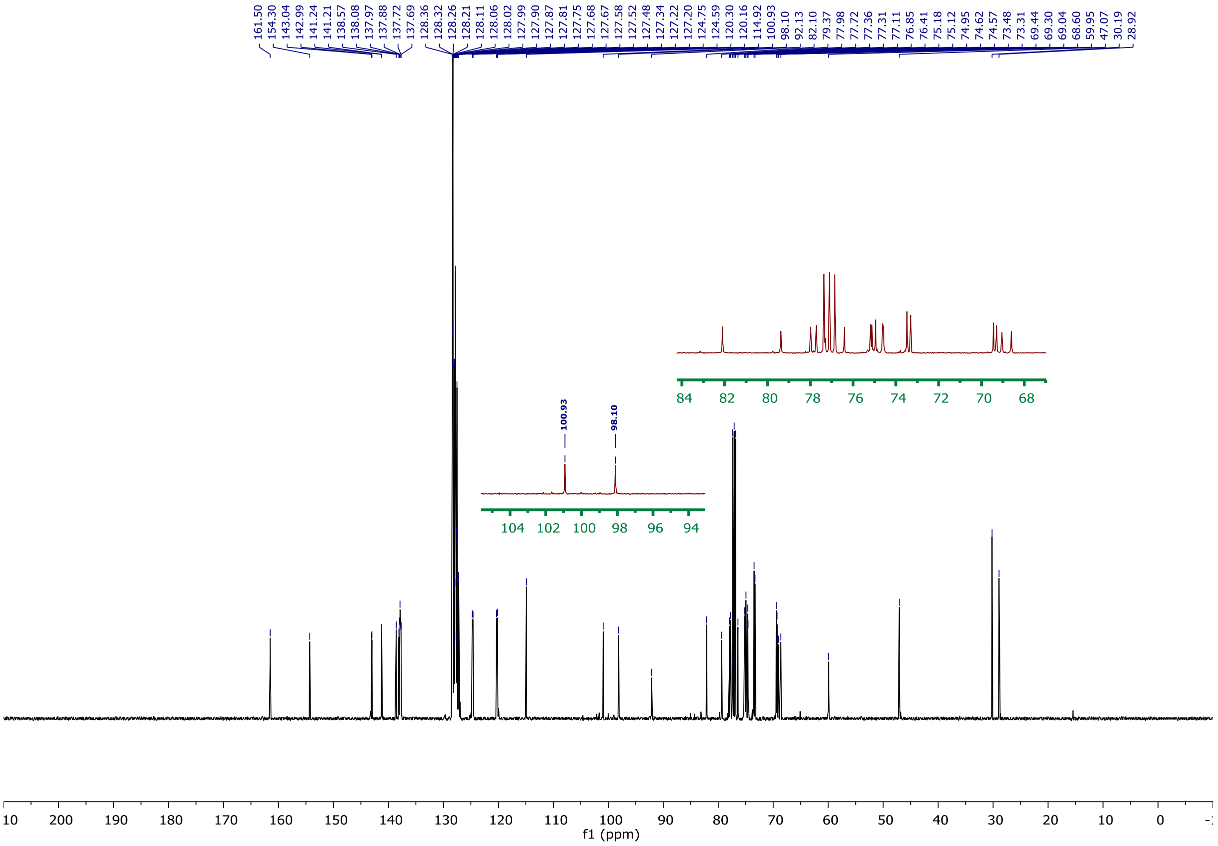

Figure S32. ^{13}C NMR ($CDCl_3$, 125 MHz) spectrum of **disaccharide 28**

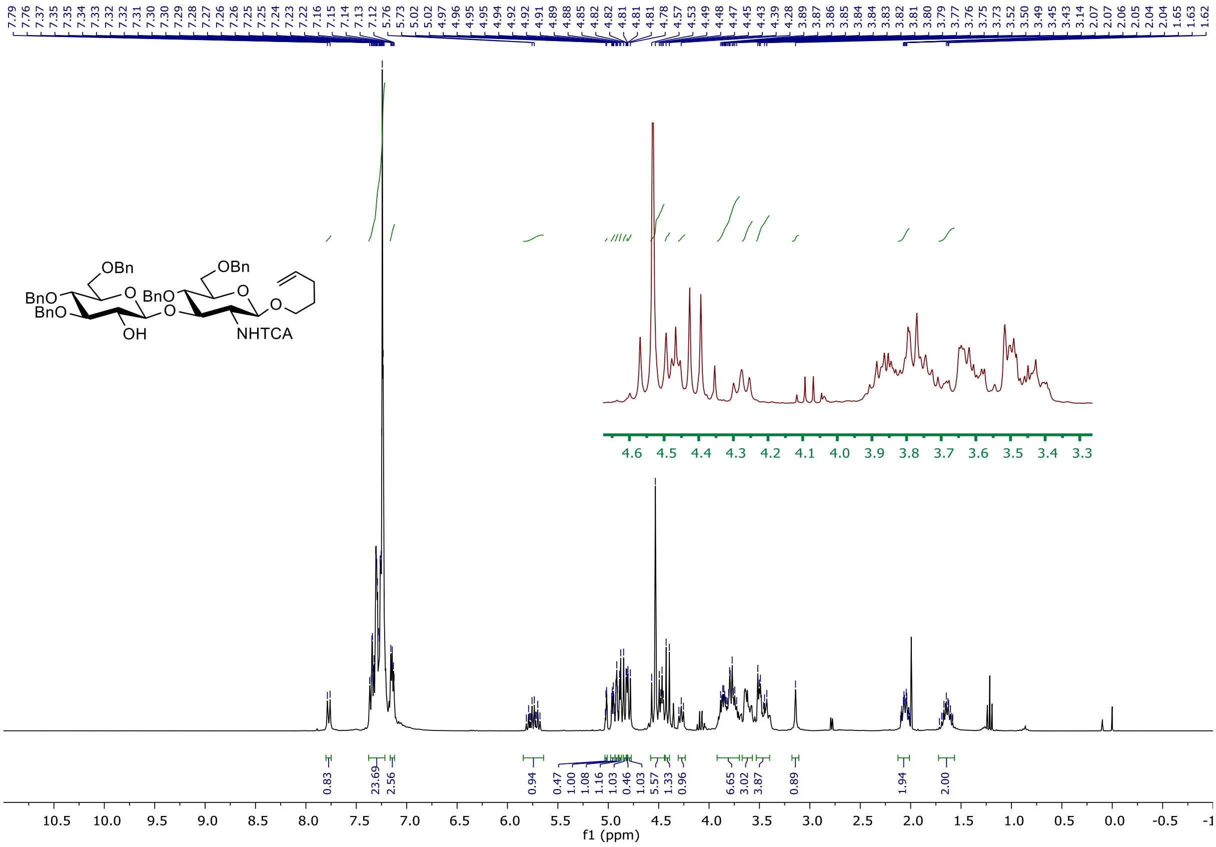

Figure S33. ^{1}H NMR ($CDCl_3$, 600 MHz) spectrum of **disaccharide acceptor 29**

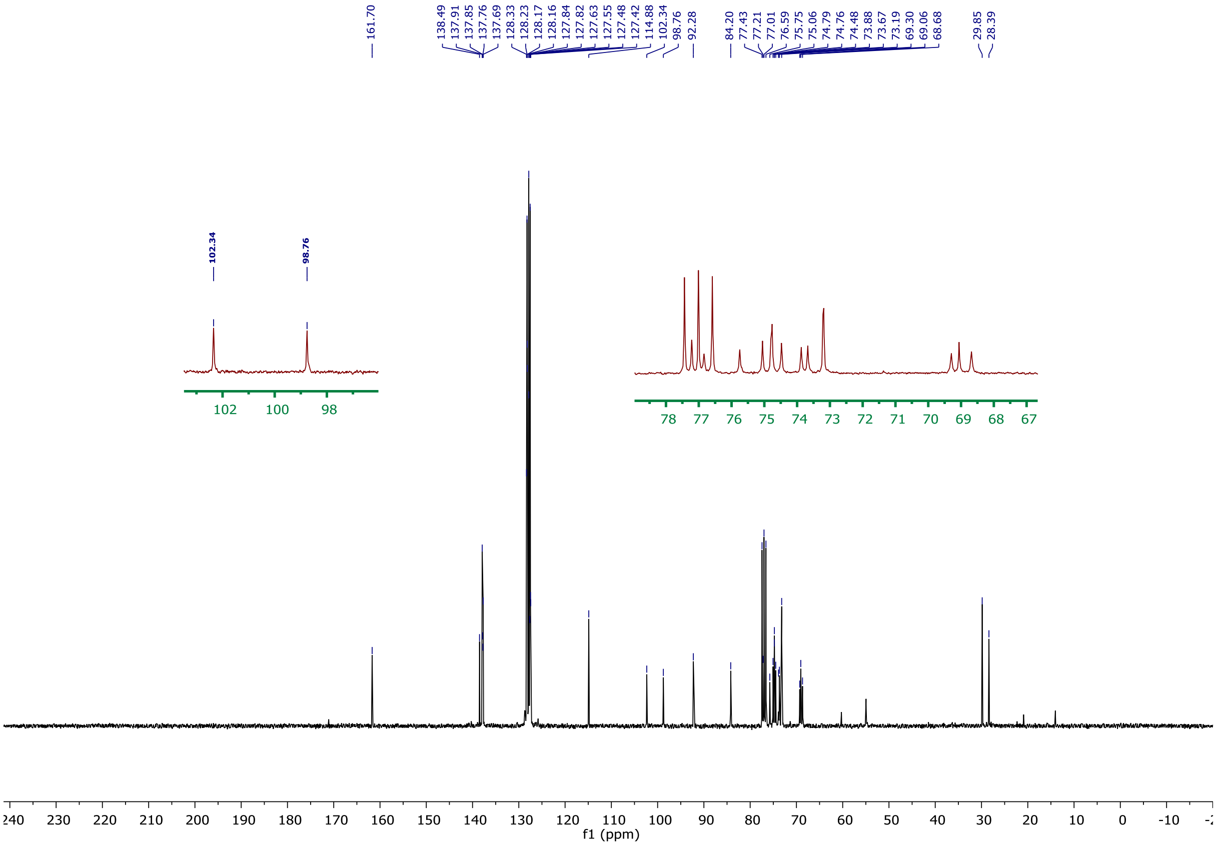

Figure S34. ^{13}C NMR ($CDCl_3$, 150 MHz) spectrum of **disaccharide acceptor 29**

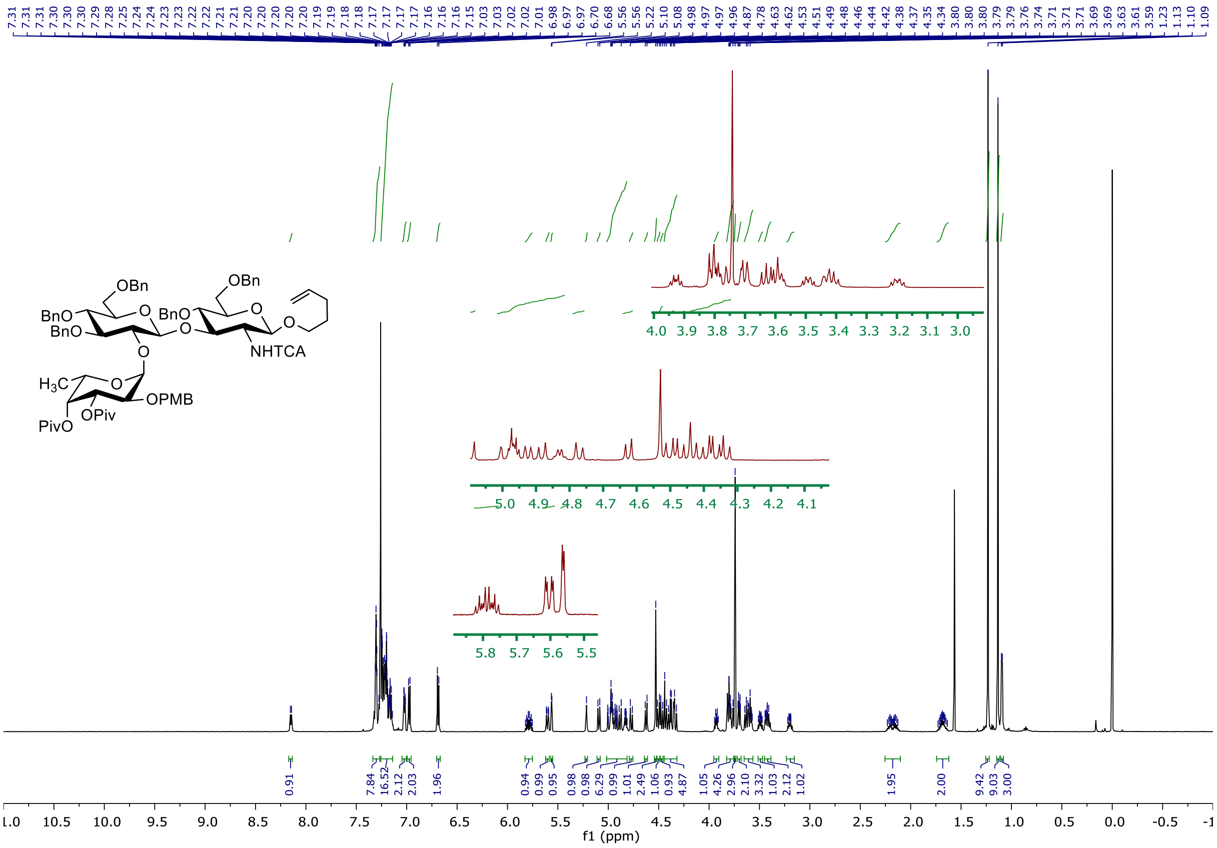

Figure S35. ^{1}H NMR ($CDCl_3$, 300 MHz) spectrum of **trisaccharide 30**

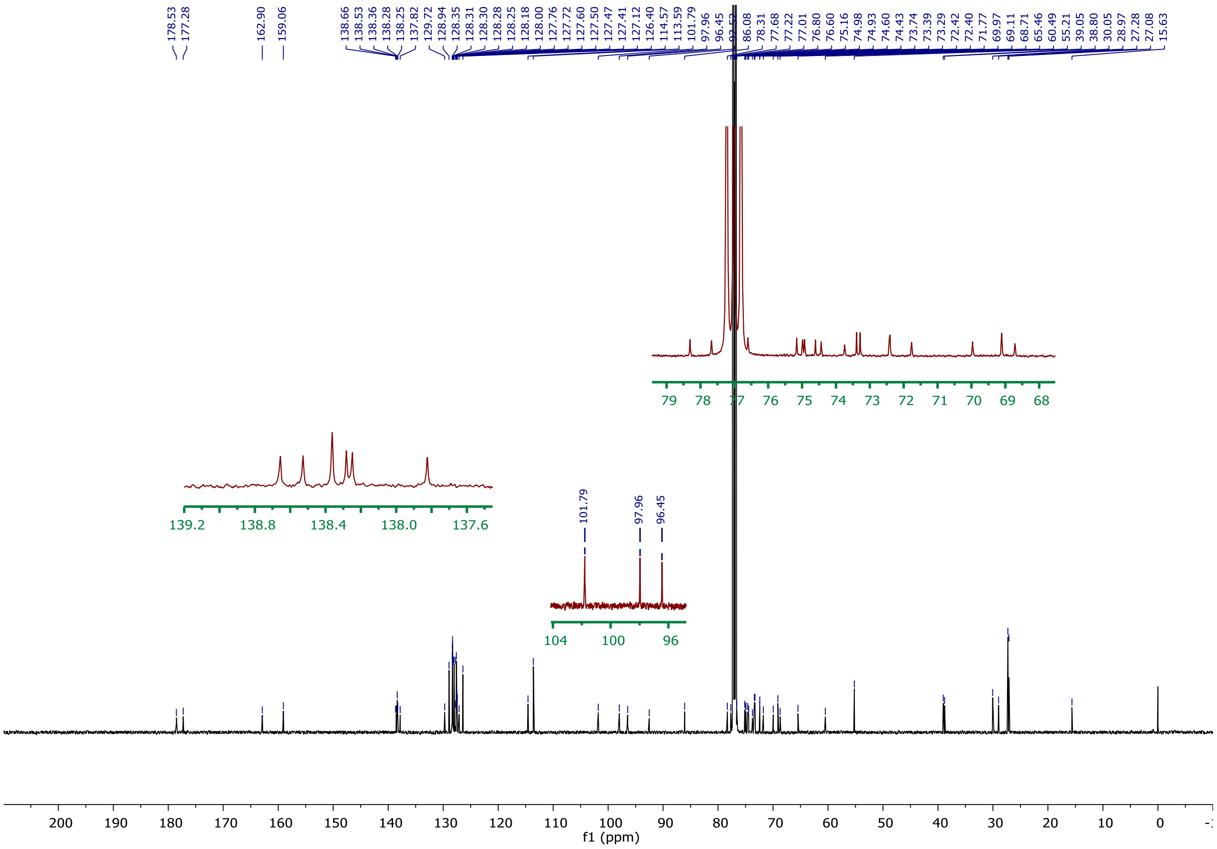

Figure S36. ^{13}C NMR ($CDCl_3$, 75 MHz) spectrum of **trisaccharide 30**

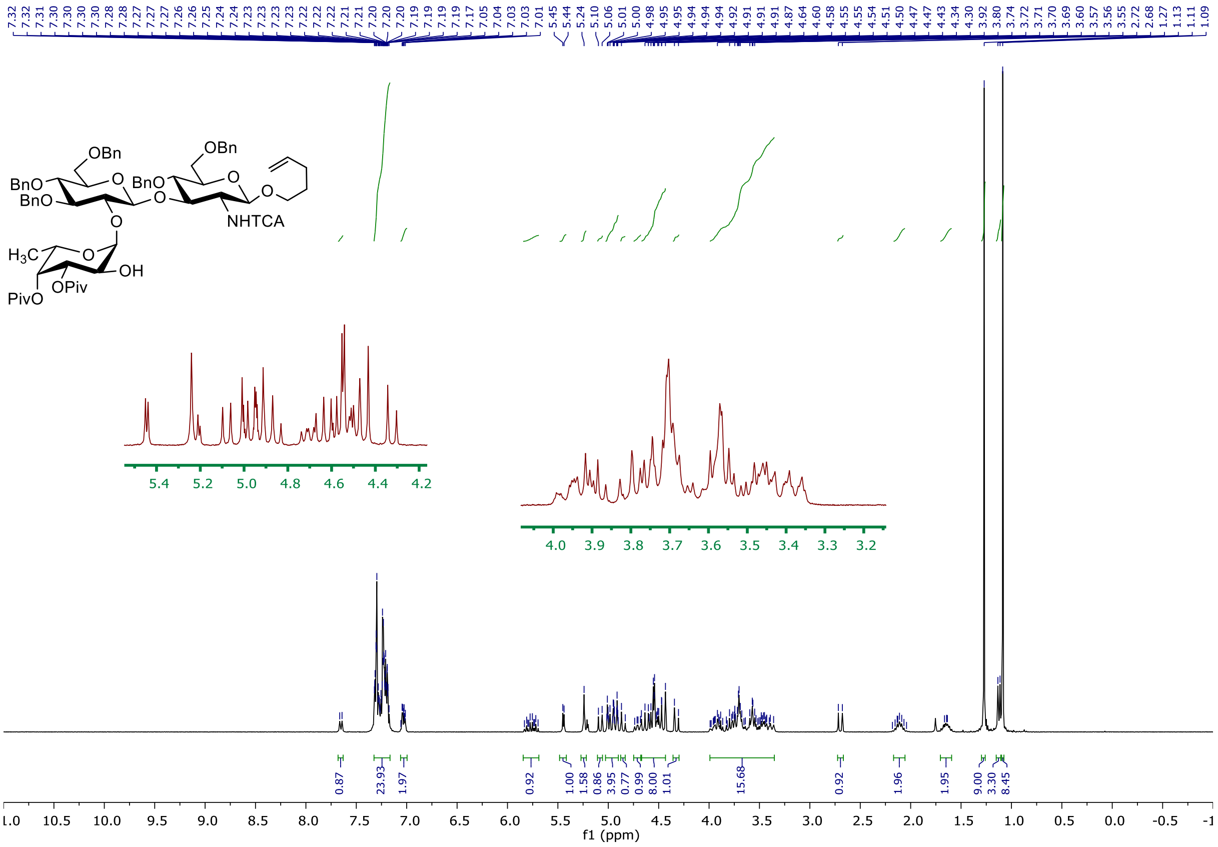

Figure S37. ^{1}H NMR ($CDCl_3$, 300 MHz) spectrum of **trisaccharide acceptor 31**

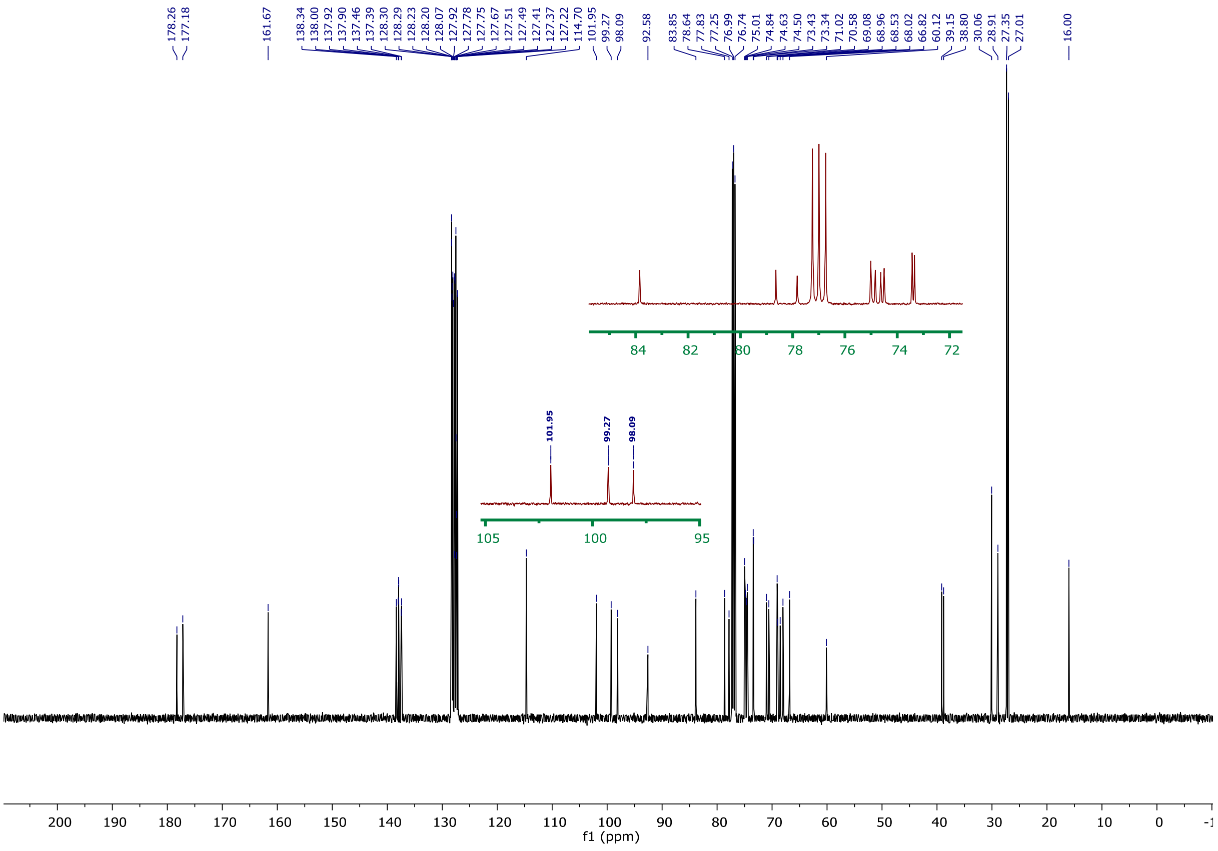

Figure S38. ^{13}C NMR ($CDCl_3$, 75 MHz) spectrum of **trisaccharide acceptor 31**

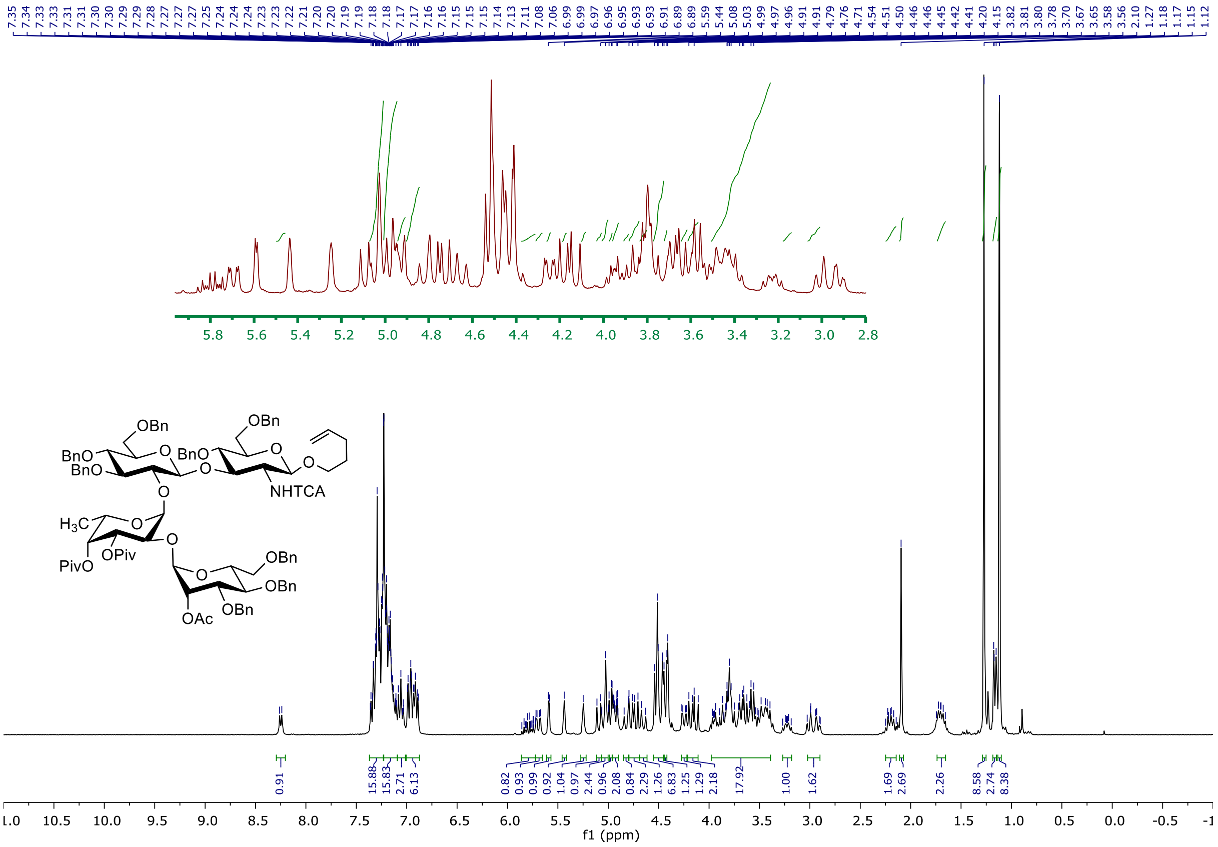

Figure S39. ^{1}H NMR ($CDCl_3$, 300 MHz) spectrum of **tetrasaccharide 3**

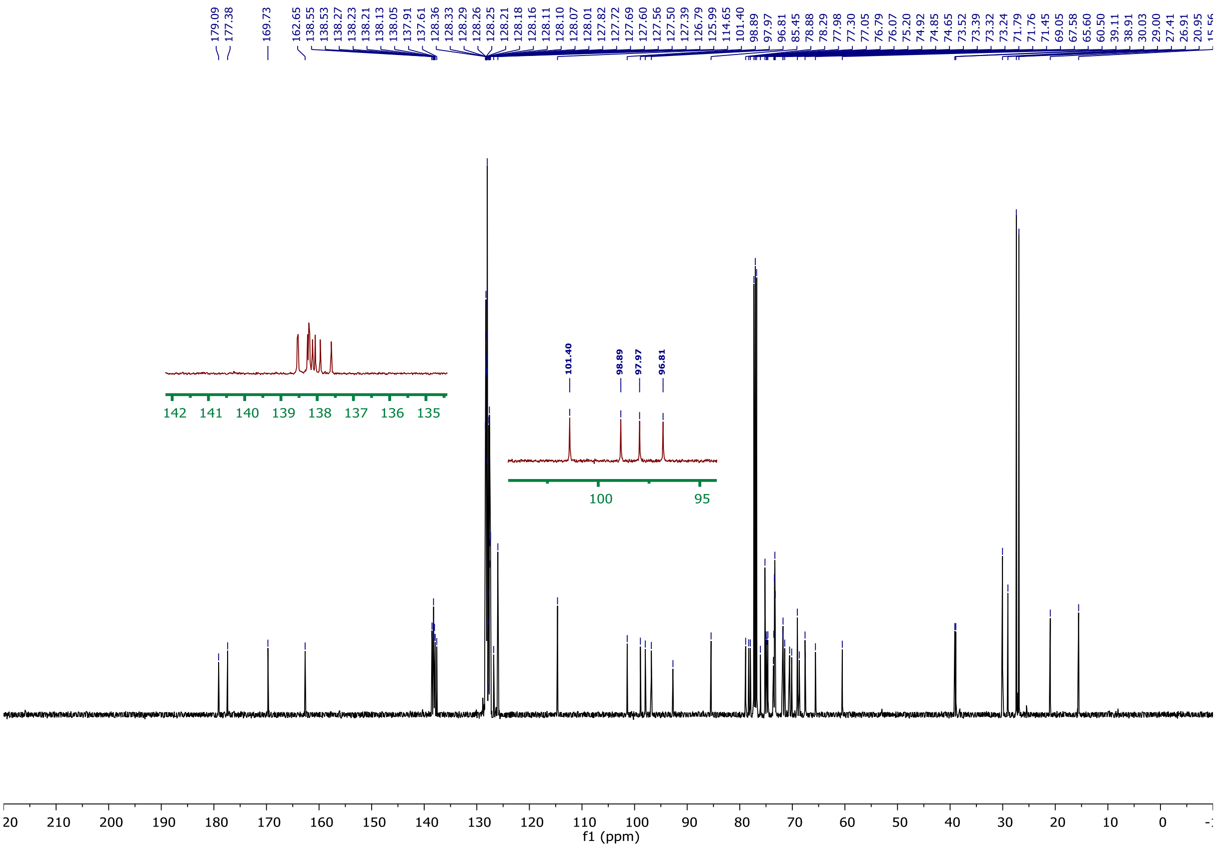

Figure S40. ^{13}C NMR ($CDCl_3$, 75 MHz) spectrum of **tetrasaccharide 3**

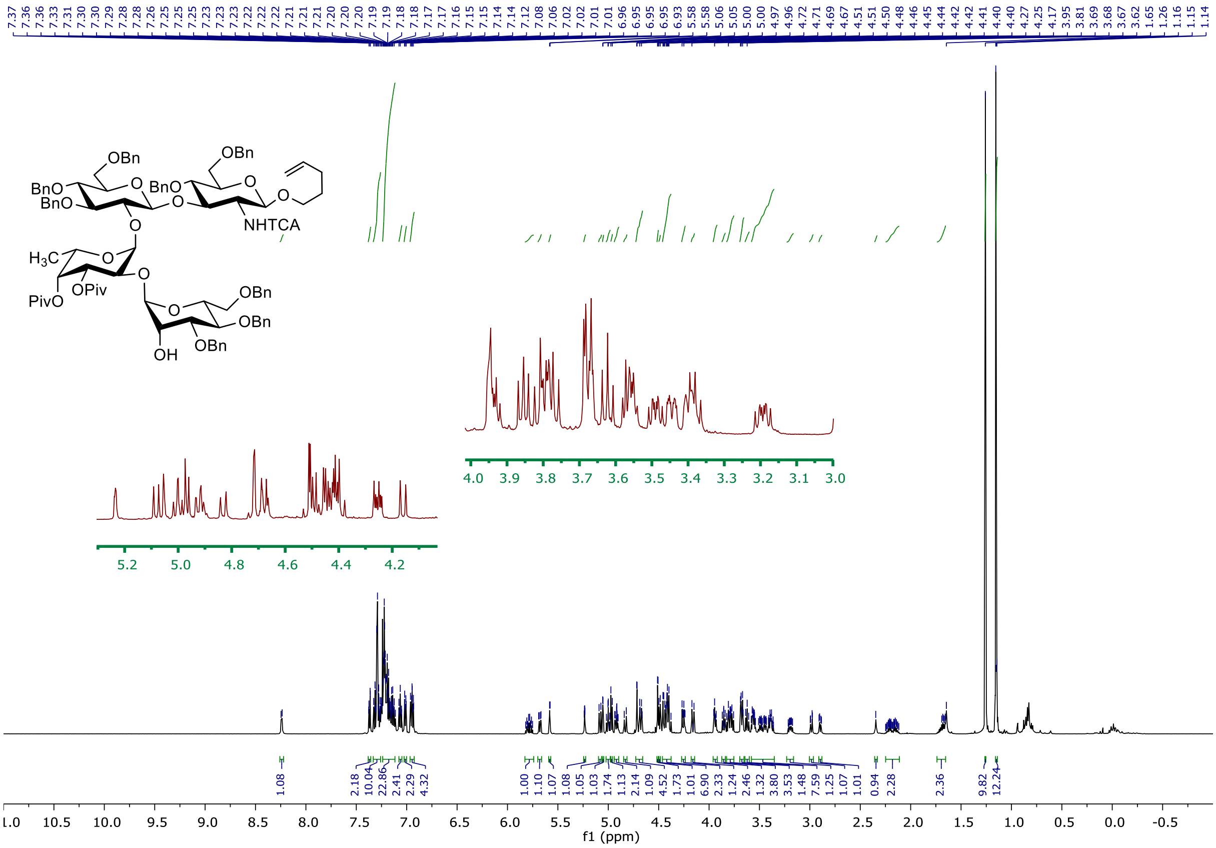

Figure S41. ^{1}H NMR ($CDCl_3$, 600 MHz) spectrum of **tetrasaccharide acceptor 32**

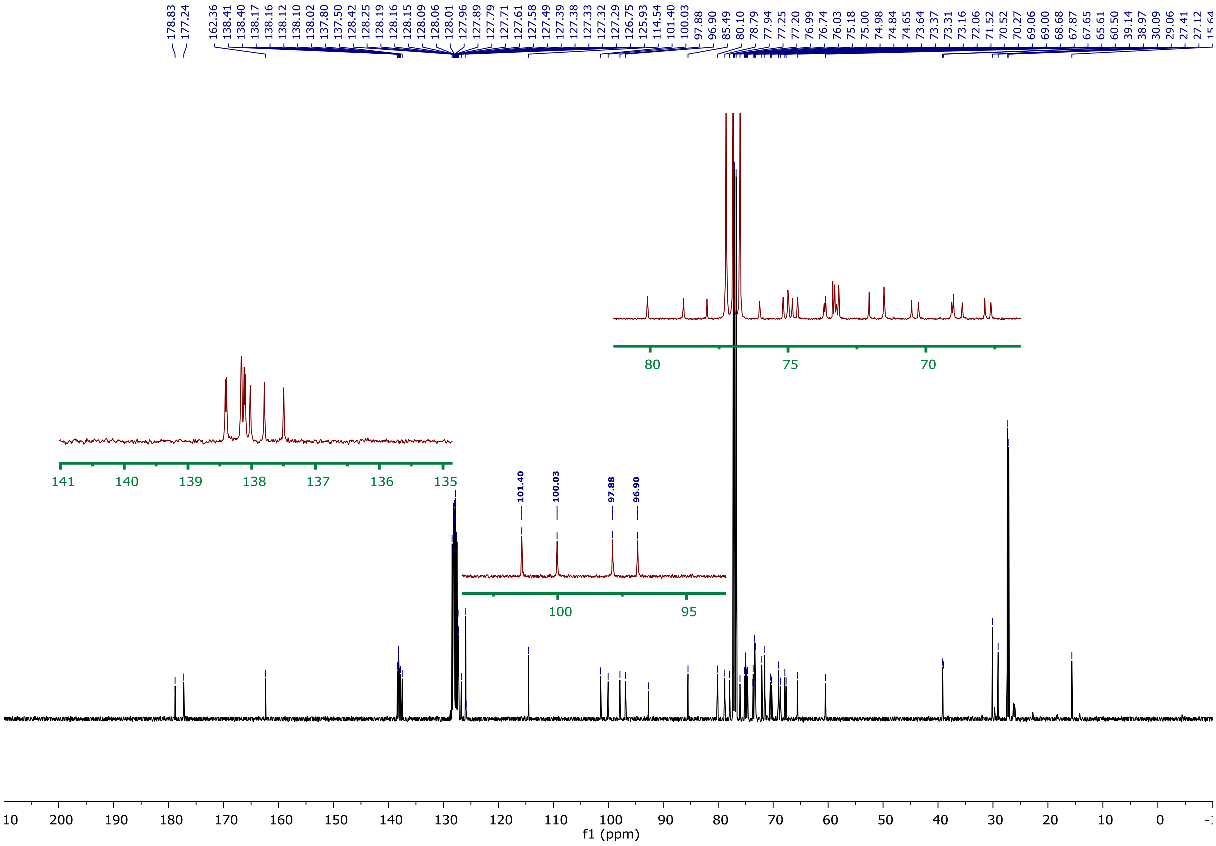

Figure S42. ^{13}C NMR ($CDCl_3$, 150 MHz) spectrum of **tetrasaccharide acceptor 32**

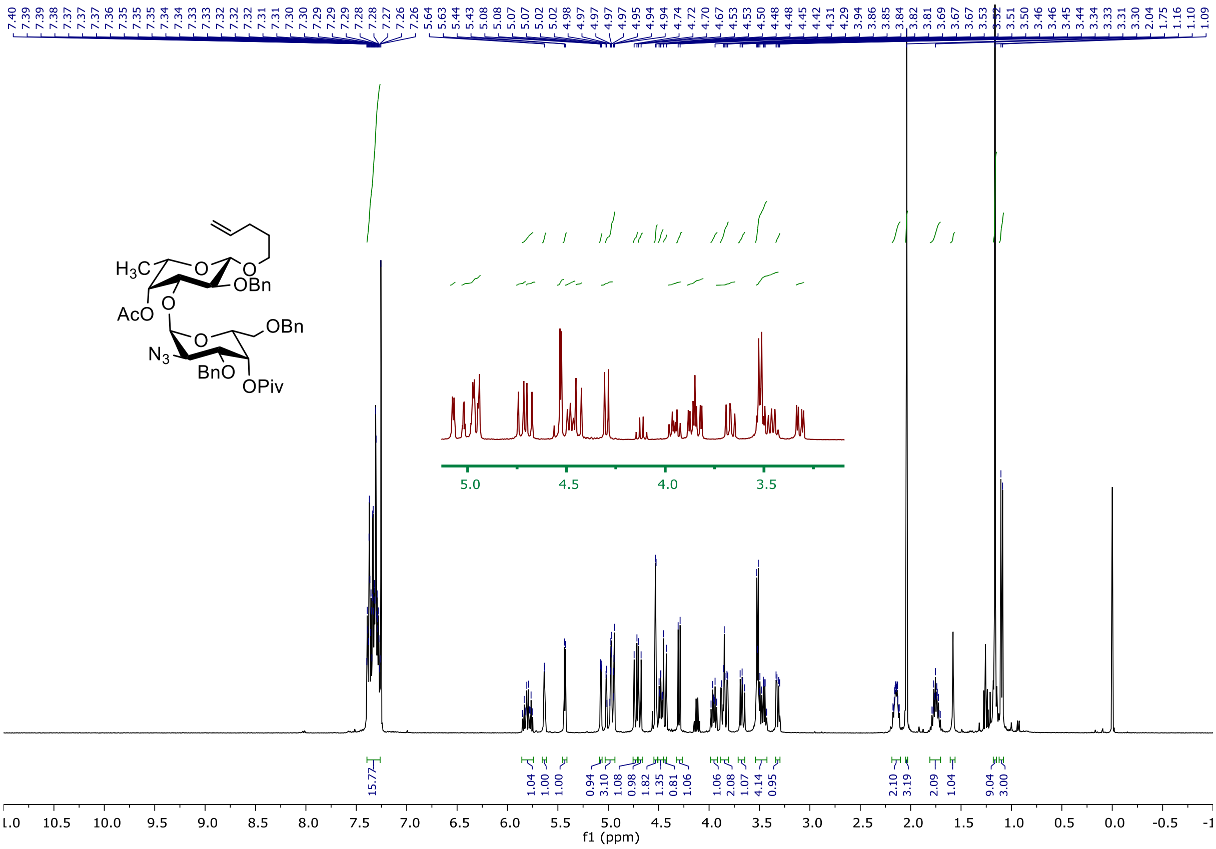

Figure S43. ^{1}H NMR ($CDCl_3$, 400 MHz) spectrum of **disaccharide 38**

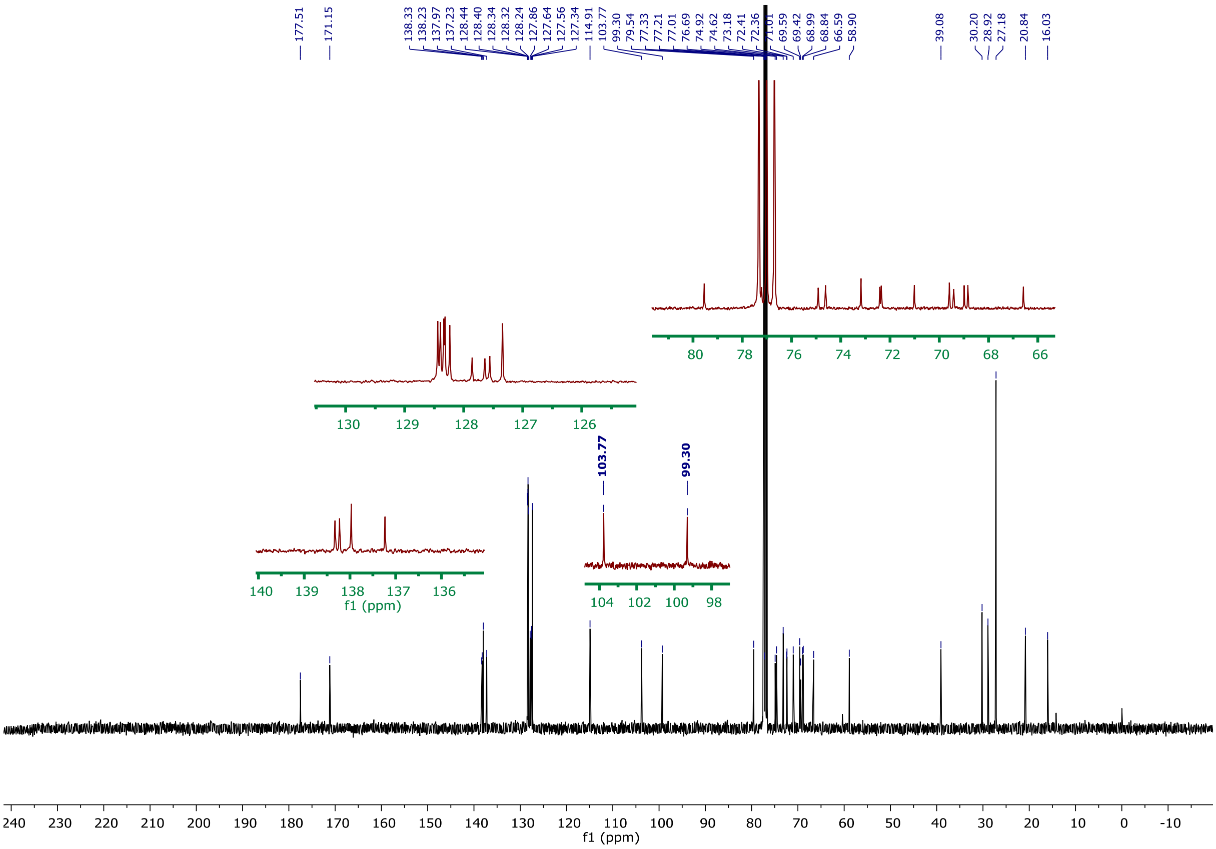

Figure S44. ^{13}C NMR ($CDCl_3$, 100 MHz) spectrum of **disaccharide 38**

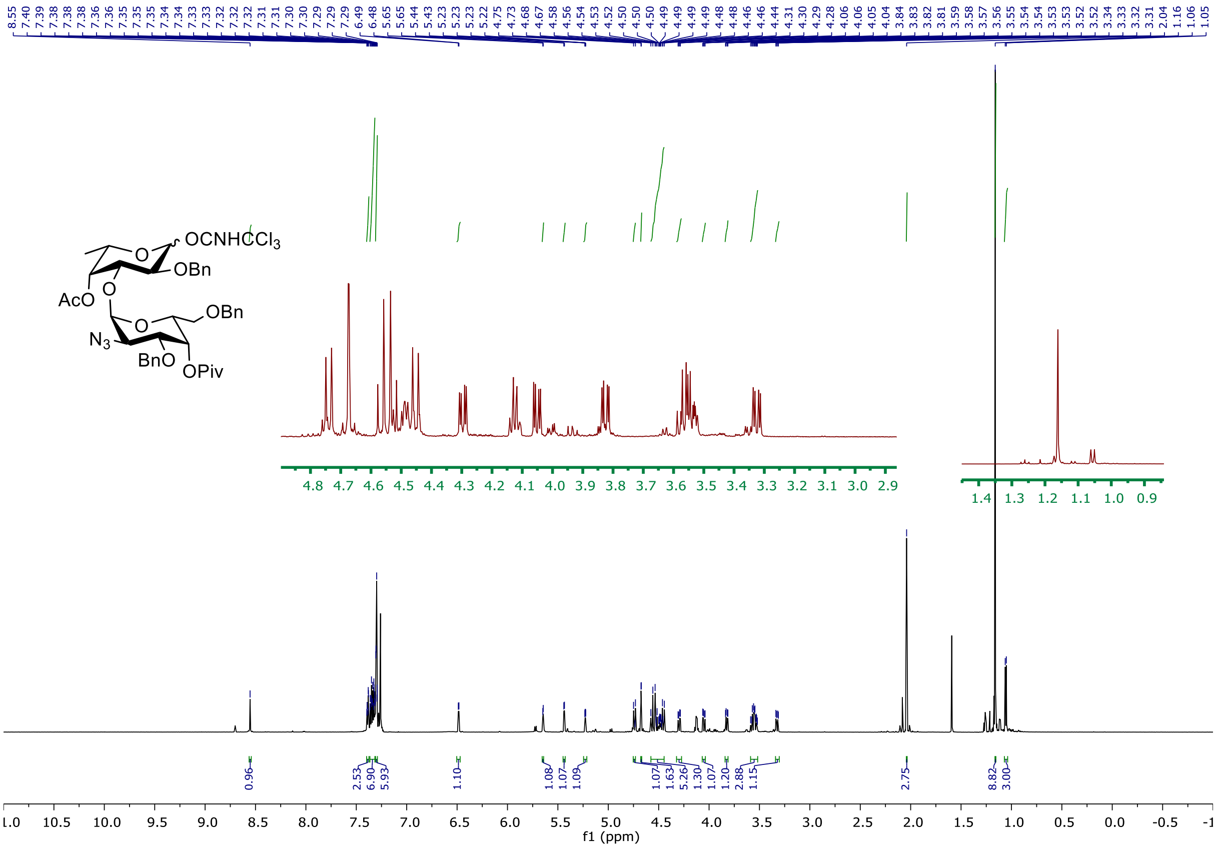

Figure S45. ^{1}H NMR ($CDCl_3$, 500 MHz) spectrum of **disaccharide donor 2**

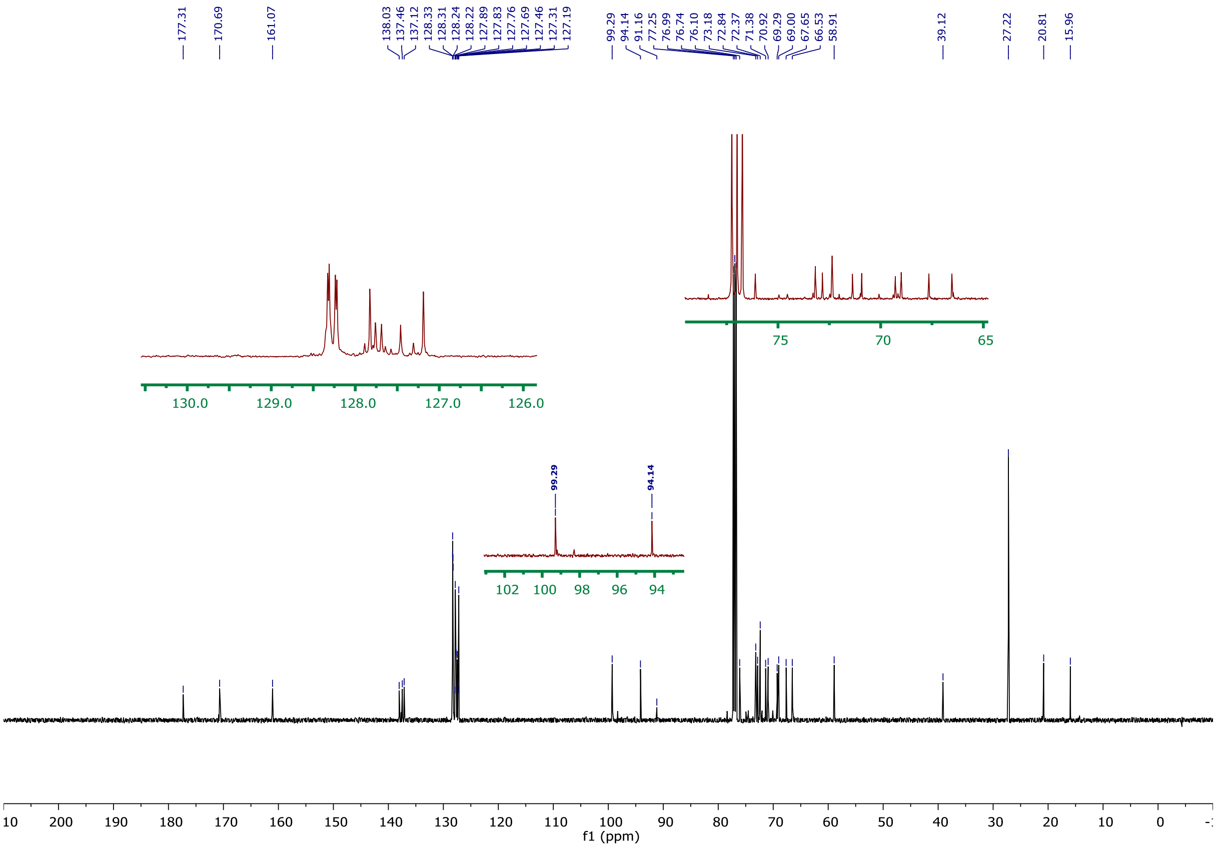

Figure S46. ^{13}C NMR ($CDCl_3$, 125 MHz) spectrum of **disaccharide donor 2**

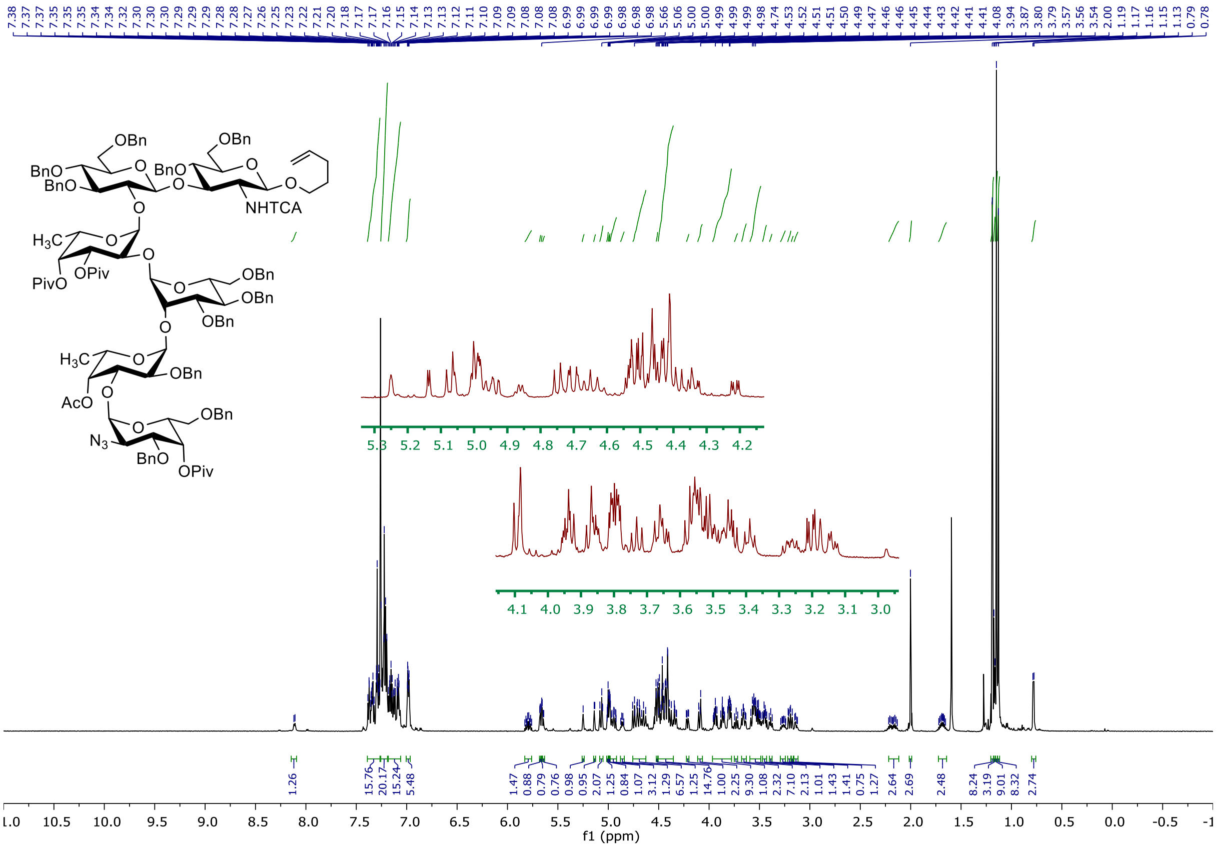

Figure S47. ^{1}H NMR ($CDCl_3$, 600 MHz) spectrum of **hexasaccharide 47**

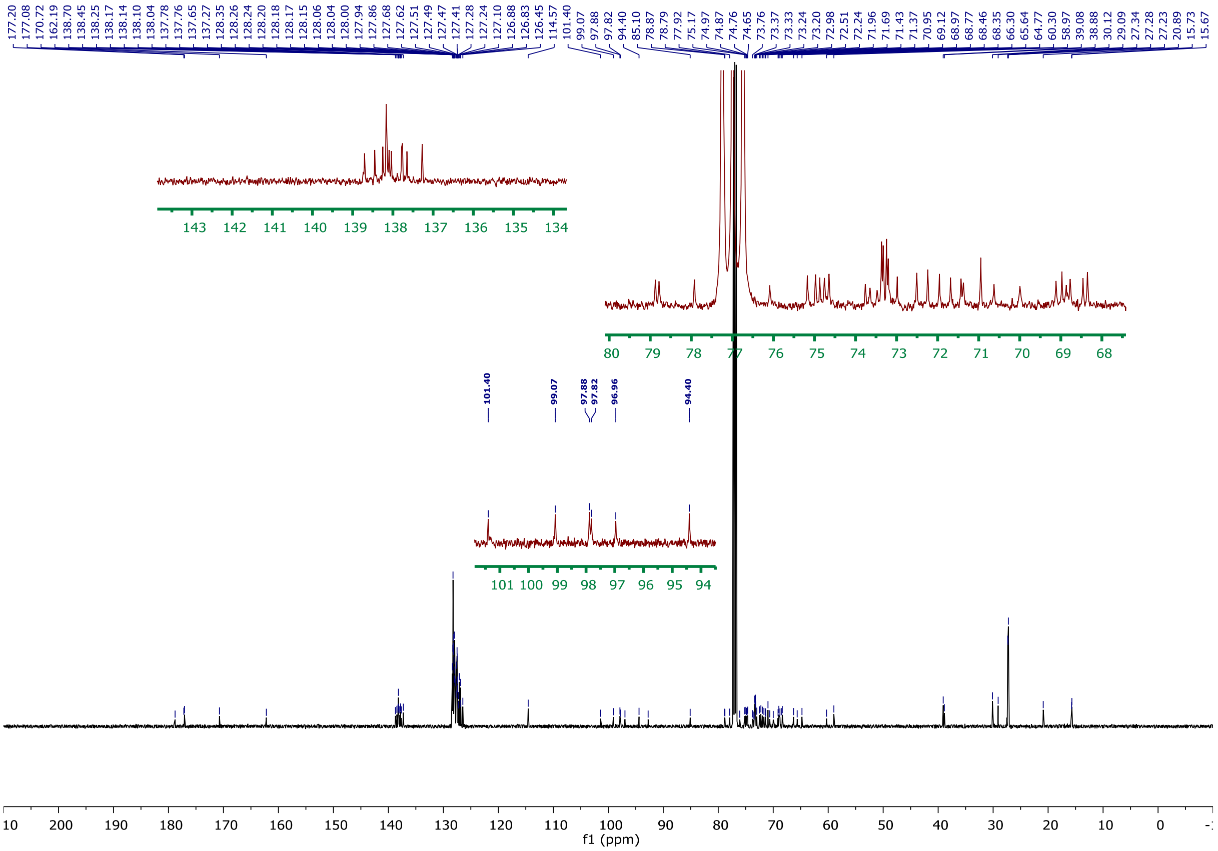

Figure S48. ^{13}C NMR ($CDCl_3$, 125 MHz) spectrum of **hexasaccharide 47**

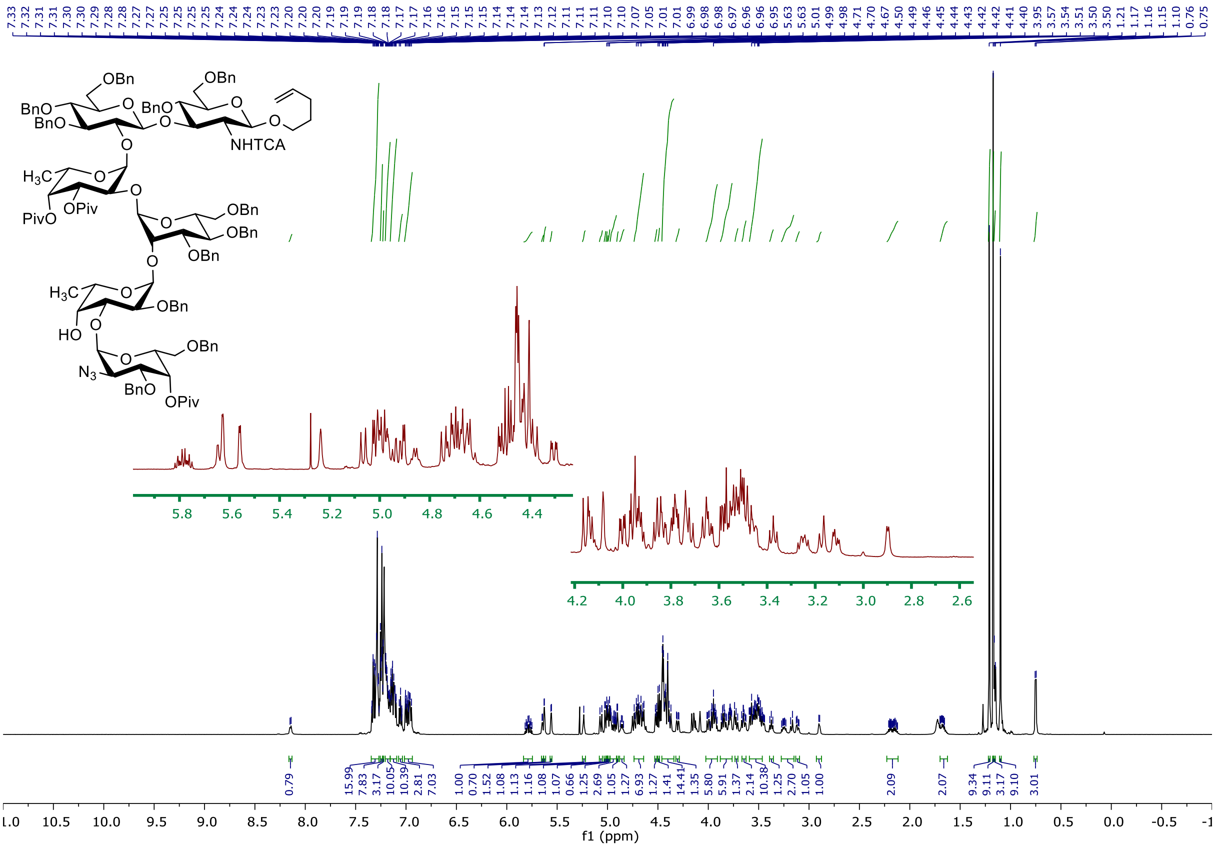

Figure S49. ^{1}H NMR ($CDCl_3$, 600 MHz) spectrum of **hexasaccharide acceptor 48**

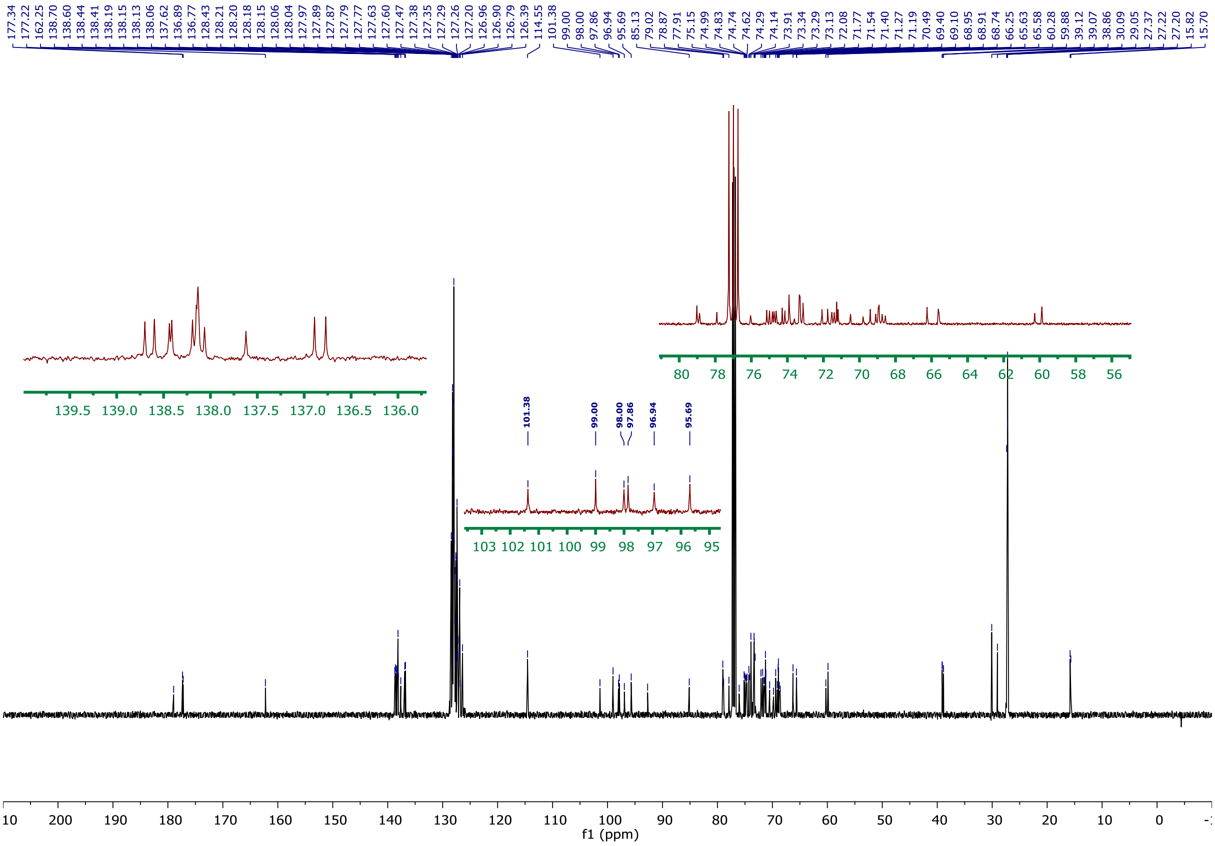

Figure S50. ^{13}C NMR ($CDCl_3$, 125 MHz) spectrum of **hexasaccharide acceptor 48**

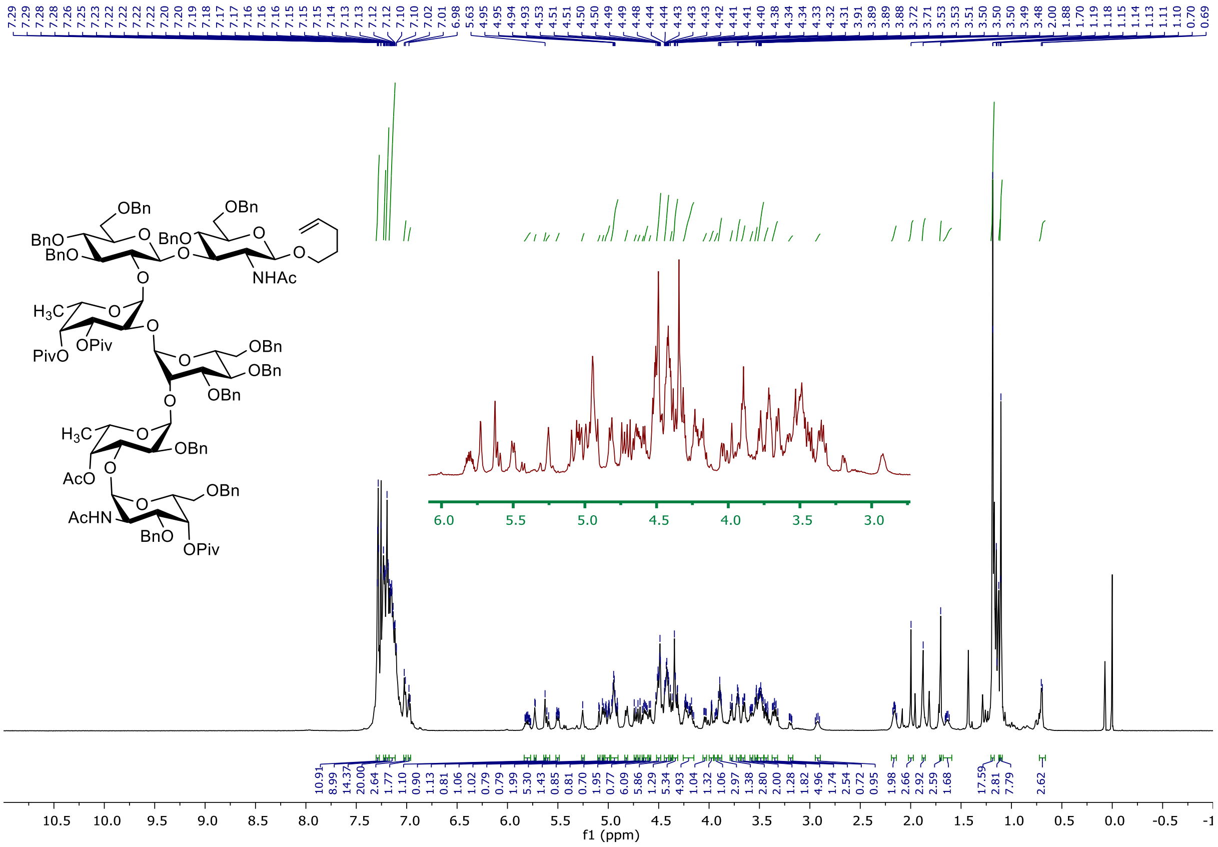

Figure S51. ^{1}H NMR ($CDCl_3$, 600 MHz) spectrum of **hexasaccharide 52**

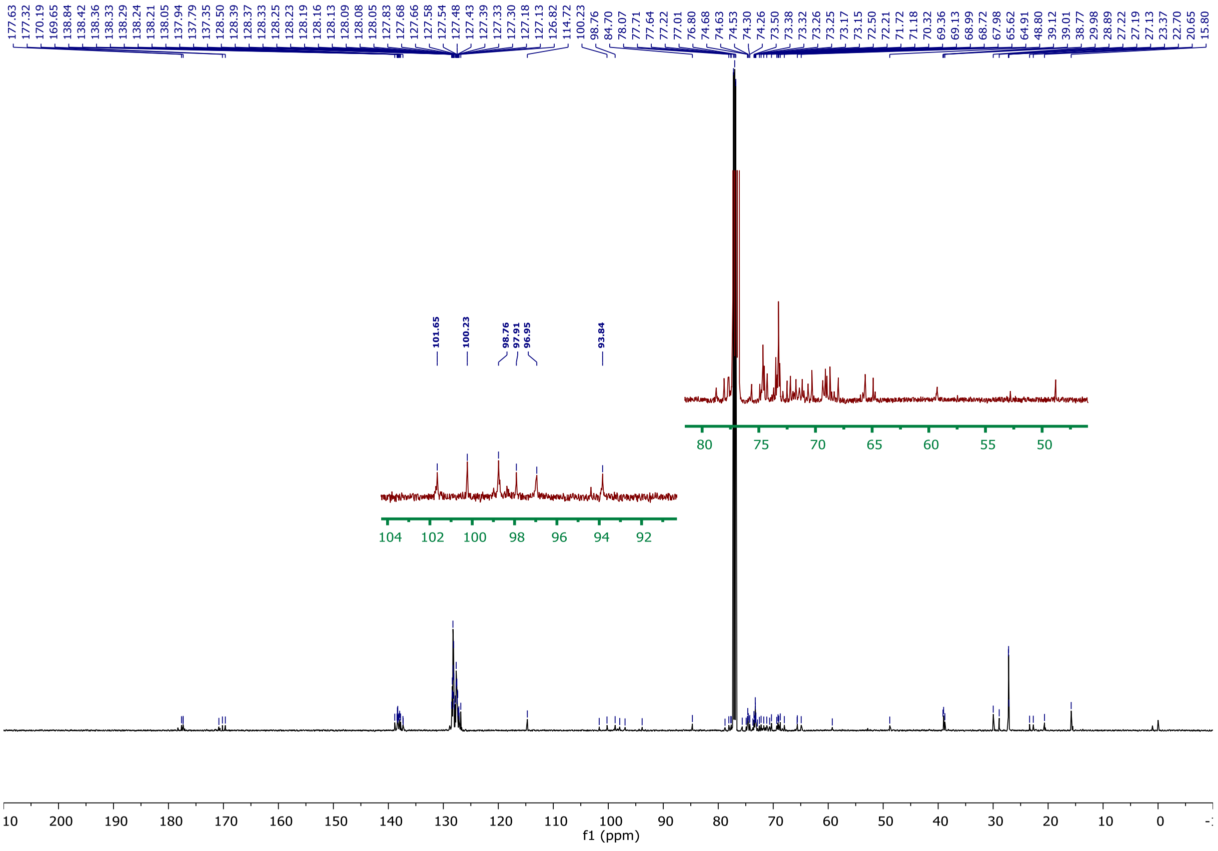

Figure S52. ^{13}C NMR ($CDCl_3$, 125 MHz) spectrum of **hexasaccharide 52**

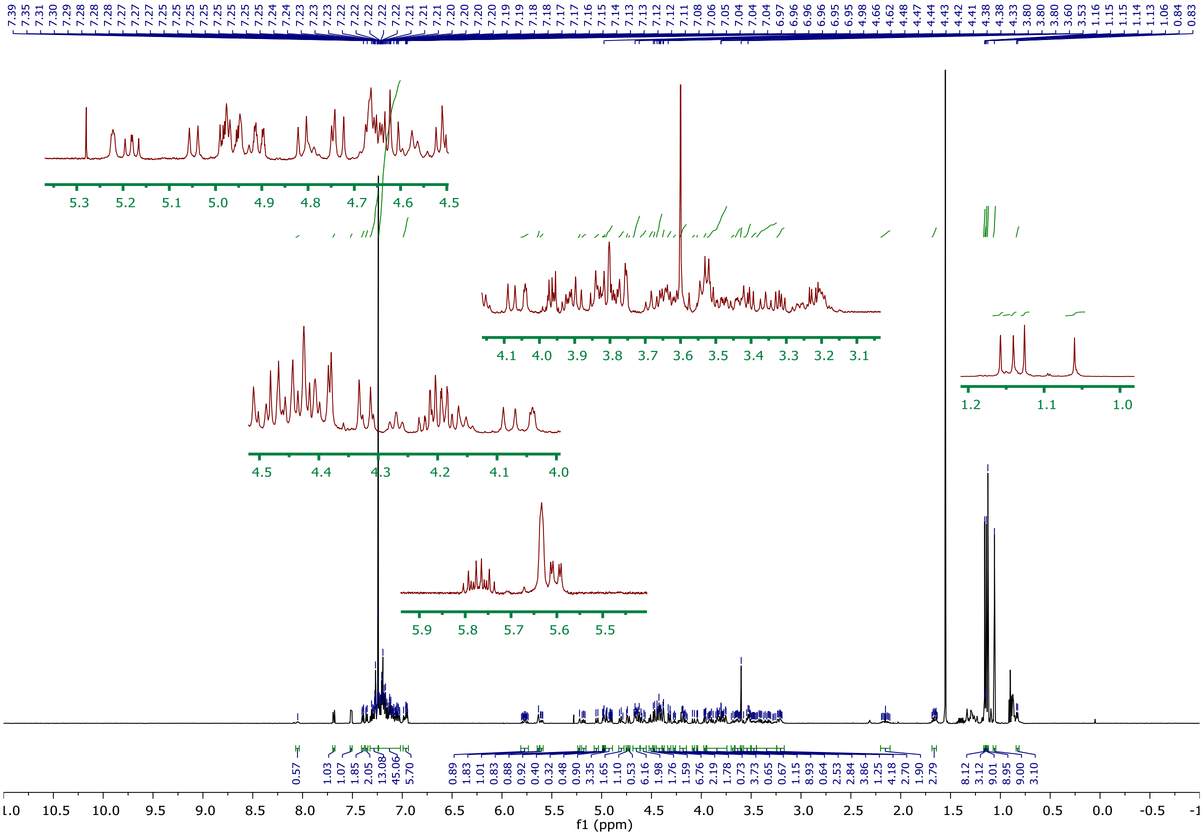

Figure S53. ^{1}H NMR ($CDCl_3$, 125 MHz) spectrum of **heptasaccharide 49**

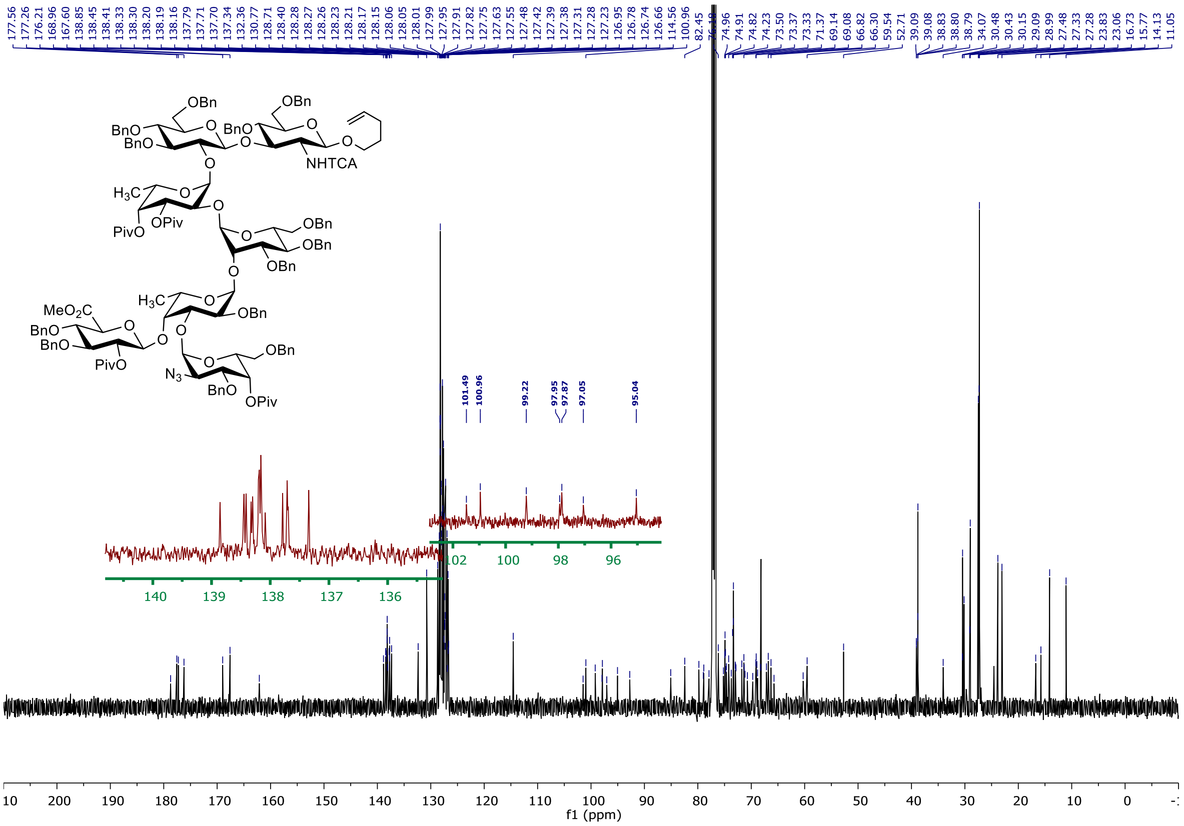

Figure S54. ^{13}C NMR ($CDCl_3$, 125 MHz) spectrum of spectrum of **heptasaccharide 49**

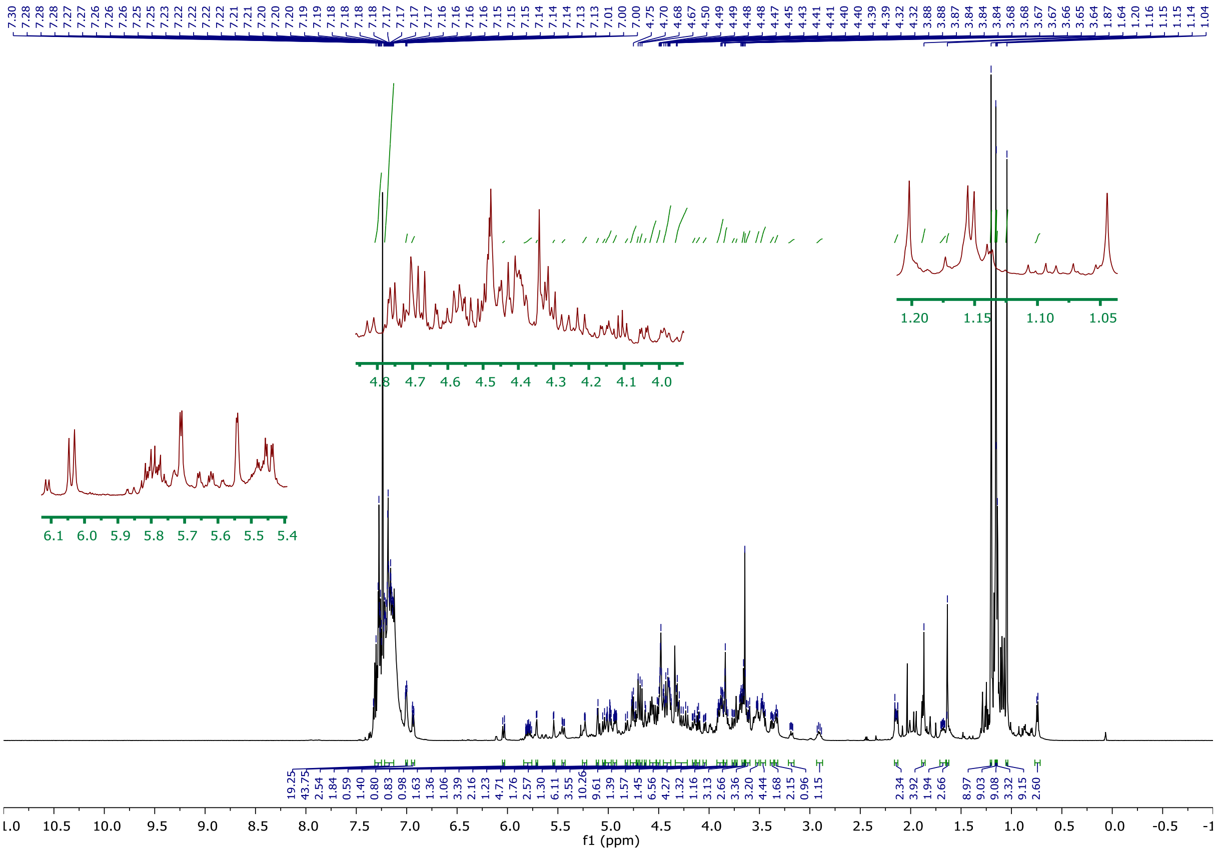

Figure S55. ^{1}H NMR ($CDCl_3$, 125 MHz) spectrum of **heptasaccharide 54**

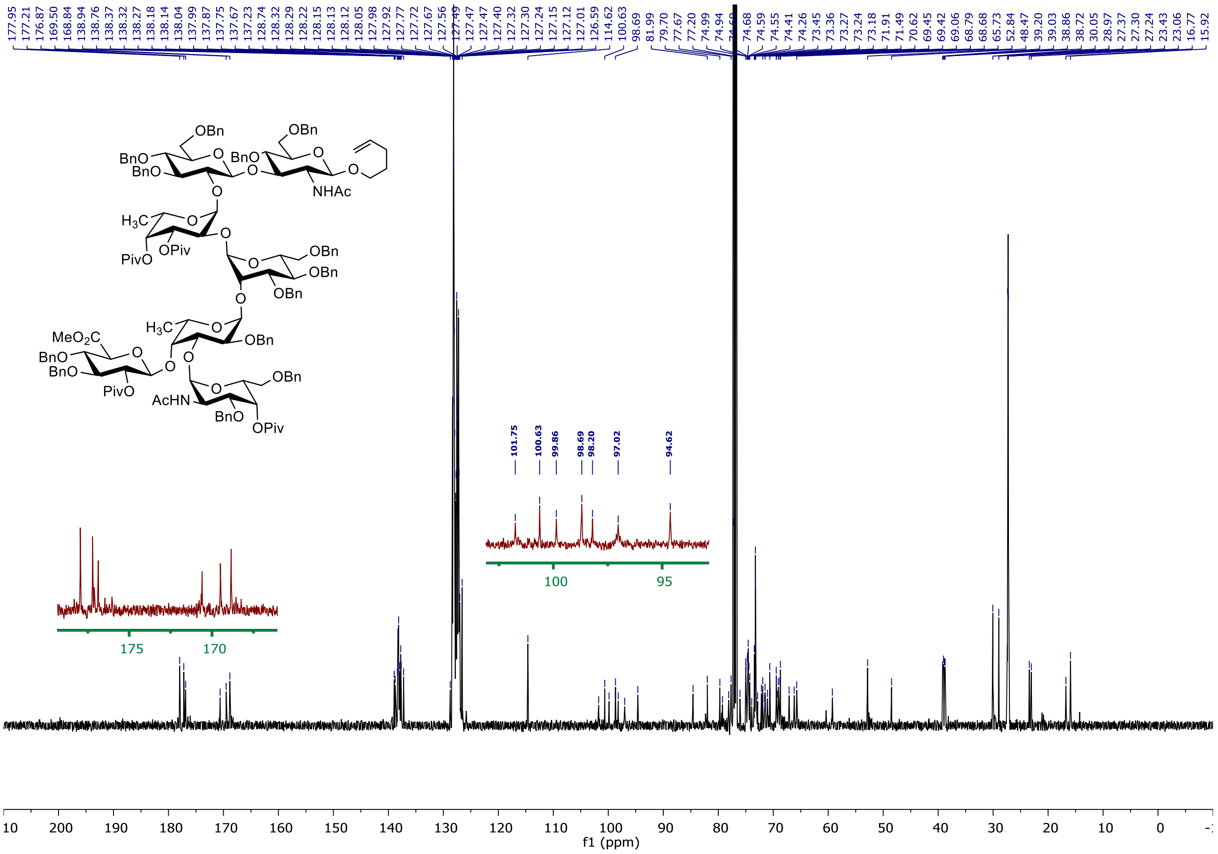

Figure S56. ^{13}C NMR ($CDCl_3$, 125 MHz) spectrum of **heptasaccharide 54**

8. Reactions of deprotection by Birch reaction

8.1. *Synthesis of the deprotected trisaccharide 51*

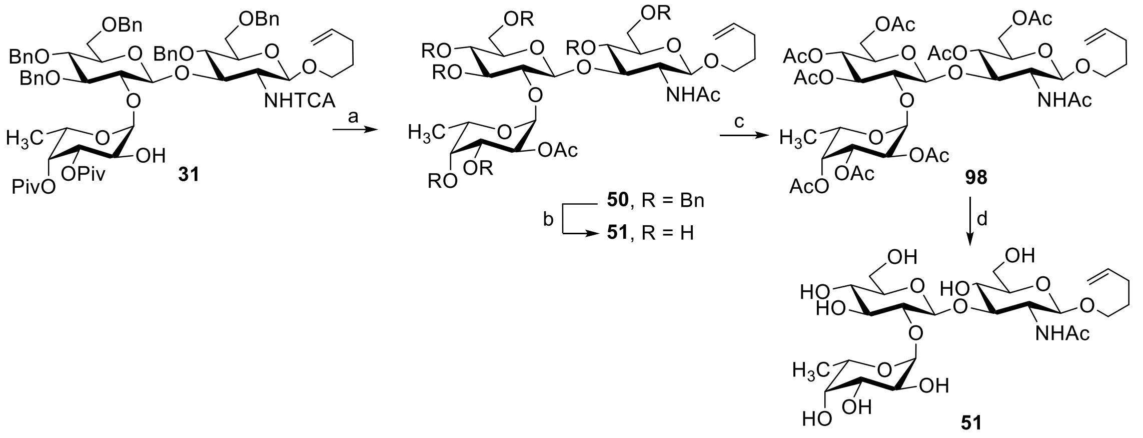

a) Zn/ Cu Couple, AcOH, 1 d, 45 °C, Then Ac_2O, Py, DMAP, overnight, 25 ºC b) Na, NH_3, THF **c)** Ac_2O, Py, cat. DMAP **d)** NaOMe (30%), MeOH

8.2. *Synthesis of the deprotected hexasaccharide 53*

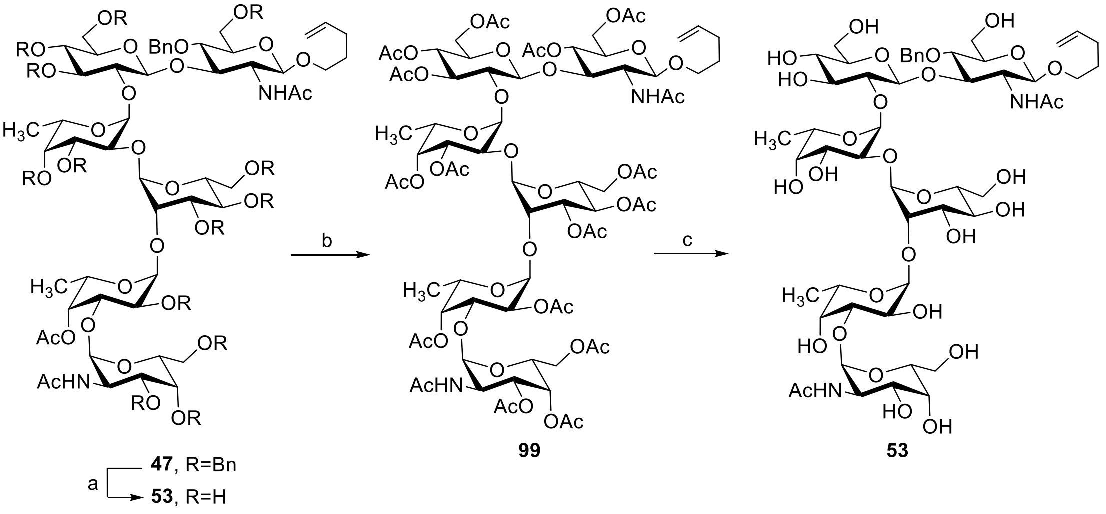

a) Na, NH_3, THF **b)** Ac_2O, py, cat. DMAP **c)** NaOMe (30%), MeOH

8.3. *Synthesis of the deprotected heptasaccharide 1*

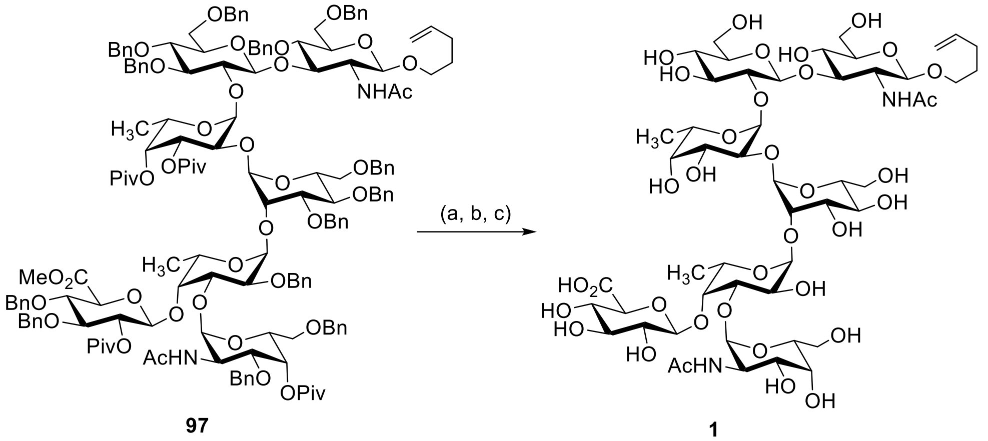

a) KOH (3 M), MeOH **b)** Na, NH_3, THF **c)** Ac_2O, py, cat. DMAP **d)** NaOMe (30%), MeOH

9. Spectroscopic data of synthesized final compounds after Birch reaction

9.1. *Synthesis of the deprotected trisaccharide 81*

***n*-Pentenyl (2-*O*-acetyl-3,4-di-*O*-pivaloyl-α-L-fucopyranosyl)-(1→2)-(3,4,6-tri-*O*-benzyl-β-D-glucopyranosyl)-(1→3)-4,6-di-*O*-benzyl-2-deoxy-2-acetamido-β-D-glucopyranoside (50)**

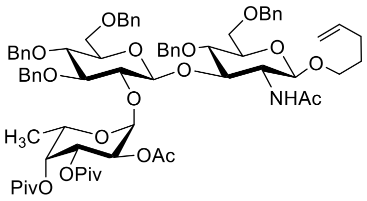

Compound **31** (35 mg, 26.5 µmol) was dissolved in AcOH (3 mL). Zn/Cu couple (330 mg) was added and the reaction mixture was stirred at 45 °C for 1 d. Afterwards it was cooled to room temperature, filtered through Celite and concentrated in vacuum. The residue was further dissolved in pyridine (2.5 mL), Ac_2O (1.2 mL) and a catalytic amount of DMAP was added. The reaction mixture was stirred overnight concentrated and co-evaporated with toluene. Purification by silica gel column chromatography (pentane / EtOAc, 8:1 → 2:1) furnished 25 mg (75%) of **50** as a colorless oil.

^{1}H NMR (300 MHz, $CDCl_3$): δ = 1.11 (s, 9 H), 1.14 (d, J = 6.5 Hz, 3 H), 1.30 (s, 9 H), 1.67 (m_c, 2 H), 1.88 (s, 3 H), 1.95 (s, 3 H), 2.17 (m, 2 H), 2.95 (dt, J = 10.2, 7.6 Hz, 1 H), 3.39 (t, J = 8.7 Hz, 1 H), 3.45–3.50 (m, 2 H), 3.56-3.78 (m, 7H), 3.90 (dt, J = 9.6, 5.8 Hz, 1 H), 4.32 (d, J = 12.1 Hz, 1 H), 4.41 (d, J = 12.1 Hz, 1 H), 4.46 (d, J = 11.2 Hz, 1 H), 4.52-4.59 (m, 3 H), 4.66 (d, J = 3.1 Hz, 1 H), 4.70 (d, J = 3.0, 1.4 Hz, 1 H), 4.74 (dd, J = 6.6, 1.4 Hz, 1 H), 4.86–4.92 (m, 2 H), 4.93–5.01 (m, 2 H), 5.01–5.08 (m, 1 H), 5.27 (dd, J = 3.1, 1.3 Hz, 1 H), 5.33 (dd, J = 10.8, 3.4 Hz, 1 H), 5.53 (dd, J = 10.9, 3.1 Hz, 1 H), 5.64 (d, J = 3.3 Hz, 1 H), 5.82 (m_c, 1 H), 7.01 (d, J = 6.9 Hz, 1 H), 7.08-7.11 (m, 2 H), 7.16-7.25 (m, 16 H), 7.28-7.32 (m, 6 H).

^{13}C NMR (75 MHz, $CDCl_3$): δ = 15.7, 20.5, 23.2, 26.9, 27.3, 28.8, 29.9, 38.8, 39.1, 59.4, 60.4, 65.6, 67.9, 68.0, 68.8, 69.3, 71.2, 73.3, 73.3, 74.6, 74.6, 74.9, 75.0, 77.9, 78.5, 85.9, 95.7, 98.8, 101.6, 114.8, 127.1, 127.3, 127.4, 127.4, 127.6, 127.6, 127.7, 128.1, 128.1, 128.3, 128.3, 128.4, 137.9, 137.9, 138.2, 138.3, 138.3, 138.7, 169.6, 171.5, 177.4, 178.3.

IR (ATR): $\tilde{\nu}$ (cm^{-1}) = 3063, 3030, 2970, 2870, 1734, 1679, 1605, 1529, 1496.

HR-MS (ESI): m/z calcd. for $C_{72}H_{91}NO_{18}Na^+$ 1280.6128, found 1280.6138.

***n*-Pentenyl (2,3,4-tri-*O*-acetyl-α-L-fucopyranosyl)-(1→2)-(3,4,6-tri-*O*-acetyl-β-D-glucopyranosyl)-(1→3)-4,6-di-*O*-acetyl-2-deoxy-2-acetamido-β-D-glucopyranoside (98)**

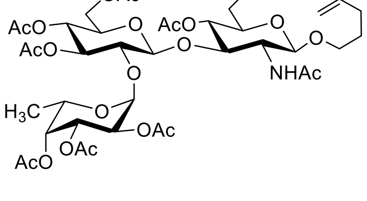

About 15 mL of NH_3 were condensed into the flask at -70 °C. Tiny pieces of sodium were added whereby the color of the solution turned to blue. Compound **50** (25 mg, 20 µmol) was dissolved in THF (4 mL). This solution was added dropwise to ammonia solution. After 30 min, some drops of methanol were added until the blue color

disappeared. Again, tiny pieces of sodium were added and the solution turned blue. After 30 min, methanol was added to quench the reaction. The cooling bath was removed and the ammonia was evaporated by a stream of N_2 bubbling through the solution. To neutralize the basic solution, Amberlite acidic resin (IR-120) was added until pH 7 was reached. The solution was filtered, washed several times with methanol and concentrated. The residue was further dissolved in pyridine (2 mL), Ac_2O (1 mL) and a catalytic amount of DMAP was added. The reaction mixture was stirred overnight. After filtration through a plug of silica, purification was performed by recycling HPLC (gel permeation chromatography) to obtain 12 mg (65%) trisaccharide **98** as a colorless powder.

^{1}H NMR (600 MHz, $CDCl_3$): δ = 1.18 (d, J = 6.4 Hz, 3 H), 1.67 (m_c, 2 H), 1.97 (s, 3 H), 1.98 (s, 3 H), 2.00 (s, 3 H), 2.00 (s, 3 H), 2.02 (s, 3 H), 2.04 (s, 3 H), 2.05 (s, 3 H), 2.06 (s, 3 H), 2.17 (s, 3 H), 2.14-2.22 (m, 2 H), 2.70 (ddd, J = 10.6, 8.1, 6.5 Hz, 1 H), 3.53 (ddd, J = 9.7, 7.5, 5.9 Hz, 1 H), 3.65-3.673 (m, 3H), 3.77 (dd, J = 9.5, 7.9 Hz, 1 H), 3.90 (dt, J = 9.7, 3.9 Hz, 1 H) 4.01 (dd, J = 12.6, 2.1 Hz, 1 H), 4.12 (dd, J = 12.1, 2.3 Hz, 1 H), 4.26 (dd, J = 12.2, 4.9 Hz, 1 H), 4.40 (dd, J = 12.5, 3.8 Hz, 1 H), 4.44 (d, J = 7.9 Hz, 1 H), 4.65 (q, J = 6.6, Hz, 1 H), 4.83 (t, J = 9.7, Hz, 1 H), 4.93-4.99 (m, 5 H), 5.06 (dt, J = 17.2, 1.7 Hz, 1 H), 5.18 (t, J = 9.5 Hz, 1 H), 5.22 (dd, J = 3.2, 1.5 Hz, 1 H), 5.39 (d, J = 3.4 Hz,1 H), 5.43 (dd, J = 11.0, 3.1 Hz,1 H), 5.82 (m_c,1 H), 7.05 (d, J = 6.5 Hz, 1 H).

^{13}C NMR (125 MHz, $CDCl_3$): δ = 15.3, 20.5, 20.5, 20.6, 20.6, 20.7, 20.8, 20.8, 23.2, 28.7, 29.9, 59.8, 61.5, 62.4, 65.1, 67.4, 68.1, 68.2, 68.8, 69.5, 71.3, 71.4, 71.6, 71.7, 75.4, 75.7, 94.3, 98.7, 100.9, 114.9, 138.1, 169.5, 169.5, 170.5, 170.5, 170.6, 170.8, 171.4, 172.5.

IR (ATR): $\tilde{\nu}$ (cm^{-1}) = 2949, 2140, 1677, 1534, 1436, 1372, 1049.

HR-MS (ESI): m/z calcd. for $C_{41}H_{59}NO_{23}Na^+$ 956.3375, found. 956.3375.

n-Pentenyl (2,3,4-Tri-hydroxy-α-L-fucopyranosyl)-(1→2)-(3,4,6-tri-hydroxy-β-D-glucopyranosyl)-(1→3)-4,6-di-hydroxy-2-deoxy-2-acetamido-β-D-glucopyranoside (51)

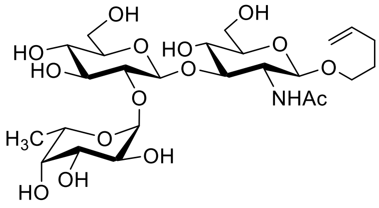

Compound **82** (10 mg, 11 µmol) was dissolved in MeOH (400 µL). A solution of sodium methoxide (30%, 300 µL) was added until pH 12 was reached. The reaction mixture was stirred for overnight. The solution was neutralized with Amberlite IR-120 acidic resin, filtered and washed carefully. A dialysis was performed for 4 days. H_2O was evaporated by lyophilizer to obtain 5.2 mg (80%) deprotected trisaccharie **51** as a colorless powder.

$[\alpha]_D^{20}$= -89.1° (c = 0.24, CH_3OH).

^{1}H NMR (600 MHz, DMSO-d_6): δ = 1.02 (d, J = 6.4 Hz, 3 H), 1.52 (m_c, 2 H), 1.80 (s, 3 H), 2.1 (m_c, 2 H), 3.11–3.19 (m, 4 H), 3.48-3.51 (m, 3H), 3.59 (dd, J = 17.8, 9.6 Hz, 1 H), 3.64-3.71 (m, 3 H), 3.73 (dt, J = 10.2, 6.1 Hz, 1 H), 4.06 (q, J = 6.5 Hz, 1 H), 4.17-4.20 (m, 2 H), 4.29-4.33 (m, 2 H), 4.42 (d, J = 2.9 Hz, 1 H), 4.46 (d, J = 7.7, Hz, 1 H), 4.58-4.63 (m, 1 H), 4.94-4.99 (m, 1 H), 5.02 (q, J = 1.8 Hz, 1 H), 5.05-5.06 (m, 1 H), 5.79 (m_c, 1 H), 7.75 (d, J = 9.1 Hz, 1 H).

^{13}C NMR (125 MHz, DMSO-d_6): δ = 16.3, 20.9, 22.9, 28.1, 29.1, 53.8, 60.8, 65.9, 67.6, 68.2, 68.7, 69.5, 69.7, 71.4, 76.8, 76.9, 77.0, 78.7, 80.2, 99.9, 99.9, 101.6, 114.9, 138.2, 168.7.

IR (ATR): $\tilde{\nu}$ (cm^{-1}) = 3311, 2926, 2882, 1653, 1553, 1133, 1072, 1023, 671.

HR-MS (ESI): m/z calcd. for $C_{25}H_{43}NO_{15}Na^+$ 620.2524, found. 620.2527.

9.2. *Synthesis of the deprotected hexasaccharide (83)*

n-Pentenyl (2-acetamido-3,4,6-tri-*O*-acetyl-2-deoxy-α-D-galactopyranosyl)-(1→3)-(2,4-di-*O*-acetyl-α-L-fucopyranosyl)-(1→2)-(3,4,6-tri-*O*-acetyl-α-D-manno-pyranosyl)-(1→2)-(3,4-di-*O*-acetyl-β-L-fucopyranosyl)-(1→2)-(3,4,6-tri-*O*-acetyl-β-D-glucopyranosyl)-(1→3)-4,6-di-*O*-acetyl-2-deoxy-2-acetamido-β-D-glucopyranoside (99)

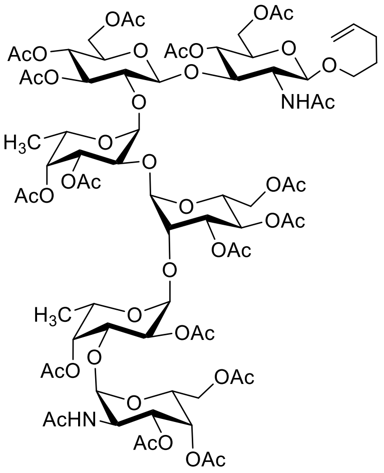

About 20 mL of NH_3 were condensed into the flask at -70 °C. Tiny pieces of sodium were added whereby the color of the solution turned to blue. Compound **47** (25 mg, 10 µmol) was dissolved in THF (6 mL). This solution was added dropwise to ammonia solution. After 30 min, some drops of methanol were added until the blue color disappeared. Again, tiny pieces of sodium were added and the solution turned blue. After 30 min, methanol was added to quench the reaction. The cooling bath was removed and the ammonia was evaporated by a stream of N_2 bubbling through the solution. To neutralize the basic solution, Amberlite acidic resin (IR-120) was added until pH 7 was reached. The solution was filtered, washed several times with methanol and concentrated. The residue was further dissolved in pyridine (2 mL), Ac_2O (1 mL) and a catalytic amount of DMAP was added. The reaction mixture was stirred overnight. After filtration through a plug of silica, purification was performed by recycling HPLC (gel permeation chromatography) to obtain 10.4 mg (60%) hexasaccharide **99** as a pale yellow powder.

^{1}H NMR (600 MHz, $CDCl_3$): δ = 1.10 (d, J = 6.5 Hz, 3 H), 1.18 (d, J = 6.5 Hz, 3 H), 1.71 (m$_c$, 2 H), 1.91 (s, 3 H), 1.96 (s, 3 H), 1.98 (s, 3H), 2.01 (s, 3 H), 2.01 (s, 3 H), 2.01 (s, 6 H), 2.05 (s, 3 H), 2.06 (s, 3 H), 2.06 (s, 6 H), 2.08 (s, 3 H), 2.08 (s, 3 H), 2.09 (s, 3 H), 2.17 (s, 3 H), 2.17-2.21 (m, 2 H), 2.20 (s, 3 H), 2.24 (s, 3 H), 3.79 (ddd, J = 10.7, 8.1, 6.3 Hz, 1 H), 3.53 (ddd, J = 9.9, 7.3, 6 Hz, 1 H), 3.69-3.72 (m, 2 H), 3.82 (dd, J = 9.0, 7.9 Hz, 1 H), 3.84-3.90 (m, 2 H), 3.93 (dd, J = 3.2, 1.9 Hz, 1 H), 3.99-4.03 (m, 3 H), 4.09-4.15 (m, 3 H), 4.22-4.24 (m, 3 H), 4.36 (dd, J = 12.5, 4.0 Hz, 1 H), 4.43-4.49 (m, 4 H), 4.59 (q, J = 6.2, 5.6 Hz, 1 H), 4.66 (d, J = 1.8 Hz, 1 H), 4.83 (t, J = 9.7 Hz, 1 H), 4.91 (dd, J = 10.2, 3.7 Hz, 1 H), 4.96-5.07 (m, 6 H), 5.08 (d, J = 3.2 Hz, 1 H), 5.20-5.21 (m, 4 H), 5.27 (d, J = 2.5 Hz, 1 H), 5.34 (t, J = 10.2 Hz, 1 H), 5.39 (dd, J = 3.3, 1.4 Hz, 1 H), 5.55 (d, J = 9.1 Hz, 1 H), 5.81 (m$_c$, 1 H), 7.15 (d, J = 6.7 Hz, 1 H).

^{13}C NMR (125 MHz, $CDCl_3$): δ = 15.5, 15.9, 20.5, 20.6, 20.6, 20.6, 20.6, 20.7, 20.7, 20.7, 20.8, 20.8, 20.9, 23.1, 23.5, 28.8, 29.9, 48.6, 59.7, 61.6, 61.6, 61.9, 62.4, 65.2, 65.6, 67.4, 67.5, 67.5, 68.5, 68.8, 69.4, 69.4, 69.5, 70.1, 70.9, 71.1, 71.3, 71.6, 71.6, 72.1, 73.5, 74.0, 94.6, 95.9, 98.5, 98.6, 98.9, 101.1, 114.9, 138.1, 169.3, 169.4, 169.6, 169.7, 169.8, 169.8, 170.2, 170.3, 170.4, 170.4, 170.5, 170.8, 170.9, 171.2, 171.2, 171.4, 172.4.

IR (ATR): $\tilde{\nu}$ (cm^{-1}) = 2949, 1746, 1677, 1534, 1436, 1372, 1049.

HR-MS (ESI): m/z calcd. for $C_{75}H_{106}N_2O_{44}Na^+$ 1761.6010, found. 1761.6025.

n-Pentenyl (2-acetamido-3,4,6-tri-hydroxy-2-deoxy-α-D-galactopyranosyl)-(1→3)-(2,4-di-hydroxy-α-L-fucopyranosyl)-(1→2)-(3,4,6-hydroxy-α-D-manno-pyranosyl)-(1→2)-(3,4-di-hydroxy-β-L-fucopyranosyl)-(1→2)-(3,4,6-tri-hydroxy-β-D-glucopyranosyl)-(1→3)-4,6-di-hydroxy-2-deoxy-2-acetamido-β-D-glucopyranoside (53)

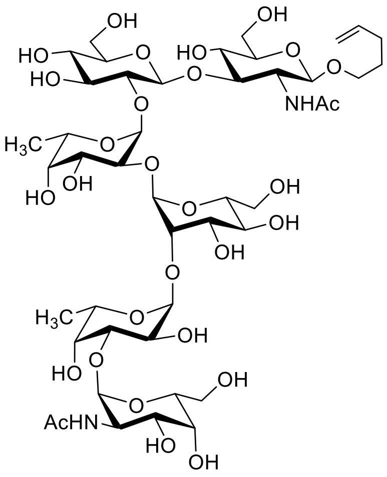

Compound **99** (10.4 mg, 6 μmol) was dissolved in MeOH (800 μL). A solution of sodium methoxide (30%, 300 μL) was added until pH 12 was reached. The reaction mixture was stirred for 1 day. The solution was neutralized with Amberlite IR-120 acidic resin, filtered and washed carefully. A dialysis was performed for 6 days. H_2O was evaporated by lyophilizer to obtain 4.8 mg (72%) hexasaccharide **53** as a colorless powder.

$[\alpha]_D^{20}$= - 29.4° (c = 0.26, CH_3OH).

^{1}H NMR (600 MHz, DMSO-d_6): δ = 1.01 (d, *J* = 6.4 Hz, 3 H), 1.07 (d, *J* = 6.5 Hz, 3 H), 1.53 (m_c, 2 H), 1.83 (s, 3 H), 1.85 (s, 3 H), 2.04 (m_c, 2 H), 3.12 (dd, *J* = 9.4 Hz, 1 H), 3.15 (s_{br}, 1 H), 3.19-3.22 (m, 2 H), 3.24 (t, *J* = 8.4 Hz, 1 H), 3.46-3.52 (m, 5 H), 3.60-3.66 (m, 5 H), 3.68-3.75 (m, 8 H), 3.80 (d, *J* = 3.0 Hz, 1 H), 3.94 (t, *J* = 6.3 Hz, 1 H), 4.03 (d, *J* = 7.3 Hz, 1 H), 4.07-4.11 (m, 2 H), 4.15 (d, *J* = 8.0 Hz, 1 H), 4.26 (d, *J* = 7.0 Hz, 1 H), 4.29 (d, *J* = 4.8 Hz, 1 H), 4.33 (d, *J* = 4.7 Hz, 1 H), 4.38 (t, *J* = 5.6 Hz, 1 H), 4.42 (d, *J* = 6.2 Hz, 1 H), 4.47 (d, *J* = 7.9 Hz, 1 H), 4.50-4.52 (m, 3 H), 4.57 (d, *J* = 6.8 Hz, 1 H), 4.60-4.62 (m, 1 H), 4.63-4.65 (m, 1 H), 4.68 (d, *J* = 7.0 Hz, 1 H), 4.72-4.75 (m, 2 H), 4.79 (d, *J* = 4.8 Hz, 1 H), 4.88 (d, *J* = 4.8 Hz, 1 H), 4.97 (d, *J* = 10.1 Hz, 1 H), 5.02 (d, *J* = 17.9 Hz, 1 H), 5.08 (s, 1 H), 5.19 (d, *J* = 3.7 Hz, 1 H), 5.24 (d, *J* = 5.6 Hz, 1 H), 5.81 (m_c, 1 H), 7.34 (d, *J* = 9.2 Hz, 1 H), 7.76 (d, *J* = 8.9 Hz, 1 H).

^{13}C NMR (125 MHz, DMSO-d_6): δ = 16.1, 16.3, 22.9, 22.9, 28.1, 29.3, 49.9, 53.6, 60.6, 60.8, 60.8, 61.1, 65.5, 65.7, 66.9, 67.2, 67.6, 68.0, 68.3, 68.4, 68.8, 69.7, 70.2, 70.8, 71.4, 71.7, 73.1, 73.9, 75.1, 76.2, 76.5, 76.9, 77.4, 78.6, 80.6, 96.8, 97.5, 98.0, 99.4, 100.2, 101.8, 114.9, 138.2, 168.8, 169.8.

^{1}H NMR and ^{13}C NMR were measured in D_2O and data was reported below.

^{1}H NMR (600 MHz, D_2O): δ = 1.20 (d, J = 6.6 Hz, 3 H), 1.23 (d, J = 6.6 Hz, 3 H), 1.63 (m_c, 2 H), 2.05 (s, 3 H), 2.06-2.09 (m_c, 2 H), 2.08 (s, 3 H), 2.73 (s, 1 H), 3.36-3.40 (m, 1 H), 3.44-3.49 (m, 4 H),3.56 (dt, J = 10.3, 6.4 Hz, 1 H), 3.66 (t, J = 9.3 Hz, 1 H), 3.72 (d, J = 6 Hz, 1 H), 3.74-3.84 (m, 10 H), 3.90-4.02 (m, 11 H), 4.06 (d, J = 3.0 Hz, 1 H), 4.11 (t, J = 6.3 Hz, 1 H), 4.20 (dd, J = 10.9, 3.9 Hz, 1 H), 4.36 (q, J = 6.7 Hz, 1 H), 4.40-4.43 (m, 2 H), 4.68 (d, J = 7.7 Hz, 1 H), 4.95 (d, J = 3.9 Hz, 1 H), 5.03 (d, J = 10.2 Hz, 1 H), 5.07 (d, J = 17.2 Hz, 1 H), 5.15 (d, J = 3.9 Hz, 1 H), 5.21 (s, 1 H), 5.23 (d, J = 3.8 Hz, 1 H), 5.89 (m_c, 1 H).

^{13}C NMR (125 MHz, D_2O): δ = 17.9, 18.0, 24.9, 25.2, 30.8, 32.2, 41.6, 52.9, 57.6, 63.1, 63.5, 63.6, 64.2, 69.1, 69.7, 69.9, 70.2, 70.5, 71.4, 72.2, 72.6, 73.1, 74.3, 74.7, 75.1, 76.1, 76.3, 78.3, 78.6, 79.1, 79.4, 80.5, 80.6, 80.9, 101.1, 101.6, 102.1, 102.4, 102.9, 104.7, 117.7, 141.6, 176.7, 177.5.

IR (ATR): $\tilde{\nu}$ (cm^{-1}) = 3322, 2924, 1650, 1552, 1413, 1373, 1314, 1070, 1025, 615.

HR-MS (ESI): m/z calcd. for $C_{45}H_{76}N_2O_{29}Na^+$ 1131.4431, found. 1131.4430

n-Pentenyl (2-acetamido-3,4,6-tri-hydroxy-2-deoxy-α-D-galactopyranosyl)-(1→3)-[(5-carboxylicacid-2,3,4-tri-hydroxy-β-D-glucopyranosyluronate)-(1→4)]-(2-hydroxy-α-L-fucopyranosyl)-(1→2)-(3,4,6-tri-hydroxy-α-D-mannopyranosyl)-(1→2)-(3,4-di-hydroxy-α-L-fucopyranosyl)-(1→2)-(3,4,6-tri-hydroxy-β-D-glucopyranosyl)-(1→3)-2-acetamido-4,6-di-hydroxy-2-deoxy-β-D-glucopyranoside (1)

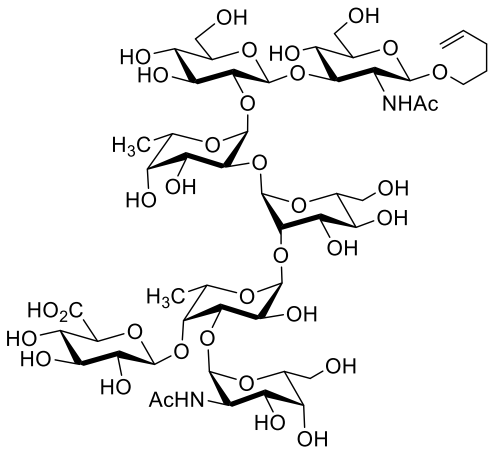

Compound **97** (10 mg, 3.5 μmol) was dissolved in MeOH (400 μL). A solution of potassium hydroxide (3 M, 120 μL) was added. The reaction mixture was stirred for 1 day at 25 ºC. The solution was neutralized with hydrogen chloride (1 M) carefully, and concentrated in *vacuo*. The resulting crude product was purified by sephadex LH20.to afford 2.9 mg (29%) of corresponding carboxylic acid as a colorless oil.

About 5 mL of NH_3 were condensed into the flask at -70 °C. Tiny pieces of sodium were added whereby the color of the solution turned to blue. Compound 100 (5 mg, 1.8 µmol) was dissolved in THF (2 mL). This solution was added dropwise to ammonia solution. After 30 min, some drops of methanol were added until the blue color disappeared. Again, tiny pieces of sodium were added and the solution turned blue. After 30 min, methanol was added to quench the reaction. The cooling bath was removed and the ammonia was evaporated by a stream of N2 bubbling through the solution. To neutralize the basic solution, Amberlite acidic resin (IR-120) was added until pH 7 was reached. The solution was filtered, washed several times with methanol and concentrated. The residue was further dissolved in pyridine (600 μl), Ac_2O (400 μl) and a catalytic amount of DMAP was added. The reaction

mixture was stirred 24 h. After filtration through a plug of silica, purification was performed by recycling HPLC (gel permeation chromatography) to obtain 2.69 mg (75%)

Compound **1** (2.69 mg, 1.34 μmol) was dissolved in MeOH (200 μL). A solution of sodium methoxide (30%, 100 μL) was added until pH 12 was reached. The reaction mixture was stirred for 1 day. The solution was neutralized with Amberlite IR-120 acidic resin, filtered and washed carefully. A dialysis was performed for 6 days. H_2O was evaporated by lyophilizer to obtain 1.4 mg (80%) hexasaccharide **1** as colorless powder.

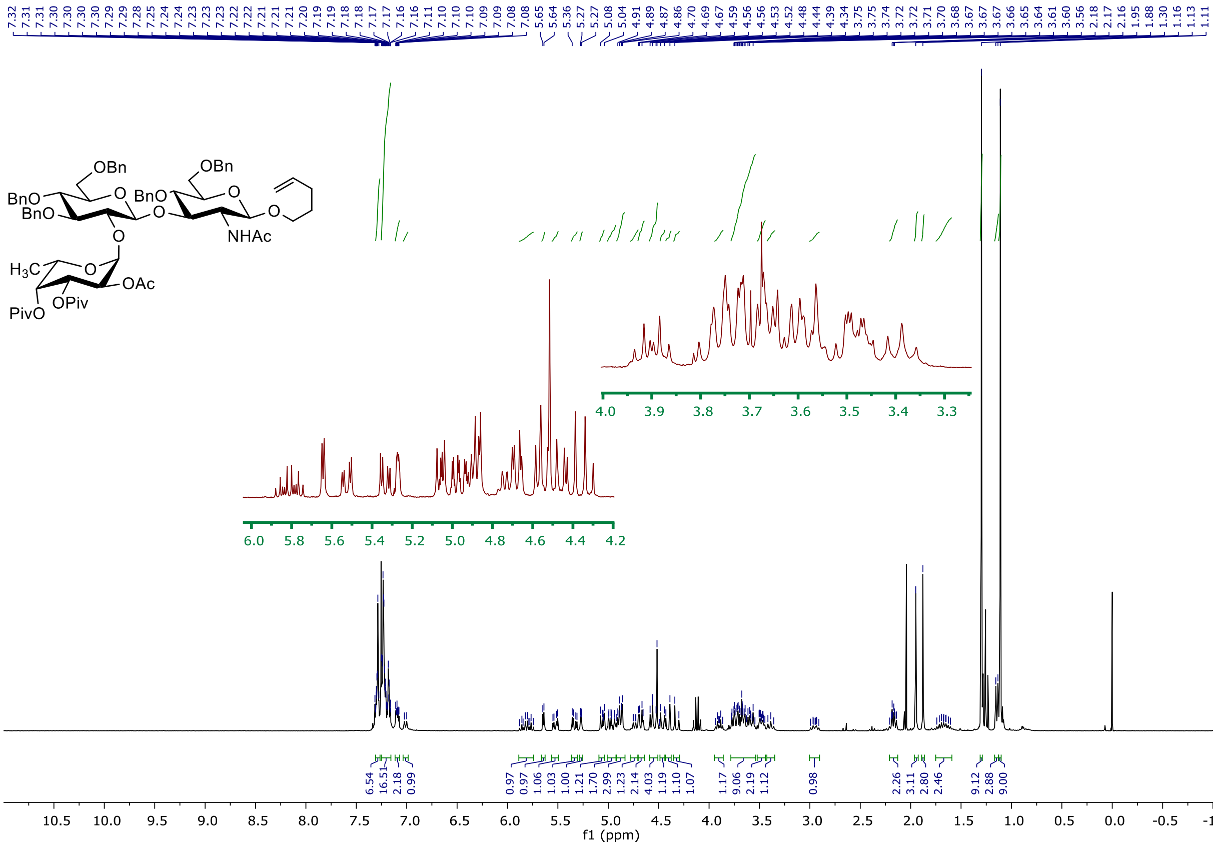

Figure S57. ^{1}H NMR ($CDCl_3$, 300 MHz) spectrum of **trisaccharide 50**

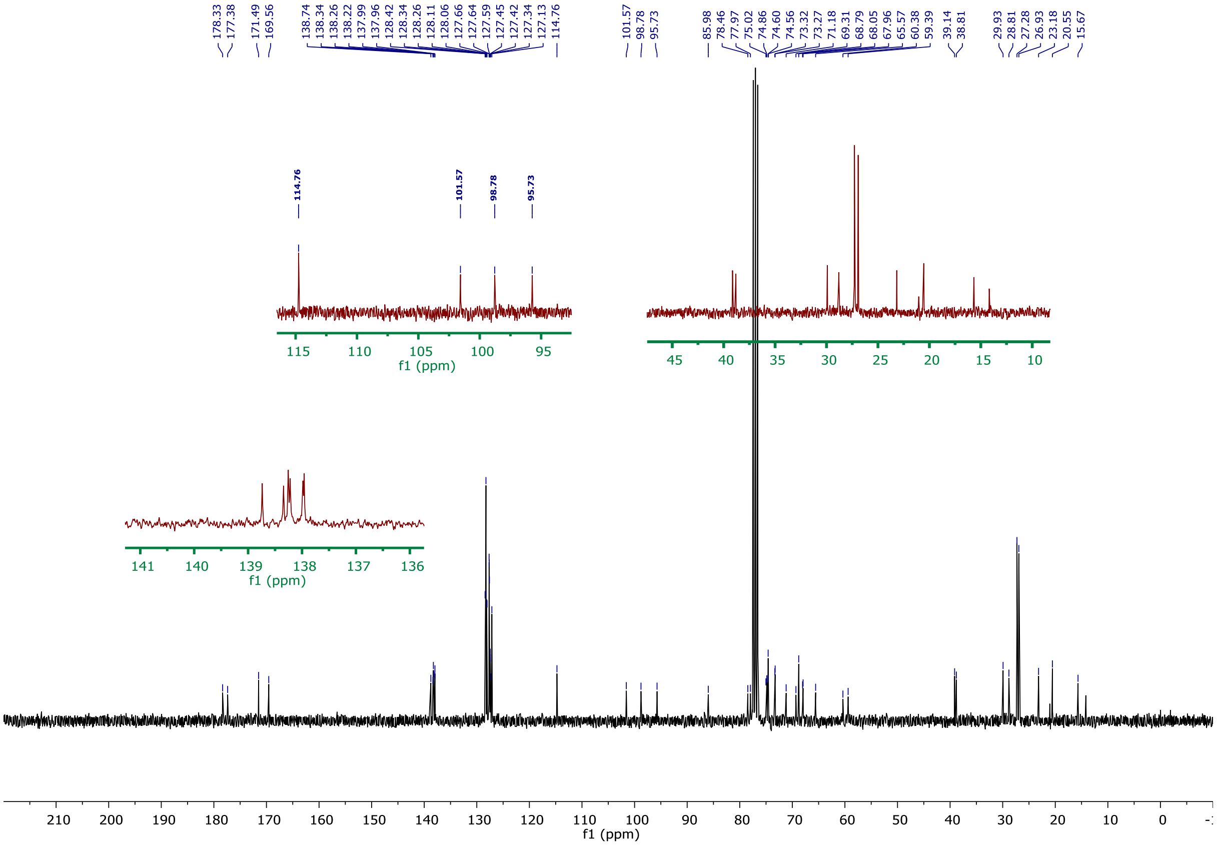

Figure S58. ^{13}C NMR ($CDCl_3$, 75 MHz) spectrum of **trisaccharide 50**

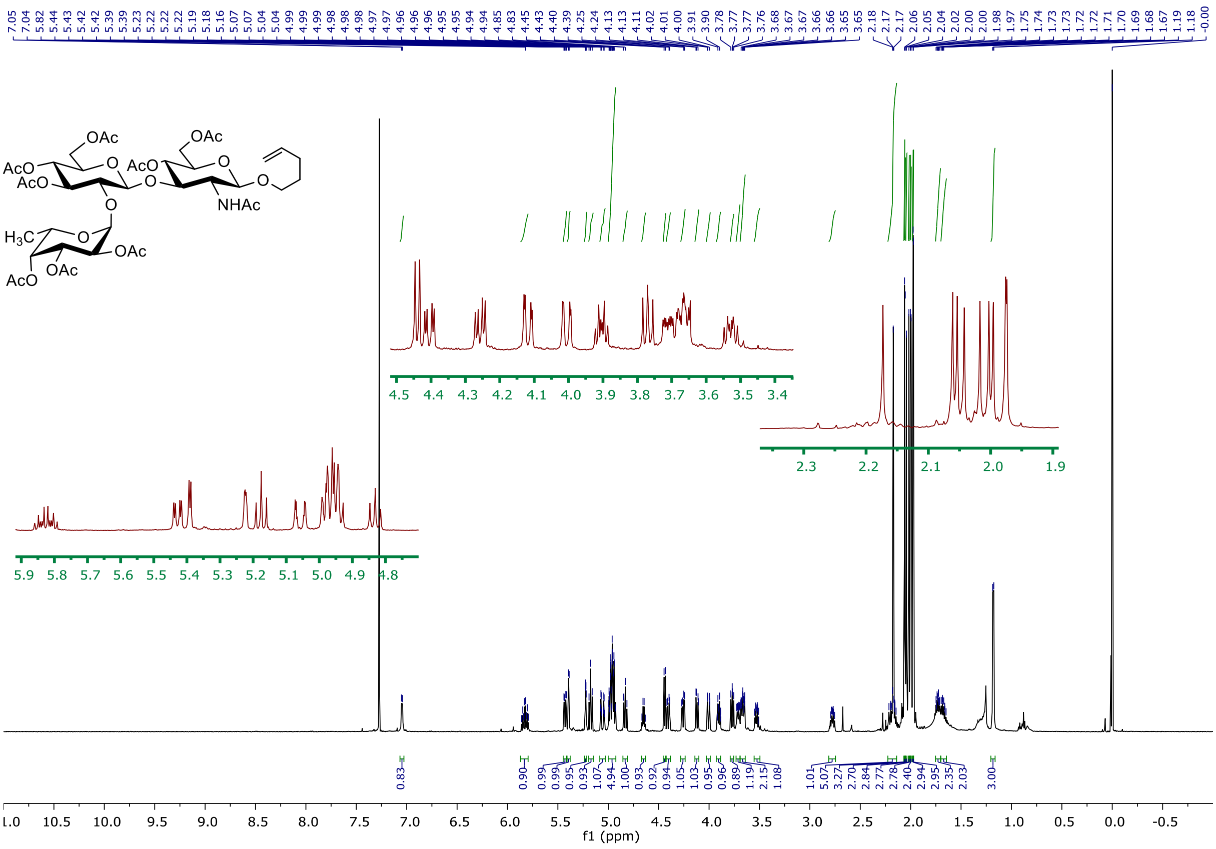

Figure S59. ^{1}H NMR ($CDCl_3$, 600 MHz) spectrum of **trisaccharide 98**

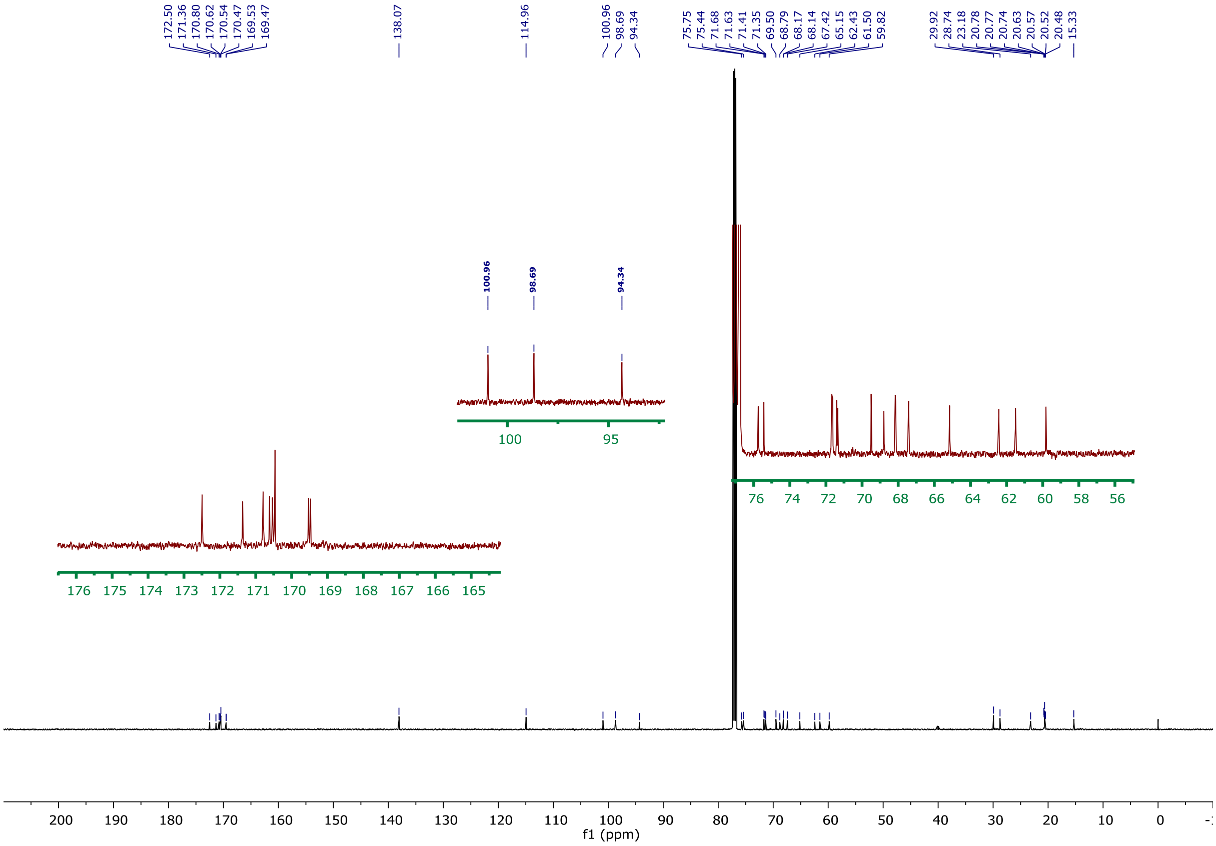

Figure S60. ^{13}C NMR ($CDCl_3$, 125 MHz) spectrum of **trisaccharide 98**

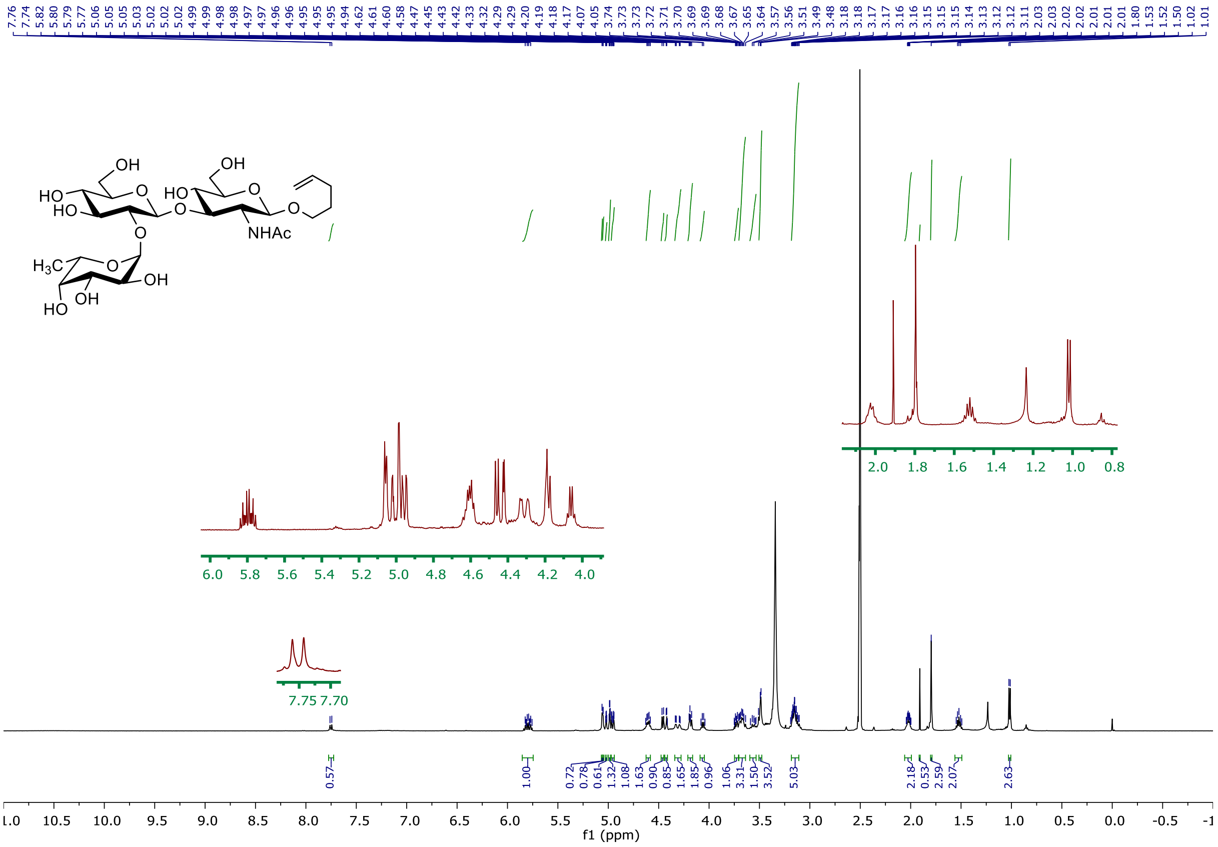

Figure S61. ^{1}H NMR ($CDCl_3$, 600 MHz) spectrum of **deprotected trisaccharide 51**

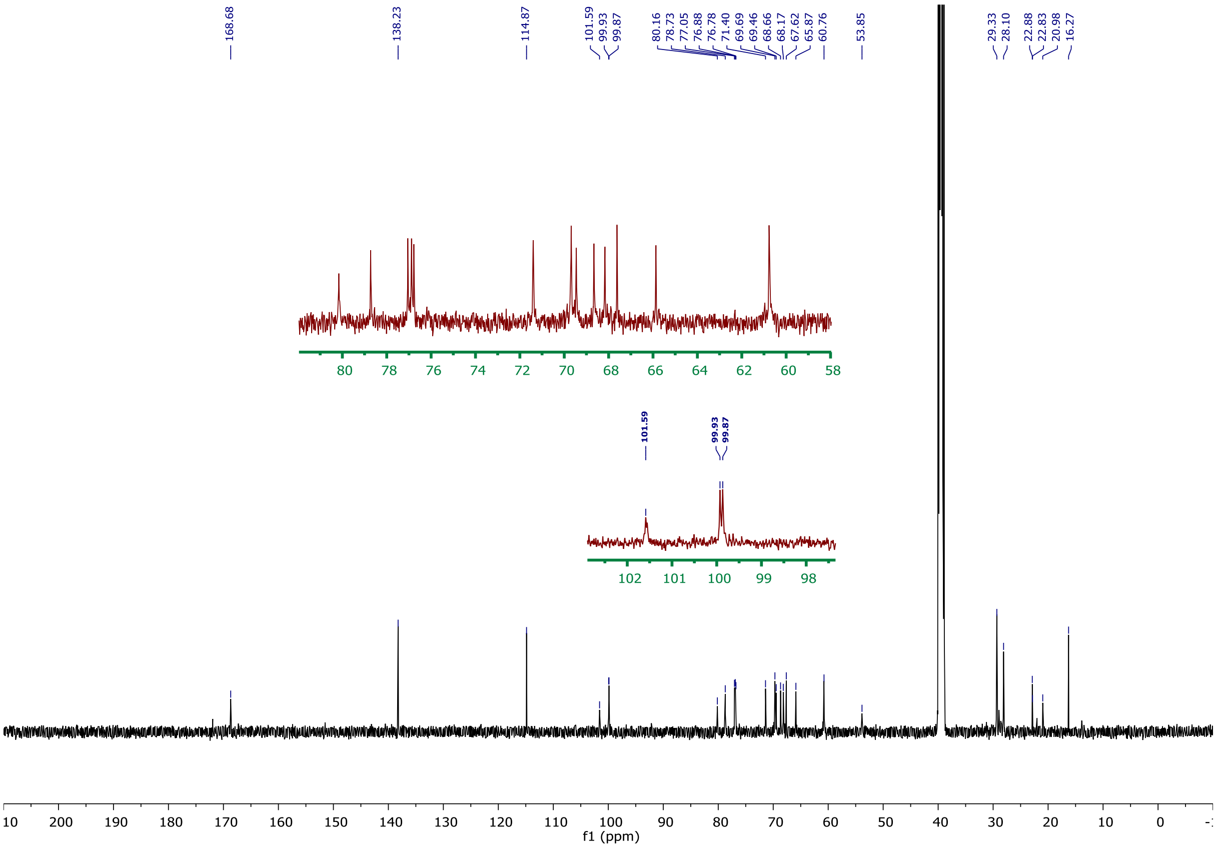

Figure S62. ^{13}C NMR ($CDCl_3$, 125 MHz) spectrum of **deprotected trisaccharide 51**

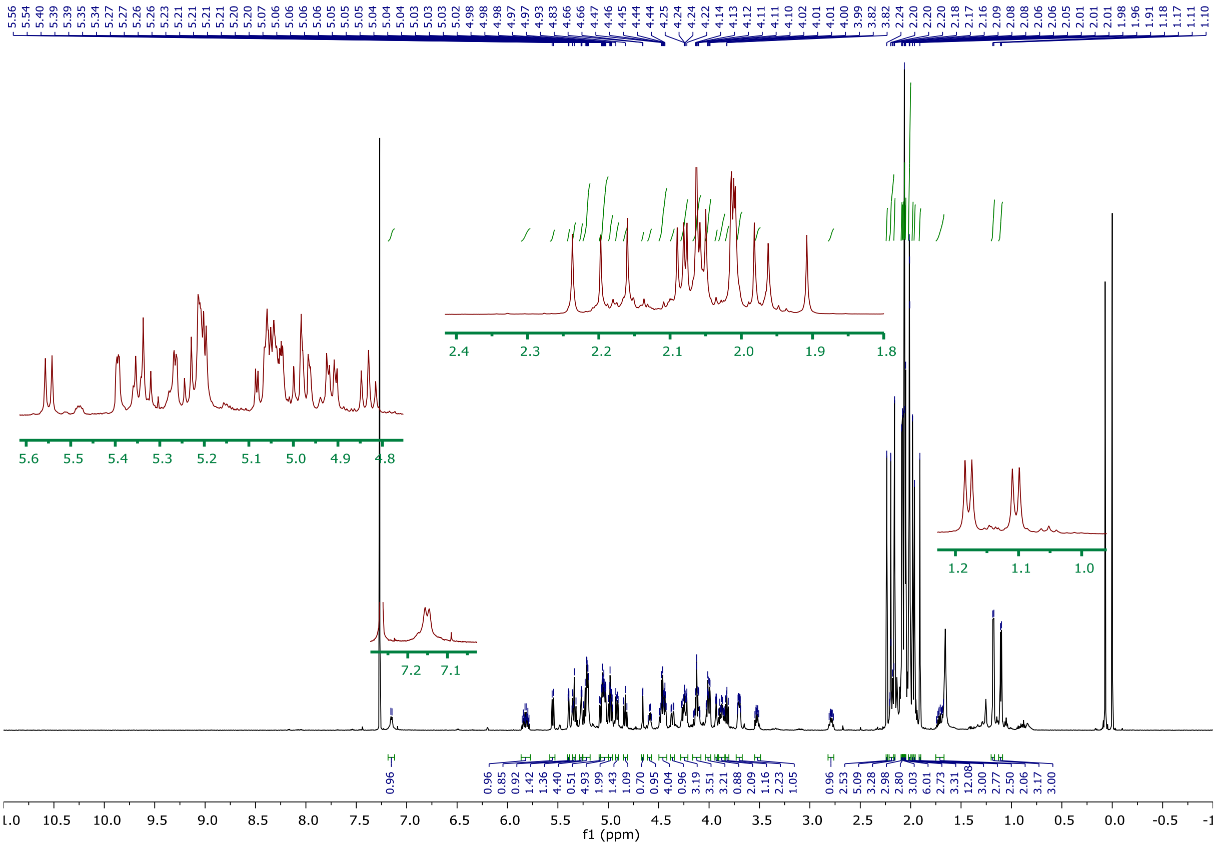

Figure S63. ^{1}H NMR ($CDCl_3$, 600 MHz) spectrum of **hexasaccharide 99**

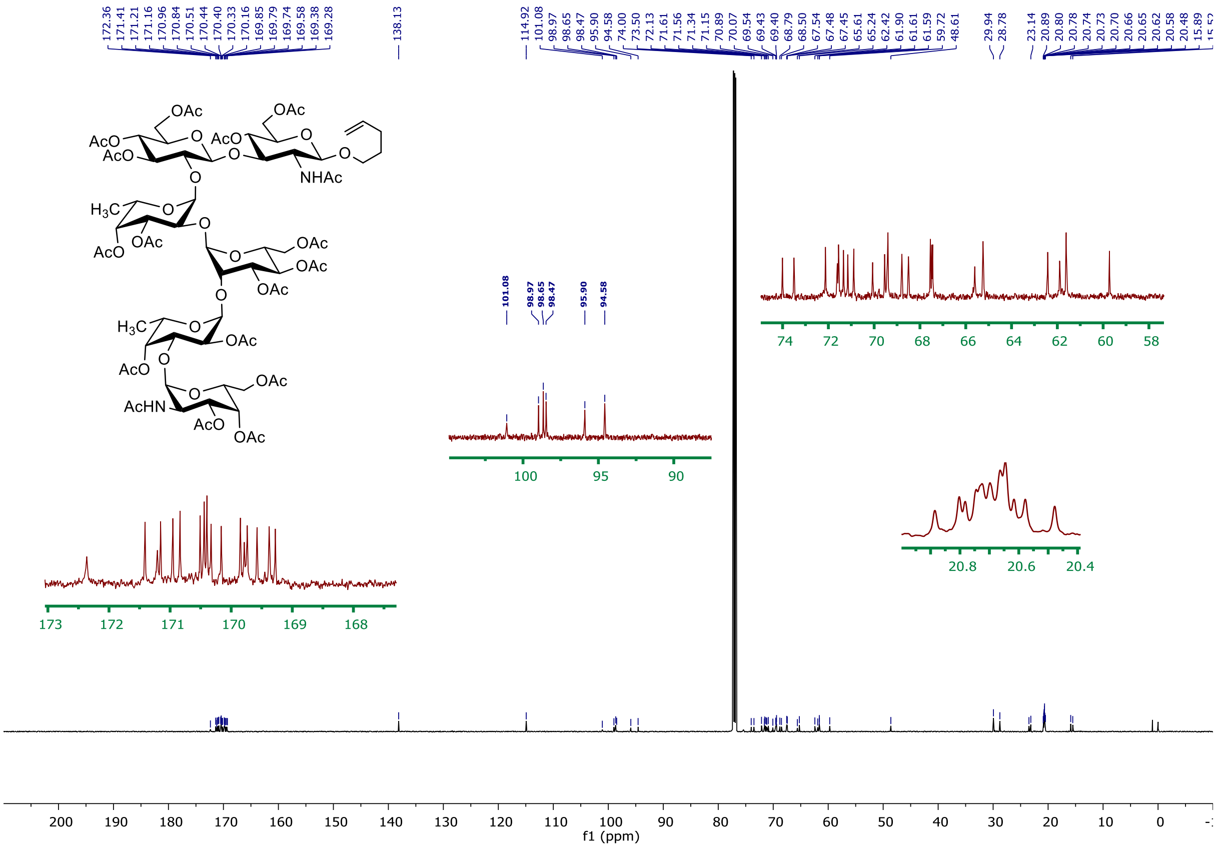

Figure S64. ^{13}C NMR ($CDCl_3$, 125 MHz) spectrum of **hexasaccharide 99**

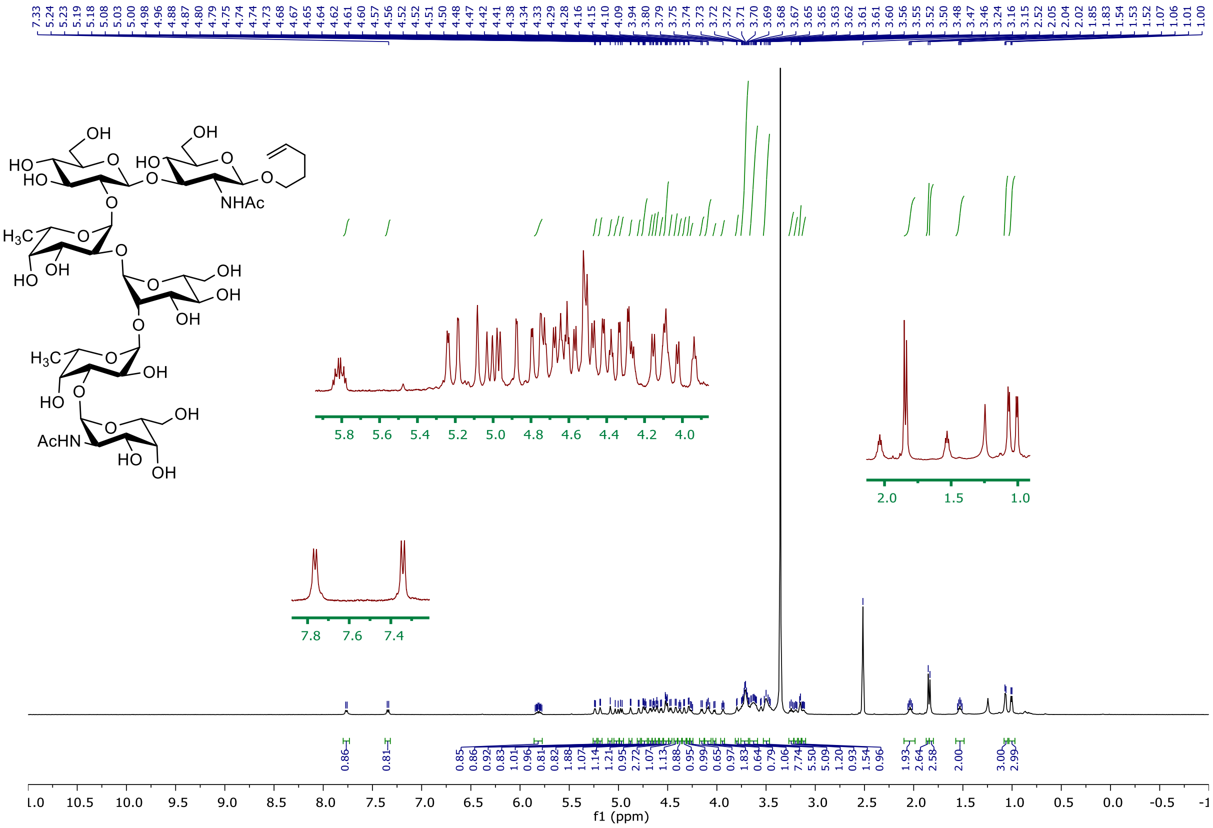

Figure S65. ^{1}H NMR (DMSO-d_6, 600 MHz) spectrum of **deprotected hexasaccharide 53**

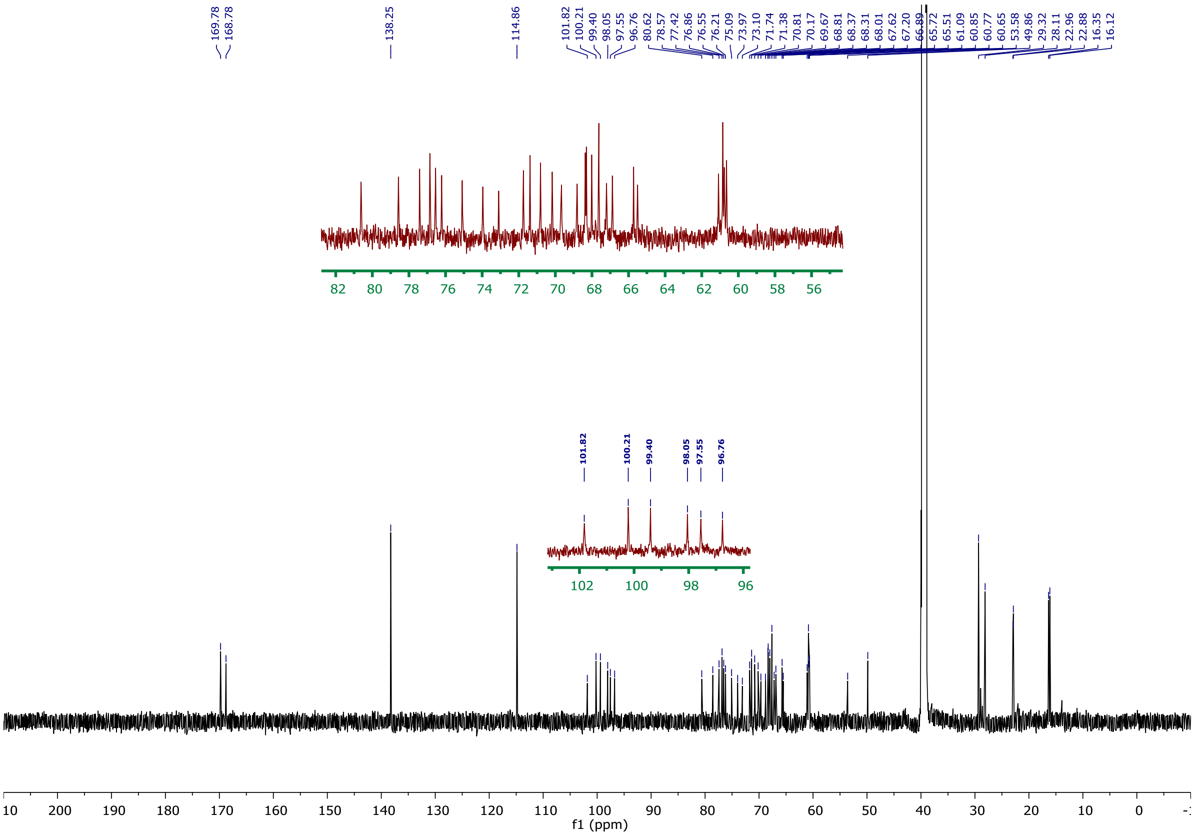

Figure S66. ^{13}C NMR (DMSO-d_6, 125 MHz) spectrum of **deprotected hexasaccharide 53**

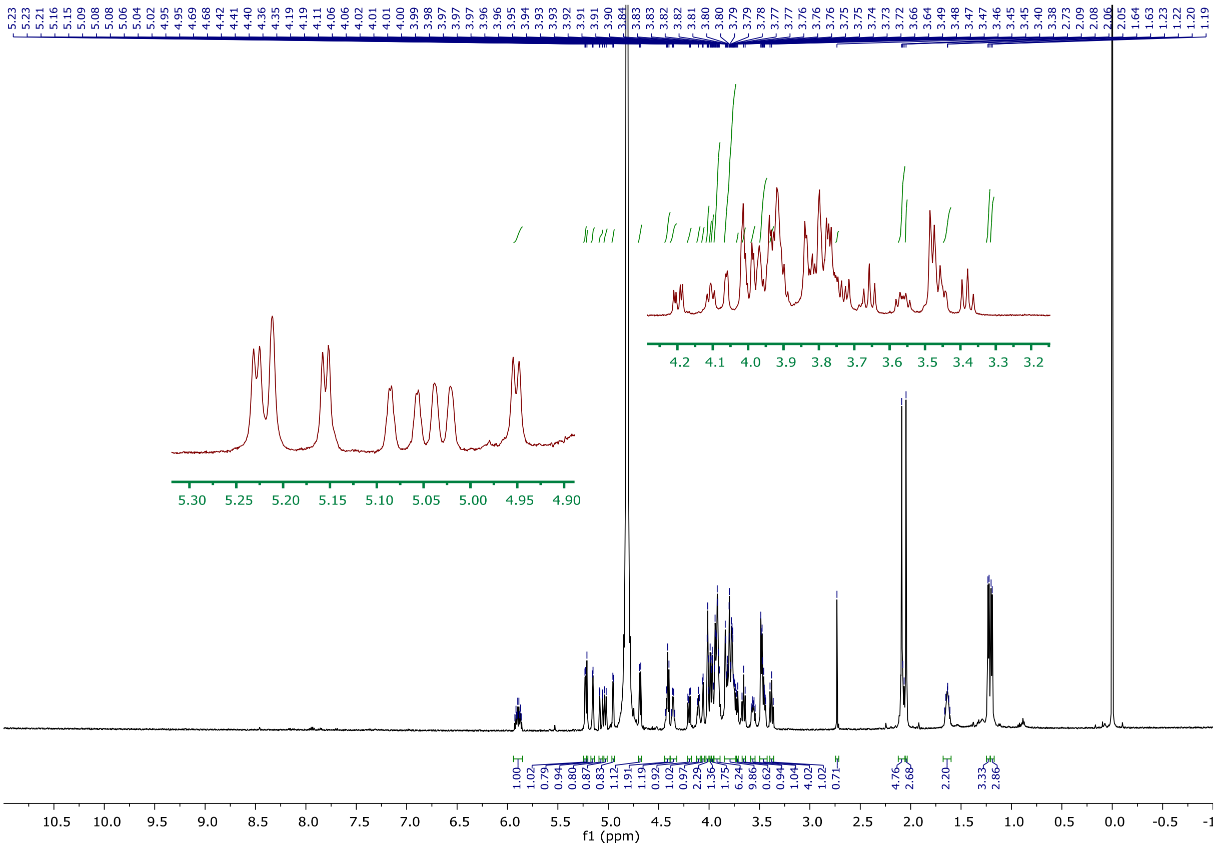

Figure S67. ^{1}H NMR (D_2O, 600 MHz) spectrum of **deprotected hexasaccharide 53**

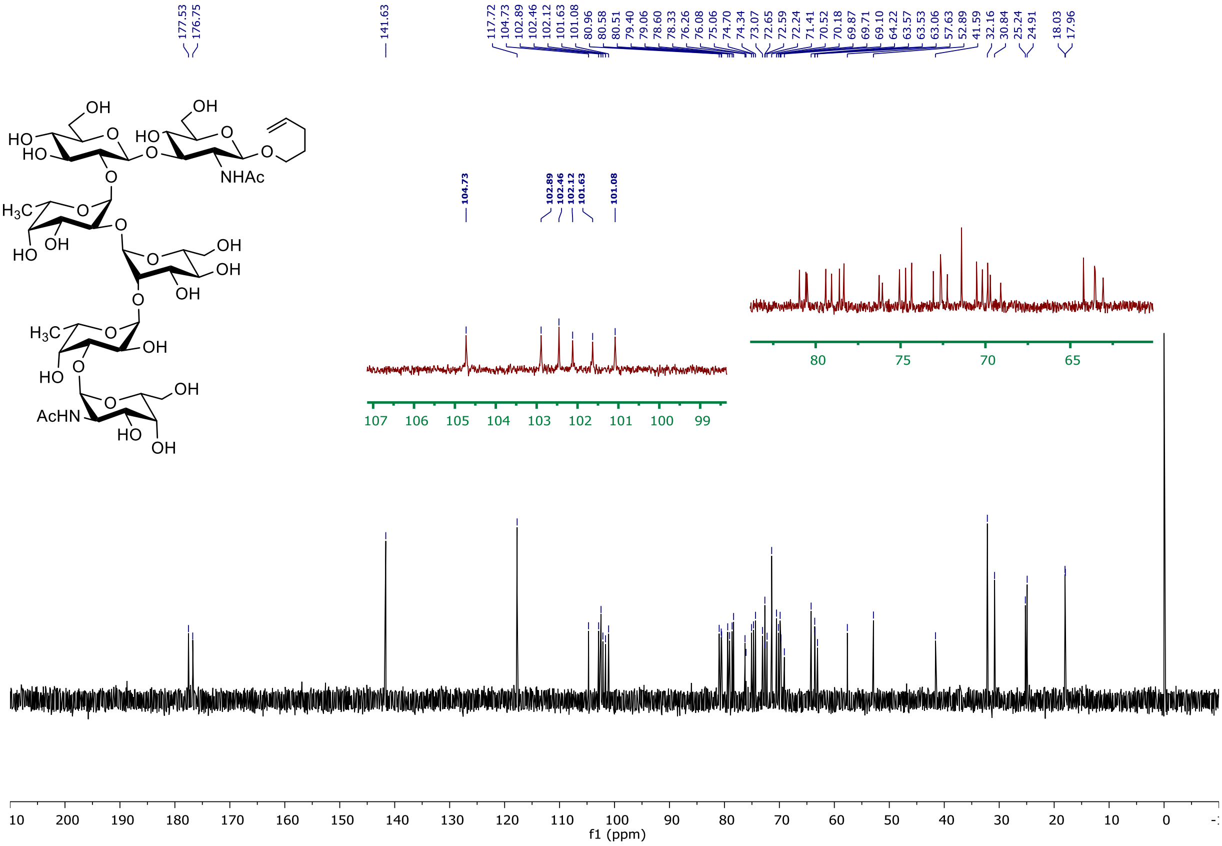

Figure S68. ^{13}C NMR (D_2O, 125 MHz) spectrum of **deprotected hexasaccharide 53**

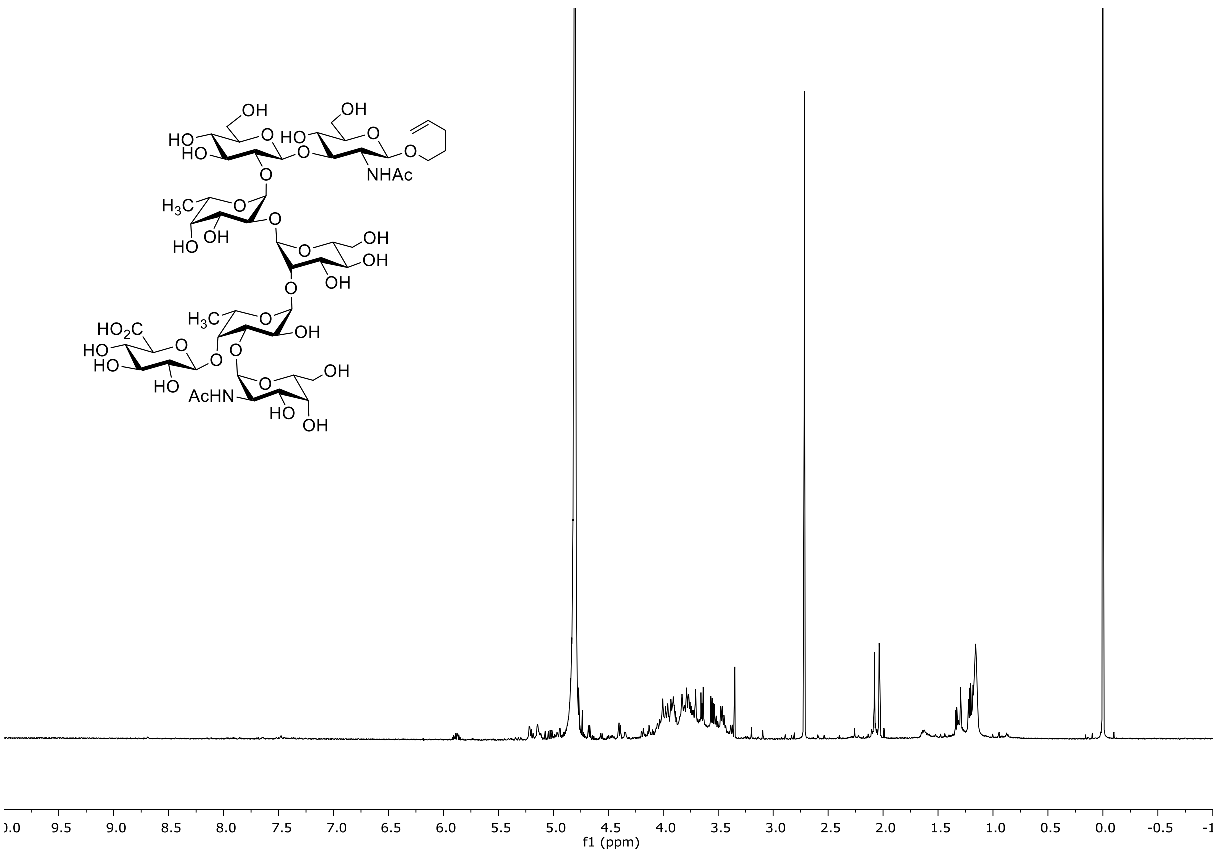

Figure S69. ^{1}H NMR (D_2O, 600 MHz) spectrum of **deprotected heptasaccharide 1**

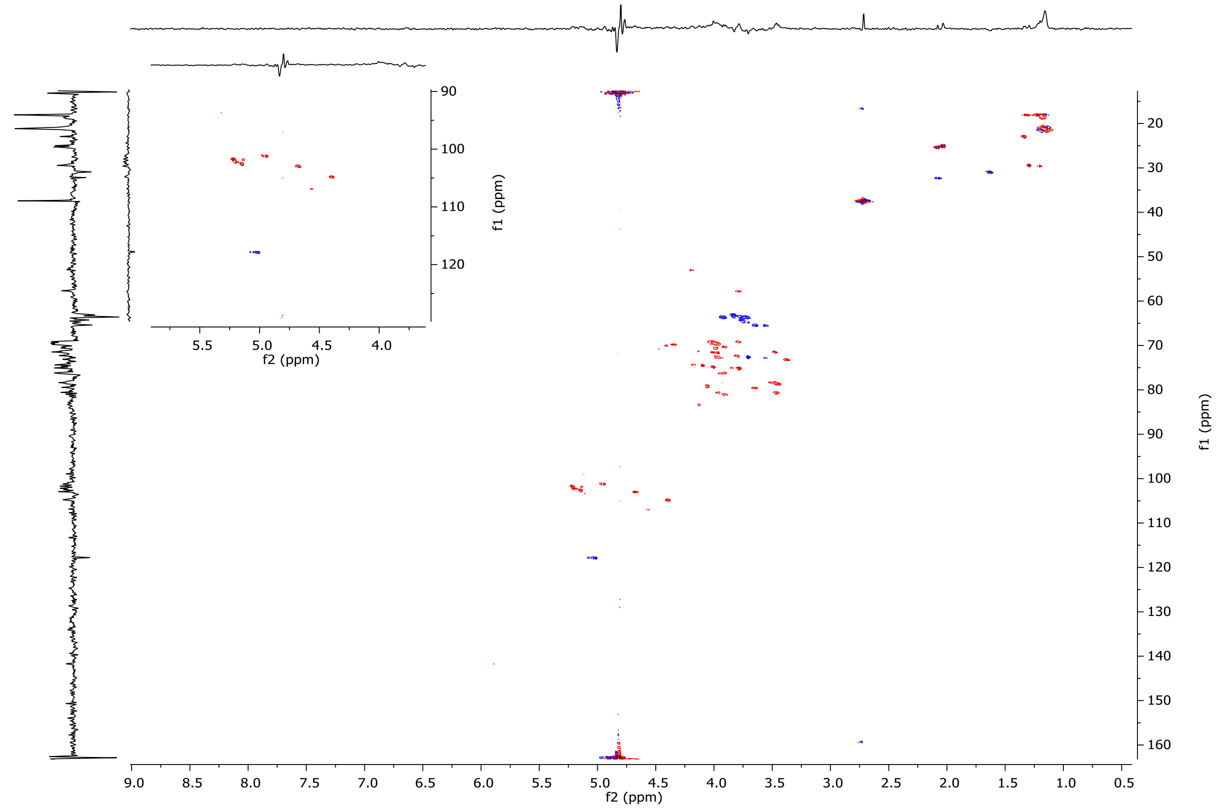

Figure S70. 2D NMR (D_2O, 125 MHz) spectrum of **deprotected heptasaccharide 1**

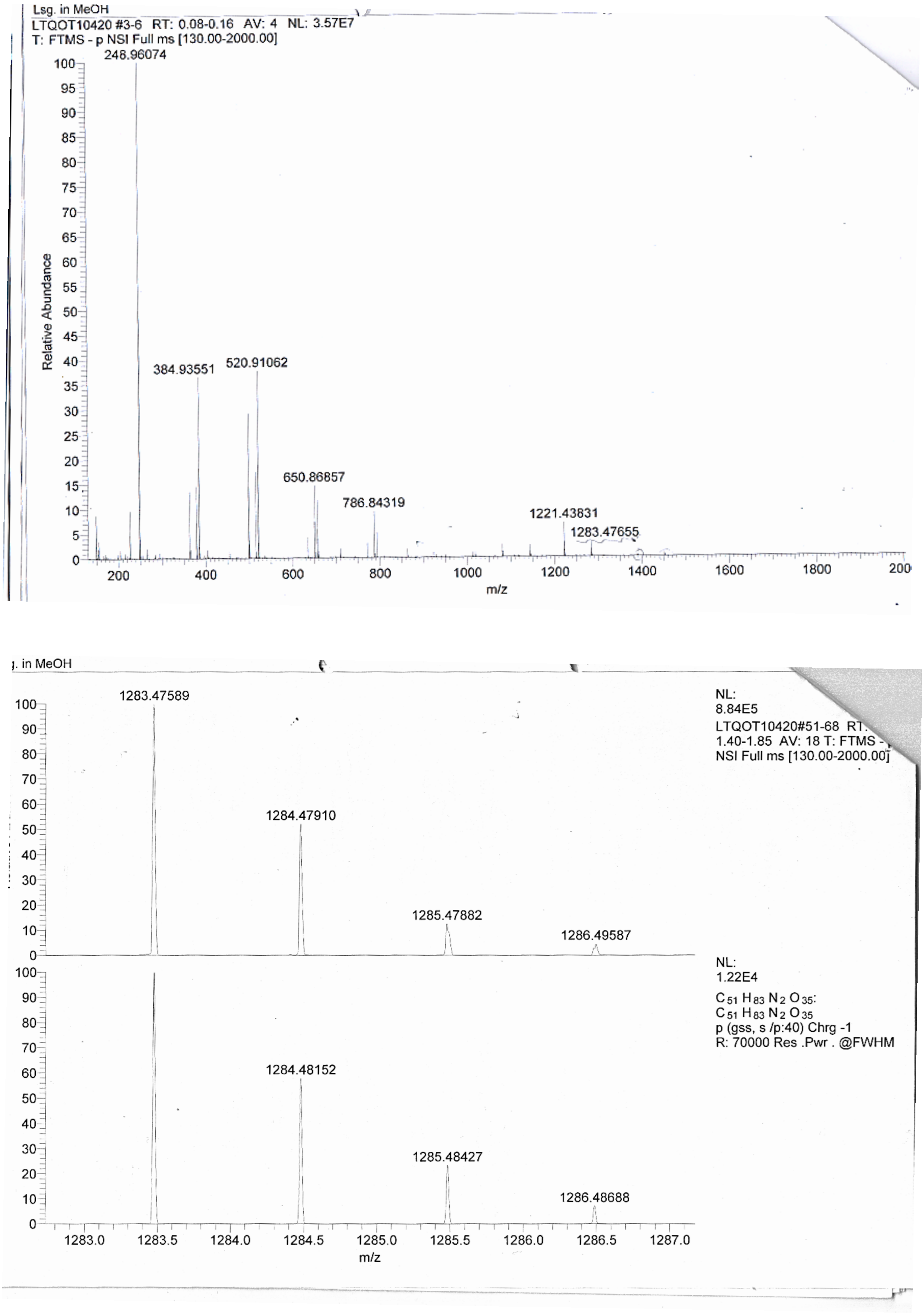

Figure S71. HR-Mass spectrum of **deprotected heptasaccharide 1**

11. Spectroscopic data of synthesized building blocks in failure strategies

11.1. *Synthesis of the trisaccharide 102 for coupling [3+4] strategy*

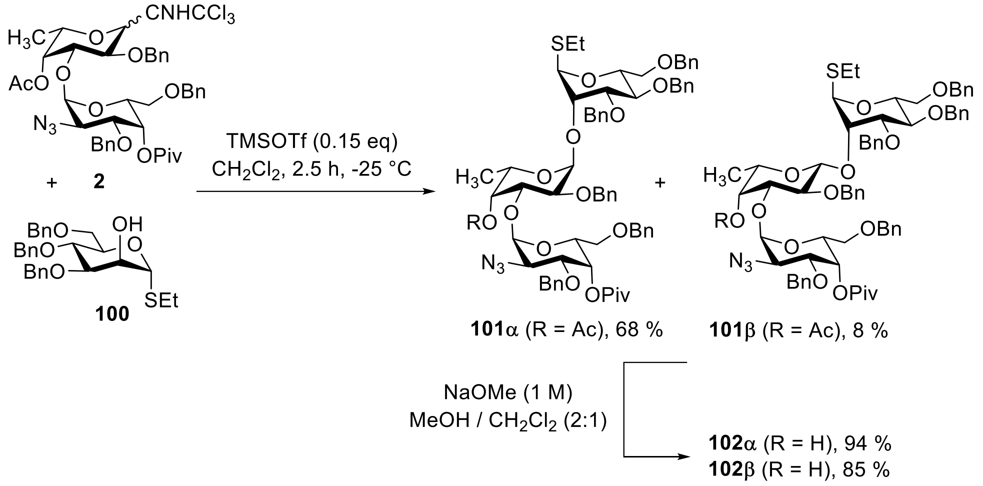

Ethyl (2-azido-3,6-di-*O*-benzyl-2-deoxy-4-*O*-pivaloyl-α-D-galactopyranosyl)-(1→3)-(4-*O*-acetyl-2-*O*-benzyl-L-fucopyranosyl)-3,4,6-tri-*O*-benzyl-1-thio-α-D-mannopyranoside (101)

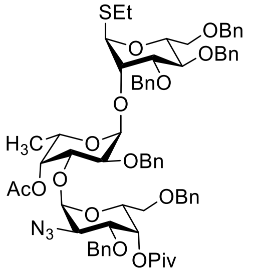

Compound **100** (23 mg, 46 μmol) and donor **2** (83 mg, 92 μmol) were mixed, co-evaporated with toluene (3x), dried *in vacuo* for 1 h and were dissolved in dichloromethane (1.0 mL). To a cooled solution at – 25 °C TMSOTf (1.2 μL, 6.9 μmol) was added and the reaction mixture was stirred for 2.5 h at – 25 °C. The reaction mixture was quenched by the addition of pyridine and evaporated to dryness. The resulting crude product was first purified by HPLC (gel permeation chromatography) and afterwards by silica gel column chromatography (pentane/ EtOAc, 8:1 → 4:1) to obtain 44 mg (76%, α/β 8:1) of trisacharide **101** as a colorless oil.

The analytical data is for the *α* anomer.

$[\alpha]_D^{24}$ = + 81.0° (c = 0.10, $CHCl_3$).

^{1}H NMR (600 MHz, $CDCl_3$): *δ* (ppm) 0.84 (d, *J* = 6.5 Hz, 3 H), 1.14 (s, 9 H), 1.24 (t, *J* = 7.2 Hz, 6 H), 2 (s, 3 H), 2.58 (mc, 2 H), 3.23 (dd, *J* = 9.9, 5.8 Hz, 1 H), 3.45 (dd, *J* = 9.9, 5.8 Hz, 1 H), 3.53 (dd, *J* = 10.0, 6.7 Hz, 1 H), 3.66 (dd, *J* = 11.5, 1.4 Hz, 1 H), 3.78–3.87 (m, 4 H), 3.94 (t, *J* = 9.5 Hz, 1 H), 4.06–4.10 (m, 2 H), 4.25 (dd, *J* = 10.0, 3.5 Hz, 1 H), 4.29–4.32 (m, 1 H), 4.40–4.56 (m, 7 H), 4.62 (s, 2 H), 4.66– 4.74 (m, 3 H), 4.80 (d, *J* = 10.8 Hz, 1 H), 4.90 (d, *J* = 3.6 Hz, 1 H, H–1B), 5.11 (d, *J* = 2.8 Hz, 1 H), 5.21 (d, *J* = 1.4 Hz, 1 H, H–1A), 5.24 (d, *J* = 3.6 Hz, 1 H, H–1C), 5.66 (d, *J* = 2.3 Hz, 1 H), 7.15–7.41 (m, 30 H).

^{13}C NMR (150 MHz, $CDCl_3$): *δ* (ppm) 15.0, 15.7, 20.8, 25.4, 27.1, 29.6, 39.0, 58.9, 64.9, 66.3, 68.5, 69.4, 70.9, 71.8, 72.0, 72.5, 72.6, 72.8, 73.1, 73.2, 73.3, 74.7, 74.8, 79.2, 81.3 (C-1A), 95.1 (C–1B), 99.2 (C–1C), 127.1,

127.4, 127.4, 127.5, 127.6, 127.7, 127.7, 127.9, 128.1, 128.2, 128.2, 128.3, 128.4, 137.3, 137.9, 138.1, 138.1, 138.3, 138.8, 170.9, 177.3.

IR (ATR): $\tilde{\nu}$ (cm^{-1}) 3089, 3063, 3030, 2958, 2924, 2868, 2106, 1734, 1605, 1496, 1478.

HR-MS (ESI): m/z calcd. for $C_{69}H_{81}N_3O_{15}SNa^+$ 1247.5314, found: 1247.5311.

The analytical data is for the *β* anomer.

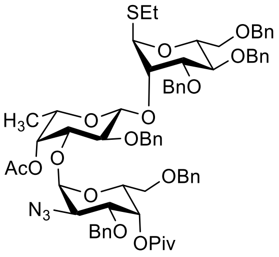

$[\alpha]_D^{24}$= + 1.0° (c = 0.12, $CHCl_3$).

^{1}H NMR (600 MHz, $CDCl_3$): *δ* (ppm) 1.03 (d, *J* = 6.4 Hz, 3 H), 1.14 (s, 9 H), 1.24–1.26 (m, 6 H), 2.03 (s, 3 H), 2.58 (m_c, 2 H), 3.25 (dd, *J* = 10.8, 3.6 Hz, 1 H), 3.38 (q, *J* = 6 Hz, 1 H), 3.48–3.53 (m, 2 H), 3.62 (dd, *J* = 10.7, 2 Hz, 1 H), 3.72–3.75 (m, 2 H), 3.80–3.83 (m, 3 H), 3.94 (t, *J* = 9.7 Hz, 1 H), 4.05–4.06 (m, 2 H), 4.24 (d, *J* = 10.8 Hz, 1 H), 4.39–4.45 (m, 4 H), 4.49 (d, *J* = 7.7 Hz, 1 H, H–1B), 4.51–4.60 (m, 4 H), 4.64 (t, *J* = 12.0 Hz, 2 H), 4.72 (d, *J* = 11.2 Hz, 1 H), 5.03 (d, *J* = 3.5 Hz, 1 H), 5.22 (d, *J* = 10.1 Hz, 1 H), 5.44 (d, *J* = 3.6 Hz, 1 H, H–1C), 5.50 (d, *J* = 1.2 Hz, 1 H, H– 1A), 5.61 (d, *J* = 2.8 Hz, 1 H), 7.01–7.42 (m, 30 H).

^{13}C NMR (150 MHz, $CDCl_3$): *δ* (ppm) 15.1, 16.0, 20.8, 25.3, 27.1, 29.6, 39.0, 58.7, 66.6, 68.7, 68.8, 69.4, 70.9, 71.9, 72.1, 72.3, 72.5, 73.1, 73.2, 74.1, 74.7, 75.0, 75.0, 77.3, 79.3, 80.4, 84.4 (C–1A), 99.2 (C–1C), 104.7 (C–1B), 127.3, 127.4, 127.4, 127.5, 127.6, 127.7, 127.8, 127.8, 127.9, 128.0, 128.2, 128.3, 128.4, 128.4, 128.5, 137.2, 138.1, 138.3, 138.5, 171.2, 177.5.

IR (ATR): $\tilde{\nu}$ (cm^{-1}) 3089, 3063, 3030, 2958, 2924, 2868, 2106, 1736, 1605, 1496, 1478.

HR-MS (ESI): m/z calcd. for $C_{69}H_{81}N_3O_{15}SNa^+$ 1204.5208, found: 1247.5311.

Ethyl (2-azido-3,6-di-*O*-benzyl-2-deoxy-4-*O*-pivaloyl-*α*-D-galactopyranosyl)-(1→3)-(2-*O*-benzyl-*α*-*L*-fucopyranosyl)-(1→2)-3,4,6-tri-*O*-benzyl-1-thio-*α*-D-mannopyranoside (102)

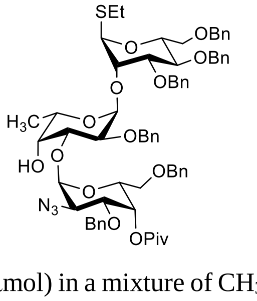

To the solution of compound **101** (34 mg, 28 μmol) in a mixture of CH_3OH and CH_2Cl_2 (1.0 mL, 2:1), a methanolic solution of NaOMe (1M, 2.8 μL, 2.8 μmol) was added and stirred for 24 h. the reaction mixture was neutralized with Amberlite acidic resin (IR-120). purification by silica gel column chromatography (pentane/EtOAc, 4:1 → 2:1) furnished 31 mg (94%) of **102** as a colorless oil.

$[\alpha]_D^{24}$= + 45.0° (c = 0.125, $CHCl_3$)

^{1}H NMR (600 MHz, CDCl$_3$): δ (ppm) 0.79 (d, J = 6.7 Hz, 3 H), 1.17 (s, 9 H), 1.24 (m, 3 H), 2.57 (m$_c$, 2 H), 2.92 (d, J = 5.1 Hz, 1 H), 3.49–3.54 (m, 2 H), 3.60 (dd, J = 10.6, 3.8 Hz, 1 H), 3.68 (dd, J = 11.4, 1.7 Hz, 1 H), 3.80–3.85 (m, 4 H), 3.96 (t, J = 9.6 Hz, 1 H), 3.99 (dd, J = 10.6, 3.3 Hz, 1 H), 4.00 (d, J = 3.4 Hz, 1 H), 4.03 (dd, J = 10.1, 3.3 Hz, 1 H), 4.08–4.13 (m, 4 H), 4.44–4.72 (m, 10 H), 4.80 (d, J = 11.5 Hz, 1 H), 4.84–4.86 (m, 2 H), 5.08 (d, J = 3.8 Hz, 1 H), 5.28 (d, J = 1.7 Hz, 1 H), 5.57 (d, J = 2.1 Hz, 1 H), 7.19–7.40 (m, 30 H).

^{13}C NMR (150 MHz, CDCl$_3$): δ (ppm) 15.0, 15.8, 25.4, 27.1, 39.1, 59.9, 65.9, 66.3, 68.9, 69.4, 69.5, 71.2, 71.3, 71.8, 72.5, 72.6, 73.2, 73.9, 74.3, 74.5, 74.7, 74.8, 74.9, 79.1, 79.4, 81.5, 96.3, 99.1, 127.2, 127.4, 127.4, 127.5, 127.5, 127.9, 127.9, 128.0, 128.0, 128.1, 128.2, 128.3, 128.3, 128.5, 136.9, 137.0, 138.2, 138.4, 138.8, 138.9, 177.5.

IR (ATR): $\tilde{\nu}$ (cm^{-1}) 3473, 3089, 3062, 3030, 2970, 2927, 2869, 2108, 1953, 1731, 1605.

HR-MS (ESI): m/z calcd. for $C_{76}H_{79}N_3O_{14}SNa^+$ 1204.5208, found: 1204.5233.

11.2. *Synthesis of the tetrasaccharide donor 105 for coupling [3+4] strategy*

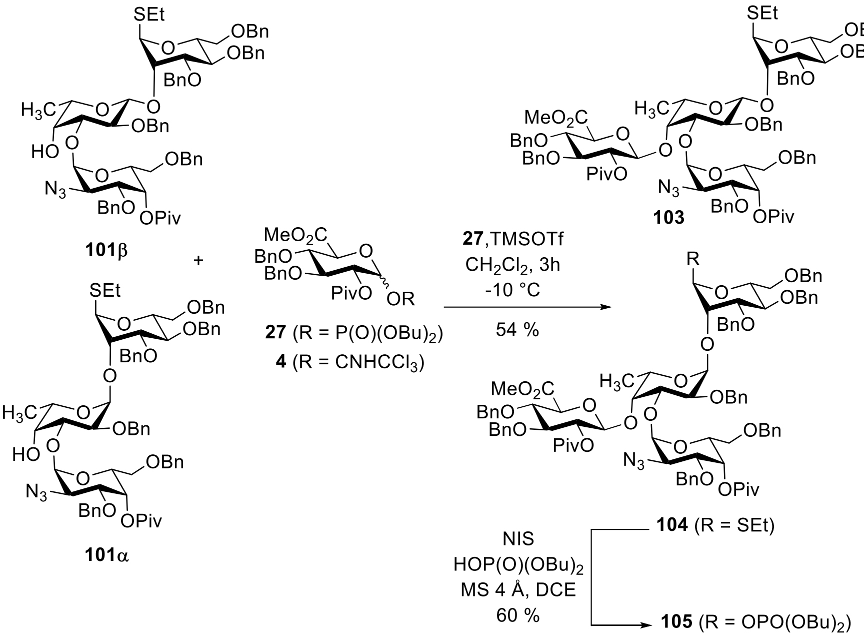

Ethyl (2-azido-3,6-di-*O*-benzyl-2-deoxy-4-*O*-pivaloyl-α-D-galactopyranosyl)-(1→3)-[(methyl-3,4-di-*O*-benzyl-2-*O*-pivaloyl-β-D-glucopyranosyluronate)-(1→4)]-(2-*O*-benzyl-α-L-fucopyranosyl)-(1→2)-3,4,6-tri-*O*-benzyl-1-thio-α-D-mannopyranoside (104)

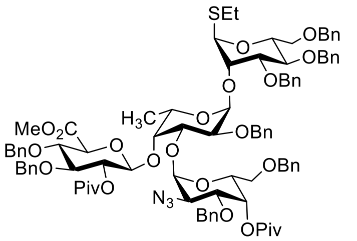

Compound **101** (80 mg, 67 µmol) and donor **27** (0.13 g, 0.20 mmol) were mixed, co-evaporated with toluene (3x), dried *in vacuo* for 1 h and were dissolved in dichloromethane (2.0 mL). To a cooled solution at – 10 °C a diluted

solution of TMSOTf (36 μL, 0.20 mmol) in CH_2Cl_2 was added and the reaction mixture was stirred for 3 h. Afterwards, it was quenched by the addition of pyridine and evaporated to dryness. The resulting crude product was first purified by HPLC (gel permeation chromatography) and afterwards by silica gel column chromatography (pentane / EtOAc, 8:1 → 6:1) to furnish 60 mg (54% isolated, 67% brsm) of tetrasaccharide **104** as a colorless oil.

$[\boldsymbol{\alpha}]_{\mathbf{D}}^{\mathbf{24}}$ = + 8.2° (c = 0.14, $CHCl_3$).

^{1}H NMR (600 MHz, $CDCl_3$): δ (ppm) 0.90 (d, J = 6.6 Hz, 3 H), 1.16–1.28 (m, 21 H), 2.59 (m_c, 2 H), 3.32 (q, J = 3.6 Hz, 1 H), 3.36 (q, J = 5.6 Hz, 1 H), 3.45 (q, J = 6.7 Hz, 1 H), 3.66 (s, 3 H), 3.67–3.70 (m, 2 H), 3.80 (q, J = 3.0 Hz, 1 H), 3.84–3.89 (m, 5 H), 3.96 (t, J = 9.5 Hz, 1 H), 4.00–4.14 (m, 5 H), 4.23 (q, J = 3.5 Hz, 1 H), 4.35–4.50 (m, 5 H), 4.52 (d, J = 8.0 Hz, 1 H, H–1D), 4.56–4.77 (m, 9 H), 4.79 (d, J = 3.4 Hz, 1 H, H–1B), 4.81–4.84 (m, 2 H), 5.10 (d, J = 3.5 Hz, 1 H, H–1C), 5.17 (s_{br}, 1 H, H–1A), 5.22 (q, J = 8.0 Hz, 1 H), 5.66 (d, J = 2.7 Hz, 1 H), 7.14–7.47 (m, 40 H).

^{13}C NMR (125 MHz, CDCl3): δ (ppm) 15.0, 16.8, 25.4, 27.2, 27.3, 29.6, 38.8, 39.0, 52.6, 59.5, 66.5, 67.2, 68.3, 69.3, 69.5, 71.3, 71.6, 72.6, 72.9, 73.3, 73.4, 73.5, 73.8, 74.2, 74.5, 74.6, 74.8, 74.9, 74.9, 76.3, 79.4, 79.8, 81.6, 82.3 (C–1A), 96.0 (C–1B), 99.4 (C–1C), 100.9 (C-1D), 127.0, 127.3, 127.4, 127.4, 127.6, 127.6, 127.7, 127.9, 128.0, 128.1, 128.1, 128.2, 128.2, 128.3, 128.3, 128.4, 128.5, 137.4, 137.9, 138.2, 138.3, 138.4, 138.9, 138.9, 169.1, 176.4, 177.7.

IR (ATR): $\tilde{\nu}$ (cm^{-1}) 3089, 3063, 3030, 2958, 2924, 2870, 2107, 1951, 1746, 1731, 1605.

HR-MS (ESI): m/z calcd. for $C_{93}H_{109}N_3O_{21}SNa^+$ 1658.7166, found: 1658.7167.

(2-Azido-3,6-di-*O*-benzyl-2-deoxy-4-*O*-pivaloyl-α-D-galactopyranosyl)-(1→3)-[(methyl-3,4-di-*O*-benzyl-2-*O*-pivaloyl-β-D-glucopyranosyluronate)-(1→4)]-(2-*O*-benzyl-α-L-fucopyranosyl)-(1–2)-3,4,6-tri-*O*-benzyl-D-mannopyranosyl Dibutyl Phosphate (105)

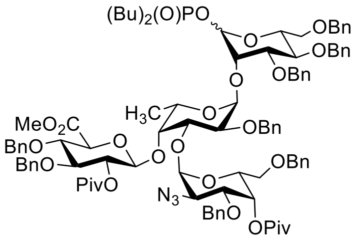

A solution of **104** (32 mg, 19 μmol) in DCE (1.0 ml) and dibutyl phosphate (5.8 μL, 29 μmol) in the presence of 4 Å molecular sieves was stirred for 30 min at room temperature. NIS (3.9 mg, 29 μol) was added and the reaction mixture was stirred for 22 h at room temperature. The reaction mixture was diluted with dichloromethane, filtered through celite and washed with an aq. solution of $Na_2S_2O_3$ (1 M). The organic layer was dried over $MgSO_4$ and evaporated to dryness. The resulting crude product was purified by silica gel column chromatography (pentane/EtOAc, 5:1 → 1:1) to afford 21 mg (60%, α/β 2.5:1) of **105** as a colorless oil.

The analytical data is for the α anomer.

$[\boldsymbol{\alpha}]_{\mathbf{D}}^{\mathbf{24}}$ = + 2.4° (c = 0.47, $CHCl_3$).

^{1}H NMR (600 MHz, $CDCl_3$): δ (ppm) 0.88 (mc, 6 H), 1.12 (d, J = 6.3 Hz, 3 H), 1.15 (s, 9 H), 1.17 (s, 9 H), 1.35 (mc, 4 H), 1.58 (mc, 4 H), 3.29 (dd, J = 11.0, 3.7 Hz, 1 H), 3.33 (dd, J = 9.7, 5.4 Hz, 1 H), 3.43–3.69 (m, 4 H), 3.64 (s, 3 H), 3.81–4.02 (m, 12 H), 4.14 (d, J = 6.8 Hz, 1 H), 4.17–4,20 (m, 1 H), 4.21 (dd, J = 11.5, 3.0 Hz, 1 H), 4.29–4.40 (m, 3 H), 4.43–4.49 (m, 3 H), 4.51 (d, J = 7.8 Hz, 1 H, H-1D), 4.54 (t, J = 11.5 Hz, 1 H), 4.57 (d, J =

3.5 Hz, 1 H, H-1B), 4.59–4.77 (m, 8 H), 4.81 (dd, *J* = 5.0, 10.8 Hz, 2 H), 5.07 (d, *J* = 3.8 Hz, 1 H, H–1C), 5.20 (dd, *J* = 9.5, 7.6 Hz, 1 H), 5.58 (dd, *J* = 6.1, 1.6 Hz, 1 H, H–1A), 5.64 (d, *J* = 2.5 Hz, 1 H), 7.13–7.45 (m, 40 H).

^{13}C NMR (125 MHz, $CDCl_3$): δ (ppm) 13.6, 18.6, 18.6, 26.9, 27.2, 27.2, 27.4, 32.2, 32.2, 38.8, 39.1, 52.7, 59.5, 66.6, 67.2, 67.7, 67.7, 68.4, 69.0, 69.3, 71.3, 71.8, 72.8, 72.9, 73.2, 73.4, 73.4, 73.5, 73.8, 73.8, 74.0, 74.9., 75.0, 75.9, 77.7, 79.7, 82.3, 95.3 (d, $^{2}J_{C,P}$ = 6.3 Hz, 1 C, C–1A), 96.6 (C-1B), 99.3 (C-1C), 100.8 (C-1D), 127.0, 127.1, 127.2, 127.2, 127.3, 127.4, 127.5, 127.6, 127.6, 127.8, 127.9, 128.0, 128.1, 128.2, 128.2, 128.2, 128.2, 128.3, 128.3, 128.5, 137.3, 137.7, 137.8, 137.9, 138.1, 138.4, 138.6, 138.6, 168.9, 176.2, 177.6.

IR (ATR): $\tilde{\nu}$ (cm^{-1}) 3089, 3063, 3030, 2960, 2931, 2871, 2107, 1746, 1730, 1605, 1496.

HR-MS (ESI): m/z calcd. for $C_{99}H_{122}N_3O_{25}PNa^+$ 1807.8031, found: 1807.8031.

11.3. Synthesis of the trisaccharide 100 / 101 for coupling [4+3] strategy

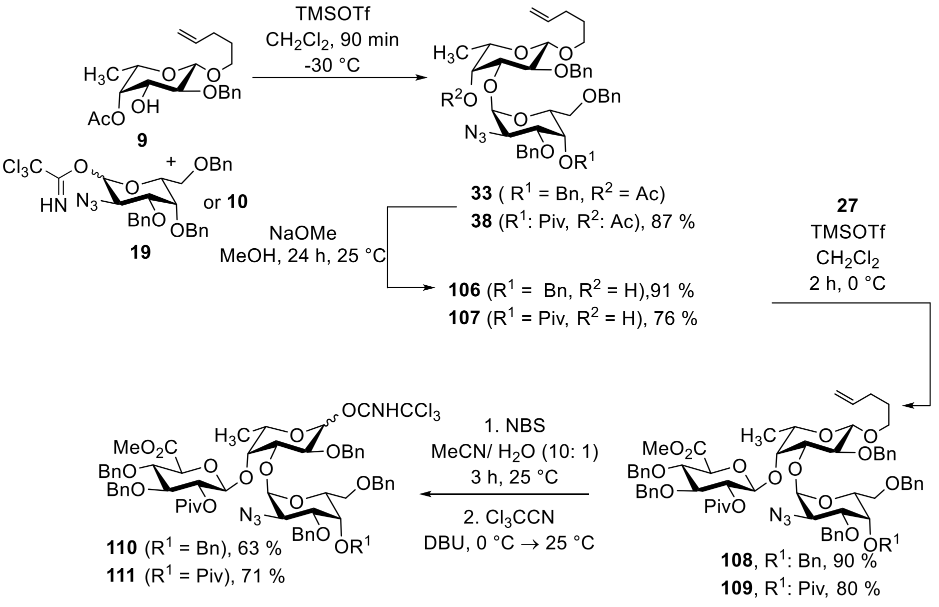

_n_-Pentenyl (2-azido-3,4,6-tri-*O*-benzyl-2-deoxy-α-D-galactopyranosyl)-(1→3)-[(methyl-3,4-di-*O*-benzyl-2-*O*-pivaloyl-β-D-glucopyranosyluronate)-(1→4)]-2-*O*-benzyl-β-L-fucopyranoside (108)

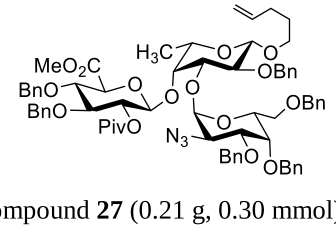

Compound **106** (60 mg, 76 µmol) and compound **27** (0.21 g, 0.30 mmol) were mixed, co-evaporated with toluene (3x), dried *in vacuo* for 1 h and dissolved in CH_2Cl_2 (15 mL). To the cooled soln. at 0 °C, TMSOTf (55 µL, 0.30 mmol) was added and the reaction mixture was stirred for 2 h. After completion of the reaction pyridine was added and the reaction mixture was evaporated to dryness. The resulting crude product was purified by HPLC (gel permeation chromatography) to furnish 85 mg (90%) of trisaccharide **108** as a colorless oil.

$[\alpha]_D^{24}$ = + 1.1° (c = 0.22, $CHCl_3$).

^{1}H NMR (300 MHz, $CDCl_3$): δ (ppm) 1.11 (d, *J* = 6.5 Hz, 3 H), 1.23 (s, 9 H), 1.62 (mc, 2 H), 2.10 (mc, 2 H), 3.23 (dd, *J* = 9.9, 4.5 Hz, 1 H), 3.29 (q, *J* = 6.7 Hz, 1 H), 3.39 (s, 3 H), 3.41–3.45 (m, 1 H), 3.52 (q, *J* = 6.5 Hz, 1 H), 3.57–3.64 (m, 2 H), 3.70–3.87 (m, 6 H), 4.05 (d, *J* = 1.5 Hz, 1 H), 4.14 (t, *J* = 5.5 Hz, 1 H), 4.20 (d, *J* = 7.7 Hz, 1

H, H–1A), 4.25 (dd, J = 11.2, 2.5 Hz, 1 H), 4.33 (d, J = 11.5 Hz, 1 H), 4.42 (d, J = 11.7 Hz, 1 H), 4.46 (d, J = 11.3 Hz, 1 H), 4.58 (d, J = 8.1 Hz, 1 H, H–1C), 4.62 (d, J = 11.3 Hz, 1 H), 4.72 (t, J = 9.5 Hz, 2 H), 4.78 (d, J = 10.0 Hz, 1 H), 4.75 (s, 1 H), 4.87–5.01 (m, 6 H), 5.22 (t, J = 8.5 Hz, 1 H), 5.38 (d, J = 3.8 Hz, 1 H, H–1B), 5.79 (mc, 1 H), 7.16–7.53 (m, 30 H).

^{13}C NMR (125 MHz, $CDCl_3$): δ (ppm) 17.2, 27.4, 29.1, 30.3, 38.8, 52.1, 59.6, 68.4, 69.8, 70.0, 70.6, 72.1, 72.9, 73.2, 74.0, 74.2, 74.7, 74.9, 74.9, 75.6, 76.1, 77.2, 79.0, 82.4, 99.7 (C– 1B), 100.9 (C–1B), 103.5 (C–1C), 114.5, 127.0, 127.2, 127.3, 127.4, 127.5, 127.5, 127.5, 127.6, 127.6, 127.7, 127.7, 127.8, 127.9, 128.0, 128.0, 128.1, 128.2, 128.2, 128.2, 128.3, 128.03, 128.4, 128.4, 137.4, 137.7, 138.1, 138.2, 138.3, 138.5, 138.8, 168.9, 176.3.

IR (Film): $\tilde{\nu}$ (cm^{-1}) 3088, 3064, 3031, 2930, 2872, 2682, 2109, 1952, 1748, 1640, 1587.

HR-MS (ESI): m/z calcd. for $C_{71}H_{83}N_3O_{16}Na^+$ 1256.5666, found: 1256.5652.

2-Azido-3,4,6-tri-*O*-benzyl-2-deoxy-α-D-galactopyranosyl-(1→3)-[(methyl-3,4-di-*O*-benzyl-2-*O*-pivaloyl-β-D-glucopyranosyluronate)-(1→4)]-2-*O*-benzyl-L-fucopyranosyl trichloroacetimidate (110)

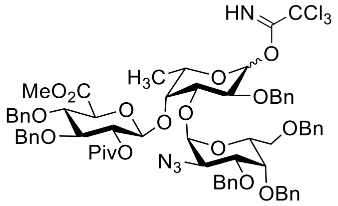

Compound **108** (38 mg, 30 μmol) was dissolved in CH_3CN (2.0 mL) and H_2O (20 μL). NBS (7.0 mg, 40 μmol). was added at room temperature and reaction mixture was stirred for 4 h. Afterwards, the reaction mixture was diluted with a solution of $Na_2S_2O_3$ (10 %) and extracted with EtOAc. The combined organic layer was washed with brine, dried over $MgSO_4$ and concentrated in *vacuo*. Purification by silica gel column chromatography [pentane / EtOAc, 3:1 → 1:1, Rf: 0.26 (pentane / EtOAc, 2:1)] afforded 25 mg (71%) of the corresponding hemiacetal as a pale yellow oil. Further, the hemiacetal was dissolved in CH_2Cl_2 (1.0 mL), Cl_3CCN (42 μL, 0.42 mmol) and added catalytic amount of DBU (0.48 μL, 3.2 μmol). Purification by silica gel column chromatography (pentane / EtOAc, 7:1 → 3:1) furnished 17.6 mg (63%) of **110** (α / β 2:1) as a pale yellow oil.

The analytical data is for the α anomer.

$[\alpha]_D^{25}$ = – 1.4° (c = 0.05, $CHCl_3$).

^{1}H NMR (300 MHz, $CDCl_3$): δ (ppm) 1.06 (d, J = 6.5 Hz, 3 H), 1.17 (s, 9 H), 3.26 (dd, J = 9.7, 4.6 Hz, 1 H), 3.38 (s, 1 H), 3.42 (s, 3 H), 3.53 (q, J = 7.1 Hz, 1 H), 3.65–3.73 (m, 3 H), 3.83– 4.04 (m, 6 H), 4.10 (t, J = 7.1 Hz, 1 H), 4.19 (dd, J = 10.5, 2.5 Hz, 1 H), 4.32–4.48 (m, 3 H), 4.56–4.79 (m, 6 H), 4.84–4.92 (m, 2 H), 5.17 (q, J = 7.5 Hz, 2 H), 5.32 (d, J = 3.8 Hz, 1 H), 6.28 (d, J = 1.9 Hz, 1 H), 7.14–7.53 (m, 30 H), 8.42 (s, 1 H).

^{13}C NMR (125 MHz, $CDCl_3$): δ (ppm) 16.8, 27.2, 27.3, 38.8, 52.2, 59.7, 69.2, 70.2, 70.6, 72.1, 72.9, 73.0, 73.2, 73.3, 74.0, 74.2, 74.9, 74.9, 75.8, 79.8, 82.3, 91.4, 95.0, 99.8, 100.8, 127.3, 127.3, 127.5, 127.5, 127.6, 127.7, 127.7, 127.8, 127.9, 127.9, 128.1, 128.2, 128.3, 128.3, 128.4, 128.5, 128.5, 137.4, 137.8, 138.2, 138.3, 138.4, 138.6, 161.2, 169.0, 176.5.

IR (film): $\tilde{\nu}$ (cm^{-1}) 3341, 3089, 3064, 3031, 2970, 2108, 1744, 1671, 1604, 1479, 1455.

HR-MS (ESI): m/z calcd. for $C_{68}H_{75}Cl_3N_4O_{16}Na^+$ 1333.4112, found: 1333.4154.

n-Pentenyl (2-Azido-3,6-di-*O*-benzyl-2-deoxy-4-*O*-pivaloyl-α-D-galactopyranosyl)(1→3)-[(methyl 3,4-Di-*O*-benzyl-2-*O*-pivaloyl-β-D-glucopyranosyluronate)-(1→4)]-2-*O*-benzyl-β-L-fucopyranoside (109)

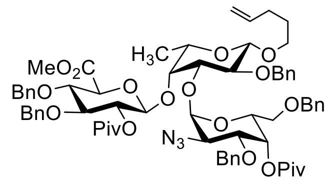

Compound **107** (48.0 mg, 62.0 μmol) and compound **27** (123 mg, 0.180 mmol) were mixed, co-evaporated with toluene (3x), dried in vacuo for 1 h and dissolved in CH_2Cl_2 (2.0 mL). To the cooled soln. at 0 °C, TMSOTf (33.0 μL, 0.180 mmol) was added and reaction mixture was stirred for 2 h. After completion of the reaction pyridine was added and the reaction mixture was evaporated to dryness. The resulting crude product was purified by HPLC (gel permeation chromatography) to furnish 61 mg (80%) of trisaccharide **109** as a colorless oil.

$[\alpha]_D^{20}$ = + 19.4° (c = 0.47, $CHCl_3$).

^{1}H NMR (300 MHz, $CDCl_3$): δ (ppm) 1.09 (d, *J* = 6.5 Hz, 3 H), 1.12 (s, 9 H), 1.20 (s, 9 H), 1.62 (m_c, 2 H), 2.10 (mc, 2 H), 3.30 (q, *J* = 6.5 Hz, 1 H), 3.34–3.44 (m, 5 H), 3.54–3.62 (m, 3 H), 3.69 (s, 3 H), 3.72–3.77 (m, 1 H), 3.80 (d, *J* = 3.0 Hz, 1 H), 3.85–3.86 (m, 1 H), 3.88–3.93 (m, 1 H), 4.19 (d, *J* = 7.5 Hz, 1 H, H–1A), 4.24 (dd, *J* = 10.8, 3.0 Hz, 1 H), 4.35–4.39 (m, 2 H), 4.45–4.48 (m, 3 H), 4.56 (d, *J* = 8.0 Hz, 1 H, H–1C), 4.63 (d, *J* = 10.6 Hz, 1 H), 4.77 (d, *J* = 10.3 Hz, 1 H), 4.80 (d, *J* = 10.6 Hz, 1 H), 4.86 (d, *J* = 10.3 Hz, 1 H), 4.90–5.00 (m, 3 H), 5.21 (t, *J* = 8.0 Hz, 1 H), 5.30 (d, *J* = 3.6 Hz, 1 H, H–1B), 5.60 (d, *J* = 3.0 Hz, 1 H), 5.79 (m_c, 1 H), 7.12-7.42 (m, 25 H).

^{13}C NMR (125 MHz, $CDCl_3$): δ (ppm) 17.1, 27.2, 27.3, 29.1, 30.2, 38.8, 39.0, 52.7, 59.4, 67.3, 68.4, 68.6, 69.8, 69.9, 71.4, 72.8, 73.4, 73.7, 74.3, 74.8, 74.9, 75.0, 75.8, 76.7, 78.9, 79.7, 82.4, 99.7 (C-1B), 101.0 (C-1C), 103.3 (C-1A), 114.6, 127.3, 127.4, 127.5, 127.5, 127.6, 127.7, 127.9, 128.1, 128.1, 128.2, 128.3, 128.3, 128.3, 128.4, 128.4, 137.4, 137.8, 138.1, 138.1, 138.2, 139.0, 169.1, 176.5, 177.9.

IR (ATR): ṽ (cm^{-1}) = 3063, 3030, 2926, 2871, 2360, 2341, 2108, 1746, 1732, 1639, 1496.

HR-MS (ESI): m/z calcd. for $C_{69}H_{85}N_3O_{17}Na^+$ 1250.5771, found: 1250.5762.

2-Azido-3,6-tri-*O*-benzyl-2-deoxy-4-*O*-pivaloyl-α-D-galactopyranosyl-(1→3)-[(methyl-3,4-di-*O*-benzyl-2-*O*-pivaloyl-β-D-glucopyranosyluronate)-(1→4)]-2-*O*-benzyl-L-fucopyranosyl trichloroacetimidate (111)

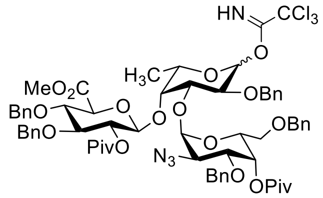

Compound **107** (90 mg, 73 μmol) was dissolved in CH_3CN (5.0 mL) and H_2O (50 μL). NBS (17 mg, 95 μmol) was added at room temperature and reaction mixture was stirred for 4 h. Afterwards, the reaction mixture was diluted with a solution of $Na_2S_2O_3$ (10 %) and extracted with EtOAc. The combined organic layer was washed with brine, dried over $MgSO_4$ and concentrated in *vacuo*. Purification by silica gel column chromatography [pentane / EtOAc, 3:1 → 1:1, Rf: 0.22 (pentane / EtOAc, 3:1)] yielded 55 mg (66%) of the corresponding

hemiacetal as a pale yellow oil. Further, the hemiacetal was dissolved in CH_2Cl_2 (1.0 mL), Cl_3CCN (95 µL, 0.94 mmol) and added catalytic amount DBU (1.0 µL, 7.1 µmol). Purification by silica gel column chromatography (pentane / EtOAc, 7:1 → 3:1) afforded 43 mg (71%) of **111** (α / β 2.5:1) as a pale yellow oil.

The analytical data is for the α anomer.

$[\boldsymbol{\alpha}]_{\mathbf{D}}^{\mathbf{25}}$ = – 2.1° (c = 0.11, $CHCl_3$).

^{1}H NMR (600 MHz, $CDCl_3$): δ (ppm) 1.06 (d, *J* = 6.8 Hz, 3 H), 1.13 (s, 9 H), 1.17 (s, 9 H), 3.38– 3.40 (m, 2 H), 3.44 (dd, *J* = 9.9, 7.5 Hz, 1 H), 3.68 (sbr, 5 H), 3.83 (q, *J* = 8.8 Hz, 2 H), 3.93– 3.97 (m, 2 H), 4.03 (s, 2 H), 4.23 (dd, *J* = 10.5, 3.3 Hz, 1 H), 4.36–4.39 (m, 2 H), 4.55 (d, *J* = 8.0 Hz, 1 H, H–1C), 4.46 (q, *J* = 10.5 Hz, 2 H), 4.62 (d, *J* = 10.5 Hz, 2 H), 4.67–4.81 (m, 5 H), 5.20 (dd, *J* = 10.5, 7.9 Hz, 1 H), 5.30 (d, *J* = 3.4 Hz, 1 H, H–1B), 5.64 (d, *J* = 2.8 Hz, 1 H, H–1A), 7.14–7.40 (m, 25 H), 8.44 (s, 1 H).

^{13}C NMR (125 MHz, $CDCl_3$): δ (ppm) 16.9, 27.2, 27.4, 38.9, 39.1, 52.8, 59.5, 67.3, 68.7, 69.2, 69.9, 71.3, 72.9, 73.3, 73.3, 73.7, 74.3, 74.9, 75.0, 75.5, 75.9, 79.7, 82.3, 91.4, 94.9 (C-1A), 99.7 (C–1B), 100.8 (C–1C), 127.2, 127.3, 127.4, 127.5, 127.6, 127.6, 127.7, 127.8, 127.8, 127.8, 128.0, 128.1, 128.2, 128.2, 128.3, 128.3, 137.3, 137.7, 137.9, 138.0, 138.1, 161.1, 169.0, 176.4, 177.7.

IR (ATR): $\tilde{\nu}$ (cm^{-1}) = 3341, 3089, 3064, 3031, 2970, 2930, 2871, 2108, 1744, 1730, 1671.

HR-MS (ESI): m/z calcd. for $C_{66}H_{77}Cl_3N_4O_{17}Na^+$ 1327.4217, found: 1327.4235.

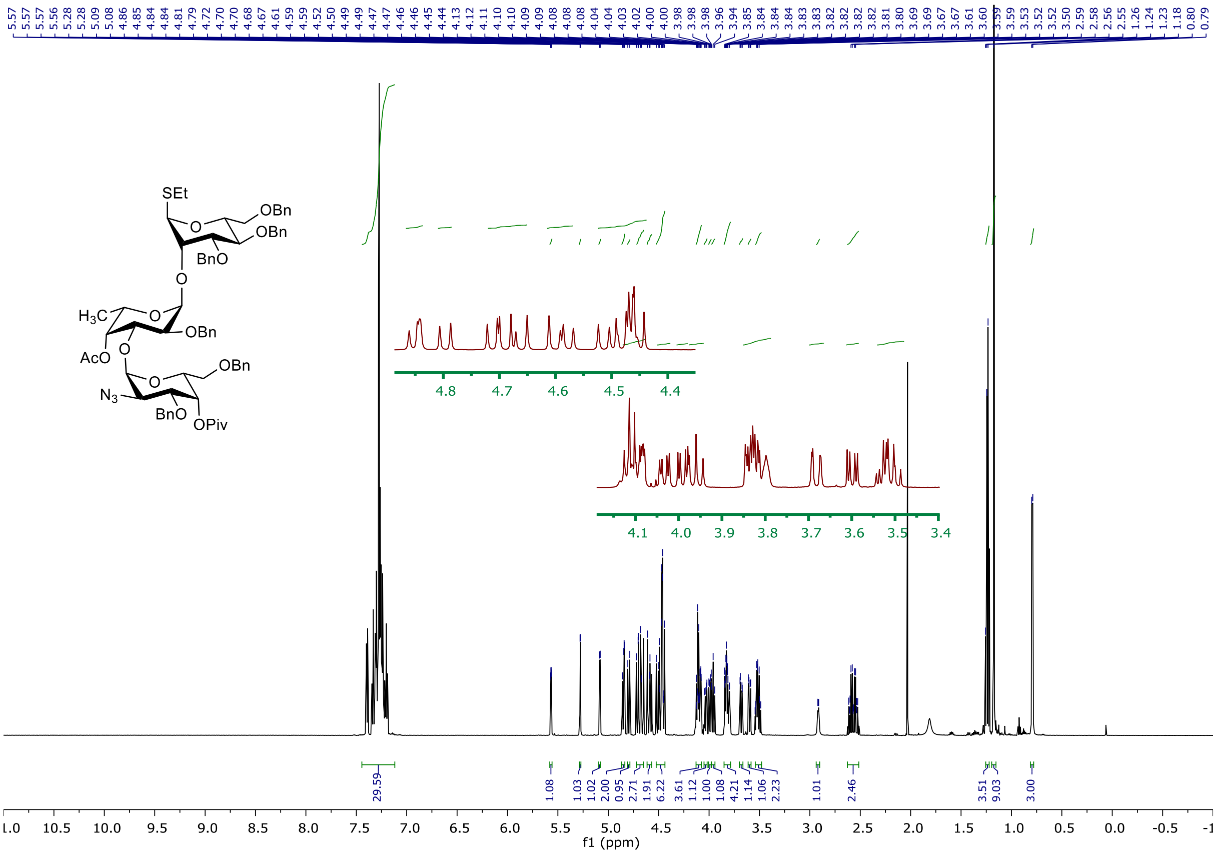

Figure S72. ^{1}H NMR ($CDCl_3$, 600 MHz) spectrum of **trisaccharide 101**

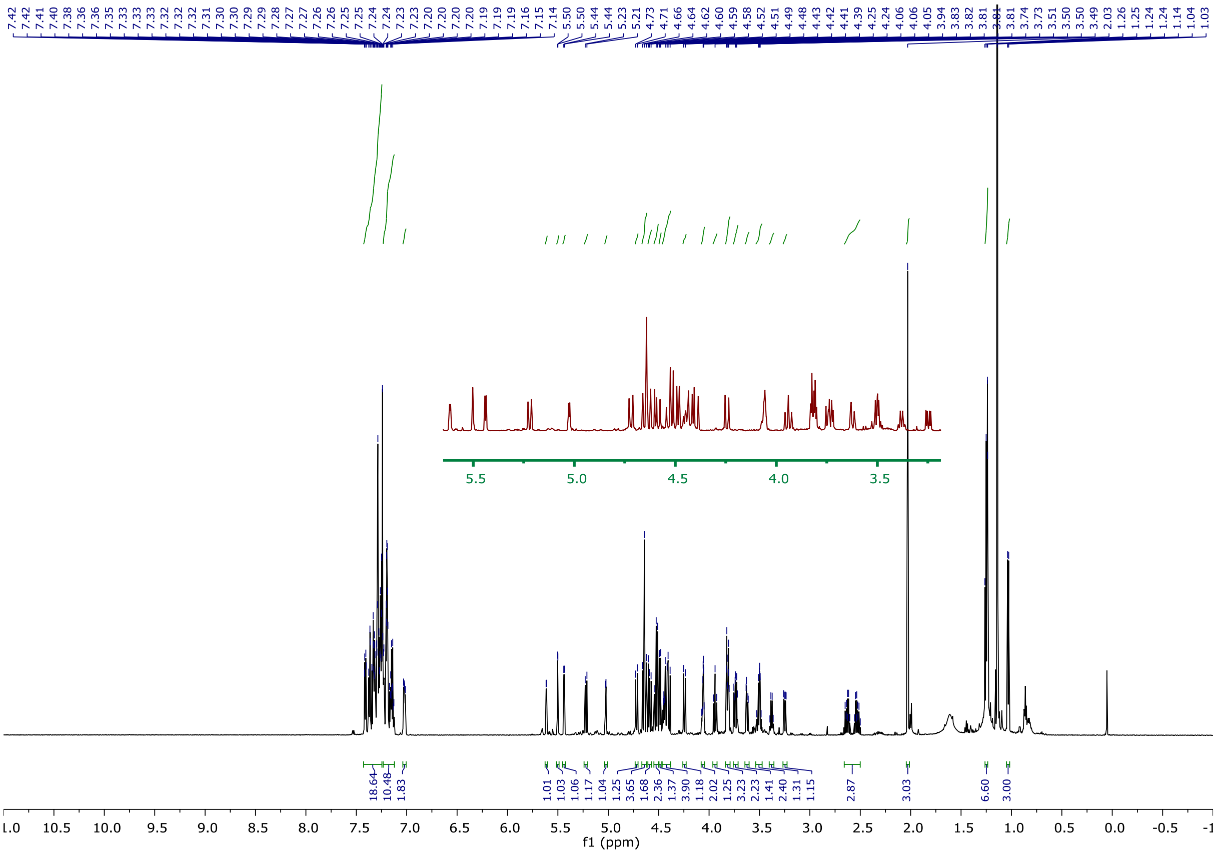

Figure S73. ^{13}C NMR ($CDCl_3$, 125 MHz) spectrum of **trisaccharide 101**

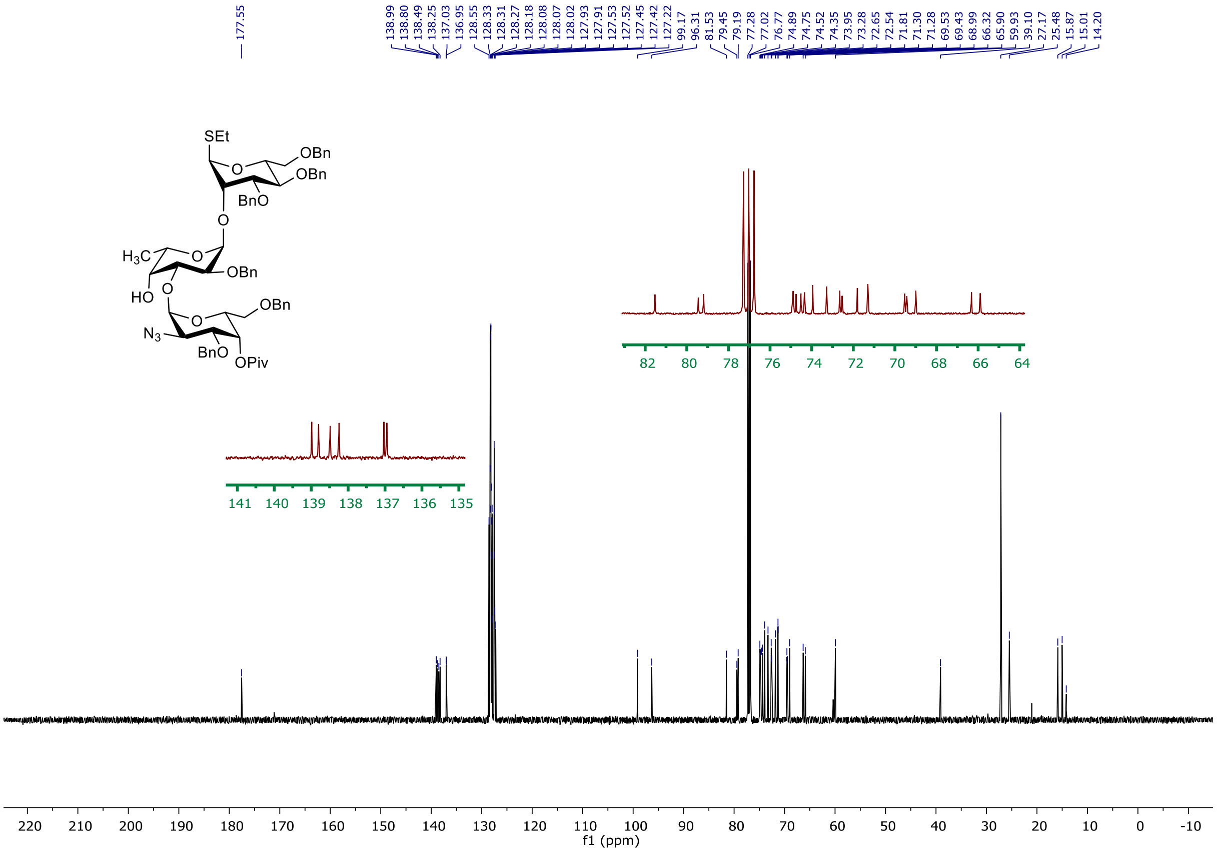

Figure S74. ^{1}H NMR ($CDCl_3$, 600 MHz) spectrum of **trisaccharide acceptor 102**

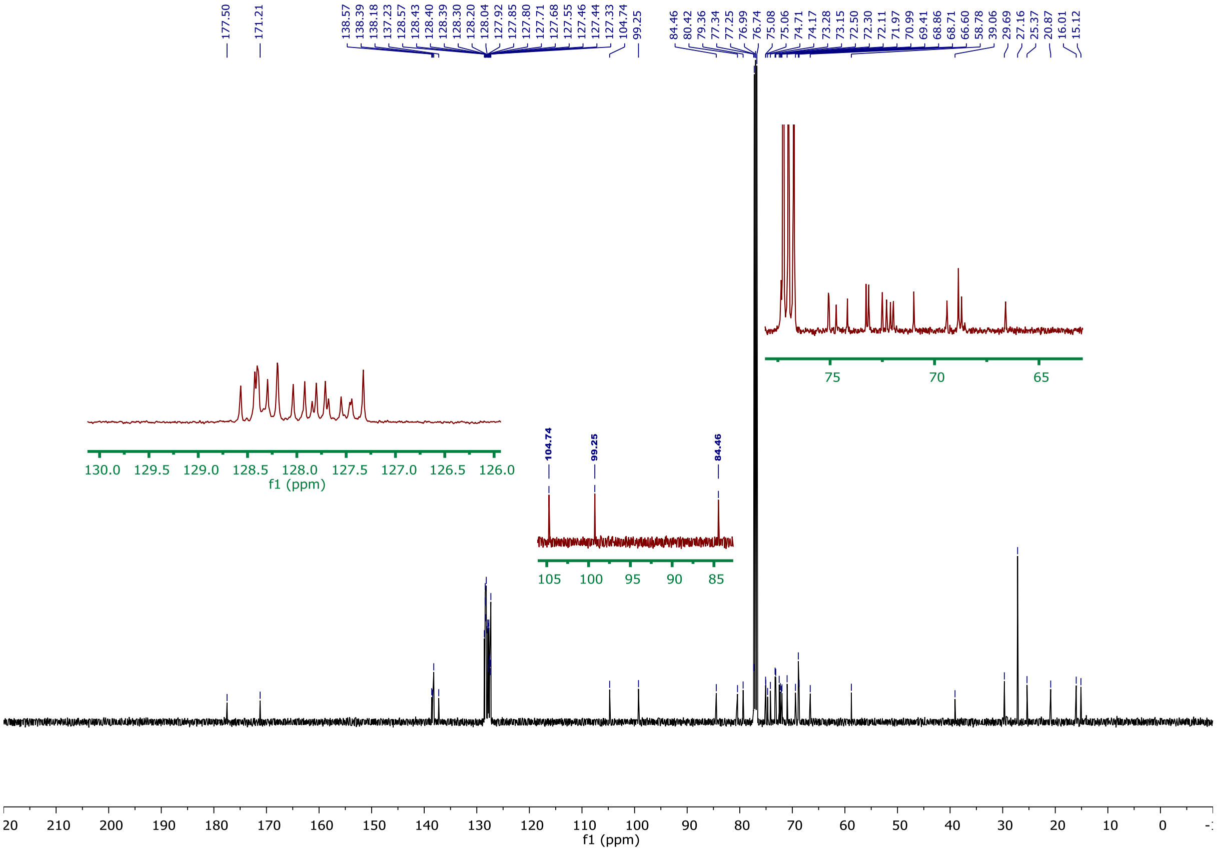

Figure S75. ^{13}C NMR ($CDCl_3$, 125 MHz) spectrum of **trisaccharide acceptor 102**

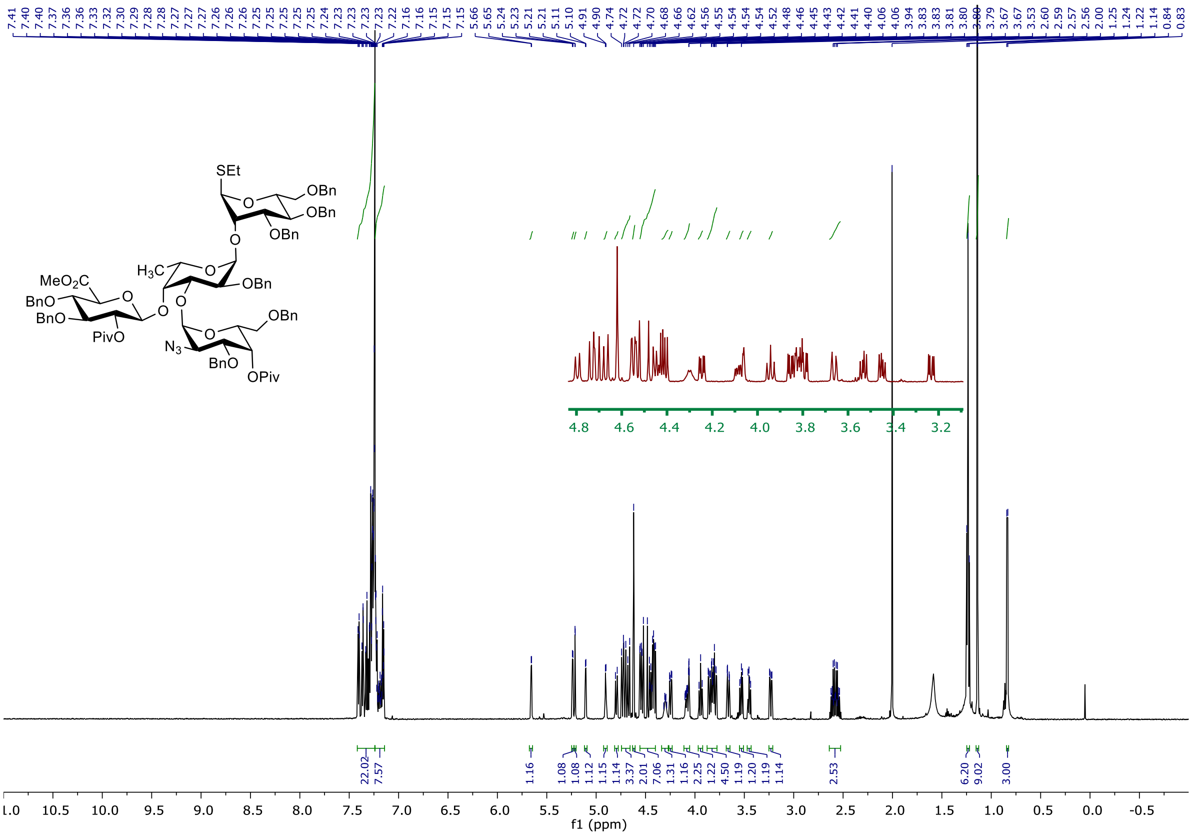

Figure S76. ^{1}H NMR ($CDCl_3$, 600 MHz) spectrum of **tetrasaccharide 104**

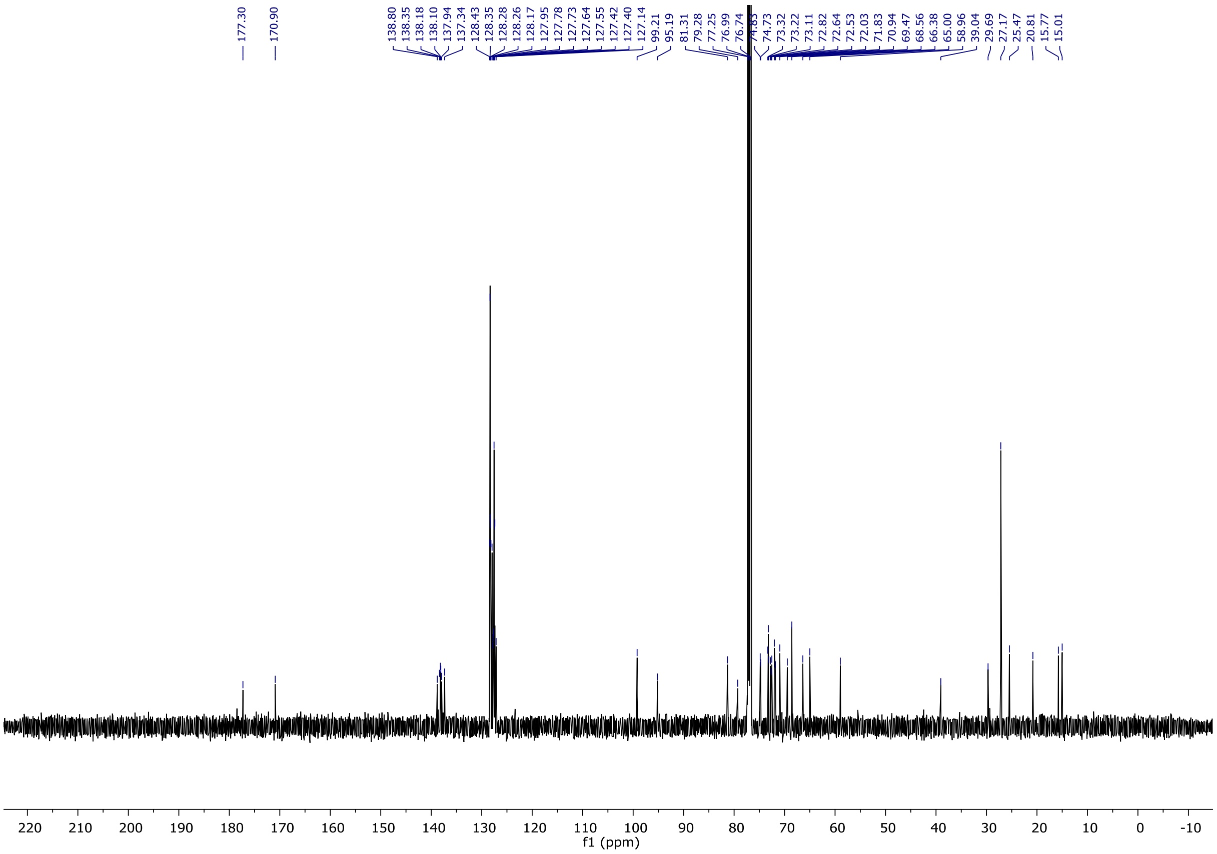

Figure S77. ^{13}C NMR ($CDCl_3$, 125 MHz) spectrum of **tetrasaccharide 104**

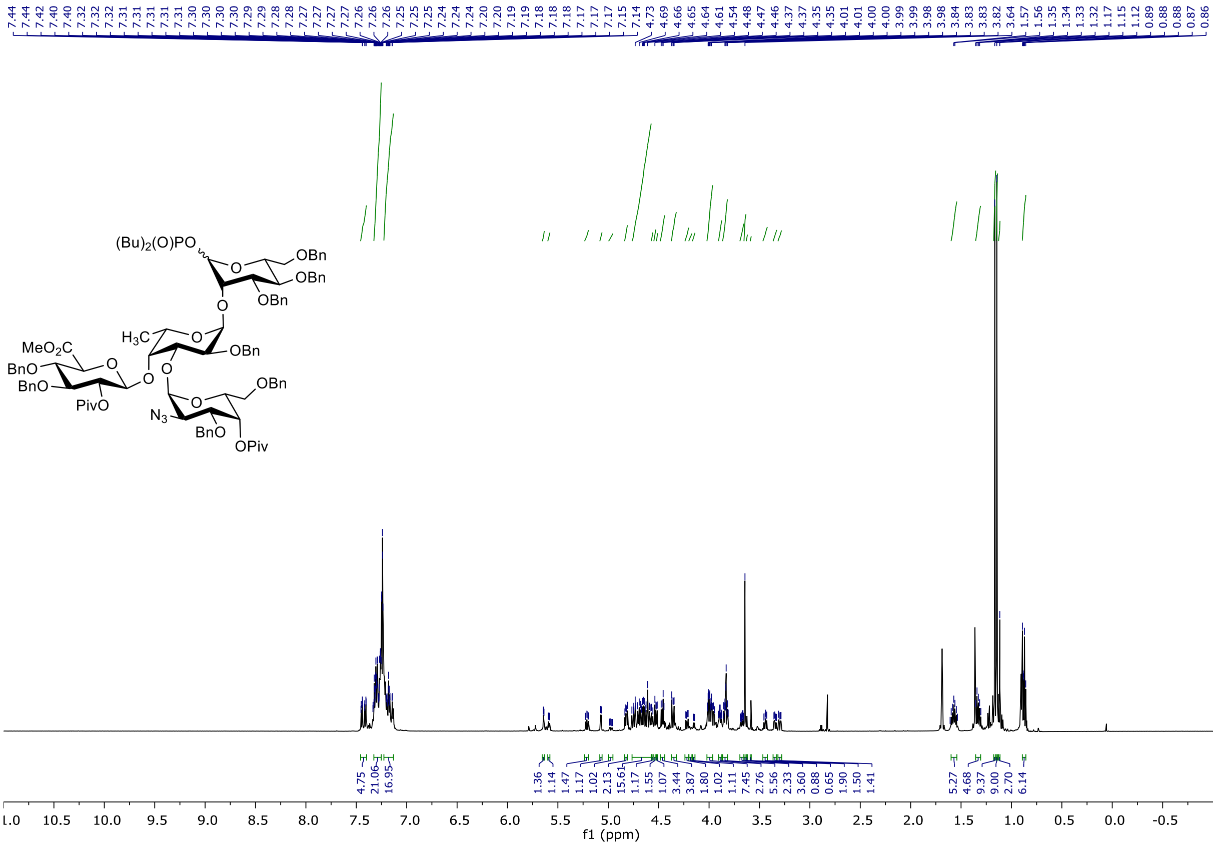

Figure S78. ^{1}H NMR ($CDCl_3$, 600 MHz) spectrum of **tetrasaccharide donor 105**

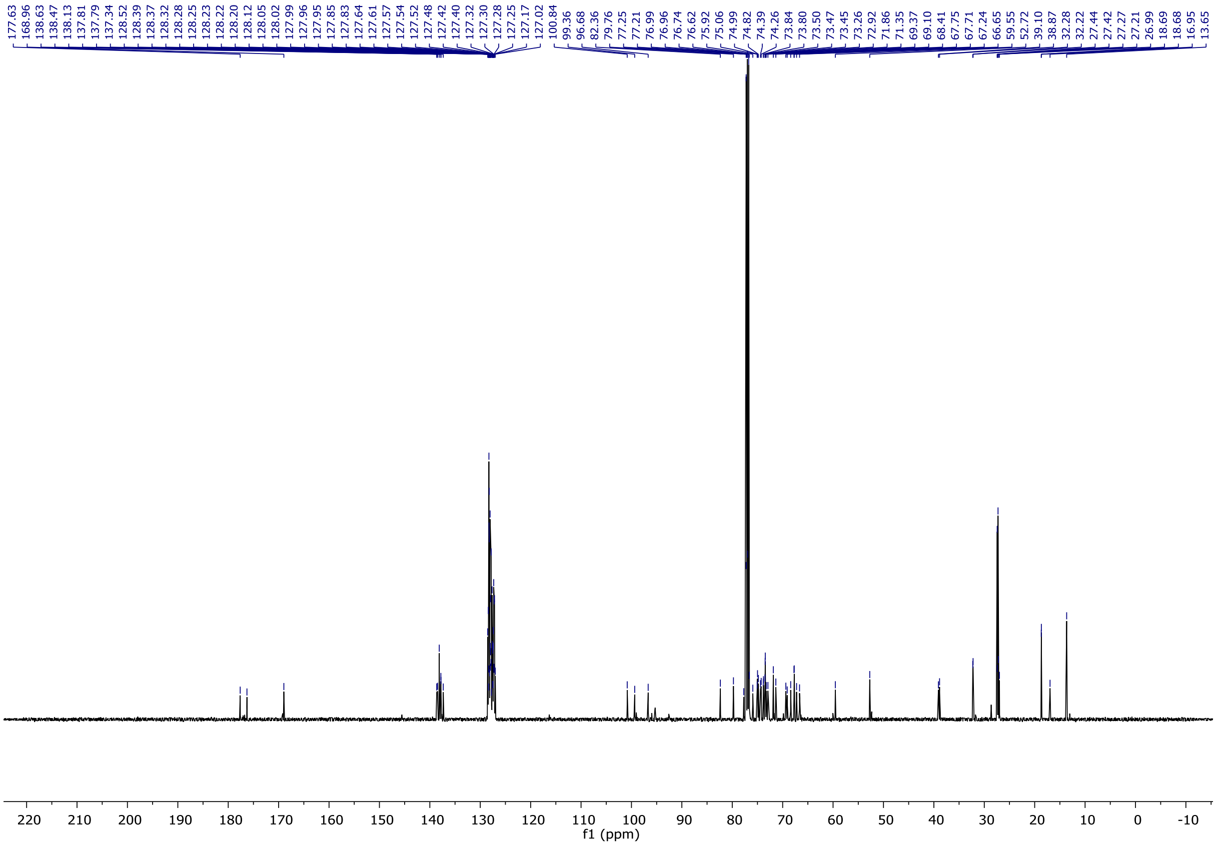

Figure S79. ^{13}C NMR ($CDCl_3$, 125 MHz) spectrum of **tetrasaccharide donor 105**

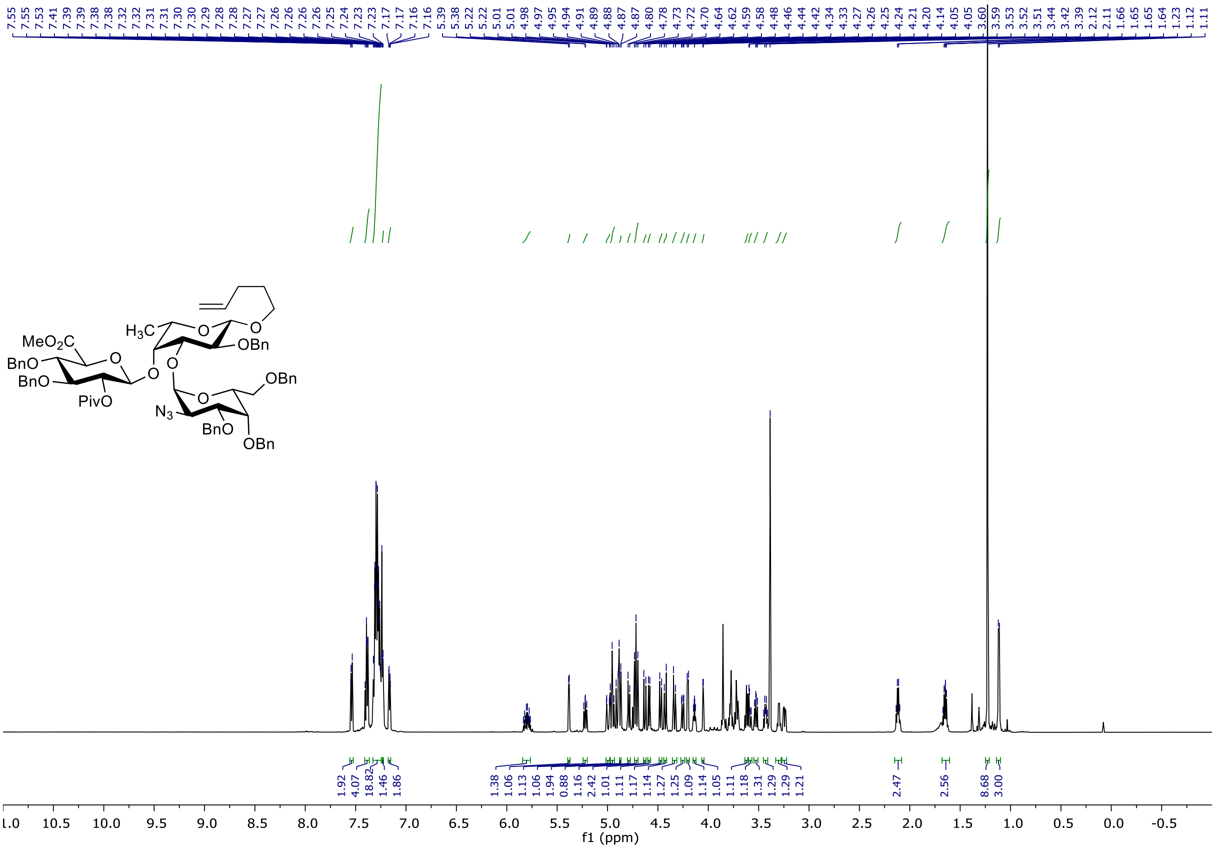

Figure S80. ^{1}H NMR ($CDCl_3$, 300 MHz) spectrum of **trisaccharide 108**

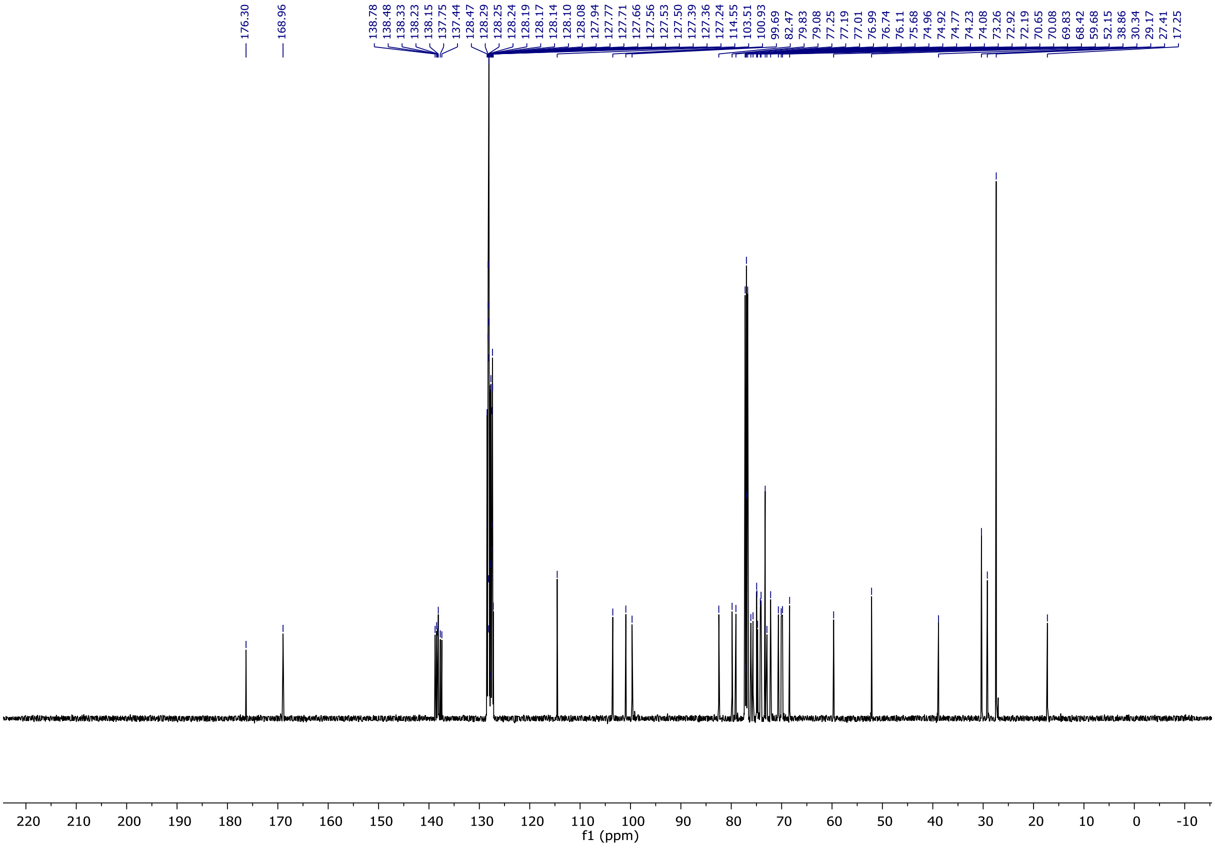

Figure S81. ^{13}C NMR ($CDCl_3$, 125 MHz) spectrum of **trisaccharide 108**

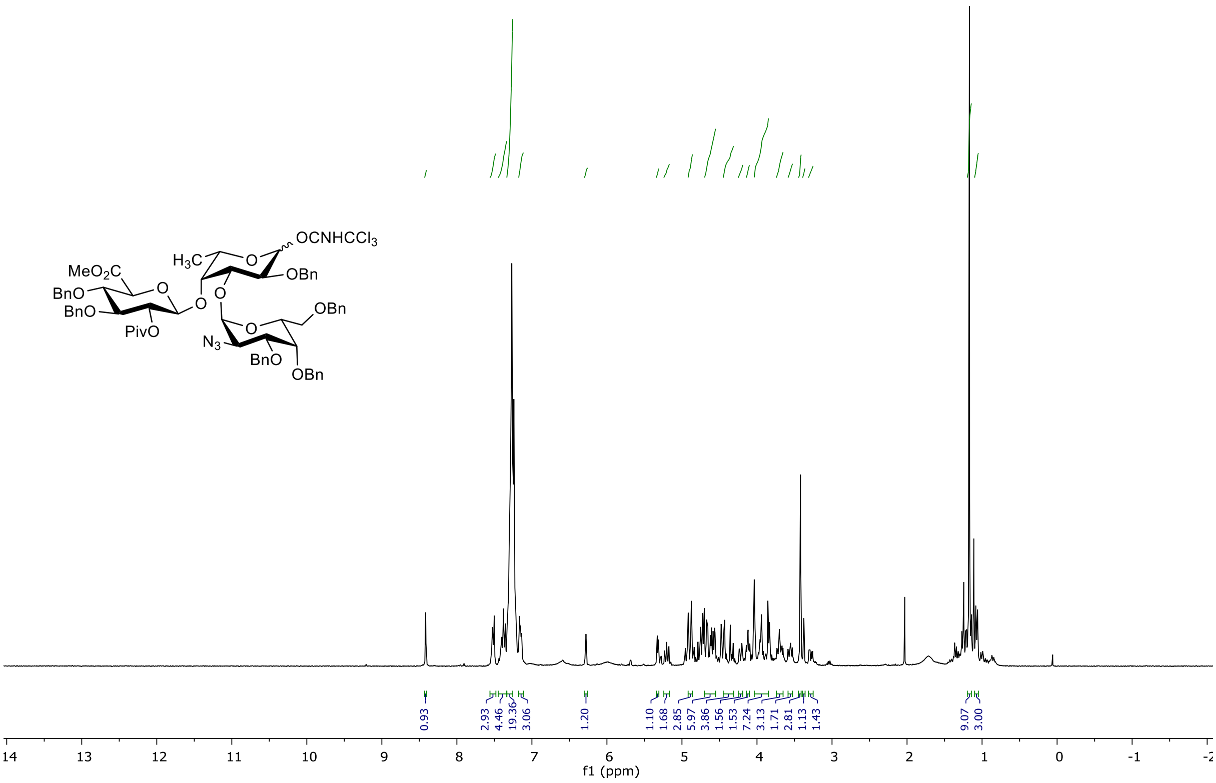

Figure S82. ^{1}H NMR ($CDCl_3$, 300 MHz) spectrum of **trisaccharide donor 110**

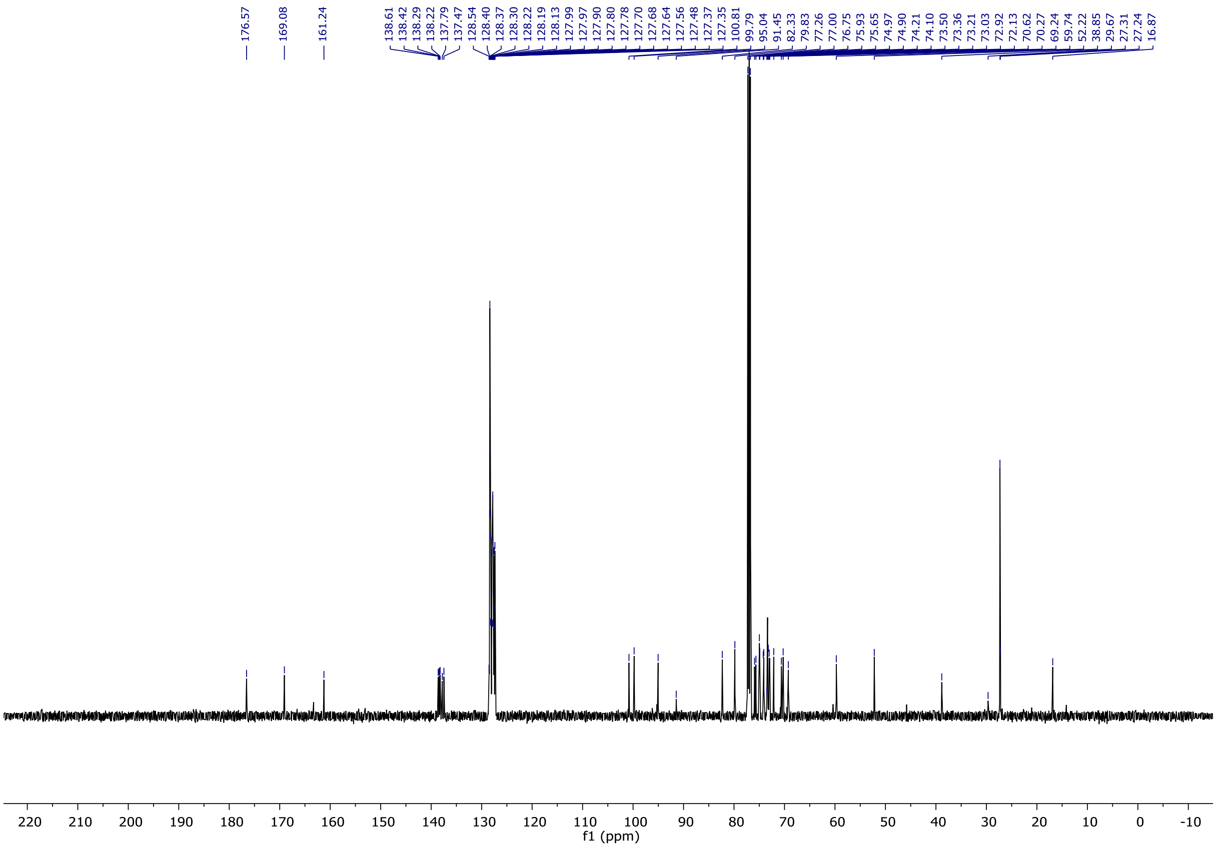

Figure S83. ^{13}C NMR ($CDCl_3$, 125 MHz) spectrum of **trisaccharide donor 110**

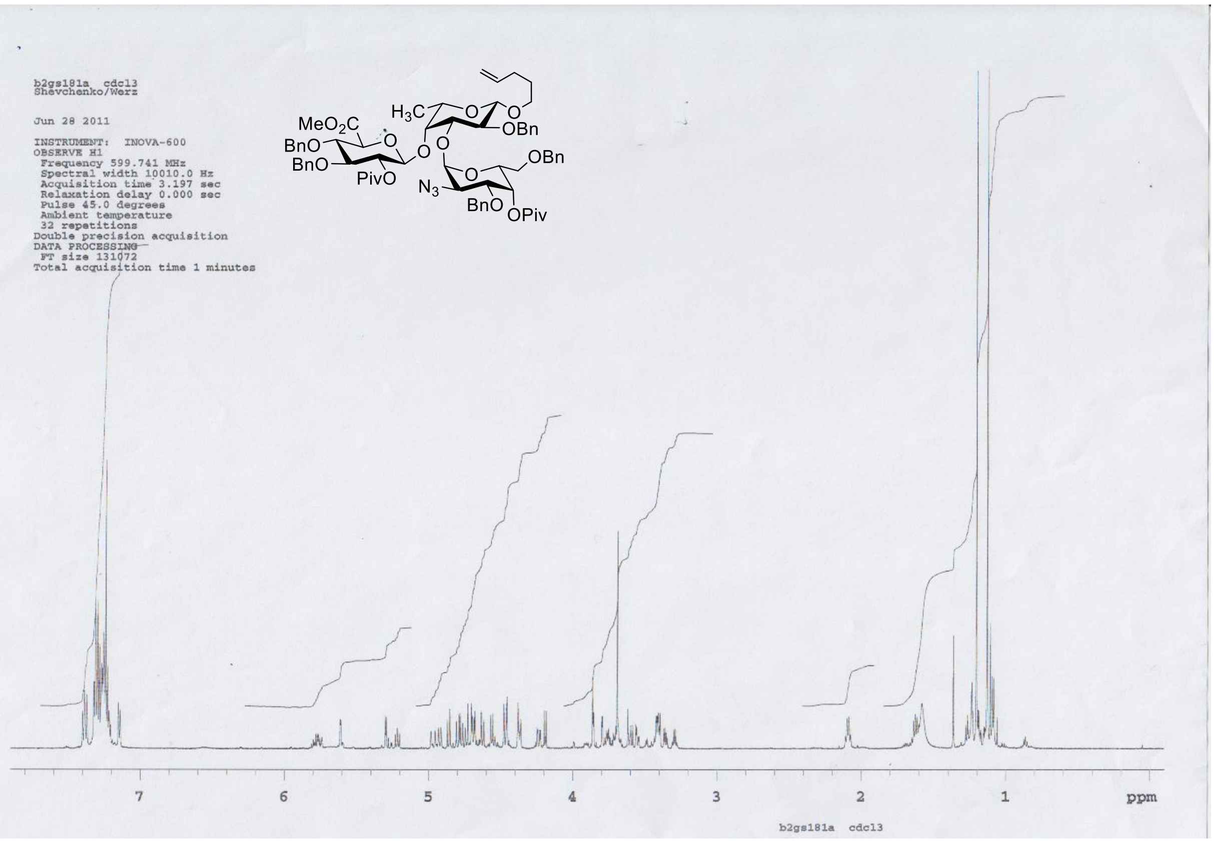

Figure S84. ^{1}H NMR ($CDCl_3$, 300 MHz) spectrum of **trisaccharide 109**

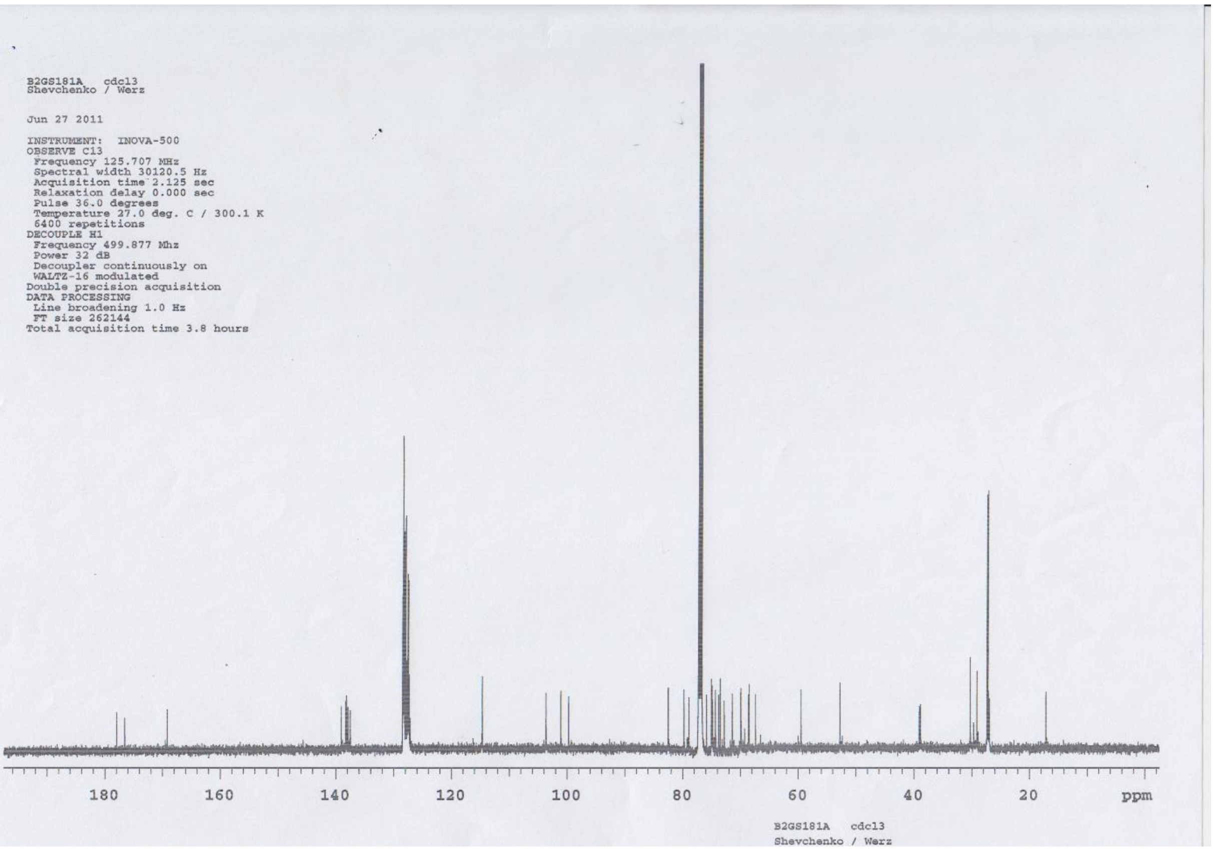

Figure S85. ^{13}C NMR ($CDCl_3$, 125 MHz) spectrum of **trisaccharide 109**

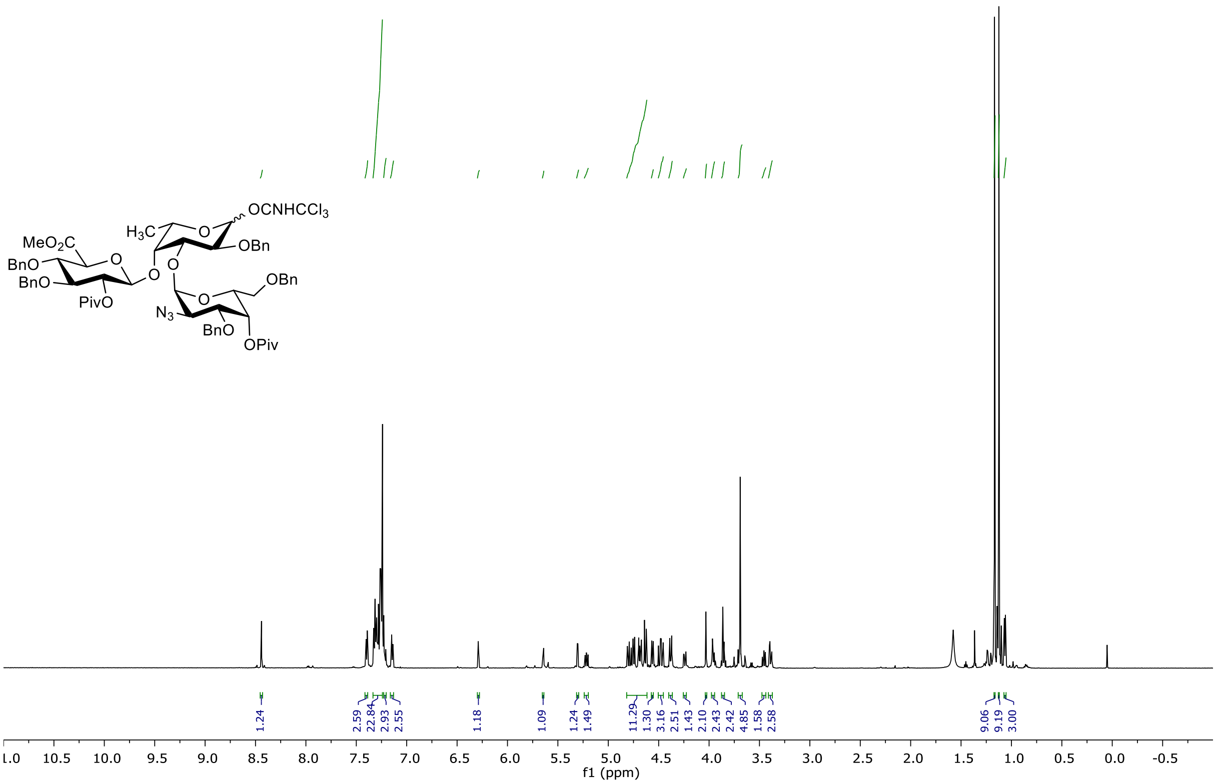

Figure S86. ^{1}H NMR ($CDCl_3$, 600 MHz) spectrum of **trisaccharide donor 111**

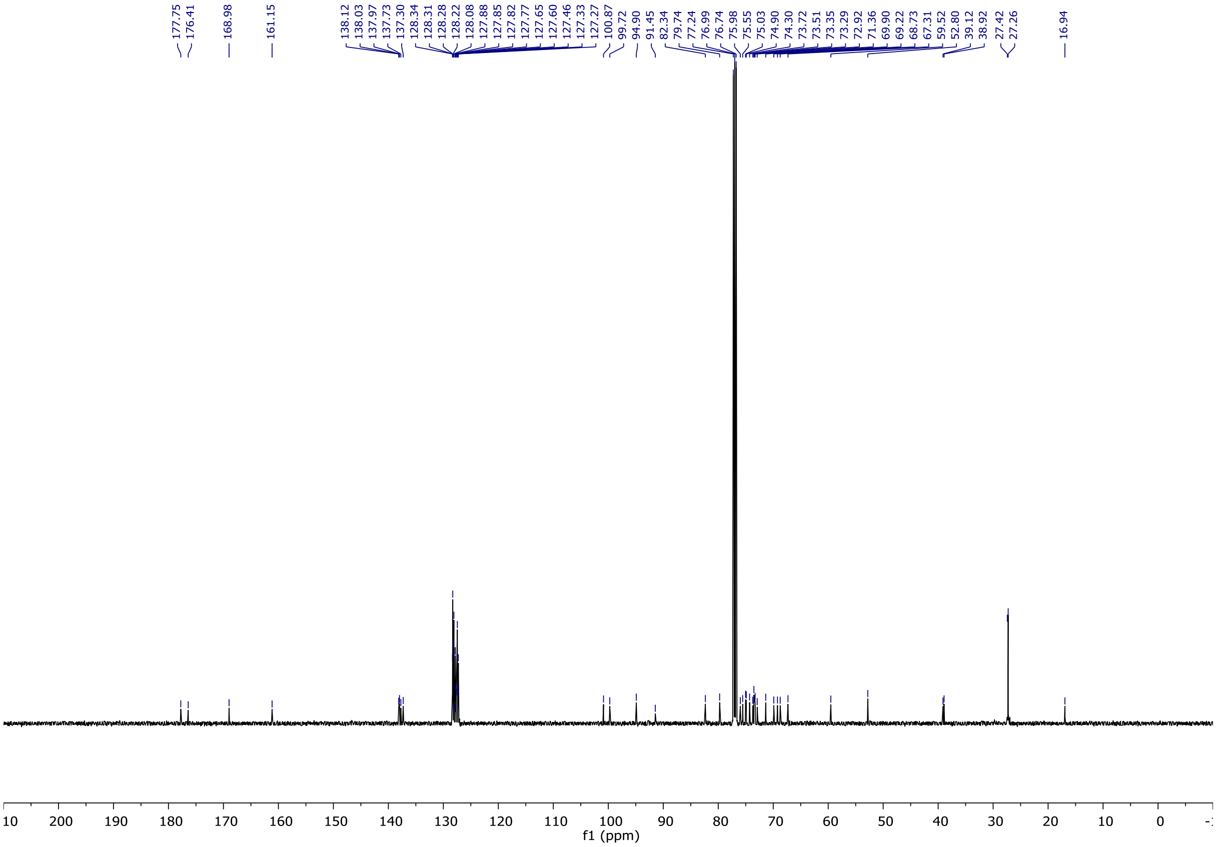

Figure S87. ^{13}C NMR ($CDCl_3$, 125 MHz) spectrum of **trisaccharide donor 111**

13. Table of attempted assembly of heptasaccharide by [4+3] coupling.

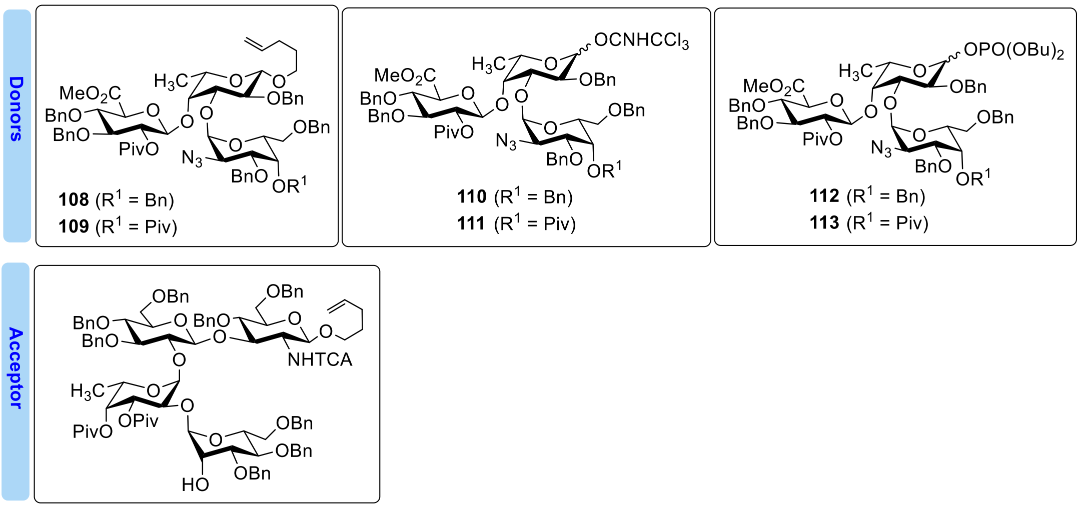

No.	Conditions	Outcome
1.	**110**, TMSOTf (cat.), - 45 °C, 2 h	No product
2.	**110**, TMSOTf (cat.), - 20 °C, 2 h	No product
3.	**110**, TMSOTf (cat.), 25 ºC, 1 h	No product
4.	**110**, TMSOTf (cat.), 0 °C, 2 h	No product
5.	**110**, $BF_3 \cdot OEt_2$ (cat.), 0 °C, 2 h	No product
6.	**110**, TESOTf (cat.), 0 °C, 3 h	No product
7.	**111**, TMSOTf (cat.), - 45 °C, 2 h	No product
8.	**111**, TMSOTf (cat.), - 20 °C, 2	No product
9.	**111**, TMSOTf (cat.), 25 ºC, 1 h	No product
10.	**111**, TMSOTf (cat.), 0 °C, 2 h	No product
11.	**111**, $BF_3 \cdot OEt_2$ (cat.), 0 °C, 2 h	No product
12.	**111**, TESOTf (cat.), 0 °C, 3 h	No product
13.	**112**, TMSOTf (quant.), 0 °C, 2 h	No product
14.	**112**, TMSOTf (quant.), - 10 °C, 2 h	No product
15.	**113**, TMSOTf (quant.), 0 °C, 2 h	No product
16.	**113**, TMSOTf (quant.), - 10 °C, 2 h	No product
17	**108**, IDCP (quant.), 0 °C, 2 h	No product
18	**109**, IDCP (quant.), 0 °C, 2 h	No product

14. Table of attempted assembly of heptasaccharide by [3+4] coupling.

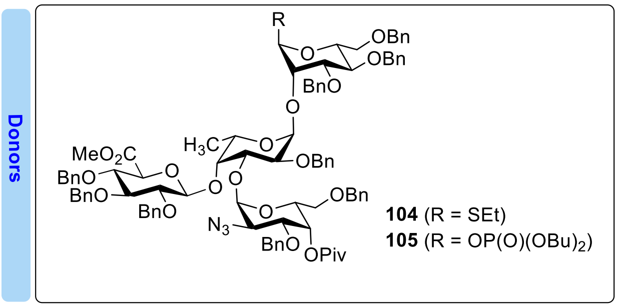

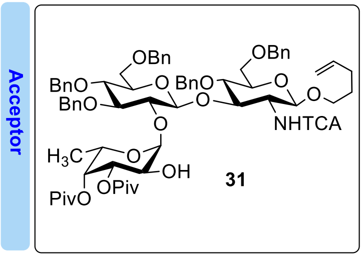

No.	Acceptor	Donor	Conditions	Outcome
**7.**	**31**	**104**	Et_2O, MeOTf, TTBP, 4Å MS, 0 °C, 24 h	No product
**8.**	**31**	**104**	CH_2Cl_2, MeOTf, TTBP, 4Å MS, 25 ºC, 16 h	No product
**9.**	**31**	**104**	CH_2Cl_2, MeOTf, TTBP, 4Å MS, 25 ºC, 24 h	No product
**10.**	**31**	**104**	CH_2Cl_2, DMTST, TTBP, 4Å MS, -10 °C → 0 °C, 24 h	No product
**11.**	**31**	**104**	CH_2Cl_2, DMTST, TTBP, 4Å MS, 0 °C → 25 ºC, 24 h	No product
**12.**	**31**	**104**	CH_2Cl_2, DMTSB, TTBP, 4Å MS, -10 °C → 0 °C, 24 h	No product
**13.**	**31**	**104**	CH_2Cl_2, DMTSB, TTBP, 4Å MS, 0 °C → 25 ºC, 24 h	No product
**14.**	**31**	**105**	CH_2Cl_2, TMSOTf (quant.), 4Å MS, -42 °C, 2 h	No product
**15.**	**31**	**105**	CH_2Cl_2, TMSOTf (quant.), 4Å MS, -21 °C, 2 h	No product
**16.**	**31**	**105**	CH_2Cl_2, TMSOTf (quant.), 4Å MS, 0 °C, 3 h	No product

15. Table of attempted assembly of tetrasaccharide by [3+1]-coupling.

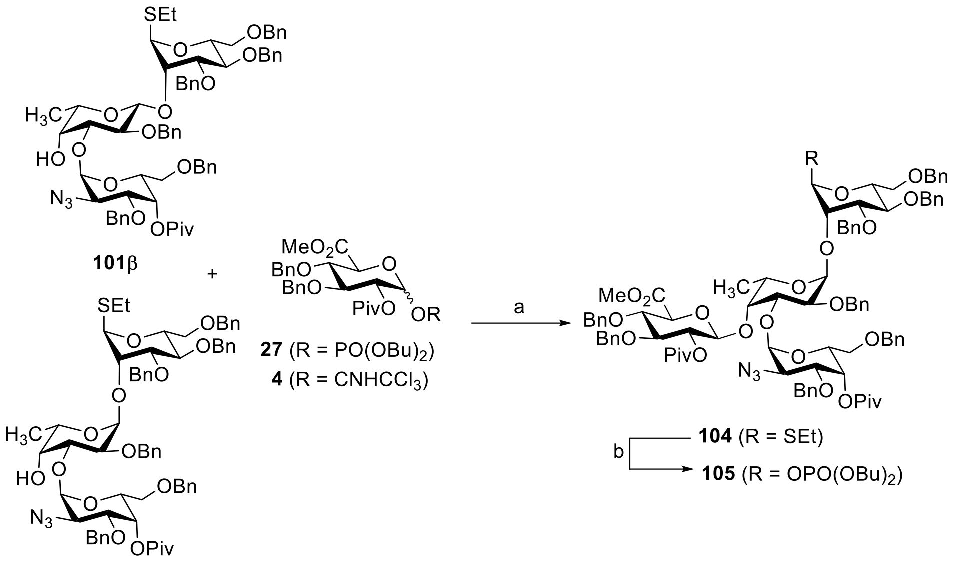

No.	Conditions	Yield% (Isolated)	Yield% (brsm)
**1.**	**101**β, **27**, TMSOTf (quant.), 0 °C, 2 h	64	68
**2.**	**101**α, **27**, TMSOTf (quant.), 0 °C, 2 h	33	38
**3.**	**101**α, **27**, TMSOTf (quant.), 0 °C, 2.5 h	35	37
**4.**	**101**α, **27**, TMSOTf (quant.), 0 °C, 3 h	33	33
**5.**	**101**α, **27**, TMSOTf (quant.)*, -10 °C, 2 h	40	60
**6.**	**101**α, **27**, TMSOTf (quant.)*, -10 °C, 3 h	**54**	**67**
**7.**	**101**α, **4**, TMSOTf (cat.), 0 °C, 2 h	20	25
**8.**	**101**α, **4**, TMSOTf (cat.), 0 °C, 3 h	22	26
**9.**	**101**α, **4**,TMSOTf (cat.), 0 °C, 5 h	20	20

*A diluted solution of TMSOTf in DCM was added very slowly

16. Table of optimization of reaction conditions for the [6+1]-coupling.

No.	Conditions	Yield (Isolated)	Yield (brsm)
**1.**	**4** (4 eq), $BF_3.OEt_2$ (0.15 eq), 0 °C, 3 h	5%	21%
**2.**	**4** (4 eq), TMSOTf (0.15 eq), -15 °C, 3 h	9%	53%
**3.**	**4** (4 eq), TMSOTf (0.15 eq), 0 °C, 3 h	20%	32%
**4.**	**27** (4 eq), TMSOTf (4 eq), 0 °C, 3 h	5%	7%
**5.**	**4** (4 eq), TESOTf (0.15 eq)*, 0 °C, 3 h	14%	98%
**6.**	**4** (4 eq), TESOTf (0.15 eq)*, 25 ºC, 3 h	20%	21%
**7.**	**4** (4 eq), TESOTf (0.15 eq)*, 0 °C, 16 h	25%	45%
**8.**	**4** (4 eq), TESOTf (0.15 eq)*, 0 °C, 24 h	**54%**	**64%**
**9.**	**4** (4 eq), TESOTf (0.15 eq)*, 0 °C, 48 h	46%	48%
**10.**	**4** (4 eq), TBSOTf (0.15 eq), 0 °C, 24 h	10%	60%

* A diluted solution of TESOTf in DCM was added slowly.

17. Table of optimization of hydrolysis of methyl ester 97.

No.	Conditions	Yield % (Isolated)	Yield % (brsm)
**1.**	LiOH (1N), H_2O_2 (30%), 25 ºC, 24 h	20	60
**2.**	LiOH (1N), H_2O_2 (30%), 40 °C, 48 h	25	63
**3.**	LiOH (1N), H_2O_2 (30%), NaOH (4N), r.t. 24 h	27	67
**4.**	KOH (3M), MeOH, 25 ºC, 24 h	29	60
**5.**	KOH (3M), MeOH, 40 °C, 5.5 d	27	55
**6.**	KOH (0.5M)-H_2O_2 (30%), 25 ºC, 24 h	27	65
**7.**	KOH (0.5M)-H_2O_2 (30%), 25 ºC, 2.5 d	25	40
**8.**	KOH (0.5M)-H_2O_2 (30%), 50 °C, 24 h	29	37
**9.**	K_2CO_3 (2%), 25 ºC, 2 d	29	35

18. Chromatogramm of recycling preparative HPLC (LC-9101) for purification of heptasaccharide.

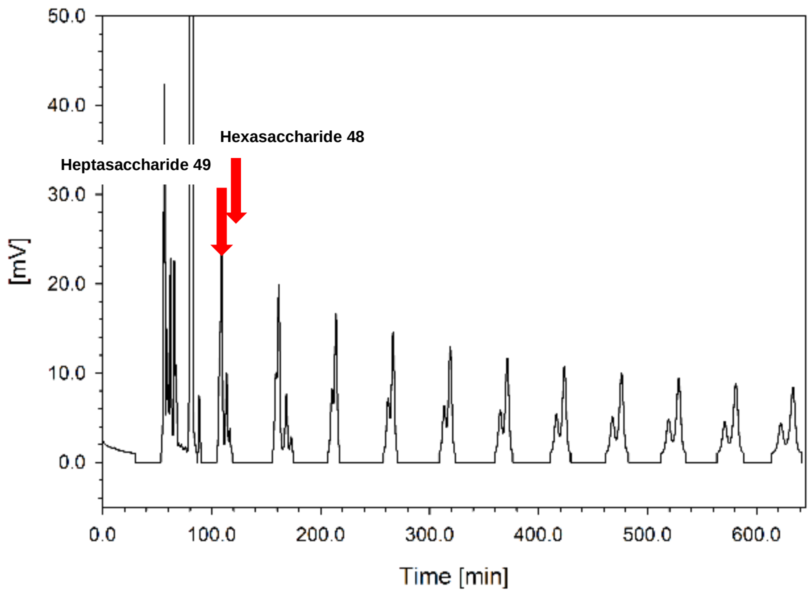

References

[1] T. Horlacher, M. A. Oberli, D. B. Werz, L. Kröck, S. Bufali, R. Mishra, J. Sobek, K. Simons, M. Hirashima, T. Niki, P. H. Seeberger, *ChemBioChem.* **2010**, *11*, 1563-1573.

[2] L. G. Melean, K. R. Love, P. H. Seeberger, *Carbohydr. Res*. **2002**, *337*, 1893–1916.

[3] M. Sakagami, H. Hamana, *Tetrahedron Lett*. **2000**, 5547–5551.

[4] D. J. Silva, H. Wang, N. M. Allanson, R. K. Jain, M. J. Sofia, *J. Org. Chem.* **1999**, *64*, 5926–5929.

[5] G. Blatter, J.-M. Beau, J.-C. Jacquinet, *Carbohydr. Res.* **1994**, *260*, 189–202.

[6] T. K. Lindhorst, J. Thiem, *Liebigs Ann. Chem*. **1990**, *12*, 1237-1241.

[7] B. Ruttens, P. Kováč, *Synthesis* **2004**, *15*, 2505-2508.

[8] S. Boonyarattanakalin, X. Liu, M. Michieletti, B. Lepenies, P. H. Seeberger, *J. Am. Chem. Soc*. **2008**, *130*, 16791–16799;

[9] A. Ravidà, X. Liu, L. Kovacs, P. H. Seeberger, *Org. Lett*. **2006**, *8*, 1815-1818;

[10] G. Soldaini, F. Cardona, A. Goti, *Org. Lett*. **2005**, *7*, 725-728;

[11] J. Banoub, P. Boullanger, M. Potier, G. Descotes, *Tetrahedron Lett*. **1986**, *27*, 4145-4148;

[12] N. E. Franks, R. Montgomery, *Carbohydr. Res*. **1968**, *6*, 286-298;

[13] R. U. Lemieux, A. R. Morgan, *Can. J. Chem*. **1965**, *43*, 2198-2204;

[14] A. S. Perlin, *Can. J. Chem*. **1963**, *41*, 399-406.

[15] O. P. Dhamale, C. Zong, K. Al-Mafraji, G. -J. Boons, *Org. Biomol. Chem*., **2014**, *12*, 2087–2098;

[16] M. Kojima, Y. Nakamura, K. Komori, S. Akai, K -C. S. Takeuchi; *Tetrahedron* **2011**, *67*, 8276-8292

[17] N. Karst, J.-C. Jacquinet, *J. Chem. Soc., Perkin Trans. 1*, **2000**, *16*, 2907-2917

[18] S. David, A. Malleron, C. Dini, *Carbohydr. Res.* **1989**, *188*, 193-200.
